# Innovative On‐Resin and in Solution Peptidomimetics Synthesis via Metal‐Free Photocatalytic Approach

**DOI:** 10.1002/chem.202402790

**Published:** 2024-11-08

**Authors:** Tommaso Gandini, Francesco Vaghi, Zoe Laface, Giovanni Macetti, Alberto Bossi, Marta Penconi, Maria Luisa Gelmi, Raffaella Bucci

**Affiliations:** ^1^ Dipartimento di Scienze Farmaceutiche Sez. Chimica Generale e Organica A. Marchesini Università degli Studi di Milano Via Venezian 21 20133 Milan Italy; ^2^ Dipartimento di Scienze Chimiche Università degli Studi di Padova Via Marzolo 1 35131 Padova Italy; ^3^ Dipartimento di Chimica Università degli Studi di Milano Via Golgi 19 20133 Milan Italy; ^4^ Istituto di Scienze e Tecnologie Chimiche ‘G. Natta' (SCITEC) Centro Nazionale Ricerche Via Fantoli 16/15 20138 Milano Italy

**Keywords:** Peptidomimetics, Photocatalysis, Solid phase peptide synthesis, Carbamoylation

## Abstract

Nowadays, peptidomimetics are widely studied, being useful tools in drug discovery and medicinal chemistry. The coupling between a carboxylic acid with an amine to form a peptide bond is the most common reaction to obtain peptides/peptidomimetics. In this work, we have investigated an innovative metal‐free photoredox‐catalyzed carbamoylation to form peptidomimetics thanks to the reaction between dihydropyridines functionalized with amino acids (or peptide sequences) and differently functionalized imines. As the organic photocatalyst, we used 4CzIPN, a donor‐acceptor cyanoarene vastly used in photoredox catalysis. By easily modulating the amino acid (or peptide sequence), which is directly attached to the dihydropyridine, we proposed this key‐reaction as new valuable method to obtain peptidomimetics, in situ building the not‐natural portion of the sequence. Moreover, we successfully employed this methodology in solid phase peptide synthesis, both inserting the new photoredox‐generated amino acid at the end or in the middle of the sequence. Peptides with different lengths and secondary structures were prepared, proving the success of this approach, even in sterically hindered environment. Herein, to the best of our knowledge, we describe the first photocatalytic protocol which allows the building of the peptide backbone, with the possibility of simultaneously inserting a non‐coded amino acid in the sequence.

## Graphical abstract

In this work we studied an innovative peptidomimetic synthesis via metal‐free photoredox approach, exploiting the reaction between dihydropyridines functionalized with amino acids (or peptide sequences) and differently functionalized imines. Furthermore, we successfully employed this methodology on solid phase peptide synthesis, obtaining peptides of different lengths and secondary structures, proving the success of this approach, even in a sterically hindered environment.

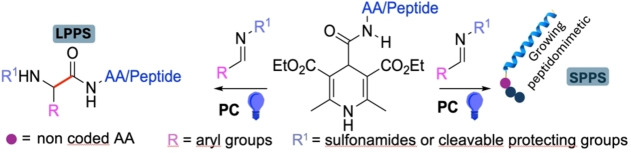

## Introduction

Peptides, with their peculiar features, represent versatile tools for scientists. Their huge diversity in structure and in biological functions makes them the perfect candidates for a plethora of applications (*i. e*. material science,[^1^, ^2^, ^3^] catalysis[^4^, ^5^] and biomedicine[^6^, ^7^]). However, in the context of biological applications, peptides can show unfavorable immune responses and have low proteolytic stability, which are serious limitations to their bench‐to‐bedside translation.^[8]^ To overcome these drawbacks, peptidomimetics are considered as their promising substitutes.[^9^, ^10^] Indeed, they are peptide analogues with unnatural backbone and could be prepared simply inserting a not‐natural amino acid (AA) or a non‐amino acidic scaffold in the sequence.[^8^, ^11^] The properly designed non‐coded AA can thus increase the conformational[^12^, ^13^] and proteolytic stability[^14^, ^15^, ^16^] of the system making peptidomimetics valuable tools for biological applications. It is known that the sulfonamide is a notable amide isostere, having similar geometry to the amide, but with an additional hydrogen bond acceptor, leading to the increased polar surface area.[^17^, ^18^]

In this context, sulfonamide‐containing compounds are known for their broad range of biological activities, such as anti‐inflammatory or antibiotic to name a few.^[19]^ Different examples of peptidomimetics containing the sulfonamide moiety are reported in the literature[^20^, ^21^, ^22^, ^23^] and recently the MacMillan research group reported on a one pot photocatalytic approach to obtain sulfonamides through copper ligand‐to‐metal charge transfer.^[24]^ It has to be underlined that the most common strategy to prepare sulfonamides consists in the use of activated sulfonyl derivatives, generally a chloride that generates hydrochloric acid, which needs to be scavenged. Of relevance, this protocol is not compatible with solid phase peptide synthesis (SPPS).

**Scheme 1:**
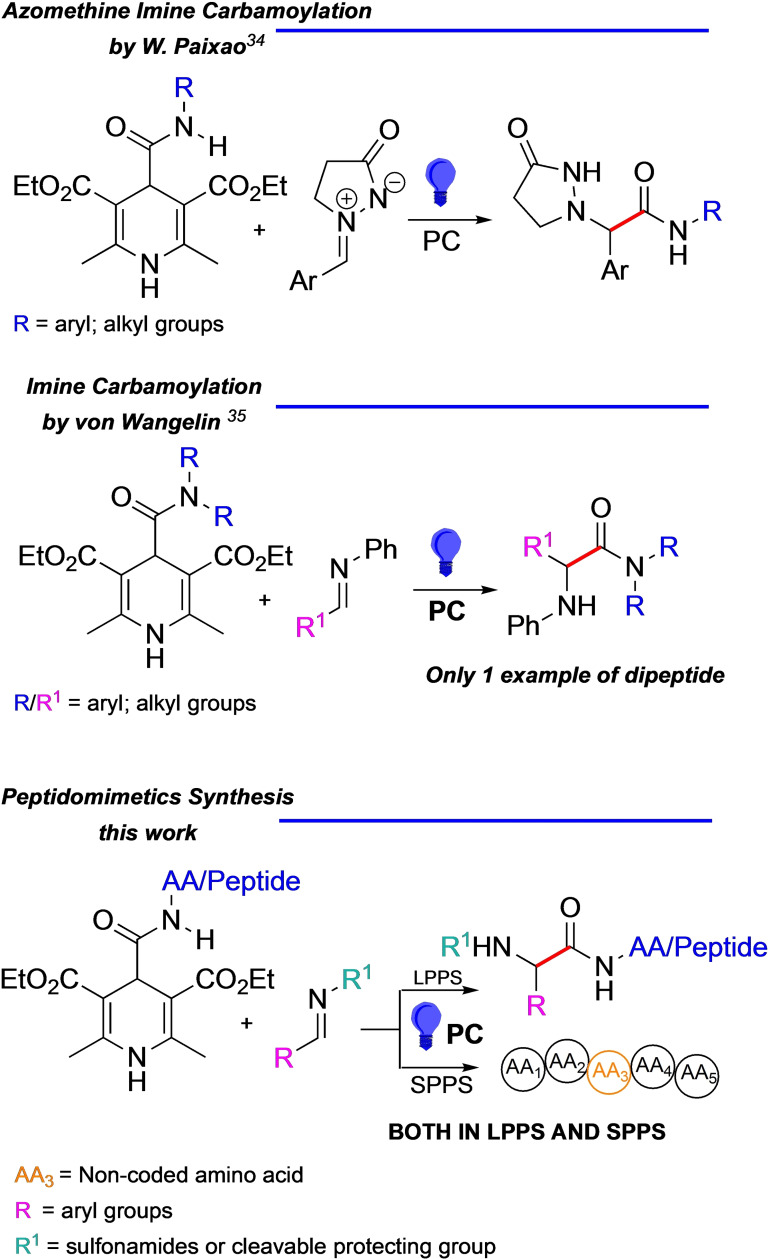
Profile of photocatalytic carbamoylation of imines and our work.

Here, we propose an innovative method to prepare peptidomimetics bearing a sulfonamide moiety or different protecting groups at *N*‐terminus, simultaneously generating the unnatural AA in the peptide sequence *via* photo‐redox catalysis. Our protocol relies on the formation of a new C=O−Cα bond thanks to the reaction between 4‐amido‐dihydropyridines (DHPs), known also as Hantzsch esters and differently functionalized imines, *i. e*. N‐sulfonyl, alkyl and aryl imines obtained from different aldehydes (Scheme 1).

DHPs are molecules that can act as hydride transfer agents to regioselectively reduce a variety of multiple bonds, *i. e*. C=C and C=N even of (hetero)aromatic substances,^[25]^ under mild reaction conditions.[^26^, ^27^, ^28^]

In the last few years, DHPs have also become useful reagents for photo‐redox reactions participating as sacrificial electron or hydrogen donors; furthermore, their 4‐alkyl or 4‐acyl‐derivatives easily work as alkyl or acyl radical precursor.[^29^, ^30^, ^31^, ^32^, ^33^, ^34^, ^35^]

Recently the von Wangelin^[35]^ and Paixao^[34]^ research groups reported on the carbamoylation of *N*‐aryl‐imines and azomethine imine, respectively, via photocatalysis (Scheme 1). Specifically, in the case of von Wangelin's work, variations of the *N*‐substituents of the imines were limited to aryl groups and linear or cyclic amines were used to functionalize the DHP, including only a single example of a dipeptide in the reported scope.^[35]^

In this work we envisage that the photocatalytic reaction between DHPs and imines could be an unconventional way to prepare peptidomimetics. In particular, we used different substituted *N*‐sulfonyl or *N*‐aryl/alkyl‐aldimines with DHP functionalized either with α‐AAs or peptides. Notably, we did not only propose a direct synthesis of ultra‐short peptidomimetics with the in‐situ formation of the non‐natural portion of the sequence, but we have also developed an easy‐to‐handle photo‐redox SPPS set‐up. (Scheme 1)

In literature there are some photocatalytic approaches where peptides, already anchored on resin, can be modified on the side chain residues.[^36^, ^37^, ^38^, ^39^, ^40^] However, to the best of our knowledge, this is the first reported method that allows the building of the peptide backbone, with the possibility of simultaneously inserting an unnatural AA at any position of the sequence.

To validate our approach, we decided to synthetize peptides characterized by different lengths and secondary structures (β‐strand, β‐hairpin and α‐helix), proving that this approach can work independently of the steric hindrance of the system.

## Results and Discussion

For the optimization of our reaction, we choose aldimine **1 a** (0.3 mmol, 1.0 eq.) and 1,4‐dihydropyridine derivatized with phenylalanine **2 a** (0.39 mmol, 1.3 eq.), obtained following the procedure reported in literature.[^34^, ^41^] Thus, a diastereomeric mixture of dipeptides **3 a/3’a** could be formed (Table 1). Inspired by the work of Yu's research group,^[31]^ we started the reaction optimization using metal‐containing photocatalysts (PCs) such as Ru(bpy)_3_Cl_2_ or Ir[p‐F(tBu)‐ppy]_3_ in CH_3_CN, changing also the wavelengths of the LED lamp (entries 1–3, Table 1). Only traces of the expected dipeptides were detected. Organic PCs were then studied. In the first attempt we selected Eosin Y, having the excited state oxidation potential comparable to Ru(bpy)_3_Cl_2_ (assuming a reductive quenching as reported by Yu et al.).^[31]^

**Table 1:** Optimization of the reaction between imine **1 a** and DHP **2 a**.

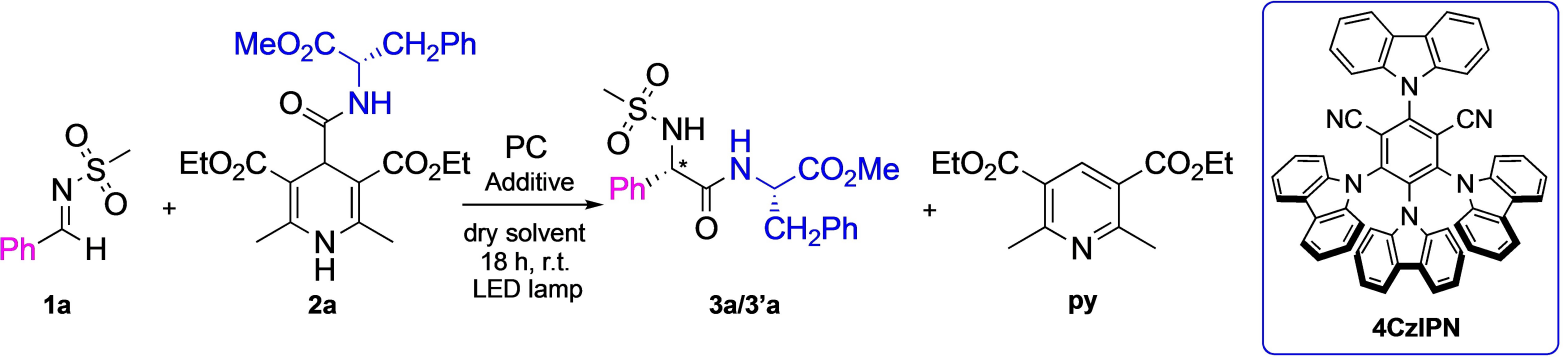					
Entry	Photocatalyst (0.025 equiv.)	λ (nm)	Solvent (0.1 M)	Additive (0.1 eq.)	Yield (**3 a:3’a**) %^[a]^
1	Ru(bpy)_3_Cl_2_ ⋅ 6H_2_O	440	CH_3_CN	/	<5
2	Ru(bpy)_3_Cl_2_ ⋅ 6H_2_O	427	CH_3_CN	/	<5
3	Ir[*p*‐F(tBu)‐ppy]_3_	440	CH_3_CN	/	<5
4	Eosin Y	467	CH_3_CN	/	10
5	4CzIPN	467	CH_3_CN	/	32
6	4CzIPN	440	CH_3_CN	/	28
7	4CzIPN	427	CH_3_CN	/	30
8	4CzIPN	White lamp	CH_3_CN	/	21
9	4CzIPN	467	*i‐*PrOH	/	12
10	4CzIPN	467	CH_2_Cl_2_	/	38
11	4CzIPN	467	CH_2_Cl_2_	TFA	18
12	4CzIPN	467	CH_2_Cl_2_	PhCO_2_H	51
13	4CzIPN	467	CH_2_Cl_2_	BF_3_⋅OEt_2_	78
14	5CzBN	467	CH_2_Cl_2_	BF_3_⋅OEt_2_	61
15	3DPAFIPN	467	CH_2_Cl_2_	BF_3_⋅OEt_2_	63
16	Eosin Y	467	CH_2_Cl_2_	BF_3_⋅OEt_2_	56

[a] Calculated after chromatography column as the sum of the two diastereoisomers.

By operating in CH_3_CN at 467 nm, diastereomers **3 a**/**3’a** were formed but in only 10 % yield (entry 4, Table 1). Taking inspiration from Paixao's work,^[34]^ we selected 4CzIPN as PC and, using the above conditions, we afforded **3 a/3’a** in 32 % (entry 5, Table 1). Thus, by using this dye, firstly we changed the LED wavelength which afforded worse results (entries 6–8, Table 1), and secondly the solvent, from CH_3_CN to *i*PrOH and CH_2_Cl_2_, finding an improvement of the yield with the latter one (38 %, entry 10, Table 1). At this point, we envisaged that the use of an additive to activate our imine could improve the yield. We observed a significant decrease of the yield by using TFA as additive (entry 11, Table 1). On the other hand, we succeeded in obtaining compound **3 a/3’a** from satisfactory to good yields by using PhCO_2_H (51 %, entry 12, Table 1) and BF_3_⋅OEt_2_ Lewis acid (78 %, entry 13, Table 1), respectively. Once optimized, we tested other organic donor‐acceptor cyanoarenes PCs, such as 5CzBN and 3DPAFIPN or Eosin Y; unfortunately, no further improvements were observed (entries 14–16, Table 1). It must be pointed out that the mentioned cyanoarenes were synthesized following a described procedure in literature,^[42]^ starting from inexpensive and commercially available compounds. Indeed, the use of this kind of PCs, replacing metal‐based dyes, makes our synthetic protocol greener and with potential compatibility with SPPS.

**Figure 1:**
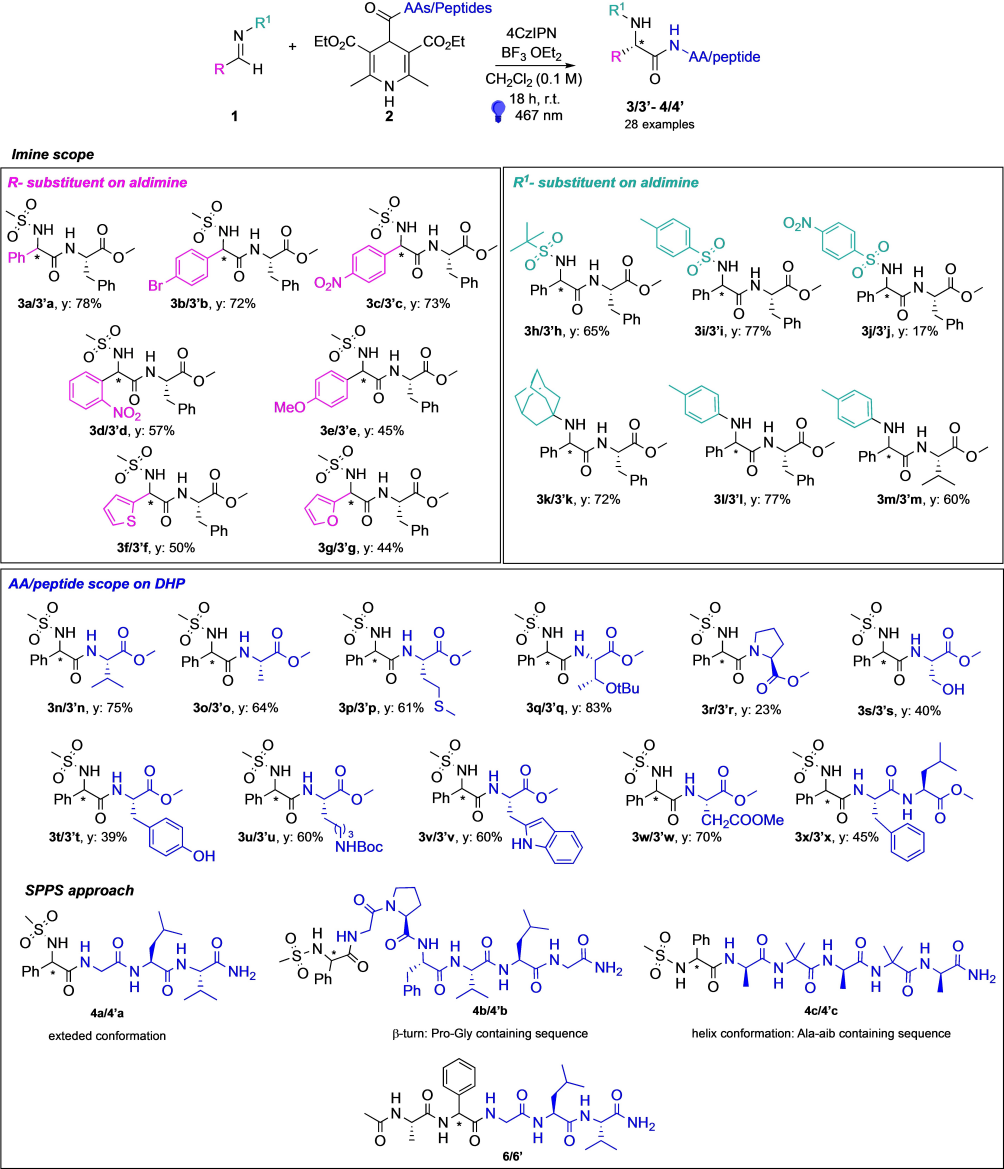
Scope of our carbamoylation reaction. y=yield. For all the synthesized compounds the diasteromeric ratio was found to be 50 : 50. Reaction conditions: Imine **1** (1.0 equiv.), DHP‐AA/Peptide **2** (1.3 equiv.), 4CzIPN (0.025 equiv.), BF_3_⋅OEt_2_ (1.0 equiv.) in dry CH_2_Cl_2_ (3.0 mL, 0.1 M).

Having established the optimal reaction conditions, we began to evaluate the scope of this transformation for a range of substituents both on the imine (R and R^1^) and on the functionalized DHP (Figure 1).

Focusing on sulfonimines, by changing the aryl moiety ‘R’, a general trend in the yield was observed. Indeed, when an EWG, such as a Br‐ or a NO_2_‐ substituent is placed in *para* position of the ring, the reaction works better than if an EDG, such as a MeO‐ group, is in the same position (compare **3 b/3’b** and **3 c/3’c** with **3 e/3’e**, Figure 1). On the other hand, a decrease in the yield was observed when the same EWG is in the *ortho* position of the ring (compare **3 c/3’c** with **3 d/3’d**) probably due to steric effects. Two examples of electron‐rich heteroaryl moieties were also reported. Even in this case, a decrease in terms of yield was detected (**3 f/3’f** and **3 g/3’g**).

**Scheme 2:**
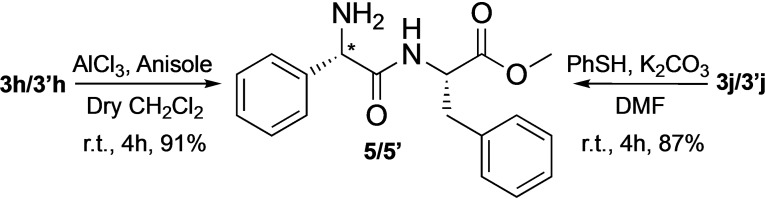
Deprotection of sulfonamide moieties.

Then, we investigated the effect of R^1^ substituent. As explained above, we mainly focused on the obtainment of peptidomimetics containing a sulfonamide‐moiety for their huge pharmaceutical interest. Between them, **3 h/3’h** and **3 j/3’j** were prepared to test the possibility to deprotect the *N*‐terminus of the obtained dipeptide respectively in acid and basic conditions (see below Scheme 2). The success of this purpose underlines the possibility of using this reaction for the preparation of peptidomimetics inserting the new C−C bond not only at the *N*‐terminus proximity, but also in the middle of a sequence when using SPPS. Beyond sulfonamides, we chose to insert R^1^ as an alkyl or an aryl substituent (Figure 1). No big differences were detected in terms of yields, even in the case of **3 k/3’k**, having a hindered adamantane moiety. Furthermore, we studied the scope of the DHP functionalization, selecting different AAs, also functionalized in the side chain. In case of the **3 n‐w/3’n‐w** dipeptides synthesis, changing the AA on DHP, we did not observe big differences in the yields. However, this is not the case of **3 r/3’r** where a decrease of the yield was probably ascribed to the steric hinderance given by the presence of the proline. The success in obtaining **3 s/3 s’** and **3 t/3 t’** (despite a 40 % yield) demonstrates that the reaction is effective even with free hydroxyl groups on the AA side chains. In case of the tripeptides **3 x/3’x**, the decrease in the yield depends on the scarce solubility of the two diastereomers (See SI).

To further demonstrate the versatility of our method, we explored the deprotection of the sulfonamide moieties. Envisaging the possibility of using our protocol for longer peptide sequences with orthogonal protecting groups on AA side chains, we investigated both acid and basic condition for the *N*‐terminus sulfonamide deprotection.

Thus, starting from **3 h/3’h** using acid conditions^[43]^ and from **3 j/3’j** with basic ones,^[44]^ we succeeded in obtaining compound **5/5’** with high yield (Scheme 2).

### Synthesis of 4 a‐c/4’a‐c and 6/6’ Through SPPS Approach

**Scheme 3:**
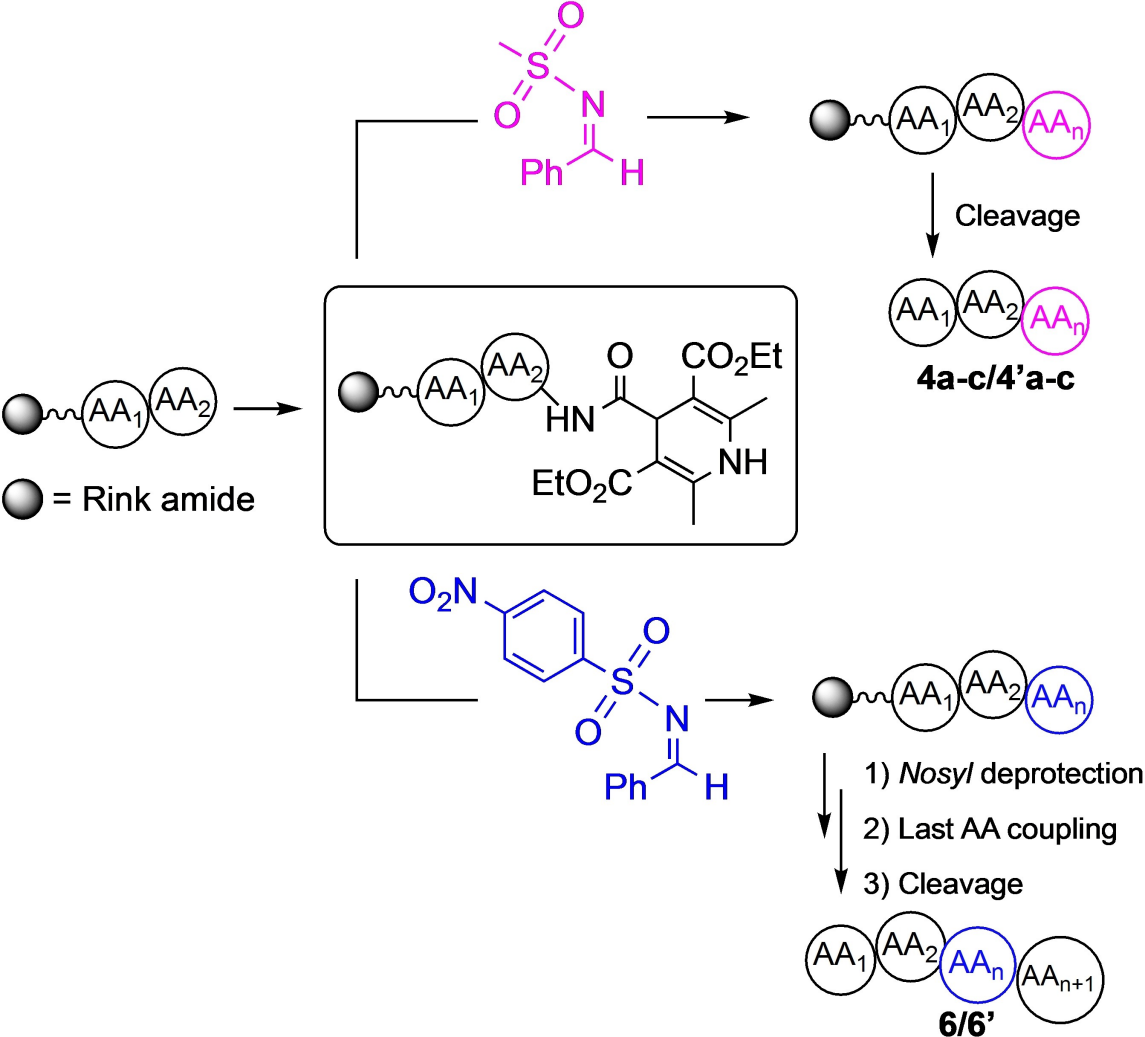
General scheme for the SPPS approach. **4 a‐c/4′ a‐c** and **6/6′** synthesis.

Subsequently, proved the feasibility of our photochemical approach, we envisaged the possibility to prepare longer peptidomimetic sequences by exploiting SPPS (Scheme 3). The reaction on peptides with different lengths and conformations was studied to demonstrate its feasibility regardless of the steric hindrance and the peptide secondary structure. Being known from the literature that Leu‐Val dipeptide induces an extended conformation,^[11]^ Pro‐Gly dipeptide is the β‐turn core^[45]^ of the prepared hairpin and Ala‐Aib containing sequence assumes a helix conformation,^[46]^ we synthesized peptides **4 a‐c/4’a‐c** (Scheme 3) choosing Rink amide as a resin. In all three cases, we succeeded in the obtainment of the above‐mentioned products starting from the DHP‐containing sequence (**2 m‐o** Figure S7 in SI).

It must be underlined that we decided to avoid using BF_3_⋅OEt_2_ to activate the imine for the SPPS approach. Indeed, taking in consideration that BF_3_⋅OEt_2_ is a Lewis acid, it could cleave the sequence anchored on the resin. Thus, we decided to work with an excess of the imine, compensating the lack of activation agent (Scheme 3 and SI for the synthetic details). Being successful the deprotection under basic condition (Scheme 2), we decided to build a further sequence on resin, aiming to insert the phenylglycine moiety in the middle of the peptide chain. Thus, in this case, instead of **1 a** (with Mesyl group on the nitrogen), we used the imine bearing Nosyl moiety on the nitrogen atom. After *N*‐terminus deprotection, the last AA was added, successfully obtaining peptides **6/6’** (Scheme 3. See also Scheme S9 of SI for the synthetic details). To the best of our knowledge, this is the first example of Nosyl deprotection on SPPS reported in literature.

### NMR and X‐Ray Characterization.

As explained before, since a new stereogenic centre is formed in the condensation process, two diastereomers are generated. In all cases, regardless of the hindrance or the electronic properties of the system, we did not observe any diasteroselection.

In some cases (**3 a**/**3’a**; **3 b**/**3’b**; **3 c**/**3’c**; **3 f**/**3’f**; **3 p**/**3’p**; **3 x**/**3’x** mixtures) the two diastereomers were separated by flash chromatography and fully characterized by NMR. Notably, similar behaviour in terms of chemical shift and protons patterns were observed for all compounds of **3** series and consequently for **3’** series. In case of the dipeptides, diagnostic signals are the NH of the natural amino acid, which resonates at lower field in **3** series with respect to **3’** ones. A different behaviour was observed for the α‐proton, found at lower field for **3’** diastereomers. Of note are also the chemical shift of the CO_2_ *Me* and *Me*SO_2_ groups, both resonating at lower field in **3’** series. An interesting diversity in the two series was found for the dipeptides containing the *L*‐Phe as the natural AA. In case of diastereoisomers **3**, the phenyl protons resonate in the typical field (*δ*=7.30–7.00 ppm). On the other hand, for **3’** isomers the same protons resonate at higher fields, showing very splitted signals [as example, here the chemical shifts for **3’b**: *δ*=7.21 (*1H*), 7.13 (*2H*), 6.68 (*2H*)], indicating that these protons are shielded. This behaviour could mean a strong π‐π interaction between the aromatic portions of the molecules.

**Figure 2:**
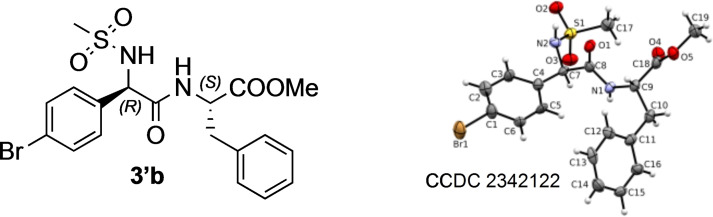
X‐Ray analysis of **3′ b**. Atoms are represented with the usual colour code (C: grey; N: blue; O: red; H: white; S: yellow; Br: gold)

X‐Ray analysis of **3’b** allows us to unequivocally assign its absolute stereochemistry (*R,S* for the **3’** series and consequently *S,S* for **3** series) (Figure 2).

While the details and **3’b** packing are explained in the SI, the π‐π interactions between the aromatic side chains of the two AA are quite interesting and is reflected on what we observed with NMR analysis. Indeed, a T‐shape configuration of the π‐π interactions are shown in the packing of **3’b** (Figure S11, SI), which results in a significant up‐field shift of the phenyl protons due to an enhancement of anisotropic shielding by the p‐electron cloud of the aryl substituent.[^47^, ^48^]

### Proposed Mechanism

**Figure 3:**
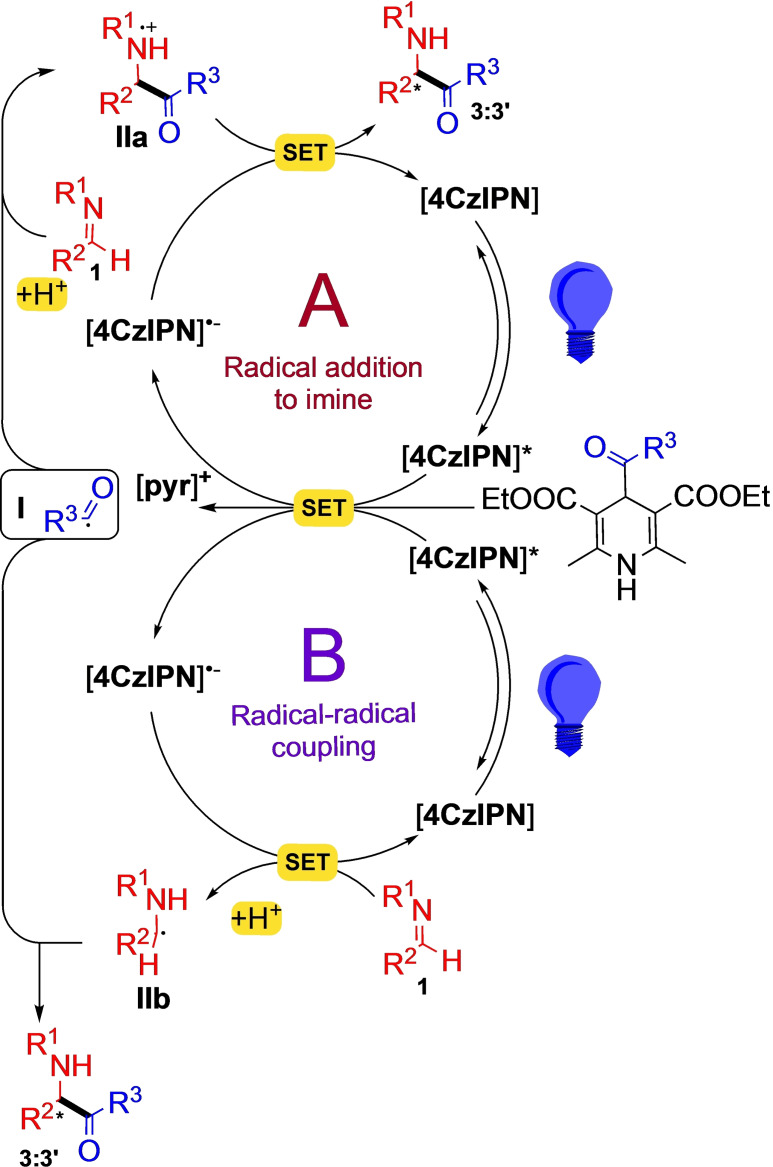
Proposed carbamoylation mechanism.

Control experiments and Stern‐Volmer analyses (see Chapter 5 of SI for the details) suggest two reductive quenching mechanisms, both starting with the formation of radical intermediate **I**, formed thanks to the reduction of the photocatalyst from its photo‐excited state (4CzIPN*) to its radical‐anion form (4CzIPN^−^). The carbamoyl radical **I** can go through two different cycles: radical addition to imine (path A, Figure 3) and radical‐radical coupling (path B, Figure 3).

In **path A**, the second step is the addition of **I** to imine, forming the radical intermediate **IIa**, which undergoes an additional single electron transfer (SET), forming the product and restoring the photocatalyst. **The path B** is based on the radical‐radical coupling between **I** and **IIb**, which is the intermediate formed by a SET event of imine **1**.

## Conclusions

In conclusion, we deeply studied a photocatalytic carbamoylation to be exploited for the preparation of peptidomimetics, with different functional groups at *N*‐terminus. We mainly focused on the sulfonamide moiety as *N*‐terminus capping, an important group for molecules of pharmaceutical interest. Beyond sulfonamides, we chose also to study the reaction with imine containing alkyl or an aryl groups on the nitrogen atom (see Figure 1). The described protocol consists in a carbamoylation reaction of imine using DHPs, functionalized with AAs/peptide sequences, and 4CzIPN as organic PC.

We synthesized more than 20 ultrashort peptidomimetics that were characterized by NMR spectroscopy, observing diagnostic signals that, together with the X‐ray analyses, allow us to assign the absolute configuration to each diastereomer.

We demonstrated that this valuable method not only works in liquid phase conditions but also in SPPS, promoting the obtainment of longer peptide sequences with diverse conformations. Moreover, the optimization of the on‐resin Nosyl deprotection, being to the best of our knowledge the first case reported in literature, allowed the insertion of the unnatural AA in any position of the peptidomimetic sequence, making our method a versatile protocol to synthesize valuable tools for disparate applications, from material to pharmaceutical chemistry.

## Supporting Information

The authors have cited additional references within the Supporting Information.[^49^, ^50^, ^51^, ^52^, ^53^, ^54^, ^55^, ^56^, ^57^, ^58^, ^59^, ^60^, ^61^, ^62^, ^63^, ^64^, ^65^, ^66^, ^67^]

## Conflict of Interests

The authors declare no conflict of interest.

1

## Supporting information

As a service to our authors and readers, this journal provides supporting information supplied by the authors. Such materials are peer reviewed and may be re‐organized for online delivery, but are not copy‐edited or typeset. Technical support issues arising from supporting information (other than missing files) should be addressed to the authors.

Supporting Information

## Data Availability

The data that support the findings of this study are available in the supplementary material of this article.
