# Supplementary material for: Innovative On‐Resin and in Solution Peptidomimetics Synthesis via Metal‐Free Photocatalytic Approach
Source: Chemistry. 2024 Nov 8;30(68):e202402790. doi: 10.1002/chem.202402790 (PMC11618039; doi:10.1002/chem.202402790)
Supplement: Supplementary file 1 — Supporting Information [file CHEM-30-e202402790-s001.pdf]

Chemistry–A European Journal

Supporting Information

Innovative On-Resin and in Solution Peptidomimetics Synthesis via Metal-Free Photocatalytic Approach

Tommaso Gandini, Francesco Vaghi, Zoe Laface, Giovanni Macetti, Alberto Bossi, Marta Penconi, Maria Luisa Gelmi, and Raffaella Bucci*

CHEMISTRY
A European Journal

Supporting Information

Innovative on-resin and in solution peptidomimetics synthesis via metal-free photocatalytic approach

Tommaso Gandini[a], Francesco Vaghi[b], Zoe Laface[a], Giovanni Macetti[c], Alberto Bossi[d], Marta Penconi[d], Maria Luisa Gelmi[a] and Raffaella Bucci*

[a] Dipartimento di Scienze Farmaceutiche, Università degli Studi di Milano, Via G. Venezian 21, 20133, Milano (Italy)

[b] Dipartimento di Scienze Chimiche, Università degli Studi di Padova, Via F. Marzolo 1, 35131, Padova (Italy)

[c] Dipartimento di Chimica, Università degli Studi di Milano, Via C. Golgi 19, 20133, Milano (Italy)

[d] Istituto di Scienze e Tecnologie Chimiche "Giulio Natta" (SCITEC) del Consiglio Nazionale delle Ricerche (CNR), Via G. Fantoli 16/15, 20138, Milano (Italy)

Summary

1. General Photochemical Remarks 3
2. General Procedures (**GP**) for the synthesis of imines 5
   2.1 **GP1**. Synthesis of sulfonyl imines **1a-j** 5
   2.2 **GP2**. Synthesis of aryl imines **1k-n** 5
3. Liquid Phase Peptide Synthesis (LPPS) 7
   3.1 **GP3**. Synthesis of functionalized DHPs **2a-l** 7
   3.2 Optimization of photocatalytic carbamoylation of imine **1a** with DHP-Phe **2a** to form products **3a/3'a** 9
   3.3 **GP4**. Photocatalytic carbamoylation of imines (LPPS) to form products **3a-x/3'a-x** 10
   3.4 Reaction scope (LPPS) 11
      3.4.1 Scope of imines (**1a-1l**) using DHP-Phe **2a** 12
      3.4.2 Scope of DHPs (**2b-2l**) using imine **1a** 18
4. Solid Phase Peptide Synthesis (SPPS) 22
   4.1 **GP5**. Synthesis of DHP functionalized peptides **2h-j** anchored on Rink Amide resin 23
   4.2 **GP6**. Photocatalytic carbamoylation of imines (SPPS) to form products **4a-c/4'a-c** and **6/6'** 24
   4.3 Removal of the -SO_2R moiety and sequence elongation to access product **6/6'** 25
   4.4 Reaction scope (SPPS) 27
5. Mechanicistic investigations 34
   5.1 Stern-Volmer analysis 34
   5.2 Control experiments 35
   5.3 Proposed catalytic cycle 39
6. NMR Spectra of the Isolated Products 40
7. Single-crystal X-ray Diffraction Analyses of **3'b** 82
8. References 86

1. General Photochemical Remarks

Steady state emission spectra and photoluminescence lifetimes were obtained with a FLS 980 spectrofluorimeter (Edinburgh Instrument Ltd.). Continuous excitation for the steady state measurements was provided by a 450 W Xenon arc lamp. Emission spectra were corrected for the wavelength-dependent sensitivity of the detector. Photoluminescence time-resolved measurements were carried out by TCSPC (time-correlated single-photon counting) method with an Edinburgh Picosecond Pulsed Diode Laser EPL-375 (Edinburgh Instrument Ltd.) and fitted with a sum of exponential decay to obtain the lifetimes of prompt and delayed fluorescence. Photoluminescence experiments were carried out in nitrogen-degassed solutions.

Photocatalyst **4CzIPN**,[1] **5CzBN**,[2] **3DPAFIPN**[2] and 3,5-diethoxycarbonyl-2,6-dimethyl-1,4-dihydropyridine-4-carboxylic acid (**A**)[3] were synthesized following the procedures reported in the literature (Figure S1).

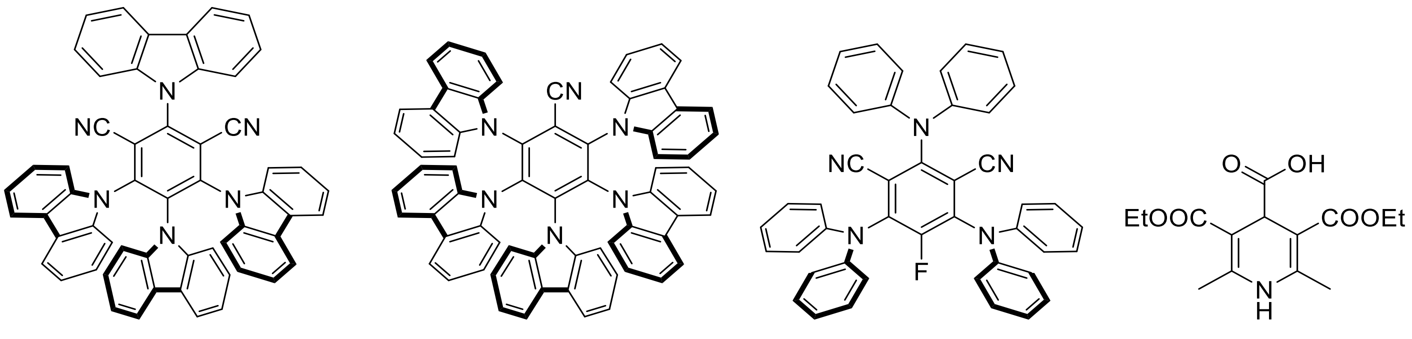

4CzIPN **5CzBN** **3DPAFIPN** DHP-COOH (**A**)

Figure S1. Photocatalysts synthesized and tested in this work, along with DHP-COOH **A**.

The photochemical reactions were run under inert atmosphere in 10 mL vials sealed with a septum, magnetically stirred at room temperature under irradiation from a Kessil PR160L lamp (λ = 467 nm, see Figure S2; distance between lamp and vial(s): ~5 cm). A fan was used to dissipate the heat generated by the LED lamp.

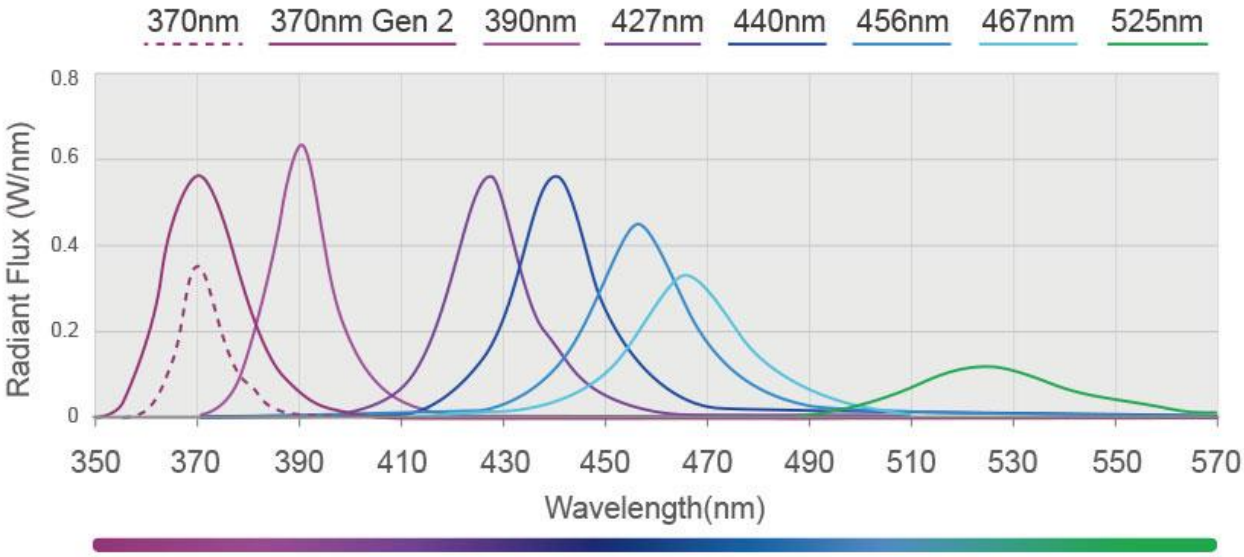

Figure S2. Emission profile of the Kessil® lamps used to irradiate the microwave vials.[4]

The experimental setup adopted in this work is presented in Figure S3.

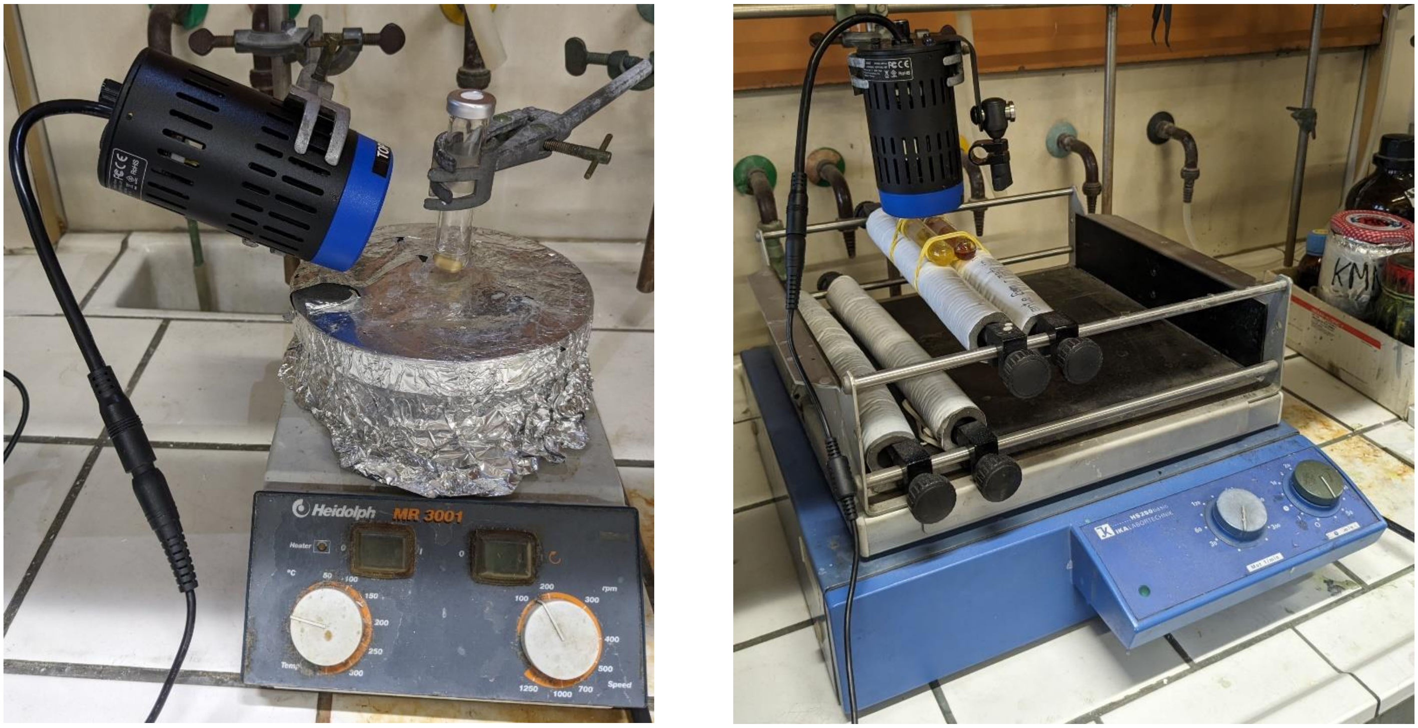

Figure S3. Experimental setup for the carbamoylation of imines with the liquid phase (*left*) or the solid phase (*right*) approaches.

2. General Procedures (GP) for the synthesis of imines

Chemicals were purchased from Sigma Aldrich, Fluorochem or TCI and were used without further purification. Analytical thin layer chromatography (TLC) was carried out using commercial silica gel plates, spots were detected with UV light and revealed either with cerium-ammonium molybdate or potassium permanganate alkaline solution. Flash column chromatography was performed using silica gel (60 Å, particle size 40-64 μm) as stationary phase, following the procedure by Still and co-workers.[5] Mass spectra (MS) were recorded on an LCQESI MS and on a LCQ Advantage spectrometer from Thermo Finningan and a LCQ Fleet spectrometer from Thermo Scientific. High resolution mass spectrometry (HRMS) were performed on Q-ToF SYNAPT G2-Si HDMS 8K mass spectrometer (Waters) using the ESI source at the Mass Spectrometry facility of the Unitech COSPECT at the University of Milan (Italy).
The NMR spectroscopic experiments were carried out either on Varian MERCURY 300 MHz (300 and 75 MHz for ^{1}H and ^{13}C, respectively), or Bruker Avance I 400 MHz spectrometers (400 and 101 MHz for ^{1}H and ^{13}C, respectively). Chemical shifts (δ) are given in ppm relative to the $CHCl_3$ internal standard, and the coupling constants J are reported in Hertz (Hz). Optical rotations ($[\alpha]_D$) were measured on a Perkin-Elmer 343 polarimeter at 20 °C (concentration in g/100 mL). Peptide sequences were purified using semi-preparative RP-HPLC Jasco using a Gemini column (5 μm, C18, 110 Å, 250 x 21.2 mm - Phenomenex), then injected in analytical HPLC JASCO NetII/ADC series, with PU-4180 pump and PDA detector MD-4010, Gemini-NX column (5 μm, C18, 150 x 4,6mm- Phenomenex). HPLC solvents were purchased from commercial sources and 0.1% of TFA was added prior to use for the purifications. UPLC-ESI-HR-MS analyses were performed at the Mass Spectrometry facility of the Unitech COSPECT technological platform at the University of Milan (Italy) on a Synapt G2-Si QToF instrument (equipped with a Z-Spray ESI-probe) (Waters) coupled with an Acquity UPLC I-Class chromatography system (Waters). The column Acquity UPLC Peptide CSH (C18, 100x2.1 mm, 1.7 μm, 130Å) was used for UPLC analyses, with an injection volume of 2 μl. UPLC solvents were purchased from commercial sources and 0.1% of formic acid was added prior to use for the purifications.

2.1 GP1. Synthesis of sulfonyl imines 1a-j

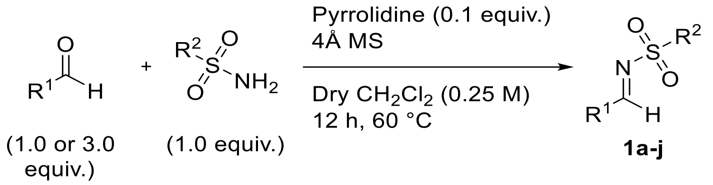

Scheme S1. Synthesis of sulfonyl imines **1a-j**.

General Procedure 1 (Scheme S1). According to the protocol reported in the literature,[6] operating in a dried Schlenk tube equipped with a stirring bar, 4Å molecular sieves (1 g for 1.0 mmol of aldehyde), sulfonamide (1.0 equiv.), pyrrolidine (0.1 equiv.), and the aldehyde (1.0 or 3.0 equiv.) were added to dry CH_2Cl_2 (0.25 M). The reaction mixture was stirred for 12 h at 60 °C. The mixture was cooled at r.t. and filtered through a Celite pad, then rinsed with CH_2Cl_2 (~50 mL). The solution was concentrated under reduced pressure and the crude product was purified by recrystallization using AcOEt and petroleum ether to yield the desired imines **1a-j**.

2.2 GP2. Synthesis of aryl imines 1k-n

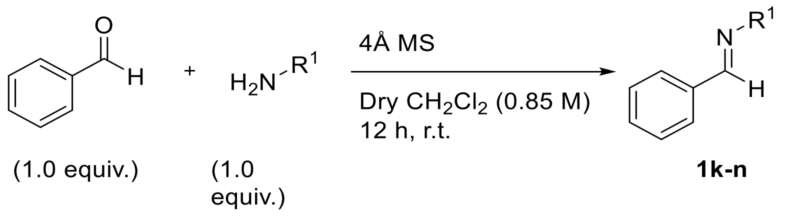

Scheme S2. Synthesis of aryl imines **1k-n**.

General Procedure 2 (Scheme S2). According to the protocol reported in the literature,[7] operating in a dried Schlenk tube equipped with a stirring bar, 4Å molecular sieves (1 g for 1.0 mmol of aldehyde), benzaldehyde (1.0 equiv.) and the amine (1.0 equiv.) were added to dry CH_2Cl_2 (0.85 M). The reaction mixture was stirred for 12 h at r.t., after which the mixture was filtered through a Celite pad, then rinsed with CH_2Cl_2 (~50 mL). The solution was concentrated under reduced pressure and the crude product was purified by recrystallization using AcOEt and petroleum ether to yield the desired imine **1k-n**.

Imines used in this work are presented in Figure S4. NMR data obtained for imines **1a**,[8] **1b**,[9] **1c**,[10] **1e**,[9] **1f**,[11] **1g**,[12] **1h**,[13] **1i**,[8] **1j**,[14] **1k**,[15] **1l**,[8] **1m**,[16] **1n**[17] are in agreement with the ones in the literature. Imine **1d**[18] is already present in the literature but no NMR spectrum was found, so we decided to report it.

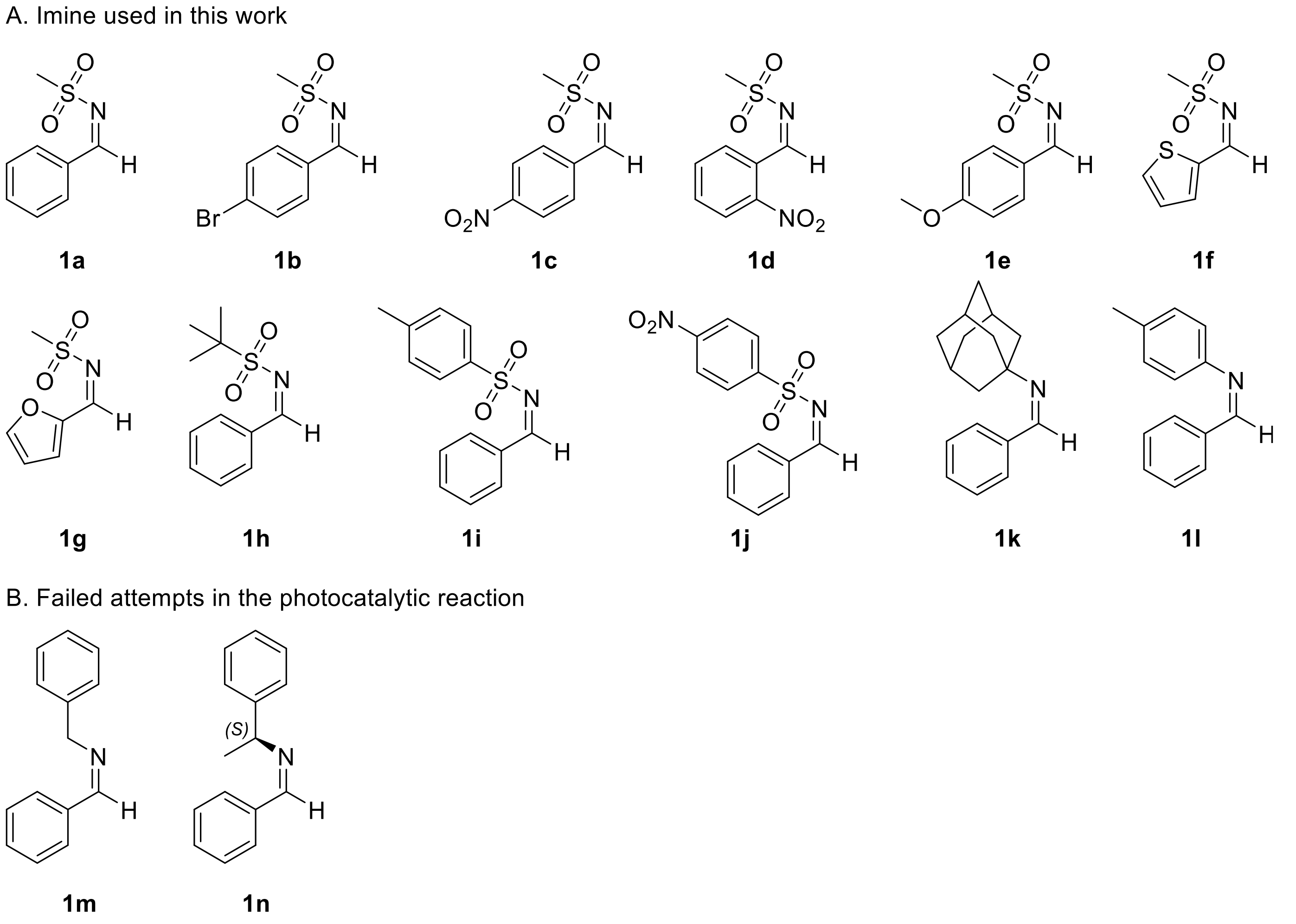

Figure S4. Imines **1a-n** used in this work.

N-(2-Nitrobenzylidene)methanesulfonamide (1d)

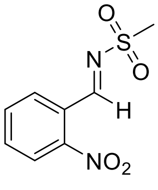

General Procedure 1. Methanesulfonamide (480 mg, 5.0 mmol, 1.0 equiv.), pyrrolidine (41 µL, 0.5 mmol, 0.1 equiv.), 2-nitrobenzaldehyde (771.0 mg, 5.0 mmol, 1.0 equiv.), in dry CH_2Cl_2 (20 mL, 0.25 M). Imine **1d** was obtained as a white solid (yield: 926.3 mg; 81%); ^1H NMR (300 MHz, $CDCl_3$) δ 9.54 (s, 1H), 8.22-8.10 (m, 2H), 7.86-7.75 (m, 2H), 3.19 (s, 3H); MS (ESI+): *m/z* $[M+H]^+$ calcd. for $C_8H_9N_2O_4S$: 229.03, found 229.62; $[M+OMe+Na]^+$ calcd. for $C_9H_{11}N_2NaO_5S$: 282.03, found 282.41.

3. Liquid Phase Peptide Synthesis (LPPS)

3.1 GP3. Synthesis of functionalized DHPs 2a-l

All dihydropyridine (DHP) 4-amido Hantzsch ester derivatives **2** were synthesized starting from 1,4-dihydropyridine-4-carboxylic acid (**A**) and an amino acid methyl ester, which was formed as reported in the literature (Scheme S3).[3]

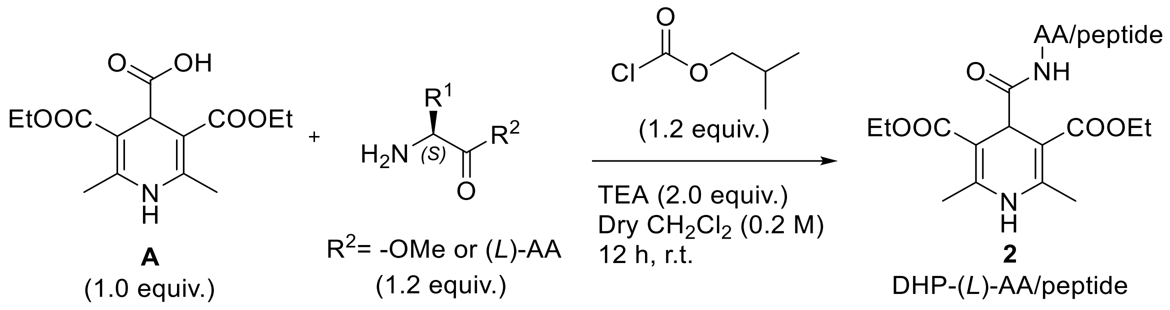

Scheme S3. Synthesis of functionalized DHPs **2a-l**.

General Procedure 3. Operating in a dried two-necked round bottomed flask equipped with a stirring bar, the 3,5-diethoxycarbonyl-2,6-dimethyl-1,4-dihydropyridine-4-carboxylic acid **A** (1.0 g, 3.36 mmol, 1.0 equiv.) was dissolved in dry CH_2Cl_2 (16.8 mL, 0.2 M), then TEA (0.93 mL, 6.72 mmol, 2.0 equiv.) was added. The reaction mixture was cooled down to 0 °C and *iso*butylchloroformate (0.52 mL, 4.03 mmol, 1.2 equiv.) was added dropwise. After 10 min., the reaction mixture was allowed to worm up to r.t. and the stirring was continued for additional 20 minutes. H_2N-(*L*)-AA-OMe·HCl (1.2 equiv.) or H_2N-(*L*)-Phe-(*L*)-Leu-OMe (1.2 equiv.), protected on the side chain when needed, was added and the mixture was stirred for 12 h at r.t. The mixture was diluted with CH_2Cl_2, washed with saturated $NaHCO_3$ (~30 mL) and water (~30 mL). Then the organic phase was dried using Na_2SO_4, filtered and concentrated under reduced pressure. The crude mixture was purified by flash chromatography to obtain the desired functionalized DHP-(*L*)-AA **2a-l**.

The functionalized DHPs used in this work are presented in Figure S5. NMR data obtained for DHP **2a**,[19] **2b**,[19] **2c**,[19] **2d**,[19] **2f**,[3] **2g**,[19] **2h**,[20] **2i**,[19] **2j**,[21] **2k**[19] and **2l**[19] are in agreement with the ones in the literature.

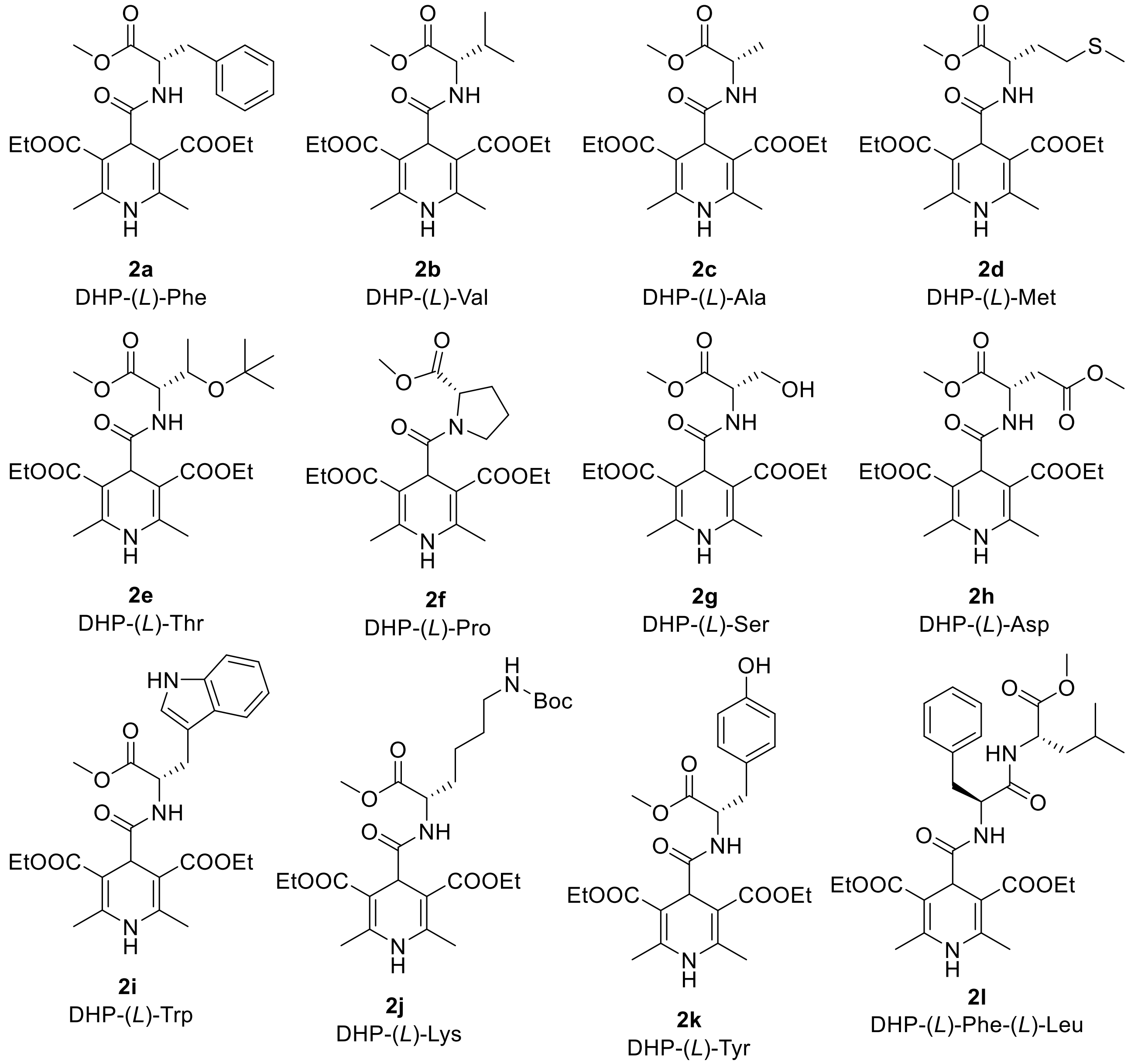

Figure S5. Functionalized DHPs **2a-l** used in this work.

Diethyl 4-((3-(*tert*-butoxy)-1-methoxy-1-oxobutan-2-yl)carbamoyl)-2,6-dimethyl-1,4-dihydropyridine-3,5-dicarboxylate (2e)

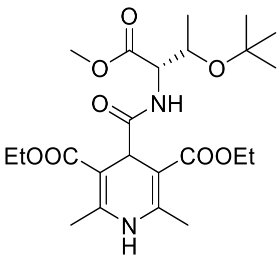

General Procedure 3. **A** (1.0 g, 3.36 mmol, 1.0 equiv.), TEA (0.93 mL, 6.72 mmol, 2.0 equiv.), isobutylchloroformate (0.52 mL, 4.03 mmol, 1.2 equiv.), (O*t*Bu)-(*L*)-Thr-OMe·HCl (911.6 mg, 4.04 mmol, 1.2 equiv.), in dry CH_2Cl_2 (16.8 mL, 0.2 M). Flash chromatography (Hex/Acetone, 9:1→1:1). DHP-(*L*)-Thr **2e** was obtained as a white solid (yield: 468.3 mg; 60%). M.p. = 148.5-149.9 °C; ^1H NMR (300 MHz, $CDCl_3$): δ 7.77 (s, 1H), 7.11 (d, *J* = 9.4 Hz, 1H), 4.74 (s, 1H), 4.44 (dd, *J* = 9.4, 2.1 Hz, 1H), 4.28-4.10 (m, 5H), 3.64 (s, 3H), 2.22 (s, 3H), 2.14 (s, 3H), 1.33-1.27 (m, 6H), 1.15-1.10 (m, 12H); ^{13}C NMR (75 MHz, $CDCl_3$): δ 175.2, 171.4, 167.5, 167.5, 147.7, 147.6, 98.0, 97.7, 74.1, 67.6, 60.1, 60.1, 58.2, 52.1, 41.6, 28.5 (x 3), 20.7, 18.9, 18.8, 14.6, 14.6; HRMS (ESI+): *m/z* $[M+Na]^+$ calcd. for $C_{23}H_{36}N_2NaO_8$: 491.2369, found 491.2371.

3.2 Optimization of photocatalytic carbamoylation of imine 1a with DHP-Phe 2a to form products 3a/3'a

Several parameters were changed in order to optimize the photocatalytic reaction conditions of imine **1a** with DHP-derivative **2a** affording a mixture of diastereoisomers **3a/3'a** (1:1 ratio) (Scheme S4). The attempts carried out to optimize the photocatalytic carbamoylation of imines are reported in Table S1.

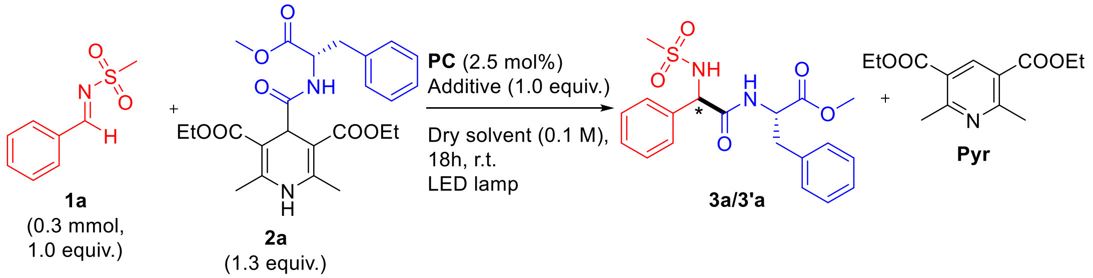

Scheme S4. Optimization of reaction conditions.

Table S1. Studying on the reaction optimization between imine **1a** and DHP-Phe **2a**.

Entry #	Photocatalyst (2.5 mol%)	LED lamp (nm)	Solvent (0.1 M)	Additive (1.0 equiv.)	**3a/3'a** Yield (%)[a]
1	$Ru(bpy)_3Cl_2 \cdot 6H_2O$	440	CH_3CN	/	<5
2	$Ru(bpy)_3Cl_2 \cdot 6H_2O$	427	CH_3CN	/	<5
3	Ir[*p*-F(*t*Bu)-ppy]$_3$	440	CH_3CN	/	<5
4	Eosin Y	467	CH_3CN	/	10
5	**4CzIPN**	467	CH_3CN	/	32
6	**4CzIPN**	440	CH_3CN	/	28
7	**4CzIPN**	427	CH_3CN	/	30
8	**4CzIPN**	White light	CH_3CN	/	21
9	**4CzIPN**	467	CH_2Cl_2	/	38
10	**4CzIPN**	467	*i*-PrOH	/	12
11	**4CzIPN**	467	CH_2Cl_2	TFA	18
12	**4CzIPN**	467	CH_2Cl_2	$PhCO_2H$	51
13	**4CzIPN**	467	CH_2Cl_2	$BF_3 \cdot OEt_2$	78
14	**5CzBN**	467	CH_2Cl_2	$BF_3 \cdot OEt_2$	61
15	**3DPAFIPN**	467	CH_2Cl_2	$BF_3 \cdot OEt_2$	63
16	Eosin Y	467	CH_2Cl_2	$BF_3 \cdot OEt_2$	56

[a] Calculated after column chromatography as the sum of the two diastereoisomers.

3.3 GP4. Photocatalytic carbamoylation of imines (LPPS) to form products 3a-x/3'a-x

Using the optimized reaction conditions (Table S1, entry 13), the scope was carried out testing imines **1a-l** and DHP-derivatives **2a-l** affording compounds **3a-x/3'a-x** (Scheme S5).

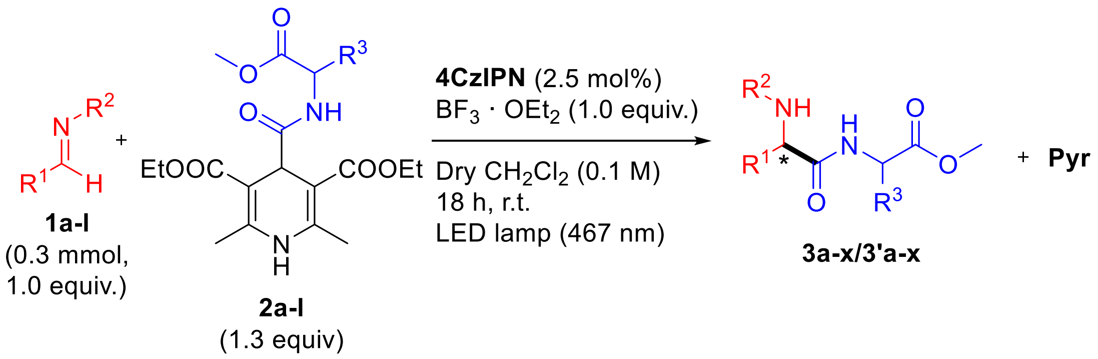

Scheme S5. Optimized reaction conditions for the carbamoylation of imines.

General Procedure 4. Operating in a 10 mL glass vial containing a stirring bar, imine **1** (0.3 mmol, 1.0 equiv.), DHP-(*L*)-AA **2** (0.39 mmol, 1.3 equiv.) and **4CzIPN** (5.9 mg, 0.0075 mmol, 0.025 equiv.) were added. The vial was sealed with a cap with septum and it was evacuated and flushed with nitrogen three times, then dry CH_2Cl_2 (3.0 mL, 0.1 M solution of the imine) and $BF_3{\cdot}OEt_2$ (37 µL, 0.30 mmol, 1.0 equiv.) were added. The reaction mixture was stirred for 18 h at r.t. under irradiation with a blue LED lamp (λ = 467 nm; distance between lamp and vial(s): ~5 cm; a fan was used to dissipate the heat generated by the lamp). The mixture was washed with H_2O (10 mL), the aqueous phase was extracted with CH_2Cl_2 (3 x 10 mL) and the combined organic layers were dried using Na_2SO_4. After filtering of the solid, the mixture was concentrated under reduced pressure. In all cases the diastereoisomeric ratio of **3/3'** from crude ^{1}H NMR analysis was 1:1. The crude mixture was purified by flash column chromatography on silica gel to afford the desired products **3/3'**; when stated, the products were recrystallized using AcOEt/petroleum ether.

3.4 Reaction scope (LPPS)

Synthesized products **3/3’** are shown in Figure S6. In all cases, a 1:1 diastereoisomeric ratio was detected by ^{1}H-NMR analysis of the crude product.

A. Scope of aldimines **1a-1l**

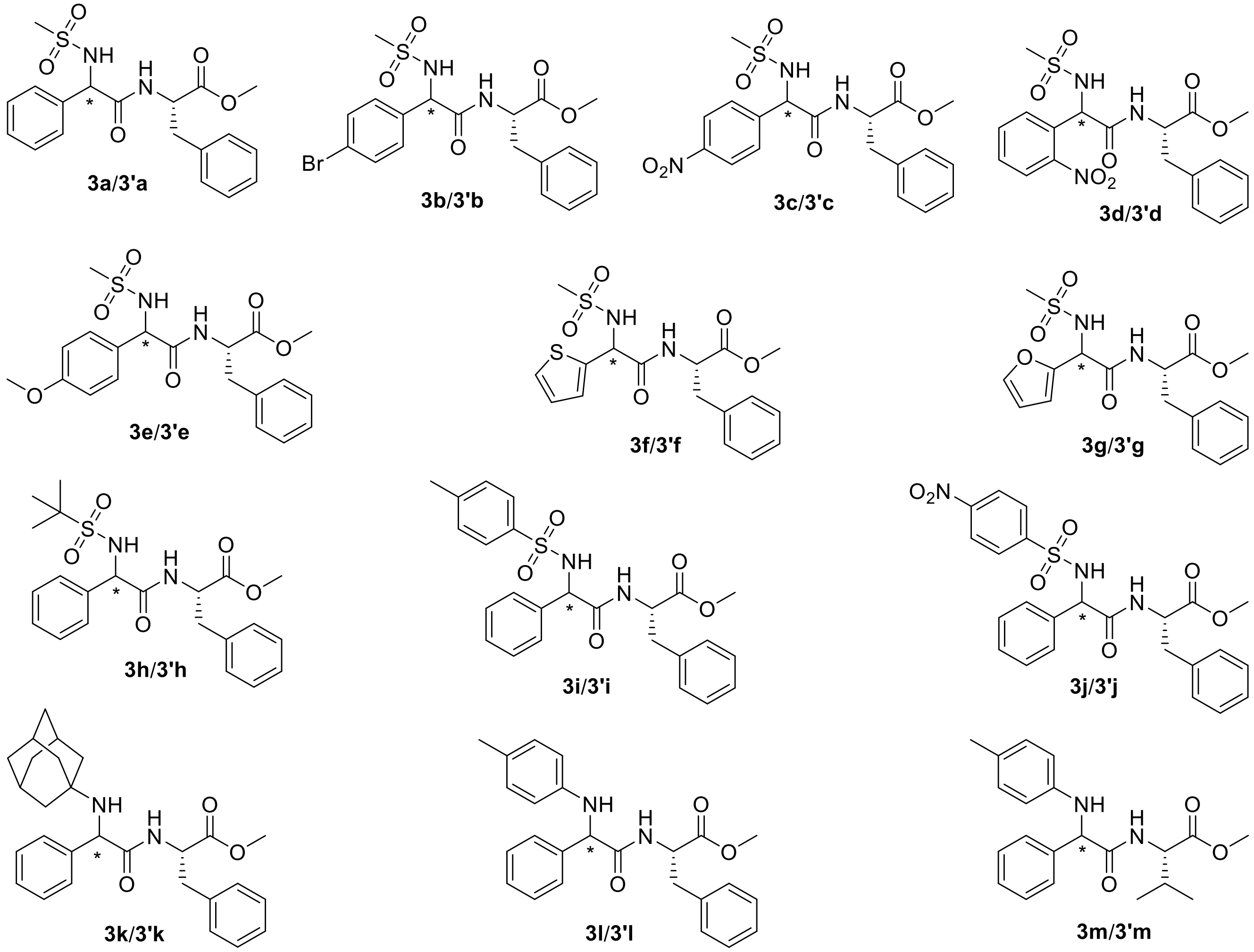

B. Scope of derivatized DHPs **2b-2l**

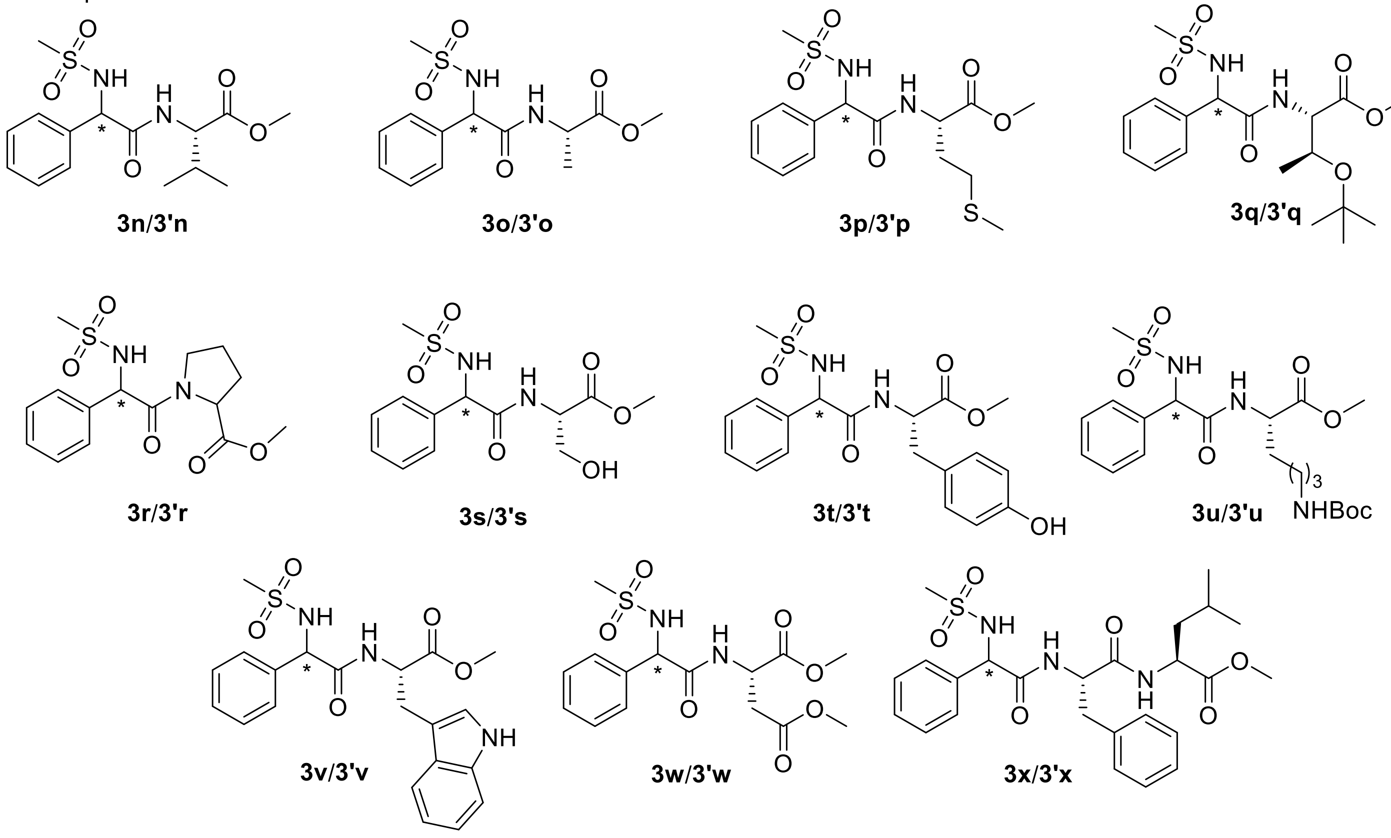

Figure S6. Reaction scope of aldimines **1a-l** (**A**) and derivatized DHPs **2b-l** (**B**).

3.4.1 Scope of imines (1a-1l) using DHP-Phe 2a

Methyl (2-(methylsulfonamido)-2-phenylacetyl)-*L*-phenylalaninate (**3a/3'a**)

General Procedure 4. Imine **1a** (55.0 mg, 0.30 mmol, 1.0 equiv.), DHP-(*L*)-Phe **2a** (178.8 mg, 0.39 mmol, 1.3 equiv.), **4CzIPN** (5.9 mg, 0.0075 mmol, 0.025 equiv.), $BF_3 \cdot OEt_2$ (37 µL, 0.30 mmol, 1.0 equiv.) in dry CH_2Cl_2 (3.0 mL, 0.1 M). Flash chromatography (Hex/EtOAc, 9:1→6:4).

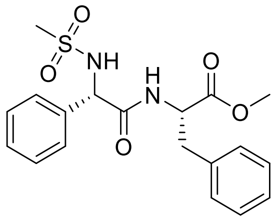

Dipeptide **3a** (R_f = 0.55 in Hex/AcOEt 1:1) was obtained as a white solid after recrystallization using Hex/EtOAc mixture (yield: 44.2 mg; 38%); M.p. = 157.8-160.6 °C; ^{1}H NMR (300 MHz, $CDCl_3$): δ 7.42-7.34 (m, 3H), 7.34-7.19 (m, 5H), 7.08-7.00 (m, 2H), 6.08 (d, J = 7.8 Hz, 1H), 5.74, 5.00 (AX system, J = 5.4 Hz, 2H), 4.86-4.74 (m, 1H), 3.67 (s, 3H), 3.18 (dd, J = 14.0, 5.5 Hz, 1H), 3.02 (dd, J = 14.0, 6.6 Hz, 1H), 2.62 (s, 3H); ^{13}C NMR (75 MHz, $CDCl_3$): δ 171.3, 168.8, 136.2, 135.5, 129.6 (x 2), 129.4, 129.3 (x 2), 128.9 (x 2), 127.9 (x 2), 127.5, 60.6, 53.7, 52.6, 42.0, 37.7; HRMS (ESI+): *m/z* $[M+Na]^+$ calcd. for $C_{19}H_{22}N_2NaO_5S$: 413.1147, found 413.1143; $[\alpha]_D^{28.6}$ = +0.5338 ± 0.0068 (*c* 1.00, CH_2Cl_2).

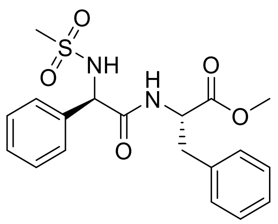

Dipeptide **3'a** (R_f = 0.48 in Hex/AcOEt 1:1) was obtained as a white solid after recrystallization using Hex/EtOAc mixture (yield: 46.9 mg; 40%); M.p. = 137.2-140.1 °C; ^{1}H NMR (400 MHz, $CDCl_3$): δ 7.44-7.36 (m, 3H), 7.35-7.27 (m, 2H), 7.18-7.10 (m, 1H), 7.10-7.01 (m, 2H), 6.67-6.59 (m, 2H), 5.99 (d, *J* = 8.0 Hz, 1H), 5.85, 5.04 (AX system, *J* = 5.2 Hz, 2H), 4.89 (dt, *J* = 8.0, 5.6 Hz, 1H), 3.73 (s, 3H), 2.97 (d, *J* = 5.6 Hz, 2H), 2.64 (s, 3H); ^{13}C NMR (101 MHz, $CDCl_3$): δ 171.3, 168.4, 136.8, 135.0, 129.7 (x 2), 129.3, 129.1 (x 2), 128.7 (x 2), 127.9 (x 2), 127.2, 60.5, 53.4, 52.7, 42.0, 37.6; MS (ESI+): *m/z* $[M+H]^+$ calcd. for $C_{19}H_{23}N_2O_5S$: 391.12, found 391.35; $[M+Na]^+$ calcd. for $C_{19}H_{22}N_2NaO_5S$: 413.11, found 413.17; $[\alpha]_D^{29.3}$ = -0.0340 ± 0.0222 (*c* 0.227, CH_2Cl_2).

Methyl (2-(4-bromophenyl)-2-(methylsulfonamido)acetyl)-*L*-phenylalaninate (3b/3'b)

General Procedure 4. Imine **1b** (78.3 mg, 0.30 mmol, 1.0 equiv.), DHP-(*L*)-Phe **2a** (178.8 mg, 0.39 mmol, 1.3 equiv.), **4CzIPN** (5.9 mg, 0.0075 mmol, 0.025 equiv.), $BF_3{\cdot}OEt_2$ (37 μL, 0.30 mmol, 1.0 equiv.) in dry CH_2Cl_2 (3.0 mL, 0.1 M). Flash chromatography (Hex/EtOAc, 7:3→1:1).

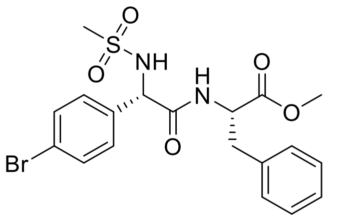

Dipeptide **3b** (R_f = 0.55 in Hex/AcOEt 1:1) was obtained as a white solid after recrystallization (yield: 52.5 mg; 37%); M.p. = 174.0-175.4 °C; ^{1}H NMR (300 MHz, $CDCl_3$): δ 7.49, 7.18 (AA'XX' system, *J* = 8.4 Hz, 4H), 7.32-7.22 (m, 3H), 7.08-6.99 (m, 2H), 6.11 (d, *J* = 7.8 Hz, 1H), 5.75, 4.97 (AX system, *J* = 5.7 Hz, 2H), 4.86-4.72 (m, 1H), 3.69 (s, 3H), 3.18 (dd, *J* = 14.0, 5.4 Hz, 1H), 3.03 (dd, *J* = 14.0, 6.7 Hz, 1H), 2.65 (s, 3H); ^{13}C NMR (75 MHz, $CDCl_3$): δ 171.3, 168.4, 135.4, 135.3, 132.7 (x 2), 129.5 (x 2), 129.3 (x 2), 129.0 (x 2), 127.5, 123.6, 59.9, 53.7, 52.7, 42.0, 37.6; HRMS (ESI+): *m/z* $[M+Na]^+$ calcd. for $C_{19}H_{21}BrN_2NaO_5S$: 491.0252 for ^{79}Br and 493.0232 for ^{81}Br, found 491.0249 and 493.0230, respectively; $[\alpha]_D^{28.6}$ = +0.5338 ± 0.0068 (*c* 1.00, CH_2Cl_2).

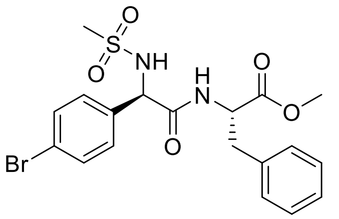

Dipeptide **3'b** (R_f = 0.47 in Hex/AcOEt 1:1) was obtained as a white solid after recrystallization (yield: 49.4 mg; 35%); M.p. = 162.5-165.0 °C; ^{1}H NMR (400 MHz, $CDCl_3$): δ 7.57-7.45 (m, 2H), 7.24-7.04 (m, 5H), 6.72-6.59 (m, 2H), 5.86 (d, *J* = 8.0 Hz, 1H), 5.81, 4.98 (AX system, *J* = 5.0 Hz, 2H), 4.92-4.79 (m, 1H), 3.76 (s, 3H), 3.09-2.86 (m, 2H), 2.68 (s, 3H); ^{13}C NMR (101 MHz, $CDCl_3$): δ 171.2, 167.9, 136.0, 134.9, 132.9 (x 2), 129.4 (x 2), 129.0 (x 2), 128.8 (x 2), 127.4, 123.6, 59.8, 53.4, 52.8, 42.1, 37.6; MS (ESI+): *m/z* $[M+H]^+$ calcd. for $C_{19}H_{22}BrN_2O_5S$: 469.04 for ^{79}Br and 471.04 for ^{81}Br, found 469.49 and 471.75, respectively; $[M+Na]^+$ calcd. for $C_{19}H_{21}BrN_2NaO_5S$: 491.03 for ^{79}Br and 493.03 for ^{81}Br, found 491.75 and 493.45, respectively; $[\alpha]_D^{28.6}$ = -0.0140± 0.0011 (*c* 0.100, CH_2Cl_2).

Methyl (2-(methylsulfonamido)-2-(4-nitrophenyl)acetyl)-*L*-phenylalaninate (3c/3'c)

General Procedure 4. Imine **1c** (68.5 mg, 0.30 mmol, 1.0 equiv.), DHP-(*L*)-Phe **2a** (178.8 mg, 0.39 mmol, 1.3 equiv.), **4CzIPN** (5.9 mg, 0.0075 mmol, 0.025 equiv.), $BF_3{\cdot}OEt_2$ (37 μL, 0.30 mmol, 1.0 equiv.) in dry CH_2Cl_2 (3.0 mL, 0.1 M). Flash chromatography (Hex/EtOAc, 8:2→1:1).

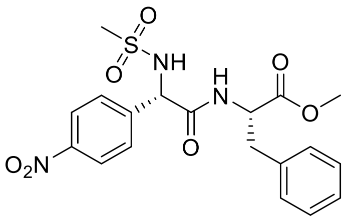

Dipeptide **3c** (R_f = 0.49 in Hex/AcOEt 1:1) was obtained as a white solid after recrystallization (yield: 46.8 mg; 36%). M.p. = 187.3-189.2 °C; ^{1}H NMR (400 MHz, $CDCl_3$): δ 8.35-8.07 (m, 2H), 7.58-7.40 (m, 2H), 7.38-7.27 (m, 3H), 7.13-6.99 (m, 2H), 6.01 (d, *J* = 7.9 Hz, 1H), 5.70, 5.09 (AX system, *J* = 5.9 Hz, 2H), 4.91-4.73 (m, 1H), 3.72 (s, 3H), 3.22 (dd, *J* = 14.0, 5.5 Hz, 1H), 3.06 (dd, *J* = 14.0, 6.7 Hz, 1H), 2.72 (s, 3H); ^{1}H NMR (300 MHz, CD_3CN): δ 8.24-8.15 (m, 2H), 7.60-7.52 (m, 2H), 7.34-7.15 (m, 6H), 6.28, 5.13 (AA'XX' system, *J* = 8.2 Hz, 2H), 4.76 – 4.64 (m, 1H), 3.61 (s, 3H), 3.20 (dd, *J* = 14.1, 5.2 Hz, 1H), 2.98 (dd, *J* = 14.0, 9.0 Hz, 1H), 2.67 (s, 3H); ^{13}C NMR (75 MHz, CD_3CN): *δ* 172.2, 169.2, 149.0, 145.4, 137.7, 130.3 (x 2), 129.6 (x 2), 129.5 (x 2), 127.9, 124.8 (x 2), 60.2, 54.9, 52.9, 41.5, 37.7; MS (ESI+): *m/z* $[M+H]^+$ calcd. for $C_{19}H_{22}N_3O_7S$: 436.12, found 436.92; $[M+Na]^+$ calcd. for $C_{19}H_{21}N_3NaO_7S$: 458.10, found 458.62; $[2M+Na]^+$ calcd. for $C_{38}H_{42}N_6NaO_{14}S_2$: 893.21, found 893.14; $[\alpha]_D^{29.2}$ = +0.4410 ± 0.0025 (*c* 0.50, CH_2Cl_2).

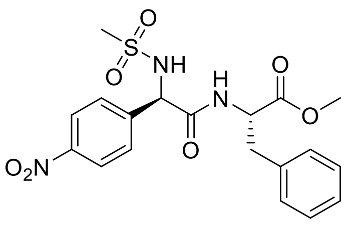

Dipeptide **3’c** (R_f = 0.41 in Hex/AcOEt 1:1) was obtained as a white solid after recrystallization (yield: 48.1 mg; 37%); M.p. = 174.8-176.0 °C; ^{1}H NMR (400 MHz, $CDCl_3$): δ 8.20, 7.42 (AA’XX’ system, J = 8.6 Hz, 4H), 7.19-7.14 (m, 1H), 7.12-7.04 (m, 2H), 6.74-6.68 (m, 2H), 5.92 (d, J = 7.9 Hz, 1H), 5.83, 5.11 (AX system, J = 5.6 Hz, 2H), 4.91-4.84 (m, 1H), 3.79 (s, 3H), 3.09 (dd, J = 14.0, 5.0 Hz, 1H); 2.93 (dd, J = 14.0, 6.7 Hz, 1H), 2.76 (s, 3H); ^{1}H NMR (300 MHz, CD_3CN): δ 8.18-8.09 (m, 2H), 7.49-7.38 (m, 2H), 7.21-7.06 (m, 4H), 7.02-6.91 (m, 2H), 6.30 (s, 1H), 5.15 (s, 1H), 4.78-4.63 (m, 1H), 3.70 (s, 3H), 3.13 (dd, J = 14.1, 4.9 Hz, 1H), 2.88 (dd, J = 14.1, 9.2 Hz, 1H), 2.80 (s, 3H). ^{13}C NMR (75 MHz, CD_3CN): δ 172.3, 169.3, 148.9, 145.5, 137.4, 130.0 (x 2), 129.3 (x 3), 127.7, 124.9 (x 2), 60.1, 54.6, 53.0, 41.6, 37.7; HRMS (ESI+): *m/z* $[M+Na]^+$ calcd. for $C_{19}H_{21}N_3NaO_7S$: 458.0998, found 458.0998; $[\alpha]_D^{28.4}$ = -0.0710 ± 0.0045 (*c* 1.00, CH_2Cl_2).

Methyl (2-(methylsulfonamido)-2-(2-nitrophenyl)acetyl)-*L*-phenylalaninate (**3d**/**3’d**)

General Procedure 4. Imine **1d** (68.5 mg, 0.30 mmol, 1.0 equiv.), DHP-(*L*)-Phe **2a** (178.8 mg, 0.39 mmol, 1.3 equiv.), **4CzIPN** (5.9 mg, 0.0075 mmol, 0.025 equiv.), $BF_3{\cdot}OEt_2$ (37 µL, 0.30 mmol, 1.0 equiv.) in dry CH_2Cl_2 (3.0 mL, 0.1 M). Flash chromatography (Hex/EtOAc, 7:3→1:1).

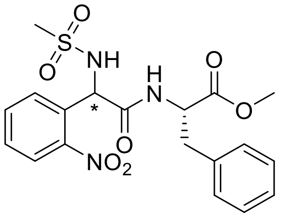

Dipeptide **3d**/**3’d** (R_f = 0.52 in Hex/AcOEt 1:1) was obtained as a white powder (yield: 74.6 mg, 57%) as a mixture of inseparable diastereoisomers; M.p. = 153.5-155.8 °C; ^{1}H NMR (300 MHz, $CDCl_3$): δ 8.06-7.92 (m, 2H), 7.73-7.61 (m, 2H), 7.61-7.46 (m, 4H), 7.37-7.19 (m, 4H), 7.19-6.97 (m, 6H), 6.80-6.69 (m, 2H), 6.28 (d, J = 7.0 Hz, 1H), 6.20 (d, J = 7.5 Hz, 1H), 5.67-5.53 (m, 2H), 4.89-4.72 (m, 2H), 3.75 (s, 3H), 3.62 (s, 3H), 3.22-2.91 (m, 4H), 2.78 (s, 3H), 2.74 (s, 3H); ^{13}C NMR (75 MHz, $CDCl_3$): δ 171.2, 171.2, 167.3, 167.2, 148.1 (x 2), 135.5, 135.2, 134.5 (x 2), 132.9, 132.4, 131.4, 130.9, 130.0, 129.8, 129.3 (x 2), 129.0 (x 2), 128.9 (x 2), 128.7 (x 2), 127.5, 127.2, 125.5, 125.4, 56.8, 56.1, 53.9, 53.7, 52.7, 52.5, 41.7, 41.6, 37.9, 37.5; HRMS (ESI+): *m/z* $[M+Na]^+$ calcd. for $C_{19}H_{21}N_3NaO_7S$: 458.0998, found 458.0998.

Methyl (2-(4-methoxyphenyl)-2-(methylsulfonamido)acetyl)-*L*-phenylalaninate (**3e**/**3’e**)

General Procedure 4. Imine **1e** (63.9 mg, 0.30 mmol, 1.0 equiv.), DHP-(*L*)-Phe **2a** (178.8 mg, 0.39 mmol, 1.3 equiv.), **4CzIPN** (5.9 mg, 0.0075 mmol, 0.025 equiv.), $BF_3{\cdot}OEt_2$ (37 µL, 0.30 mmol, 1.0 equiv.) in dry CH_2Cl_2 (3.0 mL, 0.1 M). Flash chromatography (CH_2Cl_2/EtOAc, 95:5→9:1).

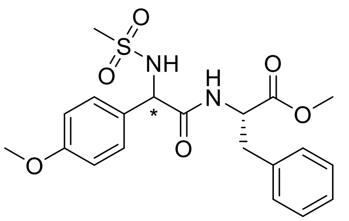

Dipeptide **3e**/**3’e** (R_f = 0.63 in CH_2Cl_2/EtOAc 9:1) was obtained as a light yellow solid (yield: 56.6 mg, 45%) as a mixture of inseparable diastereoisomers; M.p. = 94.2-99.6 °C; ^{1}H NMR (300 MHz, $CDCl_3$): δ 7.31-7.19 (m, 5H), 7.17-7.00 (m, 6H), 6.94-6.83 (m, 5H), 6.72-6.64 (m, 2H), 6.06 (d, J = 7.8 Hz, 1H), 5.98 (d, J = 8.1 Hz, 1H), 5.82 (d, J = 5.0 Hz, 1H), 5.70 (d, J = 5.1 Hz, 1H), 5.04-4.92 (m, 2H), 4.92-4.74 (m, 2H), 3.85 (s, 3H), 3.80 (s, 3H), 3.73 (s, 3H), 3.67 (s, 3H), 3.22-2.94 (m, 4H), 2.62 (s, 3H), 2.59 (s, 3H); ^{13}C NMR (75 MHz, $CDCl_3$): δ 171.4, 171.3, 169.2, 168.8, 135.5 (x 2), 135.1 (x 2), 129.3 (x 2), 129.3 (x 2), 129.2 (x 2), 129.2 (x 2), 128.9 (x 2), 128.7 (x 2), 128.1 (x 2), 127.4, 127.2, 115.0 (x 2), 114.9 (x 2), 60.1, 59.9, 55.5, 55.4, 53.7, 53.3, 52.7, 52.6, 42.0, 42.0, 37.7, 37.7; HRMS (ESI+): *m/z* $[M+Na]^+$ calcd. for $C_{20}H_{24}N_2NaO_6S$: 443.1253, found 443.1254.

Methyl (2-(methylsulfonamido)-2-(thiophen-2-yl)acetyl)-*L*-phenylalaninate (**3f**/**3'f**)

General Procedure 4. Imine **1f** (56.8 mg, 0.30 mmol, 1.0 equiv.), DHP-(*L*)-Phe **2a** (178.8 mg, 0.39 mmol, 1.3 equiv.), **4CzIPN** (5.9 mg, 0.0075 mmol, 0.025 equiv.), $BF_3{\cdot}OEt_2$ (37 µL, 0.30 mmol, 1.0 equiv.) in dry CH_2Cl_2 (3.0 mL, 0.1 M). Flash chromatography (Hex/EtOAc, 85:15→1:1) product **3f** was obtained pure, while **3'f** was enriched in the first diastereoisomer.

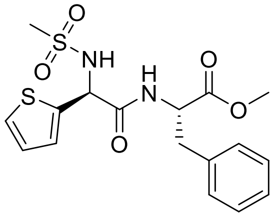

Dipeptide **3f** (R_f = 0.48 in Hex/AcOEt 1:1) was obtained as a white solid (yield: 15.4 mg; 13%); M.p. = 153.6-157.0 °C; ^{1}H NMR (400 MHz, $CDCl_3$): δ 7.37-7.22 (m, 4H), 7.11-7.02 (m, 3H), 7.00-6.93 (m, 1H), 6.24 (d, *J* = 7.8 Hz, 1H), 5.72, 5.31 (AX system, *J* = 5.4 Hz, 2H), 4.93-4.73 (m, 1H), 3.70 (s, 3H), 3.19 (dd, *J* = 14.0, 5.5 Hz, 1H), 3.06 (dd, *J* = 14.0, 6.4 Hz, 1H), 2.69 (s, 3H).; ^{13}C NMR (101 MHz, $CDCl_3$): δ 171.3, 167.9, 139.0, 135.4, 129.3 (x 2), 129.0 (x 2), 127.9, 127.6, 127.5, 127.4, 56.2, 53.9, 52.7, 42.1, 37.8; MS (ESI+): *m/z* $[M+H]^+$ calcd. for $C_{17}H_{21}N_2O_5S_2$: 397.09, found 397.55; $[M+Na]^+$ calcd. for $C_{17}H_{20}N_2NaO_5S_2$: 419.07, found 419.16; $[\alpha]_D^{21.2}$ = +0.1342 ± 0.0041 (*c* 1.00, CH_2Cl_2).

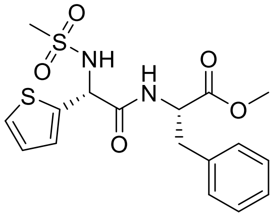

Dipeptide **3'f** (R_f = 0.51 in Hex/AcOEt 1:1) was obtained as colorless oil enriched in the first enantiomer product **3f** (yield: 44.0 mg; 37%); ^{1}H NMR (400 MHz, $CDCl_3$): δ 7.40-7.35 (m, 1H), 7.21-.10 (m, 3H), 7.10-7.03 (m, 1H), 7.03-6.99 (m, 1H), 6.81-6.68 (m, 2H), 6.15 (d, *J* = 8.1 Hz, 1H), 5.83, 5.36 (AX system, *J* = 5.3 Hz, 2H), 4.92-4.86 (m, 1H), 3.74 (s, 3H), 3.03 (d, *J* = 5.6 Hz, 2H), 2.73 (s, 3H); ^{13}C NMR (101 MHz, $CDCl_3$): δ 171.2, 167.5, 139.6, 135.0, 129.2 (x 2), 128.8 (x 2), 128.0, 127.5, 127.4, 127.3, 56.1, 53.5, 52.7, 42.1, 37.6; HRMS (ESI+): *m/z* $[M+Na]^+$ calcd. for $C_{17}H_{20}N_2NaO_5S_2$: 419.0711, found 419.0710.

Methyl (2-(furan-2-yl)-2-(methylsulfonamido)acetyl)-*L*-phenylalaninate (**3g**/**3'g**)

General Procedure 4. Imine **1g** (52.0 mg, 0.30 mmol, 1.0 equiv.), DHP-(*L*)-Phe **2a** (178.8 mg, 0.39 mmol, 1.3 equiv.), **4CzIPN** (5.9 mg, 0.0075 mmol, 0.025 equiv.), $BF_3{\cdot}OEt_2$ (37 µL, 0.30 mmol, 1.0 equiv.) in dry CH_2Cl_2 (3.0 mL, 0.1 M). Flash chromatography (Hex/EtOAc, 7:3→1:1).

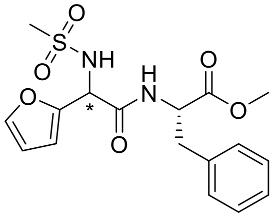

Dipeptide **3g**/**3'g** (R_f = 0.43 in Hex/EtOAc 1:1) was obtained as a light-yellow solid (yield: 57.0 mg, 44%) as a mixture of inseparable diastereoisomers; ^{1}H NMR (400 MHz, $CDCl_3$): δ 7.46-7.35 (m, 2H), 7.33-7.23 (m, 3H), 7.22-7.15 (m, 3H), 7.11-7.04 (m, 2H), 6.88-6.80 (m, 2H), 6.49-6.34 (m, 5H), 6.34-6.24 (m, 1H), 5.70, 5.19 (AX system, *J* = 6.1 Hz, 2H), 5.60, 5.15 (AX system, *J* = 6.4 Hz, 2H), 4.94-4.77 (m, 2H), 3.75 (s, 3H), 3.71 (s, 3H), 3.25-3.03 (m, 4H), 2.78 (s, 3H), 2.77 (s, 3H); ^{13}C NMR (101 MHz, $CDCl_3$): *δ* 171.3, 171.2, 166.4, 166.1, 148.7, 148.5, 143.8, 143.8, 135.5, 135.1, 129.3 (x 2), 129.3 (x 2), 128.9 (x 2), 128.8 (x 2), 127.5, 127.4, 111.1 (x 2), 110.2, 110.0, 54.5, 54.3, 53.9, 53.6, 52.7, 52.7, 41.7, 41.6, 37.8, 37.7; HRMS (ESI+): *m/z* $[M+Na]^+$ calcd. for $C_{17}H_{20}N_2NaO_6S$: 403.0940, found 403.0934.

Methyl (2-((1,1-dimethylethyl)sulfonamido)-2-phenylacetyl)-*L*-phenylalaninate (**3h**/**3'h**)

General Procedure 4. Imine **1h** (67.6 mg, 0.30 mmol, 1.0 equiv.), DHP-(*L*)-Phe **2a** (178.8 mg, 0.39 mmol, 1.3 equiv.), **4CzIPN** (5.9 mg, 0.0075 mmol, 0.025 equiv.), $BF_3{\cdot}OEt_2$ (37 µL, 0.30 mmol, 1.0 equiv.) in dry CH_2Cl_2 (3.0 mL, 0.1 M). Flash chromatography (Hex/EtOAc, 85:15→1:1).

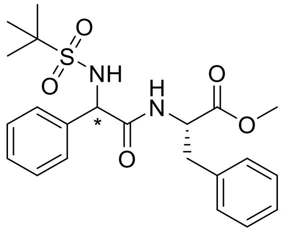

Dipeptide **3h**/**3'h** (R_f = 0.70 in Hex/AcOEt 1:1) was obtained as a pale yellow solid (yield: 84.0 mg, 65%) as a mixture of inseparable diastereoisomers; ^{1}H NMR (400 MHz, $CDCl_3$): δ 7.42-7.20 (m, 13H), 7.17-6.97 (m, 5H), 6.67-6.52 (m, 2H), 6.29 (d, *J* = 7.8 Hz, 1H), 6.16 (d, *J* = 8.0 Hz, 1H), 5.69, 5.11 (AX system, *J* = 6.8 Hz, 2H), 5.56, 5.08 (AX system, *J* = 7.3 Hz, 2H), 4.91 (dt, *J* = 8.0, 5.5 Hz, 1H), 4.81 (dt, *J* = 7.7, 5.9 Hz, 1H), 3.72 (s, 3H), 3.65 (s, 3H), 3.17 (dd, *J* = 13.9, 5.7 Hz, 1H), 3.05 (dd, *J* = 13.9, 6.2 Hz, 1H), 2.96 (d, *J* = 5.5 Hz, 2H), 1.27 (s, 9H), 1.25 (s, 9H); ^{13}C NMR (101 MHz, $CDCl_3$): *δ* 171.4, 171.3, 169.7, 169.3, 138.4, 137.8, 135.5, 135.1, 129.4 (x 2), 129.3 (x 2), 129.2 (x 2), 129.2 (x 2), 128.8 (x 2), 128.8, 128.7, 128.6 (x 2), 127.6 (x 2), 127.5 (x 2), 127.3, 127.1, 60.9, 60.7, 60.1, 60.0, 53.8, 53.4, 52.6, 52.5, 37.7, 37.6, 24.1 (x 3), 24.1 (x 3); HRMS (ESI+): *m/z* $[M+Na]^+$ calcd. for $C_{22}H_{28}N_2NaO_5S$: 455.1617, found 455.1616.

Methyl (2-((4-methylphenyl)sulfonamido)-2-phenylacetyl)-*L*-phenylalaninate (**3i**/**3'i**)

General Procedure 4. Imine **1i** (77.8 mg, 0.30 mmol, 1.0 equiv.), DHP-(*L*)-Phe **2a** (178.8 mg, 0.39 mmol, 1.3 equiv.), **4CzIPN** (5.9 mg, 0.0075 mmol, 0.025 equiv.), $BF_3{\cdot}OEt_2$ (37 µL, 0.30 mmol, 1.0 equiv.) in dry CH_2Cl_2 (3.0 mL, 0.1 M). Flash chromatography (Hex/EtOAc, 8:2→65:35).

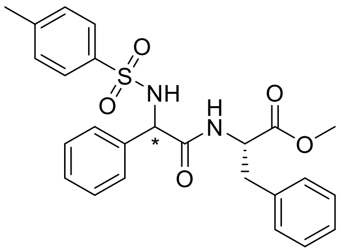

Dipeptide **3i**/**3'i** (R_f = 0.50 in Hex/AcOEt 6:4) was obtained as a pale yellow solid (yield: 107.6 mg, 77%) as a mixture of inseparable diastereoisomers; ^{1}H NMR (400 MHz, $CDCl_3$): δ 7.63-7.52 (m, 4H), 7.34-7.00 (m, 20H), 6.98-6.89 (m, 2H), 6.69-6.58 (m, 2H), 6.15 (d, *J* = 7.6 Hz, 1H), 6.07 (d, *J* = 8.1 Hz, 1H), 5.90 (d, *J* = 5.0 Hz, 1H), 5.83 (d, *J* = 5.2 Hz, 1H), 4.89-4.61 (m, 4H), 3.71 (s, 3H), 3.64 (s, 3H), 3.10-2.87 (m, 4H), 2.37 (s, 6H); ^{13}C NMR (101 MHz, $CDCl_3$): *δ* 171.2, 171.1, 168.6, 168.3, 143.7, 143.6, 136.8, 136.5, 136.1, 135.5, 134.9, 129.6 (x 2), 129.6 (x 2), 129.3 (x 2), 129.3 (x 2), 129.2 (x 2), 129.1 (x 2), 128.9, 128.9, 128.8 (x 2), 128.7 (x 2), 127.7 (x 2), 127.6 (x 2), 127.5 (x 2), 127.4 (x 2), 127.2 (x 2), 60.6, 60.6, 53.7, 53.2, 52.6, 52.6, 37.7, 37.7, 21.7, 21.6; HRMS (ESI+): *m/z* $[M+Na]^+$ calcd. for $C_{25}H_{26}N_2NaO_5S$: 489.1460, found 489.1466.

Methyl (2-((4-nitrophenyl)sulfonamido)-2-phenylacetyl)-*L*-phenylalaninate (**3j**/**3'j**)

General Procedure 4. Imine **1j** (87.1 mg, 0.30 mmol, 1.0 equiv.), DHP-(*L*)-Phe **2a** (178.8 mg, 0.39 mmol, 1.3 equiv.), **4CzIPN** (5.9 mg, 0.0075 mmol, 0.025 equiv.), $BF_3{\cdot}OEt_2$ (37 µL, 0.30 mmol, 1.0 equiv.) in dry CH_2Cl_2 (3.0 mL, 0.1 M). Flash chromatography (Hex/EtOAc, 95:5→8:2).

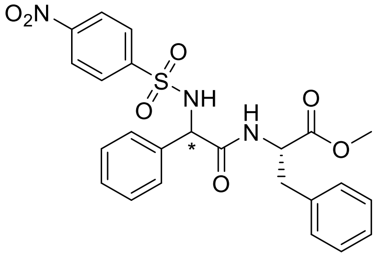

Dipeptide **3j**/**3'j** (R_f = 0.30 in Hex/AcOEt 8:2) was obtained as a light-yellow solid (yield: 25.1 mg, 17%) as a mixture of inseparable diastereoisomers; ^{1}H NMR (300 MHz, $CDCl_3$): δ 8.14-8.01 (m, 4H), 7.76-7.65 (m, 4H), 7.25-6.95 (m, 18H), 6.58-6.48 (m, 2H), 6.39 (d, *J* = 5.3 Hz, 1H), 6.33 (d, *J* = 4.9 Hz, 1H), 6.00-5.83 (m, 2H), 4.99-4.89 (m, 2H), 4.85-4.74 (m, 1H), 4.74-4.62 (m, 1H), 3.72 (s, 3H), 3.62 (s, 3H), 3.16-2.93 (m, 2H), 2.90 (d, *J* = 5.4 Hz, 2H); ^{13}C NMR (75 MHz, $CDCl_3$): *δ* 171.2, 171.1, 168.2, 167.7, 149.8 (x 2), 146.2, 146.2, 135.6, 135.3, 135.1, 134.8, 129.3 (x 2), 129.3 (x 2), 129.2 (x 2), 129.2 (x 2), 129.1 (x 2), 128.9 (x 2), 128.7 (x 2), 128.4 (x 2), 128.4 (x 2), 128.0 (x 2), 127.8 (x 2), 127.5, 127.3, 124.0 (x 2), 123.9 (x 2), 60.6, 60.4, 53.9, 53.2, 52.8, 52.6, 37.6, 37.6; HRMS (ESI+): *m/z* $[M+Na]^+$ calcd. for $C_{24}H_{23}N_3NaO_7S$: 520.1154, found 520.1154.

Methyl (2-(((1R,3R)-adamantan-1-yl)amino)-2-phenylacetyl)-*L*-phenylalaninate (**3k**/**3'k**)

General Procedure 4. Imine **1k** (71.8 mg, 0.30 mmol, 1.0 equiv.), DHP-(*L*)-Phe **2a** (178.8 mg, 0.39 mmol, 1.3 equiv.), **4CzIPN** (5.9 mg, 0.0075 mmol, 0.025 equiv.), $BF_3{\cdot}OEt_2$ (37 µL, 0.30 mmol, 1.0 equiv.) in dry CH_2Cl_2 (3.0 mL, 0.1 M). Flash chromatography (Hex/EtOAc, 8:2→1:1).

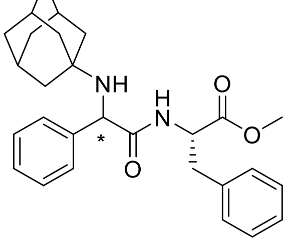

Dipeptide **3k**/**3'k** (R_f = 0.58 in Hex/AcOEt 7:3) was obtained as light orange solid (yield: 96.5 mg, 72%) as a mixture of inseparable diastereoisomers; ^{1}H NMR (400 MHz, $CDCl_3$) δ 8.50 (d, *J* = 8.3 Hz, 1H), 8.34 (d, *J* = 7.7 Hz, 1H), 7.42-7.22 (m, 17H), 7.22-7.13 (m, 3H), 7.10 (m, 2H), 5.01-4.86 (m, 2H), 4.44 (s, 1H), 4.40 (s, 1H), 3.78 (s, 3H), 3.77 (s, 3H), 3.37-3.22 (m, 2H), 3.14 (dd, *J* = 14.1, 7.9 Hz, 2H), 2.04 (m, 6H), 1.79-1.35 (m, 24H); ^{13}C NMR (101 MHz, $CDCl_3$) δ 174.4, 174.0, 172.4, 172.0, 141.9, 141.4, 136.3, 136.2, 129.4 (x 2), 129.1 (x 2), 129.0 (x 2), 128.9 (x 2), 128.8 (x 2), 128.6 (x 2), 127.7, 127.7, 127.6 (x 2), 127.3 (x 2), 127.3, 127.1, 59.5, 59.2, 53.0, 52.7, 52.3, 52.2, 52.1, 51.9, 42.9 (x 3), 42.7 (x 3), 38.2, 37.7, 36.5 (x 3), 36.4 (x 3), 29.6 (x 3), 29.5 (x 3); HRMS (ESI+): *m/z* $[M+H]^+$ calcd. for $C_{28}H_{35}N_2O_3$: 447.2648, found 447.2647; $[M+Na]^+$ calcd. for $C_{28}H_{34}N_2NaO_3$: 469.2467, found 469.2459.

Methyl (2-phenyl-2-(p-tolylamino)acetyl)-*L*-phenylalaninate (**3l**/**3'l**)

General Procedure 4. Imine **1l** (58.8 mg, 0.30 mmol, 1.0 equiv.), DHP-(*L*)-Phe **2a** (178.8 mg, 0.39 mmol, 1.3 equiv.), **4CzIPN** (5.9 mg, 0.0075 mmol, 0.025 equiv.), $BF_3 \cdot OEt_2$ (37 µL, 0.30 mmol, 1.0 equiv.) in dry CH_2Cl_2 (3.0 mL, 0.1 M). Flash chromatography (Hex/EtOAc, 95:5→1:1).

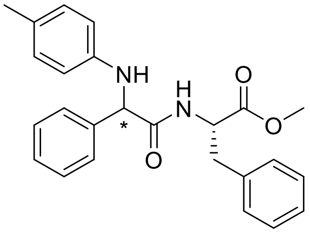

Dipeptide **3l**/**3'l** (R_f =0.48 in Hex/AcOEt 1:1) was obtained as brown solid (yield: 83.2 mg, 69%) as a mixture of inseparable diastereoisomers; ^1H NMR (400 MHz, $CDCl_3$) δ 7.47-7.40 (m, 2H), 7.40-7.32 (m, 3H), 7.32-7.24 (m, 6H), 7.24-7.11 (m, 6H), 7.11-6.95 (m, 9H), 6.77-6.66 (m, 2H), 6.60-6.44 (m, 4H), 5.02-4.93 (m, 1H), 4.93-4.82 (m, 1H), 4.74 (s, 1H), 4.67 (s, 1H), 3.71 (s, 3H), 3.64 (s, 3H), 3.25 (dd, *J* = 14.1, 5.4 Hz, 1H), 3.11-2.88 (m, 3H), 2.27 (s, 3H), 2.25 (s, 3H); ^{13}C NMR (101 MHz, $CDCl_3$) δ 171.7, 171.5, 171.4, 170.9, 144.4, 144.2, 138.7, 138.5, 136.1, 135.3, 129.9 (x 2), 129.8 (x 2), 129.3 (x 4), 129.2 (x 2), 129.2 (x 2), 128.7 (x 2), 128.7, 128.6, 128.6 (x 2), 128.5, 128.4, 127.7 (x 2), 127.4 (x 2), 127.1, 127.0, 114.4 (x 2), 114.0 (x 2), 64.9, 64.2, 53.2, 52.6, 52.4, 52.3, 37.9, 37.6, 20.5 (x 2); HRMS (ESI+): *m/z* $[M+H]^+$ calcd. for $C_{25}H_{27}N_2O_3$: 403.2022, found 403.2018; $[M+Na]^+$ calcd. for $C_{25}H_{26}N_2NaO_3$: 425.1841, found 425.1844.

Methyl (2-phenyl-2-(p-tolylamino)acetyl)-*L*-phenylalaninate (**3m**/**3'm**)

General Procedure 4. Imine **1l** (58.8 mg, 0.30 mmol, 1.0 equiv.), DHP-(*L*)-Val **2b** (160.1 mg, 0.39 mmol, 1.3 equiv.), **4CzIPN** (5.9 mg, 0.0075 mmol, 0.025 equiv.), $BF_3 \cdot OEt_2$ (37 µL, 0.30 mmol, 1.0 equiv.) in dry CH_2Cl_2 (3.0 mL, 0.1 M). Flash chromatography (CH_2Cl_2/EtOAc, 100:0→95:5).

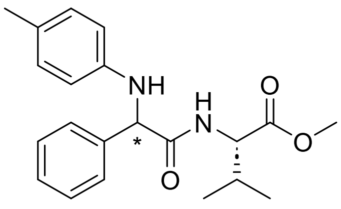

Dipeptide **3m**/**3'm** (R_f =0.43 in CH_2Cl_2/AcOEt 97:3) was obtained as brown liquid (yield: 63.8 mg, 60%) as a mixture of inseparable diastereoisomers; ^1H NMR (400 MHz, $CDCl_3$) δ 7.52-7.43 (m, 5H), 7.43-7.30 (m, 7H), 7.29-7.24 (m, 1H), 7.23-7.18 (m, 1H), 7.04-6.95 (m, 4H), 6.62-6.54 (m, 4H), 4.76 (s, 1H), 4.74 (s, 1H), 4.58 (dd, *J* = 9.3, 4.7 Hz, 1H), 4.53 (dd, *J* = 8.9, 4.9 Hz, 1H), 3.73 (s, 3H), 3.61 (s, 3H), 2.24 (s, 6H), 2.22-2.11 (m, 2H), 0.94-0.85 (m, 6H), 0.81 (d, *J* = 6.9 Hz, 3H), 0.69 (d, *J* = 6.9 Hz, 3H); ^{13}C NMR (101 MHz, $CDCl_3$) δ 172.4, 171.8, 171.7, 171.3, 144.5, 144.3, 139.1, 139.0, 129.9 (x 2), 129.9 (x 2), 129.4 (x 4), 128.8, 128.7 (x2) , 128.7, 127.7 (x 2), 127.4 (x 2), 114.5 (x 2), 114.1 (x 2), 65.2, 64.7, 57.4, 57.0, 52.3, 52.1, 31.3, 31.3, 20.6, 20.6, 19.2, 19.1, 18.0, 17.5 ; HRMS (ESI+): *m/z* $[M+H]^+$ calcd. for $C_{21}H_{27}N_2O_3$: 355.2022, found 355.2015; $[M+Na]^+$ calcd. for $C_{21}H_{26}N_2NaO_3$: 377.1841, found 377.1838.

3.4.2 Scope of DHPs (2b-2l) using imine 1a

Methyl (2-(methylsulfonamido)-2-phenylacetyl)-*L*-valinate (**3n/3’n**)

General Procedure 4. Imine **1a** (55.0 mg, 0.30 mmol, 1.0 equiv.), DHP-(*L*)-Val **2b** (160.1 mg, 0.39 mmol, 1.3 equiv.), **4CzIPN** (5.9 mg, 0.0075 mmol, 0.025 equiv.), $BF_3{\cdot}OEt_2$ (37 µL, 0.30 mmol, 1.0 equiv.) in dry CH_2Cl_2 (3.0 mL, 0.1 M). Flash chromatography (Hex/EtOAc, 9:1→1:1).

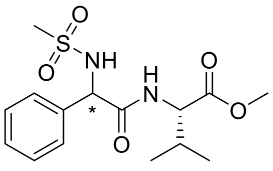

Dipeptide **3n/3’n** (R_f = 0.45 in Hex/AcOEt 1:1) was obtained as a white solid (yield: 77.4 mg, 75%) as a mixture of inseparable diastereoisomers; ^{1}H NMR (400 MHz, $CDCl_3$): δ 7.48-7.31 (m, 10H), 6.19 (d, *J* = 8.7 Hz, 2H), 5.99-5.86 (m, 2H), 5.15 (s, 1H), 5.13 (s, 1H), 4.54-4.45 (m, 2H), 3.73 (s, 3H), 3.64 (s, 3H), 2.75-2.64 (m, 6H), 2.22-1.99 (m, 2H), 0.92 (d, *J* = 6.9 Hz, 3H), 0.85 (d, *J* = 6.9 Hz, 3H), 0.71 (d, *J* = 6.9 Hz, 3H), 0.65 (d, *J* = 6.9 Hz, 3H); ^{13}C NMR (101 MHz, $CDCl_3$): δ 172.0, 171.7, 169.2, 169.1, 137.2, 136.5, 129.6 (x 4), 129.4, 129.3, 127.9 (x 2), 127.7 (x 2), 60.6, 60.5, 57.9, 57.6, 52.5, 52.4, 42.1, 41.9, 31.3, 31.3, 19.0, 18.9, 17.8, 17.4; HRMS (ESI+): *m/z* $[M+Na]^+$ calcd. for $C_{15}H_{22}N_2NaO_5S$: 365.1147, found 365.1146.

Methyl (2-(methylsulfonamido)-2-phenylacetyl)-*L*-alaninate (**3o/3’o**)

General Procedure 4. Imine **1a** (55.0 mg, 0.30 mmol, 1.0 equiv.), DHP-(*L*)-Ala **2c** (149.1 mg, 0.39 mmol, 1.3 equiv.), **4CzIPN** (5.9 mg, 0.0075 mmol, 0.025 equiv.), $BF_3{\cdot}OEt_2$ (37 µL, 0.30 mmol, 1.0 equiv.) in dry CH_2Cl_2 (3.0 mL, 0.1 M). Flash chromatography (Hex/EtOAc, 9:1→1:1).

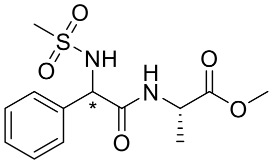

Dipeptide **3o/3’o** (R_f = 0.47 in Hex/AcOEt 1:1) was obtained as a white powder (yield: 60.0 mg, 64%) as a mixture of inseparable diastereoisomers; ^{1}H NMR (300 MHz, $CDCl_3$): δ 7.47-7.34 (m, 10H), 6.25 (d, *J* = 7.4 Hz, 1H), 6.20 (d, *J* = 7.3 Hz, 1H), 5.93-5.76 (m, 2H), 5.12-5.09 (m, 1H), 5.09-5.05 (m, 1H), 4.62-4.46 (m, 2H), 3.74 (s, 3H), 3.67 (s, 3H), 2.69 (s, 3H), 2.67 (s, 3H), 1.40 (d, *J* = 7.2 Hz, 3H), 1.30 (d, *J* = 7.2 Hz, 3H); ^{13}C NMR (75 MHz, $CDCl_3$): δ 172.9, 172.7, 168.8, 168.7, 136.8, 136.5, 129.6 (x 2), 129.6 (x 2), 129.5, 129.4, 128.0 (x 2), 127.8 (x 2), 60.6, 60.5, 52.8, 52.7, 48.8, 48.8, 42.2, 41.9, 18.3, 18.0; HRMS (ESI+): *m/z* $[M+Na]^+$ calcd. for $C_{13}H_{18}N_2NaO_5S$: 337.0834, found 337.0834.

Methyl (2-(methylsulfonamido)-2-phenylacetyl)-*L*-methioninate (**3p/3’p**)

General Procedure 4. Imine **1a** (55.0 mg, 0.30 mmol, 1.0 equiv.), DHP-(*L*)-Met **2d** (172.6 mg, 0.39 mmol, 1.3 equiv.), **4CzIPN** (5.9 mg, 0.0075 mmol, 0.025 equiv.), $BF_3{\cdot}OEt_2$ (37 µL, 0.30 mmol, 1.0 equiv.) in dry CH_2Cl_2 (3.0 mL, 0.1 M). Flash chromatography (CH_2Cl_2/EtOAc, 95:5→8:2) gave product **3p** pure, while **3’p** was obtained enriched in the first diastereoisomer.

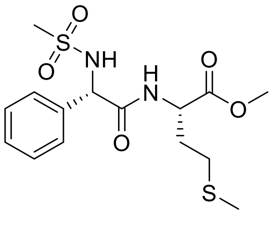

Dipeptide **3p** (R_f = 0.24 in CH_2Cl_2/EtOAc 95:5) was obtained as a white solid (yield: 31.8 mg; 28%); M.p. = 132.5-133.8 °C; ^{1}H NMR (400 MHz, $CDCl_3$): δ 7.47-7.33 (m, 5H), 6.51 (d, *J* = 7.7 Hz, 1H), 5.90 (s, 1H), 5.11 (s, 1H), 4.65 (td, *J* = 7.4, 4.9 Hz, 1H), 3.66 (s, 3H), 2.68 (s, 3H), 2.57-2.36 (m, 2H), 2.26-2.09 (m, 1H), 2.05 (s, 3H), 2.03-1.92 (m, 1H); ^{13}C NMR (101 MHz, $CDCl_3$): δ 171.7, 169.2, 136.4, 129.5 (x 2), 129.4, 128.0 (x 2), 60.6, 52.7, 52.3, 42.1, 31.0, 30.0, 15.6; HRMS (ESI+): *m/z* $[M+Na]^+$ calcd. for $C_{15}H_{22}N_2NaO_5S_2$: 397.0868, found 397.0862; $[\alpha_D{}^{20}]$ = +0.2569 ± 0.0071 (*c* 1.00, CH_2Cl_2).

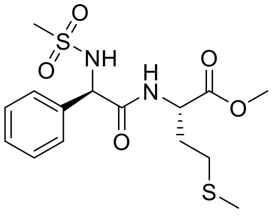

Dipeptide **3’p** (R_f = 0.21 in Hex/AcOEt 95:5) was obtained enriched with **3p** as a white solid (yield: 36.9 mg; 33%); ^{1}H NMR (400 MHz, $CDCl_3$): δ 7.49-7.33 (m, 3H), 7.25-7.17 (m, 2H), 6.60 (d, *J* = 8.0 Hz, 1H), 6.00-5.90 (m, 1H), 5.18-5.09 (m, 1H), 4.74-4.67 (m, 1H), 3.74 (s, 3H), 2.71 (s, 3H), 2.24-2.02 (m, 4H), 1.90 (s, 3H); ^{13}C NMR (101 MHz, $CDCl_3$): δ 172.0, 169.2, 137.0, 129.6 (x 2), 129.3, 127.7 (x 2), 60.5, 52.9, 52.0, 41.9, 31.0, 29.7, 15.4; MS (ESI+): *m/z* $[M+H]^+$ calcd. for $C_{15}H_{23}N_2O_5S_2$: 375.10, found 375.30; $[2M+Na]^+$ calcd. for $C_{30}H_{44}N_4NaO_{10}S_4$: 771.18, found 770.81.

Methyl O-(tert-butyl)-N-(2-(methylsulfonamido)-2-phenylacetyl)-*L*-allothreoninate (**3q**/**3'q**)

General Procedure 4. Imine **1a** (55.0 mg, 0.30 mmol, 1.0 equiv.), DHP-(*L*)-Thr **2e** (182.8 mg, 0.39 mmol, 1.3 equiv.), **4CzIPN** (5.9 mg, 0.0075 mmol, 0.025 equiv.), $BF_3{\cdot}OEt_2$ (37 µL, 0.30 mmol, 1.0 equiv.) in dry CH_2Cl_2 (3.0 mL, 0.1 M). Flash chromatography (Hex/EtOAc, 9:1→6:4).

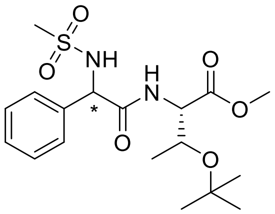

Dipeptide **3q**/**3'q** (R_f = 0.56 in Hex/AcOEt 1:1) was obtained as a white powder (yield: 100.0 mg, 83%) as a mixture of inseparable diastereoisomers; ^{1}H NMR (400 MHz, $CDCl_3$): δ 7.47-7.29 (m, 10H), 6.54 (d, J = 9.0 Hz, 1H), 6.44 (d, J = 8.9 Hz, 1H), 6.08-5.96 (m, 2H), 5.20-5.13 (m, 2H), 4.42-4.34 (m, 2H), 4.23-4.08 (m, 2H), 3.69 (s, 3H), 3.56 (s, 3H), 2.75 (s, 3H), 2.70 (s, 3H), 1.16-1.14 (m, 3H), 1.01-0.96 (m, 18H), 0.86 (d, J = 6.2 Hz, 3H); ^{13}C NMR (101 MHz, $CDCl_3$): δ 170.8, 170.5, 169.7 (x 2), 137.1, 136.6, 129.3 (x 2), 129.3 (x 2), 129.1, 129.0, 128.0 (x 2), 127.6 (x 2), 74.3, 74.2, 67.2, 67.1, 60.6, 60.5, 58.4, 58.3, 52.4, 52.2, 42.0, 41.7, 28.3 (x 3), 28.2 (x 3), 21.3, 20.8; HRMS (ESI+): *m/z* $[M+Na]^+$ calcd. for $C_{18}H_{28}N_2NaO_6S$: 423.1566, found 423.1567.

Methyl (2-(methylsulfonamido)-2-phenylacetyl)-*L*-prolinate (**3r**/**3'r**)

General Procedure 4. Imine **1a** (55.0 mg, 0.30 mmol, 1.0 equiv.), DHP-(*L*)-Pro **2f** (159.3 mg, 0.39 mmol, 1.3 equiv.), **4CzIPN** (5.9 mg, 0.0075 mmol, 0.025 equiv.), $BF_3{\cdot}OEt_2$ (37 µL, 0.30 mmol, 1.0 equiv.) in dry CH_2Cl_2 (3.0 mL, 0.1 M). Flash chromatography (Hex/EtOAc, 8:2→4:6).

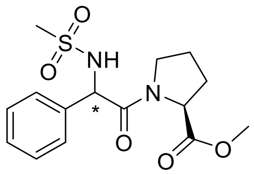

Dipeptide **3r**/**3'r** (R_f = 0.42 in Hex/AcOEt 1:1) was obtained as light yellow solid (yield: 23.0 mg, 23%) as a mixture of inseparable diastereoisomers; ^{1}H NMR (400 MHz, $CDCl_3$): δ 7.51-7.28 (m, 10H), 5.92 (d, J = 7.0 Hz, 1H), 5.87 (d, J = 7.9 Hz, 1H), 5.28-5.26 (m, 1H), 5.26-5.22 (m, 1H), 4.59-4.53 (m, 1H), 4.53-4.46 (m, 1H), 3.74 (s, 3H), 3.73-3.71 (m, 1H), 3.70 (s, 3H), 3.57-3.49 (m, 1H), 3.12-3.03 (m, 2H), 2.71 (s, 3H), 2.61 (s, 3H), 2.28-1.76 (m, 8H); ^{13}C NMR (101 MHz, $CDCl_3$): δ 172.1, 171.9, 168.1, 167.6, 135.6, 135.5, 129.5 (x 2), 129.3 (x 2), 129.2 (x 2), 128.7 (x 2), 128.2 (x 2), 59.6, 59.5, 59.3, 59.2, 52.6, 52.4, 46.9 (x 2), 42.3, 41.6, 29.0, 28.9, 25.0, 24.6; HRMS (ESI+): *m/z* $[M+Na]^+$ calcd. for $C_{15}H_{20}N_2NaO_5S$: 363.0991, found 363.0991.

Methyl (2-(methylsulfonamido)-2-phenylacetyl)-*L*-serinate (**3s**/**3's**)

General Procedure 4. Imine **1a** (55.0 mg, 0.30 mmol, 1.0 equiv.), DHP-(*L*)-Ser **2g** (155.4 mg, 0.39 mmol, 1.3 equiv.), **4CzIPN** (5.9 mg, 0.0075 mmol, 0.025 equiv.), $BF_3{\cdot}OEt_2$ (37 µL, 0.30 mmol, 1.0 equiv.) in dry CH_2Cl_2 (3.0 mL, 0.1 M). Flash chromatography (CH_2Cl_2/EtOAc, 9:1→4:6).

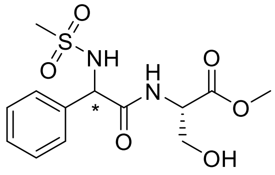

Dipeptide **3s**/**3's** (R_f = 0.19 in CH_2Cl_2/AcOEt 1:1) was obtained as white solid (yield: 39.6 mg, 40%) as a mixture of inseparable diastereoisomers; ^{1}H NMR (500 MHz, CD_3CN): δ 7.49-7.33 (m, 11H), 7.23 (d, J = 7.8 Hz, 1H), 6.30-6.22 (m, 2H), 5.18-5.12 (m, 2H), 4.52-4.44 (m, 2H), 3.90-3.82 (m, 2H), 3.82-3.74 (m, 1H), 3.71 (s, 3H), 3.70-3.64 (m, 1H), 3.63 (s, 3H), 3.28-3.17 (m, 2H), 2.83 (s, 3H), 2.78 (s, 3H); ^{13}C NMR (126 MHz, CD_3CN): δ171.6, 171.4, 170.9, 170.5, 138.4, 138.3, 129.9 (x2), 129.8 (x2), 129.5, 129.5, 128.6 (x2), 128.5 (x2), 62.5, 62.4, 61.1, 61.0, 55.9, 55.9, 53.0, 52.9, 41.6, 41.4; HRMS (ESI+): *m/z* $[M+Na]^+$ calcd. for $C_{13}H_{18}N_2NaO_6S$: 353.0783, found 353.0779.

Methyl (2-(methylsulfonamido)-2-phenylacetyl)-*L*-tyrosinate (3t/3't)

General Procedure 4. Imine **1a** (55.0 mg, 0.30 mmol, 1.0 equiv.), DHP-(*L*)-Tyr **2k** (185.1 mg, 0.39 mmol, 1.3 equiv.), **4CzIPN** (5.9 mg, 0.0075 mmol, 0.025 equiv.), $BF_3{\cdot}OEt_2$ (37 µL, 0.30 mmol, 1.0 equiv.) in dry CH_2Cl_2 (3.0 mL, 0.1 M). Flash chromatography (Hex/EtOAc, 8:2→1:1).

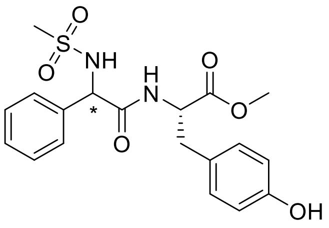

Dipeptide **3t/3'tt** (R_f = 0.48 in Hex/AcOEt 1:1) was obtained as white solid (yield: 47.5 mg, 39%) as a mixture of inseparable diastereoisomers; ^{1}H NMR (400 MHz, CD_3CN): δ7.44-7.21 (m, 10H), 7.13-6.96 (m, 4H), 6.92 (s, 1H), 6.87 (s, 1H), 6.81-6.66 (m, 4H), 6.62-6.50 (m, 2H), 6.21, 5.04 (AX system, J = 8.5 Hz, 2H), 6.15, 5.00 (AX system, J = 8.3 Hz, 2H), 4.67-4.58 (m, 2H), 3.68 (s, 3H), 3.60 (s, 3H), 3.13-2.79 (m, 4H), 2.75 (s, 3H), 2.65 (s, 3H); ^{13}C NMR (101 MHz, CD_3CN): δ 172.5, 172.4, 170.3, 170.3, 156.8, 156.7, 138.3, 138.2, 131.4, 131.2, 129.8, 129.8, 129.5, 129.4, 128.7, 128.5, 128.3, 128.2, 116.2, 116.1, 61.0, 60.9, 55.0, 54.9, 52.9, 52.8, 41.5, 41.3, 37.0, 36.9; HRMS (ESI+): *m/z* $[M+Na]^+$ calcd. for $C_{19}H_{22}N_2NaO_6S$: 429.1096, found 429.1095

Methyl N^6-(*tert*-butoxycarbonyl)-N^2-(2-(methylsulfonamido)-2-phenylacetyl)-*L*-lysinate (3u/3'u)

General Procedure 4. Imine **1a** (55.0 mg, 0.30 mmol, 1.0 equiv.), DHP-(*L*)-Lys **2j** (210.5 mg, 0.39 mmol, 1.3 equiv.), **4CzIPN** (5.9 mg, 0.0075 mmol, 0.025 equiv.), $BF_3{\cdot}OEt_2$ (37 µL, 0.30 mmol, 1.0 equiv.) in dry CH_2Cl_2 (3.0 mL, 0.1 M). Flash chromatography (Hex/EtOAc, 85:15→4:6).

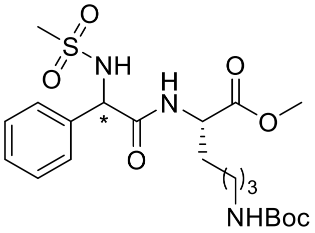

Dipeptide **3u/3'u** (R_f = 0.33 in Hex/AcOEt 1:1) was obtained as light-yellow solid (yield: 84.9 mg, 60%) as a mixture of inseparable diastereoisomers; ^{1}H NMR (500 MHz, $CDCl_3$): δ 7.51-7.30 (m, 10H), 6.56 (d, J = 8.0 Hz, 1H), 6.50 (d, J = 7.7 Hz, 1H), 6.14-5.91 (m, 2H), 5.19--5.09 (m, 2H), 4.66 (s, 1H), 4.58-4.46 (m, 3H), 3.72 (s, 3H), 3.63 (s, 3H), zzzzzzzzzz3.15-3.01 (m, 2H), 3.01-2.86 (m, 2H), 2.71 (s, 3H), 2.69 (s, 3H), 1.93-1.51 (m, 5H), 1.50-1.39 (m, 19H), 1.31 (q, J = 7.3 Hz, 4H), 1.04 (p, J = 7.8 Hz, 2H); ^{13}C NMR (126 MHz, $CDCl_3$): δ 172.4, 172.1, 169.3 (x2), 156.2 (x2), 137.1, 136.5, 129.5 (x4), 129.4, 129.3, 127.9 (x2), 127.7 (x2), 60.5, 60.4, 52.8, 52.7, 52.6, 52.5, 42.1, 41.8, 40.1 (x2), 31.6, 31.5, 29.8, 29.7, 29.4 (x2), 28.6 (x6), 22.6, 22.2; HRMS (ESI+): *m/z* $[M+Na]^+$ calcd. for $C_{21}H_{33}N_3NaO_7S$: 494.1937, found 494.1938.

Methyl (2-(methylsulfonamido)-2-phenylacetyl)-*L*-tryptophanate (3v/3'v)

General Procedure 4. Imine **1a** (55.0 mg, 0.30 mmol, 1.0 equiv.), DHP-(*L*)-Trp **2i** (194.1 mg, 0.39 mmol, 1.3 equiv.), **4CzIPN** (5.9 mg, 0.0075 mmol, 0.025 equiv.), $BF_3{\cdot}OEt_2$ (37 µL, 0.30 mmol, 1.0 equiv.) in dry CH_2Cl_2 (3.0 mL, 0.1 M). Flash chromatography (Hex/EtOAc, 8:2→3:7).

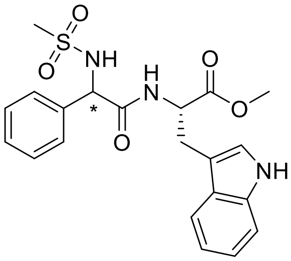

Dipeptide **3v/3'v** (R_f = 0.28 in Hex/AcOEt 1:1) was obtained as white solid (yield: 77.3 mg, 60%) as a mixture of inseparable diastereoisomers; ^{1}H NMR (400 MHz, $CDCl_3$): δ 8.33 (s, 1H), 8.15 (s, 1H), 7.56-7.41 (m, 1H), 7.39-7.20 (m, 13H), 7.20-7.06 (m, 3H), 7.06-6.94 (m, 1H), 6.90 (d, J = 2.4 Hz, 1H), 6.54 (s, 1H), 6.52 (s, 1H), 6.43 (d, J = 2.4 Hz, 1H), 6.05 (d, J = 6.6 Hz, 1H), 5.98 (d, J = 6.6 Hz, 1H), 5.08-5.00 (m, 2H), 4.95-4.77 (m, 2H), 3.63 (s, 3H), 3.60 (s, 3H), 3.40-3.03 (m, 4H), 2.59 (s, 3H), 2.51 (s, 3H); ^{13}C NMR (101 MHz, $CDCl_3$): δ 172.0, 171.8, 169.1, 169.1, 136.9, 136.4, 136.2, 136.1, 129.4 (x2), 129.3 (x2), 129.1, 129.0, 127.8 (x2), 127.7 (x2), 127.4, 127.2, 123.4, 123.3, 122.3, 122.3, 119.8, 119.8, 118.4, 118.3, 111.6, 111.4, 109.3, 108.9, 60.4, 60.4, 53.4, 52.9, 52.7, 52.6, 41.6, 41.5, 27.4, 27.3; HRMS (ESI+): *m/z* $[M+Na]^+$ calcd. for $C_{21}H_{23}N_3NaO_5S$: 452.1256, found 452.1257.

(3*S*)-dimethyl (2-(methylsulfonamido)-2-phenylacetyl)-L-aspartate (3w/3'w)

General Procedure 4. Imine **1a** (55.0 mg, 0.30 mmol, 1.0 equiv.), DHP-(*L*)-Asp **2h** (171.08 mg, 0.39 mmol, 1.3 equiv.), **4CzIPN** (5.9 mg, 0.0075 mmol, 0.025 equiv.), $BF_3 \cdot OEt_2$ (37 µL, 0.30 mmol, 1.0 equiv.) in dry CH_2Cl_2 (3.0 mL, 0.1 M). Flash chromatography (Hex/EtOAc, 8:2→1:1).

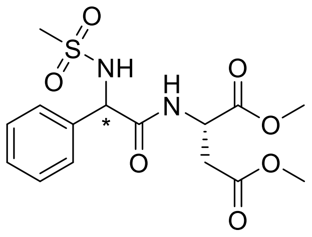

Dipeptide **3w/3'w** (R_f = 0.14 in Hex/AcOEt 1:1) was obtained as white solid (yield: 78.2 mg, 70%) as a mixture of inseparable diastereoisomers; ^1H NMR (500 MHz, $CDCl_3$): δ 7.46-7.33 (m, 10H), 6.81 (d, *J* = 8.3 Hz, 1H), 6.77 (d, *J* = 8.0 Hz, 1H), 5.95-5.84 (m, 2H), 5.13 (d, *J* = 5.9 Hz, 1H), 5.11 (d, *J* = 5.7 Hz, 1H), 4.85-4.75 (m, 2H), 3.74 (s, 3H), 3.66 (s, 3H), 3.62 (s, 3H), 3.51 (s, 3H), 3.05-2.81 (m, 3H), 2.76-2.70 (m, 4H), 2.69 (s, 3H); ^{13}C NMR (126 MHz, $CDCl_3$): δ 171.5, 171.1, 170.6, 170.5, 169.2, 169.1, 136.8, 136.4, 129.5 (x4), 129.4, 129.2, 127.9 (x2), 127.7 (x2), 60.5, 60.5, 53.2, 53.0, 52.3, 52.2, 49.1 (x2), 42.1, 41.8, 35.7, 35.6; HRMS (ESI+): *m/z* $[M+Na]^+$ calcd. For $C_{15}H_{20}N_2NaO_7S$: 395,0883, found 395.0886.

Methyl (2-(methylsulfonamido)-2-phenylacetyl)-*L*-phenylalanyl-*L*-leucinate (3x/3'x)

General Procedure 4. Imine **1a** (55.0 mg, 0.30 mmol, 1.0 equiv.), DHP-(*L*)-Phe-(*L*)-Leu **2l** (222.9 mg, 0.39 mmol, 1.3 equiv.), **4CzIPN** (5.9 mg, 0.0075 mmol, 0.025 equiv.), $BF_3 \cdot OEt_2$ (37 µL, 0.30 mmol, 1.0 equiv.) in dry CH_2Cl_2 (3.0 m L, 0.1 M). One diastereoisomer was precipitated directly from the crude reaction mixture using CH_2Cl_2/Hex, while the second one was purified by flash chromatography (Hex/AcOEt, 95:5→3:7). The two diastereomers were found to have a completely different solubility. The first one is soluble only in DMSO, while the second one in chlorinated solvents.

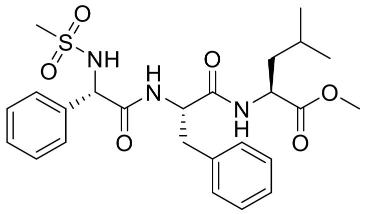

Tripeptide **3x** was precipitated as a white solid from the crude mixture (yield: 31.7 mg; 21%). ^1H NMR (400 MHz, DMSO-d_6): δ 8.59, 5.00 (AX system, *J* = 8.6 Hz, 2H), 8.34 (d, *J* = 7.7 Hz, 1H), 7.86-7.80 (m, 1H), 7.55-7.48 (m, 1H), 7.46-7.36 (m, 1H), 7.35-7.15 (m, 8H), 4.75-4.59 (m, 1H), 4.33-4.19 (m, 1H), 3.59 (s, 3H), 3.06 (dd, *J* = 13.9, 4.5 Hz, 1H), 2.80 (dd, *J* = 13.9, 10.0 Hz, 1H), 2.46 (s, 3H), 1.60-1.39 (m, 3H), 0.85 (d, *J* = 6.1 Hz, 3H), 0.77 (d, *J* = 6.1 Hz, 3H); ^{13}C NMR (101 MHz, DMSO-d_6): δ 172.6, 170.8, 169.1, 137.72, 137.5, 129.2 (x2), 128.2 (x2), 128.1 (x2), 127.6, 127.2 (x2), 126.3, 59.4, 53.5, 51.8, 50.2, 40.2, 39.6, 37.5, 24.1, 22.7, 21.2.; HRMS (ESI+): *m/z* $[M+Na]^+$ calcd. for $C_{25}H_{33}N_3NaO_6S$: 526.1988, found 526.1986.

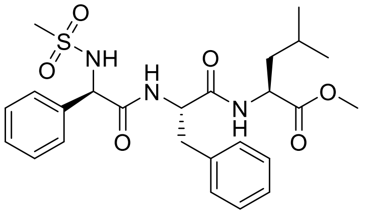

Tripeptide **3'x** (R_f = 0.36 in Hex/AcOEt 1:1) was obtained as a white solid (yield: 36.2 mg; 24%). ^1H NMR (400 MHz, $CDCl_3$): δ 7.43-7.33 (m, 3H), 7.33-7.27 (m, 1H), 7.25-7.20 (m, 1H), 7.20-7.06 (m, 3H), 6.95-6.83 (m, 2H), 6.51 (d, *J* = 8.0 Hz, 1H), 6.38 (d, *J* = 7.7 Hz, 1H), 6.09, 5.08 (AX system, *J* = 6.0 Hz, 2H), 4.77-4.71 (m, 2H), 4.58-4.46 (m, 1H), 3.71 (s, 3H), 2.99-2.86 (m, 2H), 2.64 (s, 3H), 1.62-1.44 (m, 3H), 0.96-0.83 (m, 6H); ^{13}C NMR (101 MHz, $CDCl_3$): δ 172.9, 170.2, 169.3, 136.6, 135.7, 129.6 (x 2), 129.3 (x 2), 129.0, 128.7 (x 2), 127.8 (x 2), 127.1, 60.6, 54.5, 52.5, 51.2, 42.0, 41.4, 38.2, 24.9, 22.8, 22.1; MS (ESI+): *m/z* $[M+H]^+$ calcd. for $C_{25}H_{34}N_3O_6S$: 504.22, found 504.91; $[M+Na]^+$ calcd. for $C_{25}H_{33}N_3NaO_6S$: 526.20, found 526.10; (ESI–): *m/z* $[M-H]^-$ calcd. for $C_{25}H_{32}N_3O_6S$: 502.21, found 502.54.

4. Solid Phase Peptide Synthesis (SPPS)

The different synthetic seps adopted for the SPPS are presented in Scheme S6.

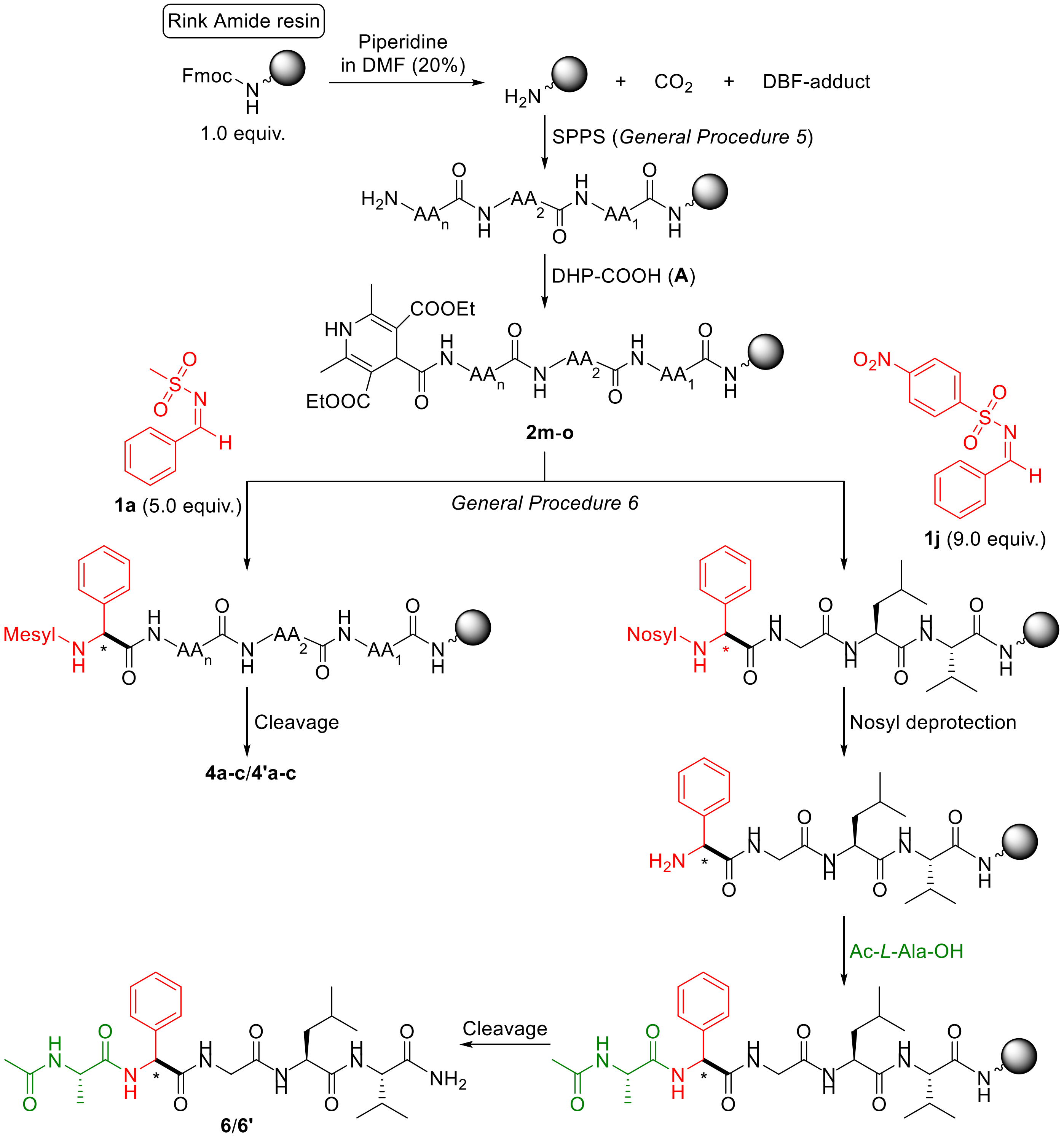

Scheme S6. General SPPS approach.

4.1 GP5. Synthesis of DHP functionalized peptides 2h-j anchored on Rink Amide resin

General Procedure 5. The synthesis of peptides **2m-o** was performed on Rink Amide MBHA resin (100-200 mesh, 0.3-0.8 mmol/g loading, 1.0 equiv.), using standard Fmoc chemistry protocols. The Fmoc removal was performed with a 20% piperidine solution in *N*,*N*-dimethylformamide (5 + 15 minutes), while each amino acid (5.0 equiv.) was activated using DIC (5.0 equiv.) and Oxyma Pure (5.0 equiv.) in DMF (4 mL) before addition to the resin. The only coupling performed with a different approach was with Fmoc-Aib-OH (3.0 equiv.), which was activated using DIC (5.0 equiv.) and HOBt (5.0 equiv.) in DMF (4 mL). After the addition of each amino acid, a Kaiser test was carried out to verify the success of the coupling. The following step was the derivatization of the *N*-terminus using the DHP-COOH (**A**) carried out by previously activating the carboxylic group using DIC and Oxyma Pure, as described above. The DHP-containing peptides were confirmed with ESI analyses as a result of a minicleavage (100 µl, TFA/TIS/H_2O 95:2.5:2.5); these sequences are presented in Figure S6.

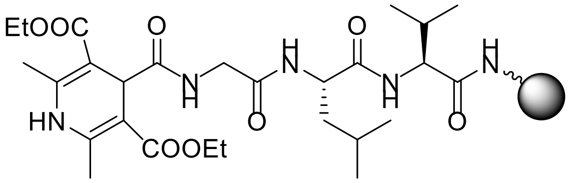

2m: DHP-Gly-(*L*)-Leu-(*L*)-Val-Rink Amide

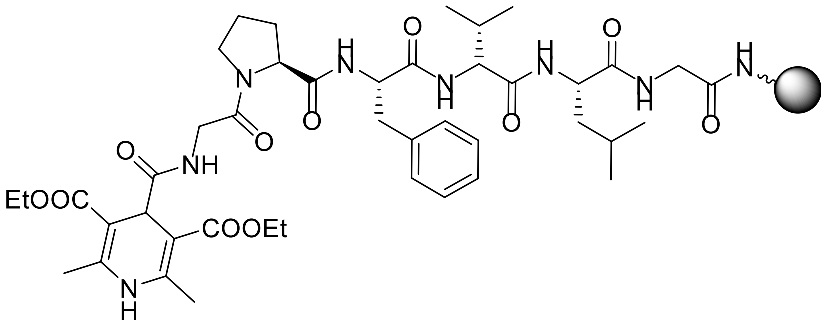

2n: DHP-Gly-(*L*)-Pro-(*L*)-Phe-(*L*)-Val-(*L*)-Leu-Gly-Rink Amide

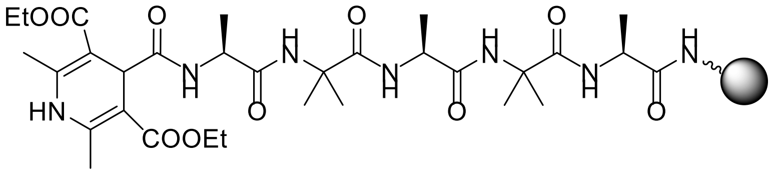

2o: DHP-(*L*)-Ala-Aib-(*L*)-Ala-Aib-(*L*)-Ala-Rink Amide

Figure S7. DHP-containing sequences synthesized using the SPPS approach.

4.2 GP6. Photocatalytic carbamoylation of imines (SPPS) to form products 4a-c/4'a-c and 6/6'

In the carbamoylation reaction using the SPPS approach, acid conditions (*i.e.* $BF_3 \cdot Et_2O$) were avoided to prevent side reactions. On the other hand, to enhance the reactions, each coupling was carried out using an excess of imine. The condition adopted for the carbamoylation of imines using DHP-containing peptides anchored to Rink amide resin are reported in Scheme S7.

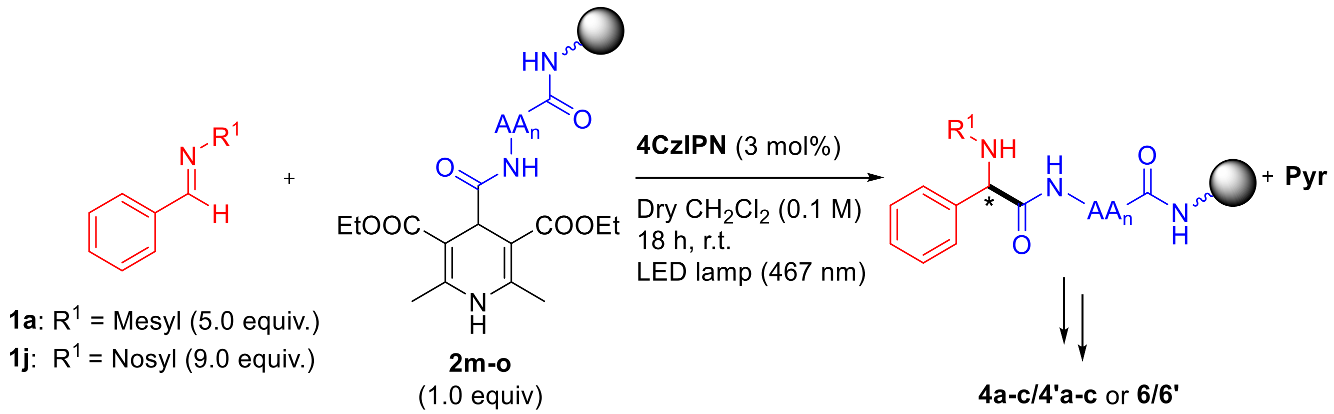

Scheme S7. Reaction conditions used for the carbamoylation of imines using the SPPS approach.

General Procedure 6. The DHP-containing peptide anchored to the resin (see *General Procedure 5*) was added in a 10 mL glass vial. With respect to the initial amount of Rink Amide resin, **4CzIPN** (2.60 mg, 0.0033 mmol, 0.03 equiv.) and either imine **1a** bearing Mesyl or **1j** featuring Nosyl group at nitrogen atom (5.0 or 9.0 equiv., respectively) were added. The vial was sealed with a cap with septum and it was evacuated and flushed with nitrogen three times. Then, dry CH_2Cl_2 (4.0 mL, 0.0275 M) were added. The reaction mixture was shacked for 20 minutes to allow the resin to swell, then the blue LED lamp was switched on (λ = 467 nm; distance between lamp and vial: ~5 cm; a fan was used to dissipate the heat generated by the lamp) and shacked for 18 h at r.t. The mixture was transferred in a SPPS reactor, the solvent and the excess of imine were filtered off, then the resin was washed with CH_2Cl_2 (3 x 5 mL) and finally dried using Et_2O. Following an ESI analysis of a minicleavage to confirm the presence of the desired product **4/4'** or **6/6'**, each peptide was cleaved from the resin using a TFA/TIS/H_2O mixture (95:2.5:2.5 ratio), then the peptide was precipitated using ice-cold Et_2O and centrifuged. The crude peptides were purified by semipreparative HPLC using H_2O/CH_3CN mixture and lyophilized.

Prior to cleave the peptide from the resin, in case of product **6/6'** the Nosyl residue was removed and an additional amino acid was inserted, following the steps depicted in Scheme S9 (see **Chapter 4.3** below for the synthetic details).

4.3 Removal of the -SO_2R moiety and sequence elongation to access product 6/6’

Following the reported procedures, we managed to remove either the *tert*-butylsulfonyl group under acid conditions[22] or the nosyl group in a basic environment[23] forming the same mixture of product **5**/**5’** (Scheme S8).

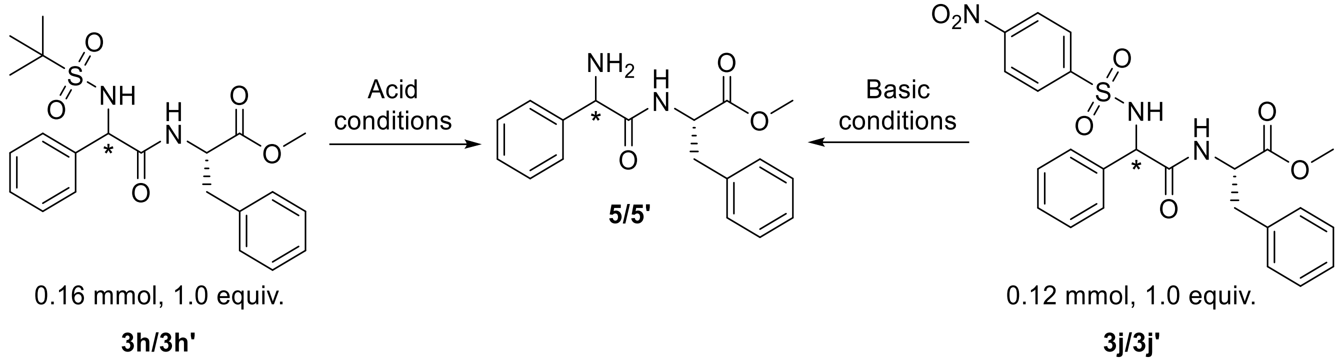

Scheme S8. Cleavage approaches of the sulfonamide moieties.

Acid conditions (liquid phase)[22]

Working in a round bottomed flask under inert atmosphere, the mixture of diastereoisomers **3h**/**3’h** (0.16 mmol, 1.0 equiv.) was dissolved in CH_2Cl_2 (0.15 M), then anisole (0.21 mmol, 1.3 equiv) and $AlCl_3$ (0.32 mmol, 2.0 equiv.) were added. The mixture was stirred at r.t. and the consumption oft he reagents was monitored by TLC (Hex/AcOEt 6:4). The reaction turned brown and after 4 h reached completion. The crude mixture was diluted with CH_2Cl_2 (5 ml) and carefully poured into a separating funnel containing $NaOH_{(aq)}$ (8 ml, 2 M). The aqueous layer was extracted using CH_2Cl_2 (5 x 5 ml). The combined organic layers were dried over Na_2SO_4, filtered and concentrated under reduced pressure. Flash chromatography (CH_2Cl_2/MeOH, 100:0→95:5) afforded methyl (2-amino-2-phenylacetyl)-*L*-phenylalaninate (**5**/**5’**) as light brown solid (45.3 mg, 91%) as a mixture of inseparable diastereoisomers.

Basic conditions (liquid phase)[23]

In a round bottomed flask, the mixture of diastereoisomers **3j**/**3’j** (0.12 mmol, 1.0 equiv.) was dissolved in DMF (0.65 M), then K_2CO_3 (0.60 mmol, 5.0 equiv.) and tiophenol (0.24 mmol, 2.0 equiv.) were added. The mixture was stirred at r.t. and the consumption of the reagents was monitored by TLC (Hex/AcOEt 6:4). Upon completion, the reaction was quenched with HCl and extracted with CH_2Cl_2 (6 x 5 ml). The combined organic layers were dried over Na_2SO_4, filtered and concentrated under reduced pressure. Flash chromatography (CH_2Cl_2/MeOH, 100:0→95:5) afforded methyl (2-amino-2-phenylacetyl)-*L*-phenylalaninate (**5**/**5’**) as light brown solid (32.6 mg, 87%) as a mixture of inseparable diastereoisomers.

Methyl (2-amino-2-phenylacetyl)-*L*-phenylalaninate (5/5’)

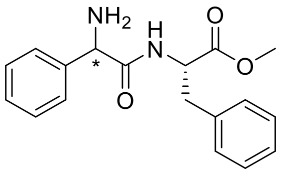

^{1}H NMR (400 MHz, $CDCl_3$) δ 7.67 (d, J = 8.2 Hz, 1H), 7.41-7.16 (m, 17H), 7.08-6.94 (m, 4H), 4.97-4.81 (m, 2H), 4.54 (s, 2H), 3.75 (s, 3H), 3.72 (s, 3H), 3.22-3.01 (m, 4H), 2.05 (br s, 4H); ^{13}C NMR (101 MHz, $CDCl_3$) δ 172.7, 172.5, 172.1, 171.9, 140.7, 140.5, 136.0, 135.9, 129.4 (x 2), 129.3 (x 2), 129.0 (x 2), 128.9 (x 2), 128.7 (x 4), 128.3, 128.1, 127.3, 127.2, 127.1 (x 2), 127.0 (x 2), 59.9, 59.8, 53.1, 52.9, 52.4, 52.4, 38.0, 37.9; MS (ESI+): *m/z* $[M+H]^+$ calcd. for $C_{18}H_{21}N_2O_3$: 313.15, found 313.05; $[2M+H]^+$ calcd. for $C_{36}H_{41}N_4O_6$: 625.30, found 625.26.

Using the conditions reported above, we attempted the removal of the methylsulfonyl group from the products **3a**/**3’a**. Unfortunately, the reaction did not work and the starting mixture of diastereoisomers **3a**/**3’a** was recovered.

As we synthesized some peptidomimetics with the SPPS approach, we decided to try and form the *in situ* non-natural amino acid of the sequence bearing the Nosyl group, which can be cleaved in basic environment (Scheme S9). We adapted the reaction

conditions from the procedure described above. In a vial 5 ml of previously prepared saturated solution of K_2CO_3 in DMF were poured in a vial (carefully, no suspended K_2CO_3 was placed in the vial), then PhSH (10.0 equiv. with respect of the initial amount of Rink Amide resin) was added. This solution was quantitatively transferred into the reactor with the swelled resin, and the reaction was run for 6h, after which time the solution was filtered off, freshly prepared cleavage cocktail was added and the reaction was run overnight. The resin was washed with DMF (10 x 5 ml), CH_2Cl_2 (5 x 5 ml) and Et_2O (3 x 5 ml). A cleavage of the peptide on a small amount of resin was carried out to verify the Nosyl removal, which was confirmed with ESI analysis. Finally, following the reported *General Procedure 5*, *N*-acetyl-*L*-alanine was coupled (x 2 times) and finally purified to achieve **6/6'** as a mixture of diastereoisomers.

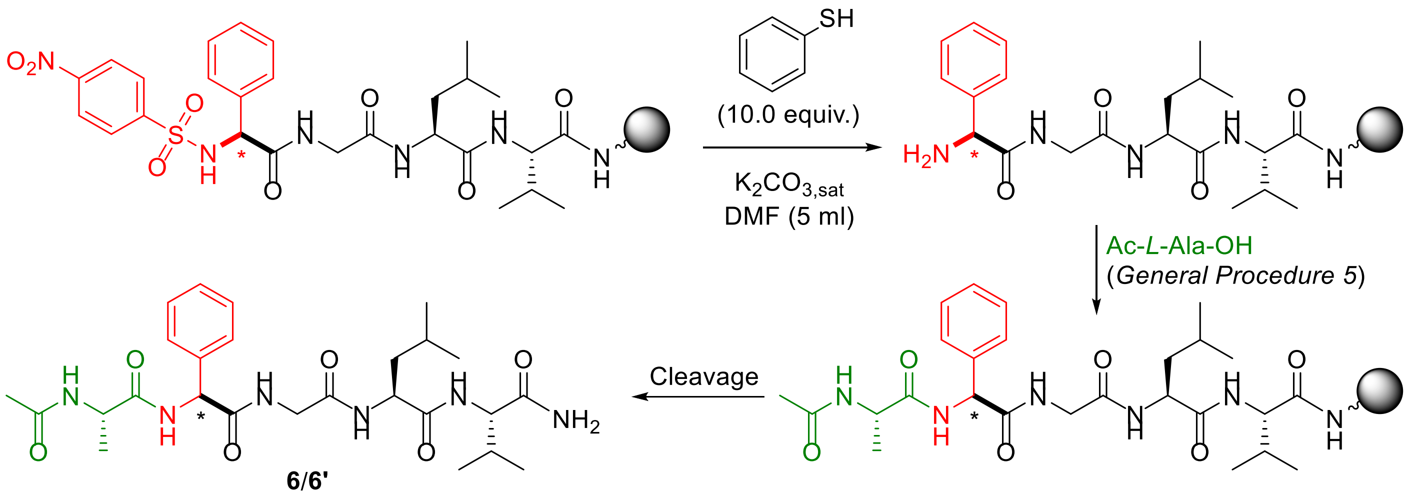

Scheme S9. Subsequent steps from the Nosyl-protected peptide anchored to Rink Amide resin to obtain product **6/6'**.

4.4 Reaction scope (SPPS)

The products formed using the SPPS approach are reported in Figure S8.

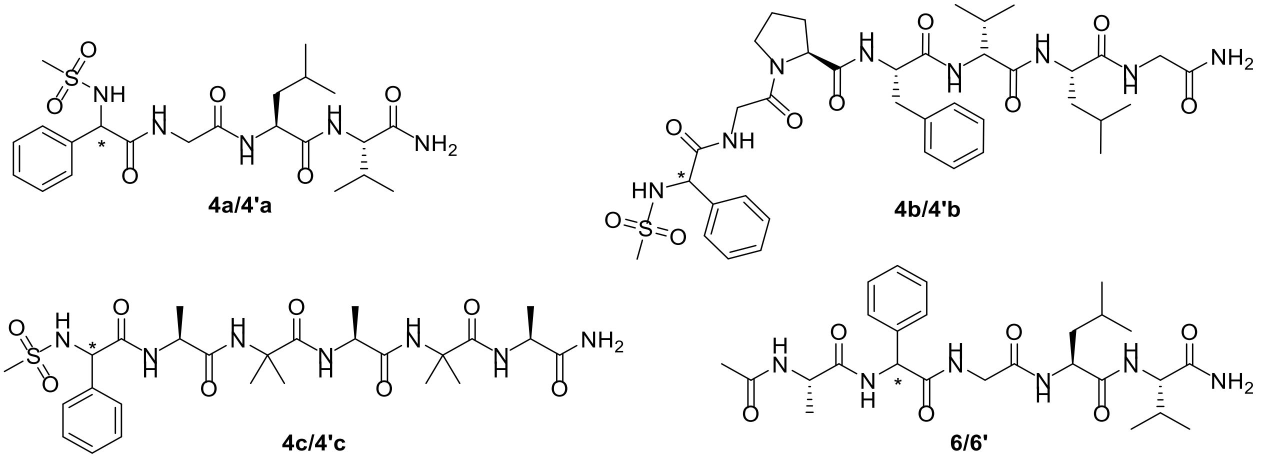

Figure S8. Scope of derivatized DHPs **2h-j** anchored to Rink Amide resin.

Mesyl-PhGly-Gly-(*L*)-Leu-(*L*)-Val-NH$_2$ (4a/4'a)

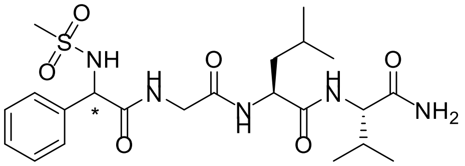

General Procedure 6. DHP-Gly-(*L*)-Leu-(*L*)-Val-Rink Amide resin **2m** (obtained from 250 mg of Fmoc-Rink Amide resin), imine **1a** (146.7 mg, 0.80 mmol, 5.0 equiv.), **4CzIPN** (3.8 mg, 0.005 mmol, 0.03 equiv.) in dry CH_2Cl_2 (4.0 ml). The crude peptide was purified with semipreparative HPLC using H_2O/CH_3CN mixture (gradient from 15 to 60% of CH_3CN in 20 minutes). The two diastereoisomers were collected together and lyophilized (white solid, 4.4 mg).

HRMS (ESI+): *m/z* $[M+Na]^+$ calcd. for $C_{22}H_{35}N_5NaO_6S$: 520.2206, found 520.2205. Analytical HPLC: gradient from 20 to 40% of CH_3CN in 30 minutes.

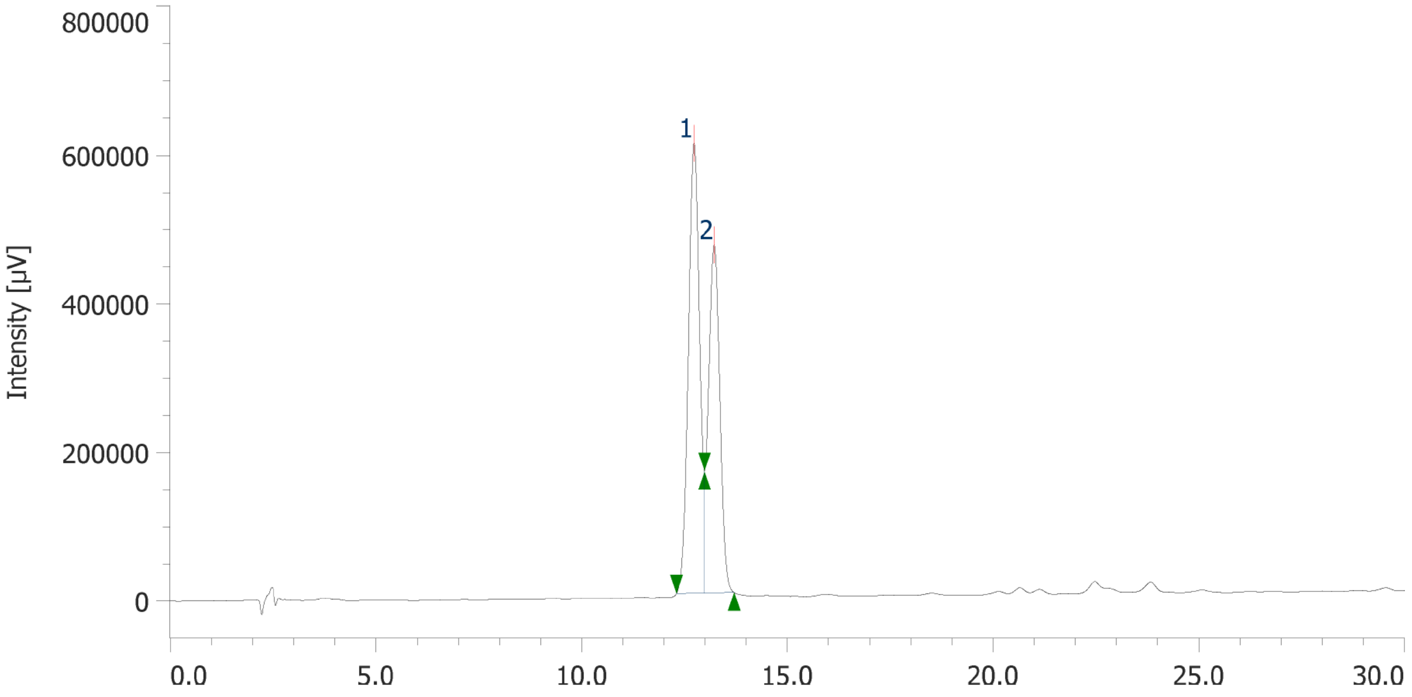

#	Peak Name	CH	tR [min]	Area [μV·sec]	Height [μV]	Area%	Height%	Resolution	Symmetry Factor	Factor
1	Unknown	5	12.7	11519947	605246	56.3	56.393	0.956	N/A	1.00000
2	Unknown	5	13.2	8942451	468015	43.7	43.607	N/A	N/A	1.00000
Total				20462398	1073261					

UPLC-MS: gradient from 20 to 40% of CH_3CN in 11 minutes.

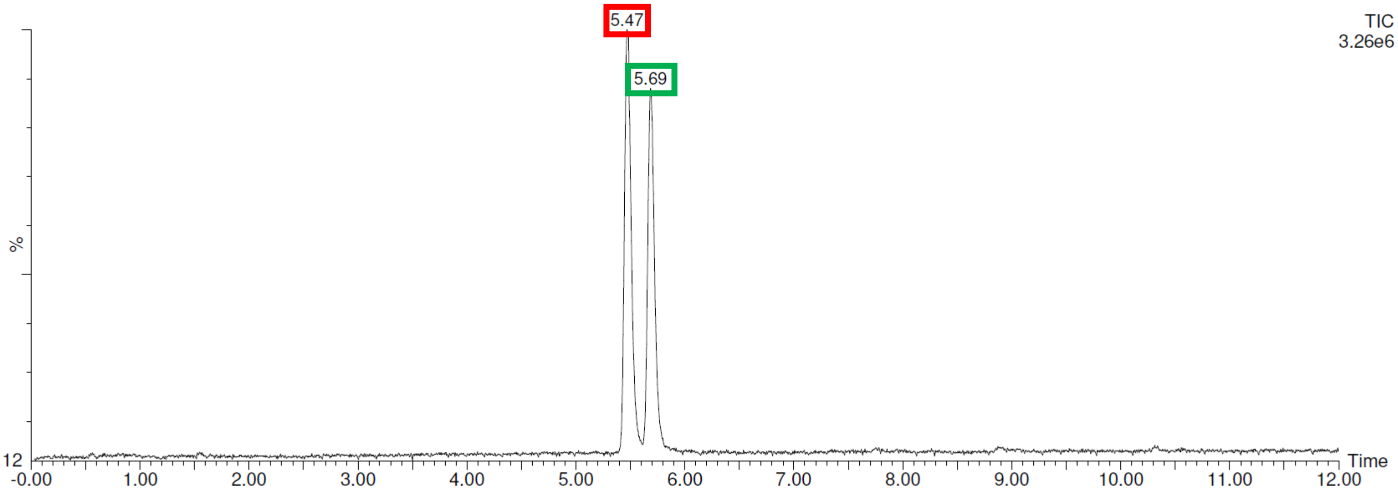

TG108_02 990 5.470 AM2 (Ar,40000.0,0.00,0.00); Cm (985:995)

1: TOF MS ES+
4.17e+006

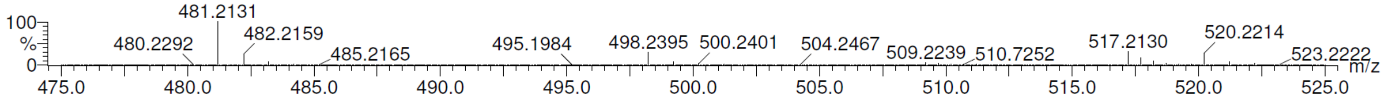

Minimum:				-5.0				
Maximum:		20.0	5.0	300.0				
Mass	Calc. Mass	mDa	PPM	DBE	i-FIT	Norm	Conf(%)	Formula
498.2395	498.2386	0.9	1.8	7.5	1519.6	n/a	n/a	C22 H36 N5 O6 S

TG108_02 1028 5.685 AM2 (Ar,40000.0,0.00,0.00); Cm (1023:1035)

1: TOF MS ES+
3.89e+006

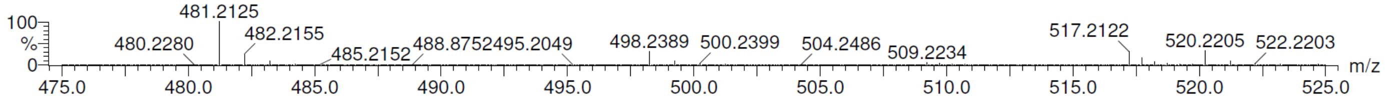

Minimum:				-5.0				
Maximum:		20.0	5.0	300.0				
Mass	Calc. Mass	mDa	PPM	DBE	i-FIT	Norm	Conf(%)	Formula
498.2389	498.2386	0.3	0.6	7.5	1471.6	n/a	n/a	C22 H36 N5 O6 S

Mesyl-PhGly-Gly-(*L*)-Pro-(*L*)-Phe-(*L*)-Val-(*L*)-Leu-Gly-NH$_2$ (4b/4'b)

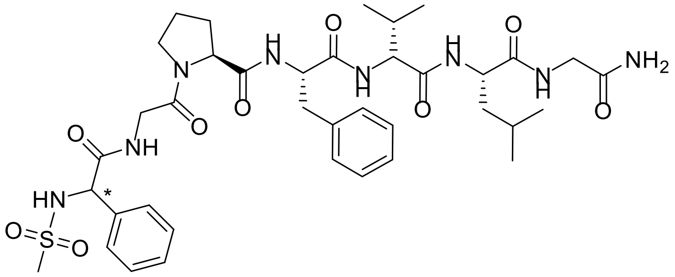

General Procedure 6. DHP-Gly-(*L*)-Pro-(*L*)-Phe-(*L*)-Val-(*L*)-Leu-Gly-Rink Amide resin **2n** (obtained from 250 mg of Fmoc-Rink Amide resin), imine **1a** (100.8 mg, 0.55 mmol, 5.0 equiv.), **4CzIPN** (2.6 mg, 0.0030 mmol, 0.03 equiv.) in dry CH_2Cl_2 (4.0 ml). The crude peptide was purified with semipreparative HPLC using H_2O/CH_3CN mixture (gradient from 15 to 60% of CH_3CN in 20 minutes). The two diastereoisomers were collected together and lyophilized (white solid, 6.3 mg).

HRMS (ESI+): *m/z* $[M+Na]^+$ calcd. for $C_{38}H_{54}N_8NaO_9S$: 821.3632, found 821.3629. Analytical HPLC: gradient from 20 to 40% of CH_3CN in 30 minutes.

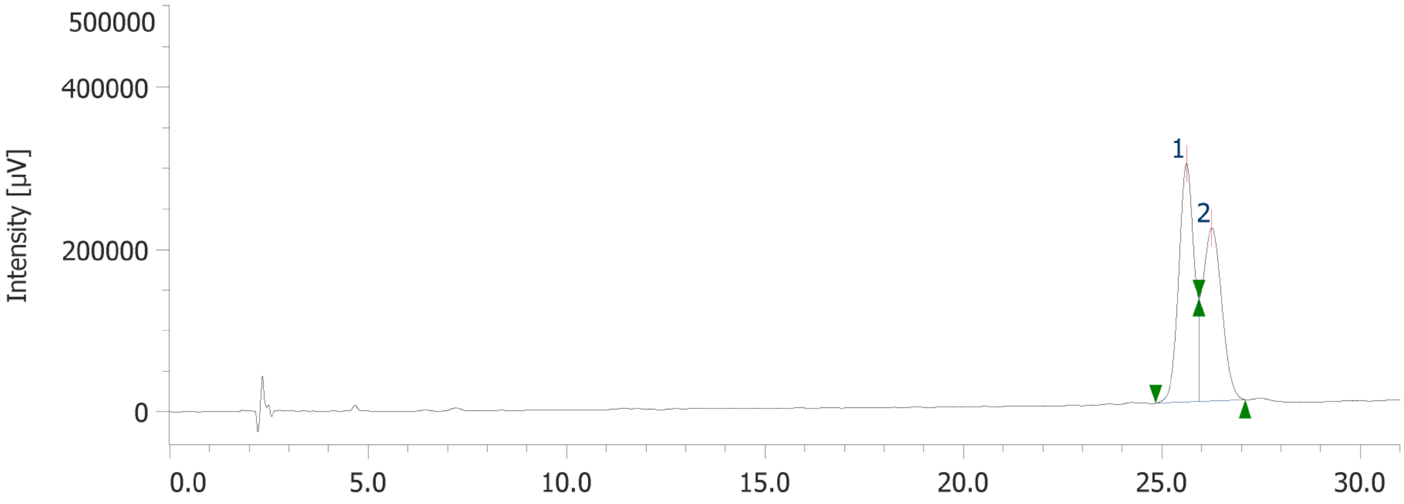

#	Peak Name	CH	tR [min]	Area [µV·sec]	Height [µV]	Area%	Height%	Resolution	Symmetry Factor	Factor
1	Unknown	5	25.6	7817385	293790	52.0	57.929	N/A	N/A	1.00000
2	Unknown	5	26.3	7216835	213365	48.0	42.071	N/A	N/A	1.00000
Total				15034220	507155					

UPLC-MS: gradient from 20 to 40% of CH_3CN in 11 minutes.

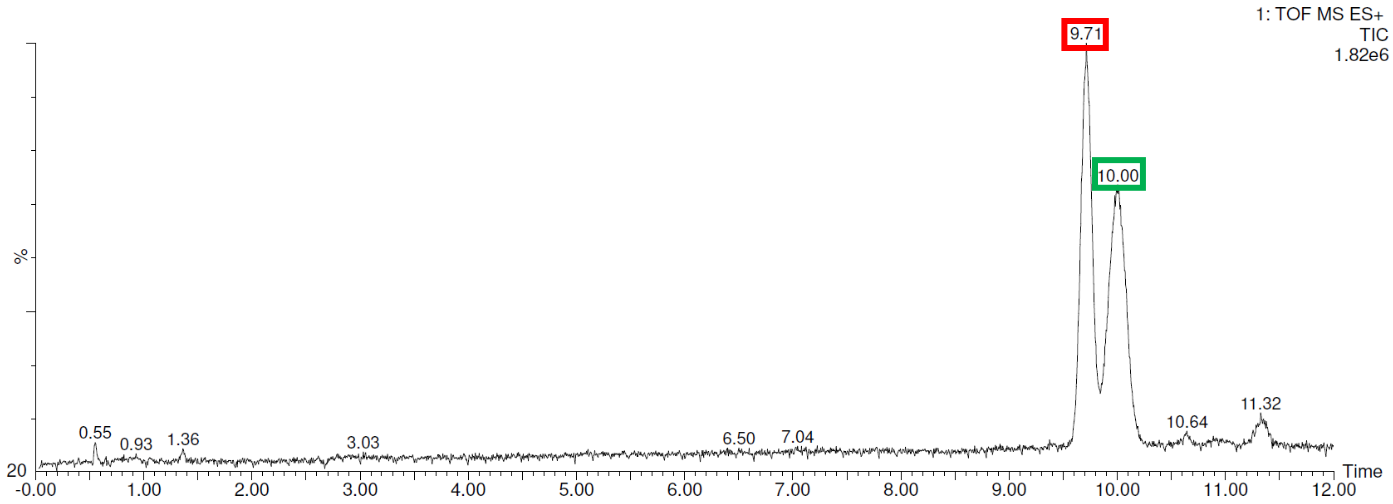

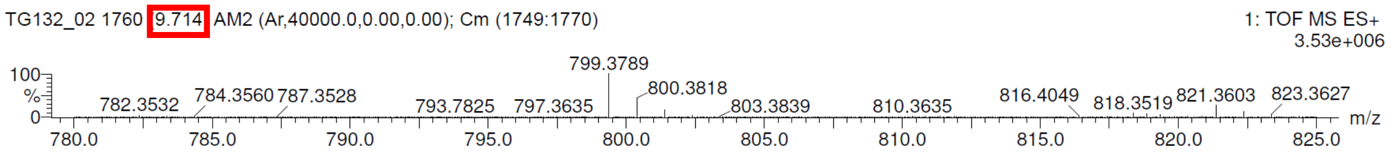

Minimum:				-5.0				
Maximum:		20.0	5.0	300.0				
Mass	Calc. Mass	mDa	PPM	DBE	i-FIT	Norm	Conf(%)	Formula
799.3789	799.3813	-2.4	-3.0	15.5	1120.2	n/a	n/a	C38 H55 N8 O9 S

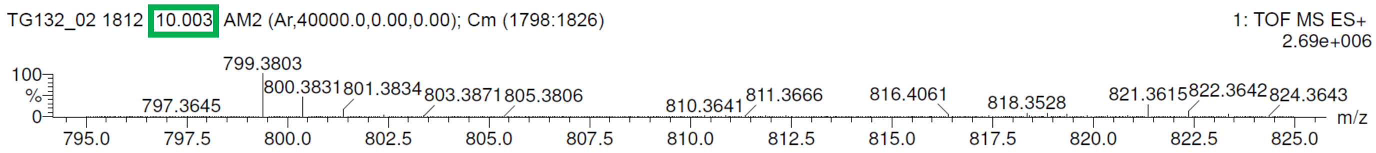

Minimum:				-5.0				
Maximum:		20.0	5.0	300.0				
Mass	Calc. Mass	mDa	PPM	DBE	i-FIT	Norm	Conf(%)	Formula
799.3803	799.3813	-1.0	-1.3	15.5	1029.0	n/a	n/a	C38 H55 N8 O9 S

Mesyl-PhGly-(*L*)-Ala-Aib-(*L*)-Ala-Aib-(*L*)-Ala-NH_2 (4c/4'c)

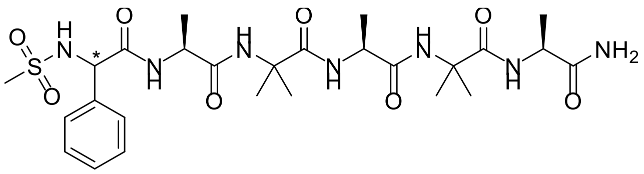

General Procedure 6. DHP-(*L*)-Ala-Aib-(*L*)-Ala-Aib-(*L*)-Ala-Rink Amide resin **2o** (obtained from 150 mg of Fmoc-Rink Amide resin), imine **1a** (77.9 mg, 0.43 mmol, 5.0 equiv.), **4CzIPN** (2.0 mg, 0.0026 mmol, 0.03 equiv.) in dry CH_2Cl_2 (4.0 ml). The crude peptide was purified with semipreparative HPLC using H_2O/CH_3CN mixture (gradient from 10 to 70% of CH_3CN in 20 minutes). The two diastereoisomers were collected together and lyophilized (yellow solid, 4.0 mg).

HRMS (ESI+): *m/z* $[M+Na]^+$ calcd. for $C_{26}H_{41}N_7NaO_8S$: 634.2635, found 634.2631. Analytical HPLC: gradient from 10 to 60% of CH_3CN in 20 minutes.

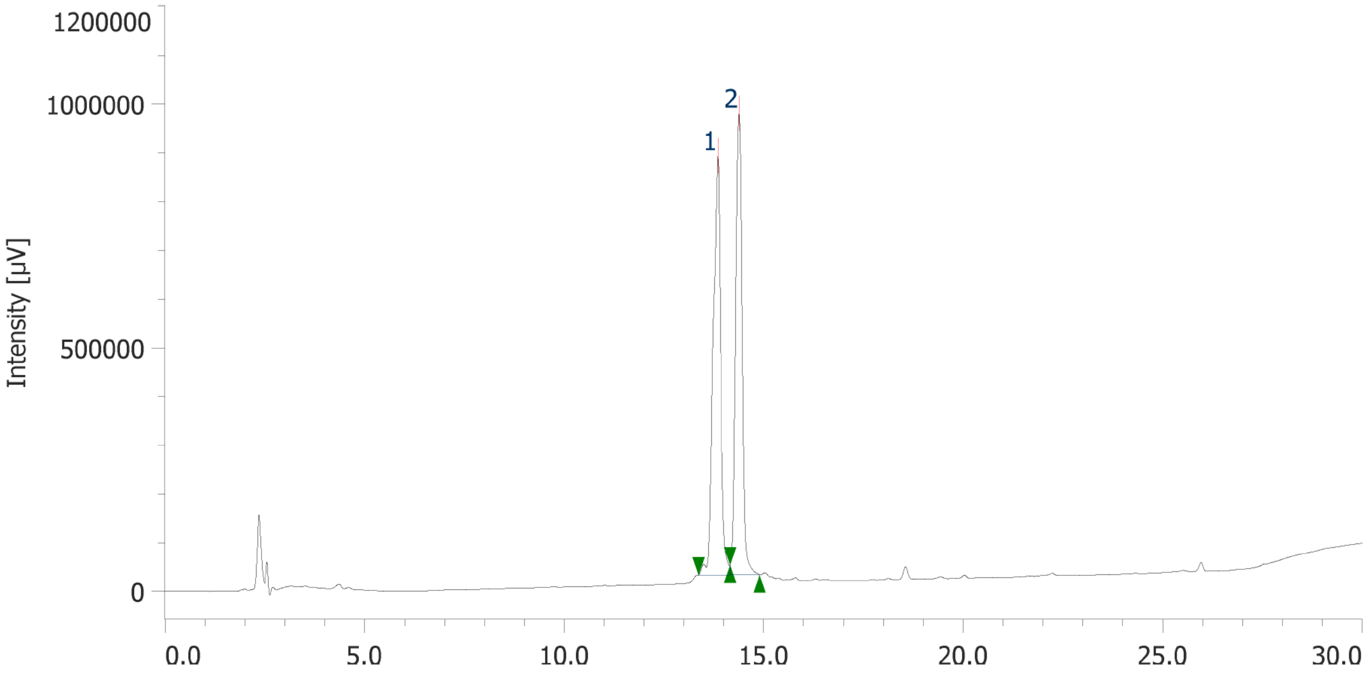

#	Peak Name	CH	tR [min]	Area [μV·sec]	Height [μV]	Area%	Height%	Resolution	Symmetry Factor	Factor
1	Unknown	5	13.8	11390505	859371	51.4	47.591	1.612	0.920	1.00000
2	Unknown	5	14.4	10764663	946379	48.6	52.409	N/A	1.003	1.00000
Total				22155168	1805750					

UPLC-MS: gradient from 10 to 60% of CH_3CN in 7.5 minutes.

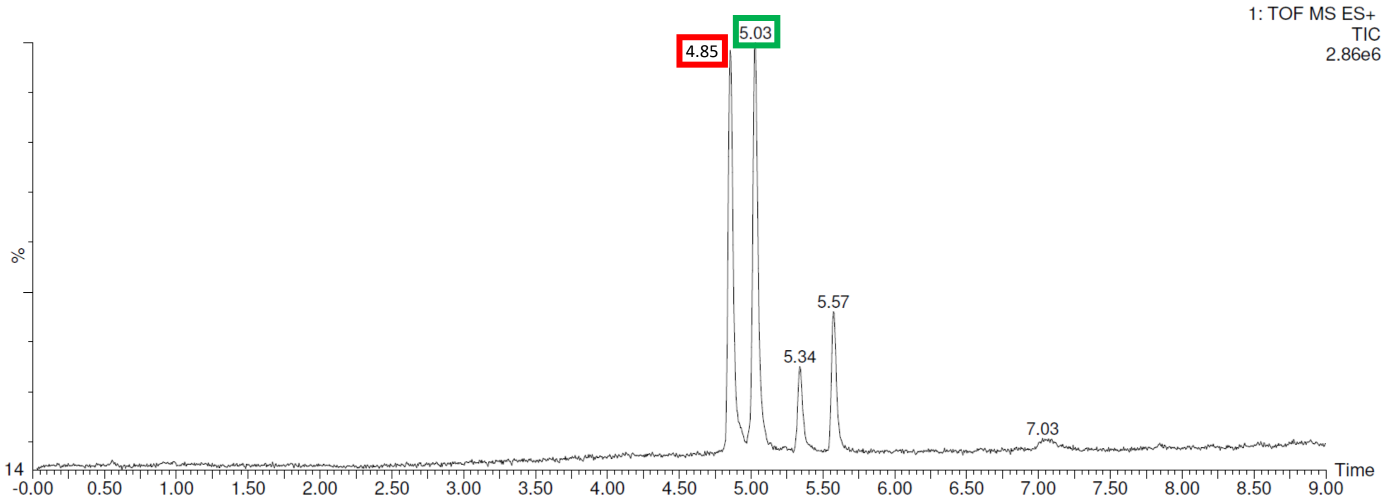

TG142_02 877 4.854 AM2 (Ar,40000.0,0.00,0.00); Cm (874:881)　　1: TOF MS ES+ 2.63e+006

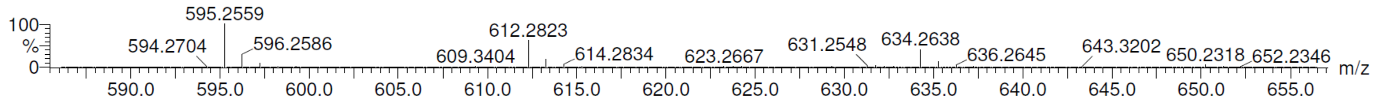


```
Minimum:                                  -5.0
Maximum:                    20.0    5.0   300.0

Mass        Calc. Mass  mDa     PPM     DBE     i-FIT   Norm    Conf(%) Formula

612.2823    612.2816    0.7     1.1     9.5     1182.2  n/a     n/a     C26 H42 N7 O8 S
```

TG142_02 909 5.029 AM2 (Ar,40000.0,0.00,0.00); Cm (905:912)　　1: TOF MS ES+ 2.34e+006

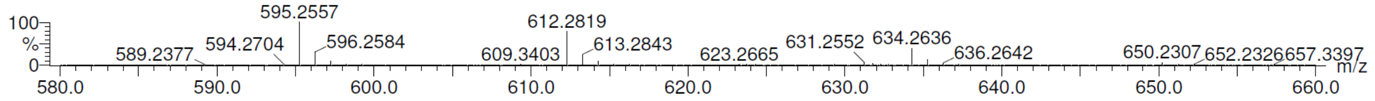


```
Minimum:                                  -5.0
Maximum:                    20.0    5.0   300.0

Mass        Calc. Mass  mDa     PPM     DBE     i-FIT   Norm    Conf(%) Formula

612.2819    612.2816    0.3     0.5     9.5     1281.1  n/a     n/a     C26 H42 N7 O8 S
```

Ac-(*L*)-Ala-PhGly-Gly-(*L*)-Leu-(*L*)-Val-NH$_2$ (6/6')

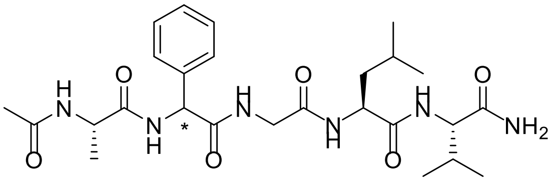

General Procedure 6. DHP-Gly-(*L*)-Leu-(*L*)-Val-Rink Amide resin **2m** (obtained from 250 mg of Fmoc-Rink Amide resin), imine **1j** (365.7 mg, 1.26 mmol, 9.0 equiv.), **4CzIPN** (3.3 mg, 0.0041 mmol, 0.03 equiv.) in dry CH_2Cl_2 (4.0 ml). The nosyl residue was removed to obtain the free amino group at the *N*-terminus, adapting the procedure reported by Hayashi for the SPPS approach (see **Chapter 4.3** for synthetic details). To confirm the formation of the desired product, a minicleavage was carried out together with a ESI analysis. The last coupling was carried out following the *General Procedure 5* using H_2N-PhGly-Gly-(*L*)-Leu-(*L*)-Val-Rink Amide resin and *N*-acetyl-(*L*)-alanine (92.1 mg, 0.69 mmol, 5.0 equiv.). The crude peptide was purified with semipreparative HPLC using H_2O/CH_3CN mixture (gradient from 20 to 60% of CH_3CN in 20 minutes). The two diastereoisomers were collected together and lyophilized (white solid, 4.2 mg).

HRMS (ESI+): *m/z* $[M+Na]^+$ calcd. for $C_{26}H_{40}N_6NaO_6$: 555.2907, found 555.2905. Analytical HPLC: gradient from 20 to 60% of CH_3CN in 20 minutes.

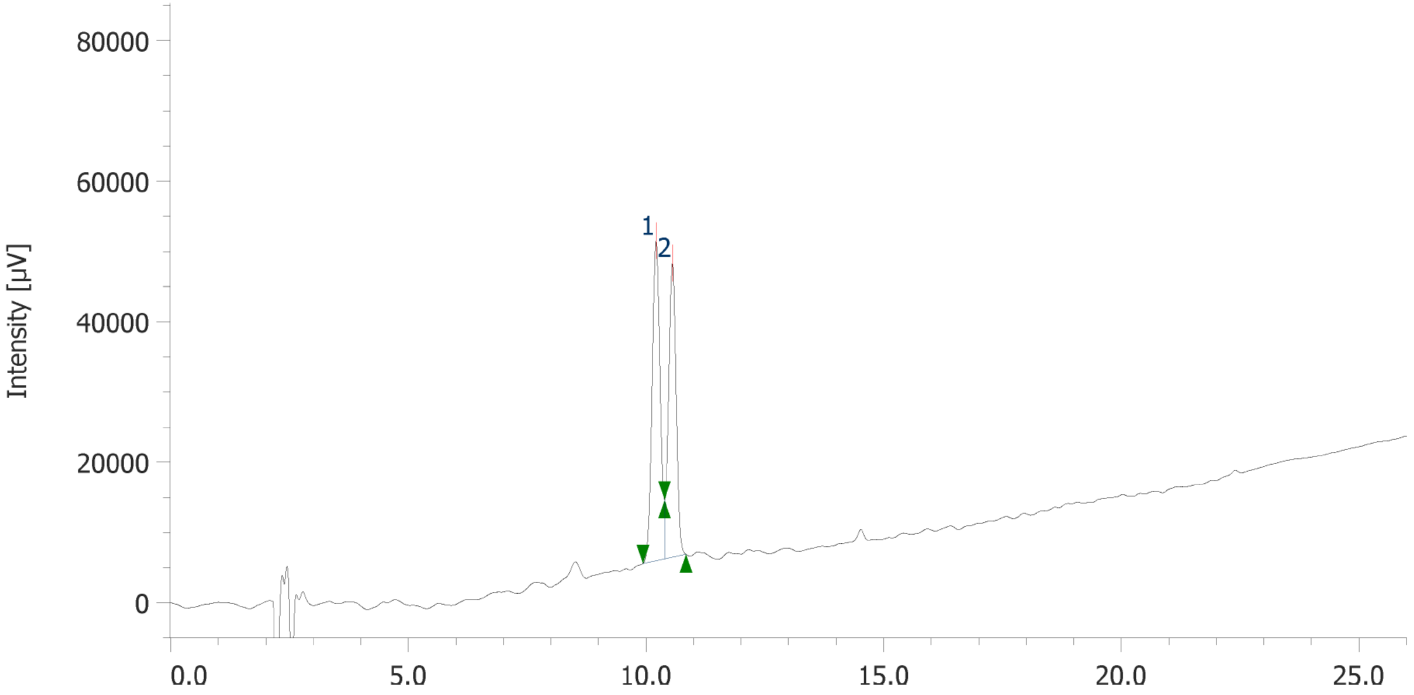

#	Peak Name	CH	tR [min]	Area [µV·sec]	Height [µV]	Area%	Height%	Resolution	Symmetry Factor	Factor
1	Unknown	5	10.2	528666	45516	52.9	52.117	1.164	N/A	1.00000
2	Unknown	5	10.6	470430	41819	47.1	47.883	N/A	N/A	1.00000
Total				999096	87335					

UPLC-MS: gradient from 20 to 60% of CH_3CN in 7.5 minutes.

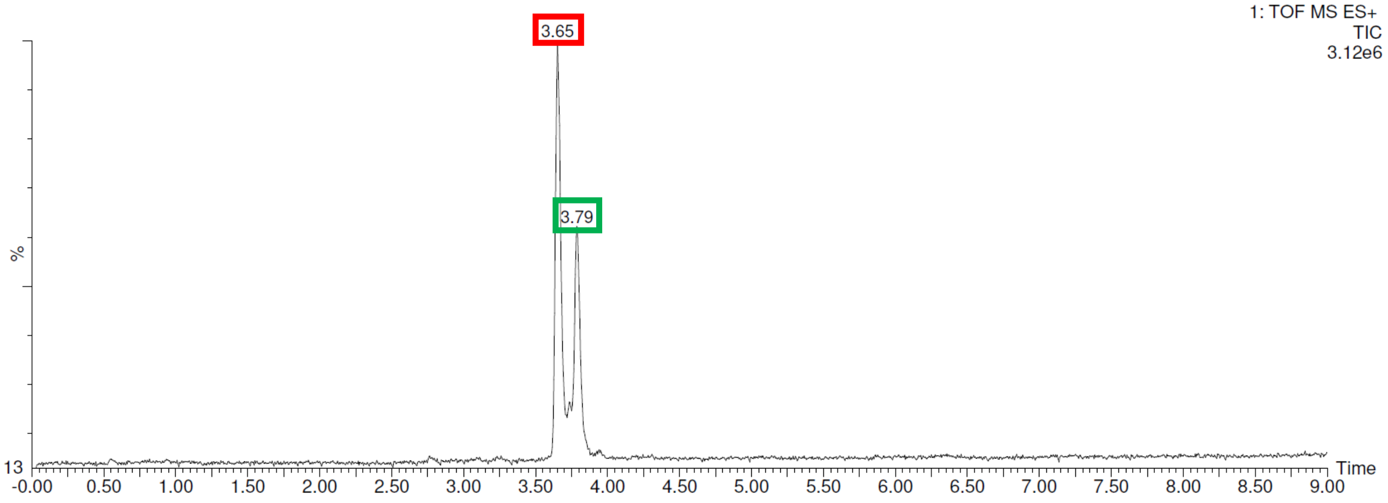

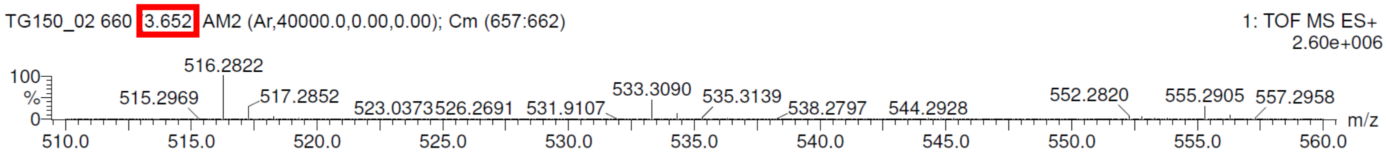

		mDa	PPM	DBE				
Minimum:				-5.0				
Maximum:		20.0	5.0	300.0				
Mass	Calc. Mass	mDa	PPM	DBE	i-FIT	Norm	Conf(%)	Formula
533.3090	533.3088	0.2	0.4	9.5	1364.4	n/a	n/a	C26 H41 N6 O6

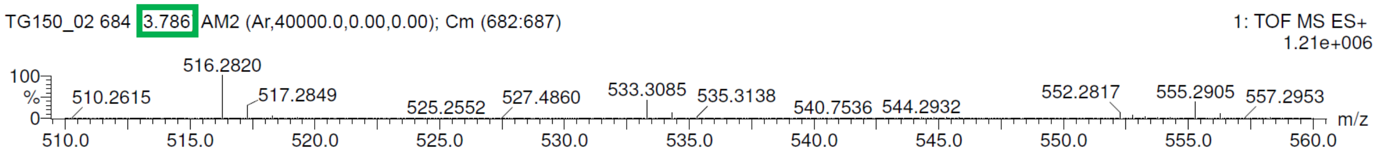

		mDa	PPM	DBE				
Minimum:				-5.0				
Maximum:		20.0	5.0	300.0				
Mass	Calc. Mass	mDa	PPM	DBE	i-FIT	Norm	Conf(%)	Formula
533.3085	533.3088	-0.3	-0.6	9.5	1302.0	n/a	n/a	C26 H41 N6 O6

5. Mechanicistic investigations

5.1 Stern-Volmer analysis

A 0.01 mM stock solution of photocatalyst in acetonitrile was used to prepare a 0.01 M solution of the quencher. Aliquots of this solution were added to a 5 mL volumetric flask and diluted with the same (0.01 mM) photocatalyst solution. All solutions were degassed with nitrogen for 15 minutes before measuring time-resolved luminescence. The lifetimes of the delayed fluorescence obtained from the fit of the exponential decay were plotted against the quencher concentration according to the Stern-Volmer equation:

$$\frac{\tau_0}{\tau} = 1 + K_{SV}[Q]$$

where τ_0 and τ are the lifetimes of delayed fluorescence in the absence and in the presence of quencher Q, and K_{SV} is the Stern-Volmer quenching constant, which was calculated from the slope of the linear fit against the quencher concentration $[Q]$. The quenching rate constant k_q is obtained from the following relationship:

$$k_q = \frac{K_{SV}}{\tau_0}$$

Following, the Stern-Volmer plot is presented (Figure S9) and the calculated quenching rate constant are reported in Table S2.

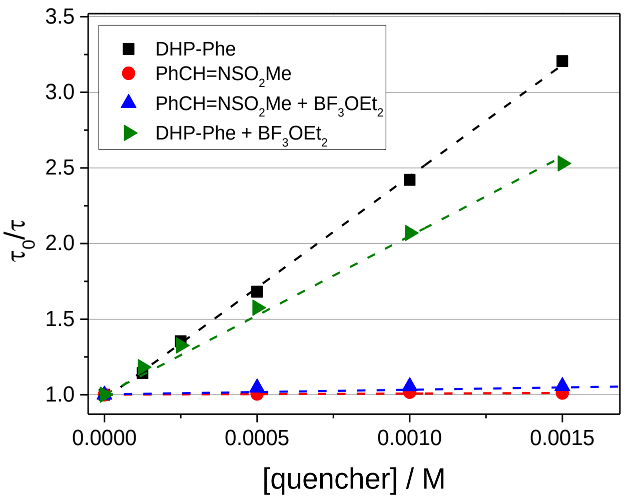

Figure S9. Stern-Volmer plot of delayed lifetimes (excitation at 375 nm, emission at 560 nm) of 0.01 mM **4CzIPN** in nitrogen-degassed solution in CH_3CN in the presence of DHP-(*L*)-Phe **2a** or *N*-benzylidenemethanesulfonamide **1a**, with or without $BF_3 \cdot OEt_2$.

Table S2. Quenching rate constants (k_q) of **4CzIPN** calculated from the Stern-Volmer plot.

Quencher (**Q**)	k_q (s^{-1} M^{-1})	
	without $BF_3 \cdot Et_2O$	with $BF_3 \cdot Et_2O$
DHP-(*L*)-Phe (**2a**)	8.37×10^8	5.30×10^8
N-benzylidenemethanesulfonamide (**1a**)	4.20×10^6	1.55×10^7

5.2 Control experiments

- Reaction of imine **1a** in the presence of Hantzsch's ester (**HE**) instead of derivatized DHP (Scheme S10).

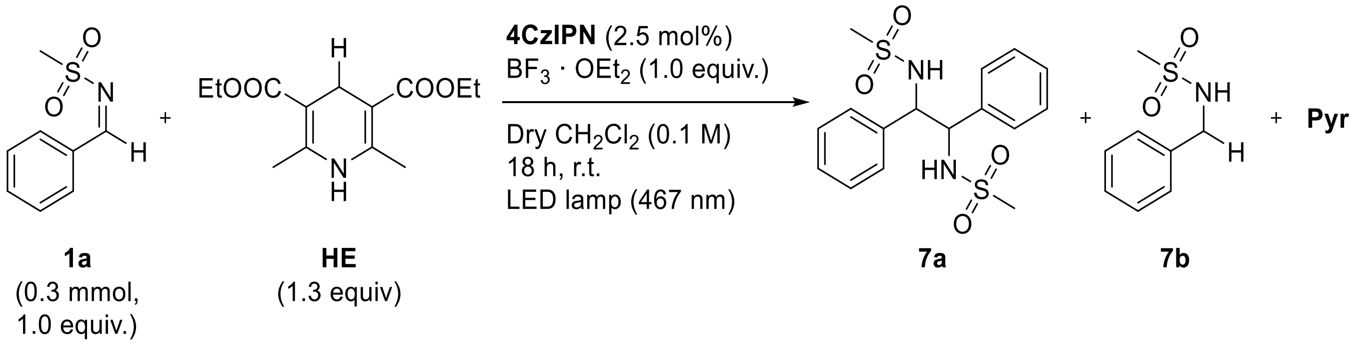

Scheme S10. Reaction between imine **1a** and **HE** with the formation of the pinacol like product **7a** and the reduced one **7b**.

This reaction was carried out using the optimized conditions to gain more information about the reaction mechanism. After column chromatography we isolated product **7a**, derived by the pinacol-like coupling of the starting imine **1a**, and product **7b**, which derived from the reduction of imine **1a**, as expected being **HE** an efficient reducing agent.

The ^{1}H NMR of product **7a** is in agreement with the literature.[9]

^{1}H NMR (400 MHz, $CDCl_3$) δ 7.25-7.18 (m, 6H), 7.14-7.03 (m, 4H), 6.11 (dd, *J* = 4.8, 2.3 Hz, 2H), 4.71 (dd, *J* = 4.8, 2.3 Hz, 2H), 2.60 (s, 6H).

The ^{1}H NMR of product **7b** is in agreement with the literature.[24]

^{1}H NMR (300 MHz, $CDCl_3$) δ 7.42-7.30 (m, 5H), 4.64 (s, 1H), 4.33 (d, *J* = 6.1 Hz, 2H), 2.88 (s, 3H).

- Reaction of imine **1a** in the absence of derivatized DHP **2a** (Scheme S11).

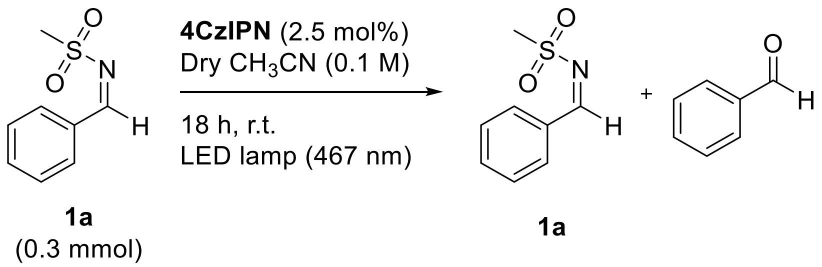

Scheme S11. Optimized reaction conditions of imine **1a** without functionalized DHP.

After column chromatography the starting imine **1a** was partially recovered, together with the photocatalyst **4CzIPN** and benzaldehyde derived from degradation of **1a**. No formation of the pinacol product **5b** was observed.

➢ Reaction of DHP-Phe **2a** in the absence of sulfonyl imine **1a** (Scheme S12).

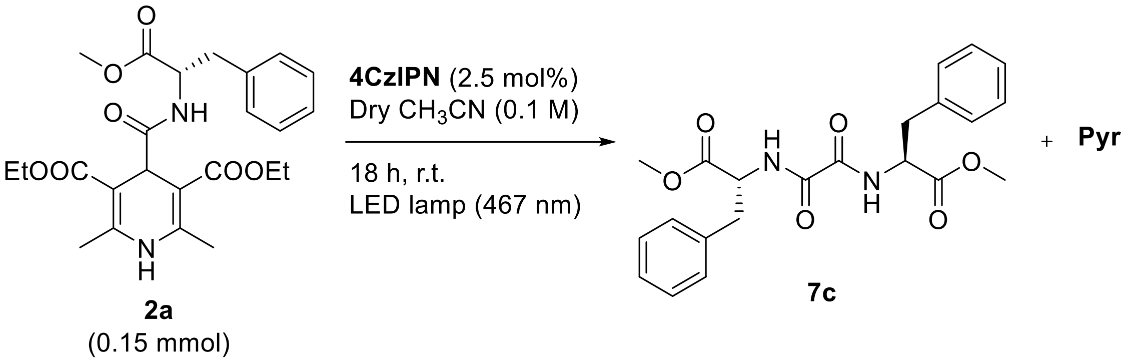

Scheme S12. Optimized reaction conditions without imine.

A reaction was run in the presence only of functionalized DHP-Phe **2a** and **4CzIPN**. The formation of by-product **7c** was observed by mass analysis of the crude product. MS (ESI+): *m/z* $[M+H]^+$ calcd. for $C_{22}H_{25}N_2O_6$: 413.16, found 413.04.

➢ Reactions between imine **1** and DHP **2** in the absence of the photocatalyst **4CzIPN**.

A reaction between imine **1a** and DHP-Phe **2a** was run in the absence of **4CzIPN** and the products **3a**/**3'a** were formed as a mixture of diastereoisomers in 56% yield (78% in case of using **4CzIPN**) (Scheme S13).

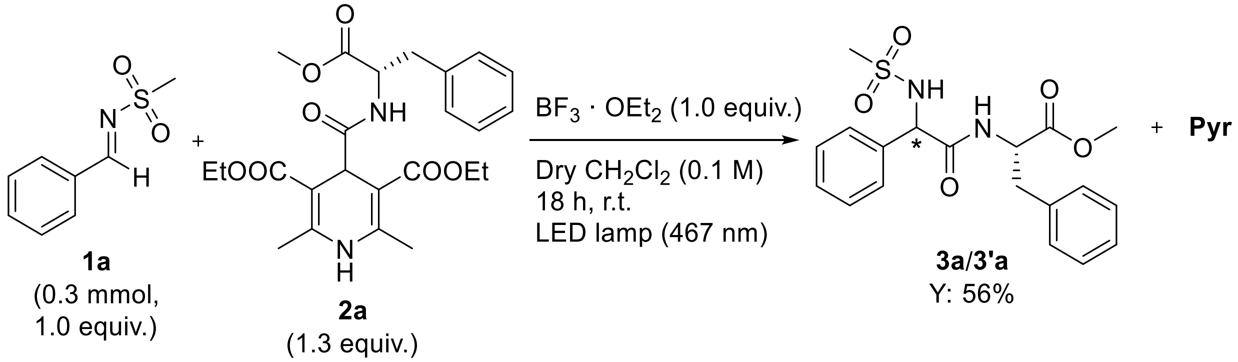

Scheme S13. Test without photocatalyst **4CzIPN**.

We also tested two poorly reactive substrates (i.e. imines **1e**,**f** and DHP-Met **2d**) without the photocatalyst in order to ascertain the efficacy of our methodology:

- Replacing imine **1a** with imine **1e**, bearing the methoxy group in the *para* position: products **3e**/**3'e** were formed in 24% yield (45% in the presence of **4CzIPN**);
- Replacing imine **1a** with imine **1f**, bearing the tiophene moiety: products **3f**/**3'f** were not formed;
- Replacing DHP-Phe **2a** with DHP-Met **2d**: products **3p**/**3'p** were obtained in 28% yield (61% by using **4CzIPN**).

➢ Reaction performed under non-inert atmosphere.

A reaction between imine **1a** and DHP-Phe **2a**, using the optimized condition, was performed under air instead of the inert atmosphere and the mixture of products **3a**/**3'a** was formed in 72% yield (Scheme S14).

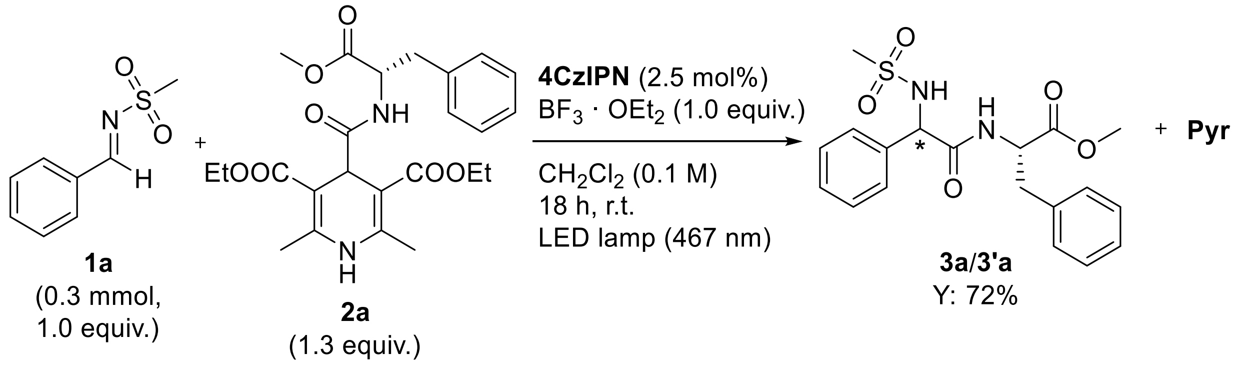

Scheme S14. Reaction between **1a** and **2a** run under non-inert conditions.

➢ Reaction performed in the dark.

The reaction between imine **1a** and DHP-Phe **2a** was performed using the optimized reaction conditions without the LED lamp (467 nm) and no product was detected either from NMR or MS analyses of the crude mixture.

➢ Reaction using HPK 125W high pressure mercury lamp (near UV range) without **4CzIPN**.

A test was performed between imine **1a** and DHP-Phe **2a** replacing the LED lamp (467 nm) with an HPK 125W high pressure Hg lamp and removing the photocatalyst **4CzIPN** (Scheme S15). The mixture of products **3a**/**3'a** was formed in 72% yield (78% by using the LED lamp together with **4CzIPN**).

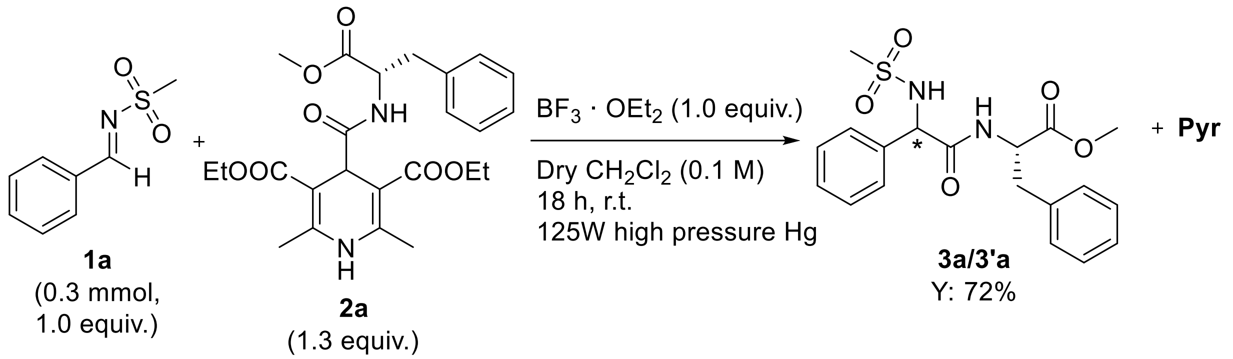

Scheme S15. Reaction performed using UV lamp instead of photocatalyst **4CzIPN** and visible light (467 nm).

- Stability test of DHP-Phe **2a** and imine **1a** under visible irradiation and different conditions.

Three different tests were performed:

1. In a sealed NMR tube, DHP-Phe **2a** (9.7 mg) was dissolved in CD_3CN. No changes in the NMR spectra were observed before and after irradiating the sample for 18 hours (Scheme S16);

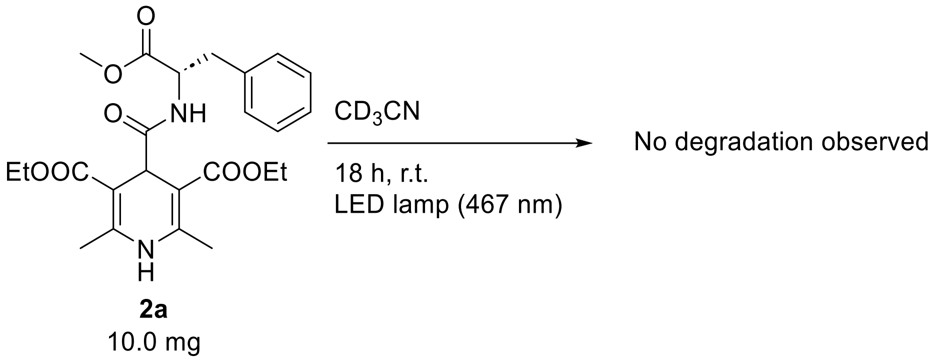

Scheme S16. Stability test of DHP-Phe **2a** under visible light irradiation (467 nm).

2. In a sealed NMR tube, imine **1a** (11.8 mg) was dissolved CD_3CN and $BF_3{\cdot}OEt_2$ (2.0 µl) was added. No changes in the NMR spectra were observed before and after irradiating the sample for 18 hours;
3. In a sealed NMR tube, DHP-Phe **2a** (10.1 mg) was dissolved CD_3CN and $BF_3{\cdot}OEt_2$ (2.0 µl) was added. An NMR spectrum was taken before and after irradiating the sample for 18 hours. It was detected the formation of pyridine (**Pyr**) byproduct.

5.3 Proposed catalytic cycle

Based on the reported literature exploiting the Hantzsch ester derivatives,[3,19,25] and the results obtained from both Stern-Volmer and control experiments (see **Chapter 5** and **6**), we propose the mechanism presented in Scheme S17. From Stern-Volmer quenching experiments, it is clear that the photoexcited photocatalyst (**4CzIPN***) is quenched by the derivatized DHP **2**, both in the presence and in the absence of the Lewis acid additive (Figure S9). Hence, for both proposed catalytic cycles, the first step is the reductive quenching of the photocatalyst to give its radical anion form **4CzIPN**$^{\bullet-}$, the well-known oxidized species **[Pyr]**$^+$ and the carbamoyl radical **I**. As a result, two different scenarios are plausible:

A. Radical addition to imine

In the presence of the Lewis acid additive, the carbamoyl radical **I** reacts with the imine **1** to form the adduct **IIa** generating a new C-C bond. Then, **IIa** interacts with the photocatalyst in its radical anion form (**4CzIPN**$^{\bullet-}$) to generate the desired product as a mixture of diastereoisomers **3/3'**, restoring the photocatalyst in its ground state. This catalytic cycle is corroborated by the control experiment presented in Scheme S12, where the pinacol-like coupling between radical **I** was detected by MS analysis.

B. Radical-radical coupling

As the redox potentials vs SCE of **4CzIPN** [*E* (**4CzIPN**/**4CzIPN**$^{\bullet-}$) = -1.24 V][2] and N-benzylidenemethanesulfonamide (**1a**) [*E* = -1.45 V][26] are similar, a SET between the radical anion **4CzIPN**$^{\bullet-}$ and imine **1** to form the species **IIb** can occur. Then, a radical-radical coupling between **I** and **IIb** is plausible, thus forming the desired product as a mixture of diastereoisomers **3/3'**. Thanks to the control experiment presented in Scheme S10 where the pinacol-like coupling of the imine **1** was observed, we can affirm that this catalytic cycle can not be excluded.

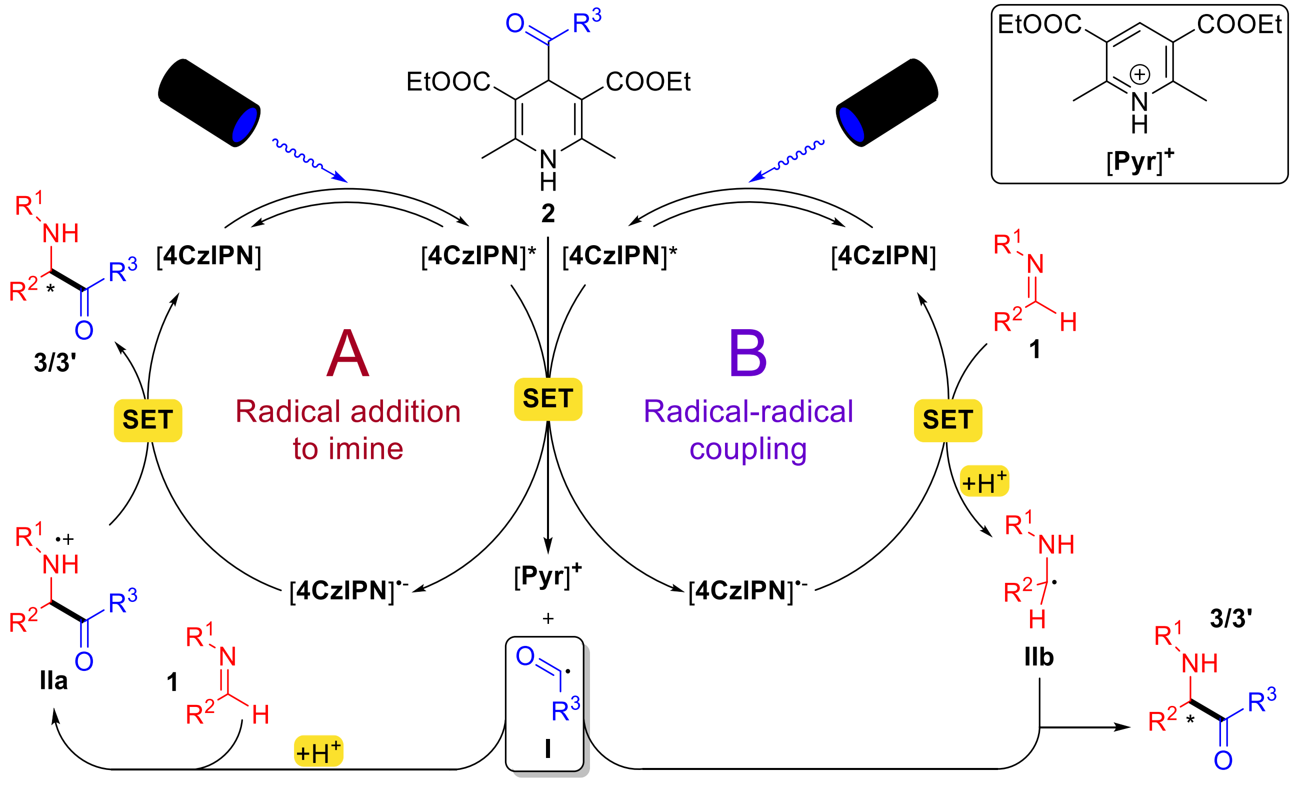

Scheme S17. Proposed catalytic cycles.

6. NMR Spectra of the Isolated Products

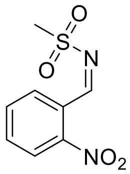

1d

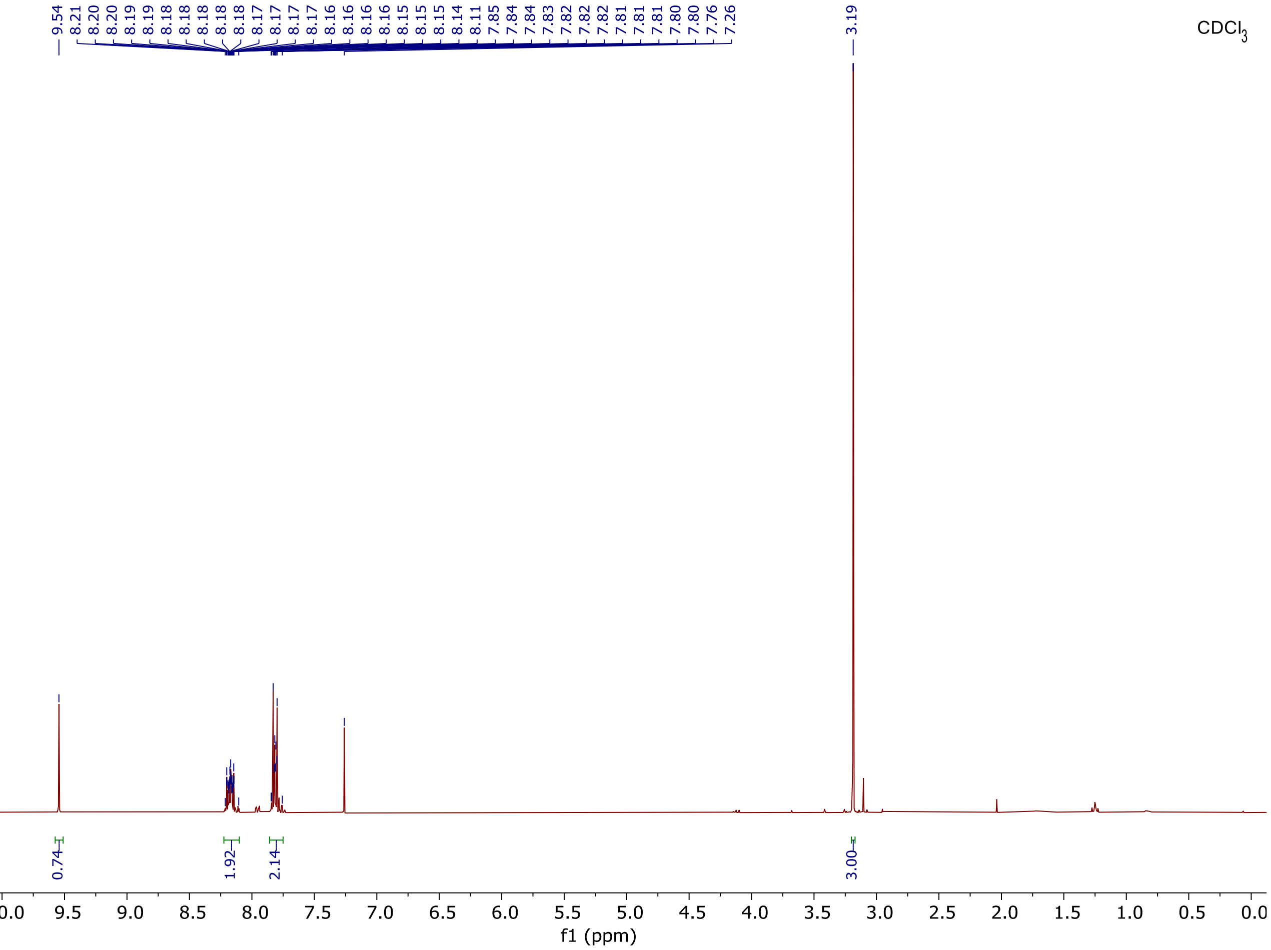

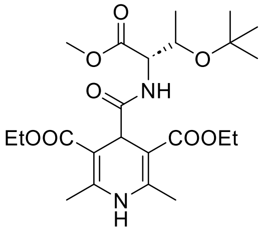
O
O
NH
O
EtOOC
COOEt
N
H

2e

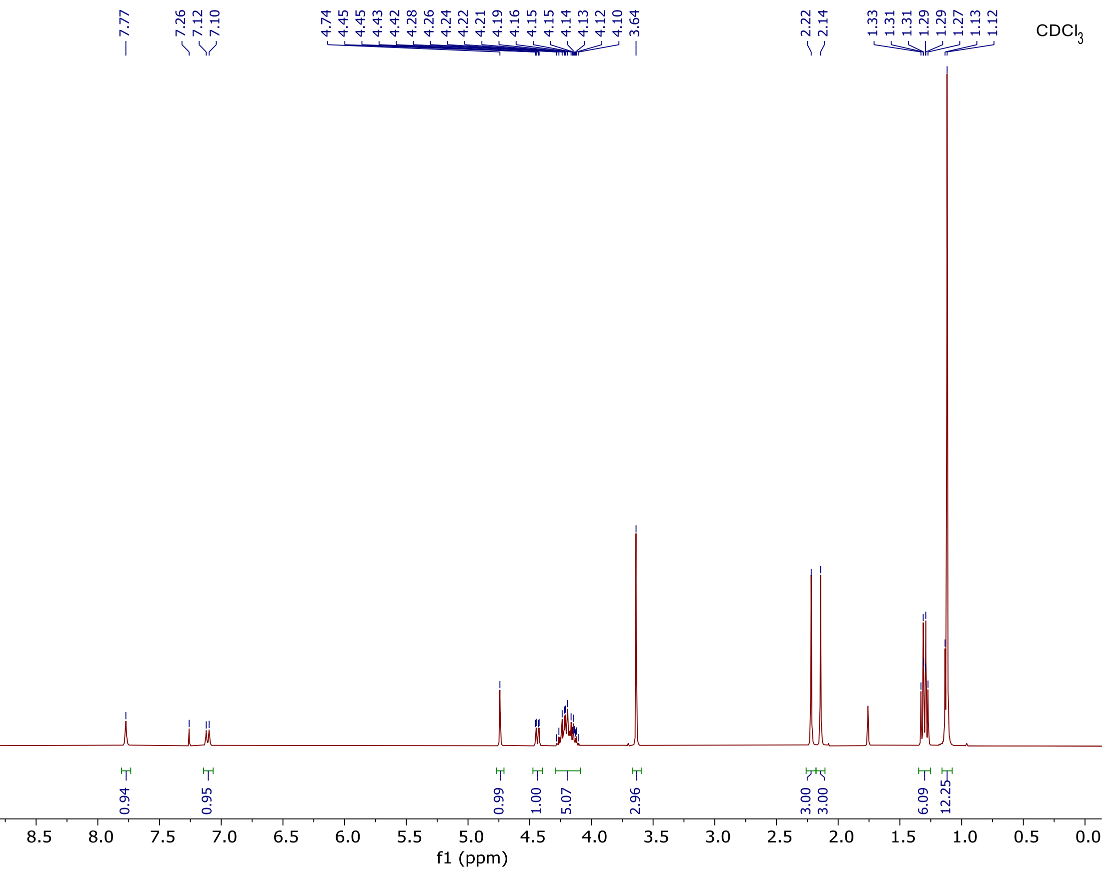
7.77
7.26
7.12
7.10
4.74
4.45
4.45
4.43
4.42
4.28
4.26
4.24
4.22
4.21
4.19
4.16
4.15
4.15
4.14
4.13
4.12
4.10
3.64
2.22
2.14
1.33
1.31
1.31
1.29
1.29
1.27
1.13
1.12
$CDCl_3$
0.94
0.95
0.99
1.00
5.07
2.96
3.00
3.00
6.09
12.25
10.0 9.5 9.0 8.5 8.0 7.5 7.0 6.5 6.0 5.5 5.0 4.5 4.0 3.5 3.0 2.5 2.0 1.5 1.0 0.5 0.0
f1 (ppm)

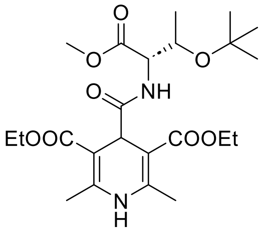
O
O
NH
O
EtOOC
COOEt
N
H

2e

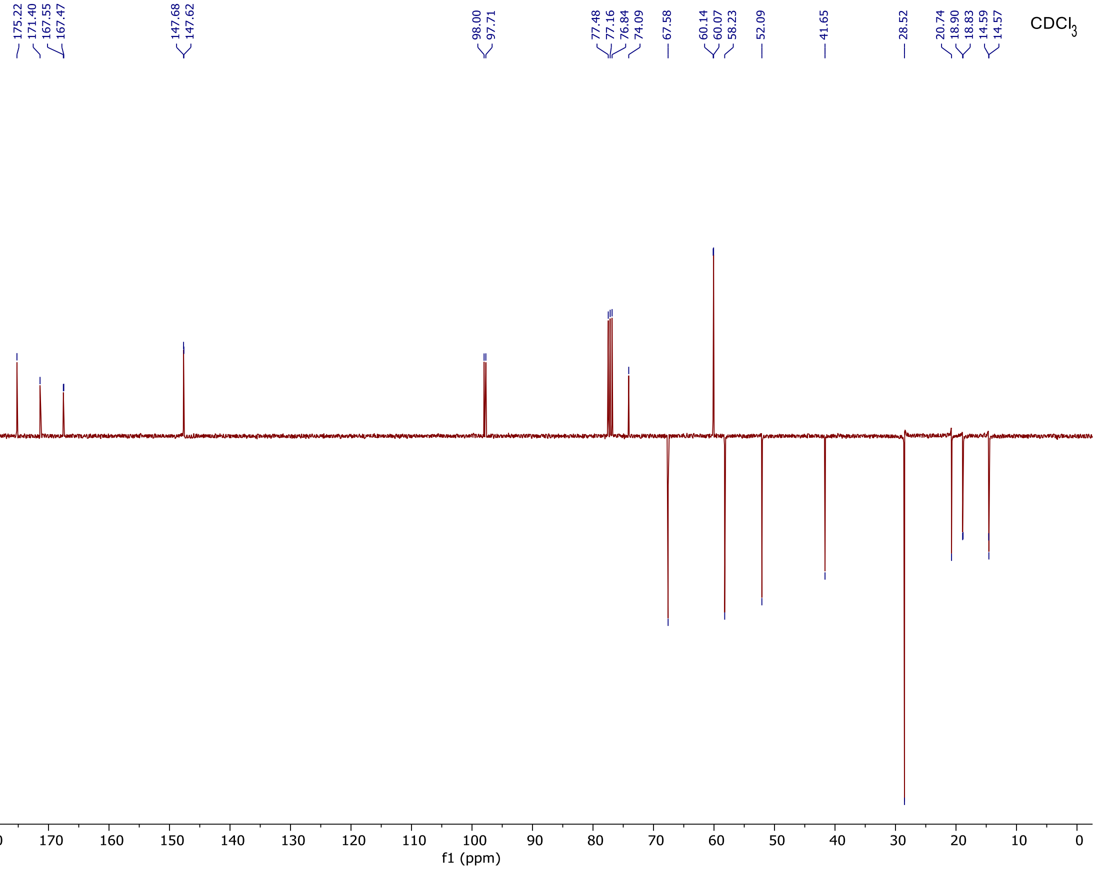
175.22
171.40
167.55
167.47
147.68
147.62
98.00
97.71
77.48
77.16
76.84
74.09
67.58
60.14
60.07
58.23
52.09
41.65
28.52
20.74
18.90
18.83
14.59
14.57
$CDCl_3$
200 190 180 170 160 150 140 130 120 110 100 90 80 70 60 50 40 30 20 10 0
f1 (ppm)

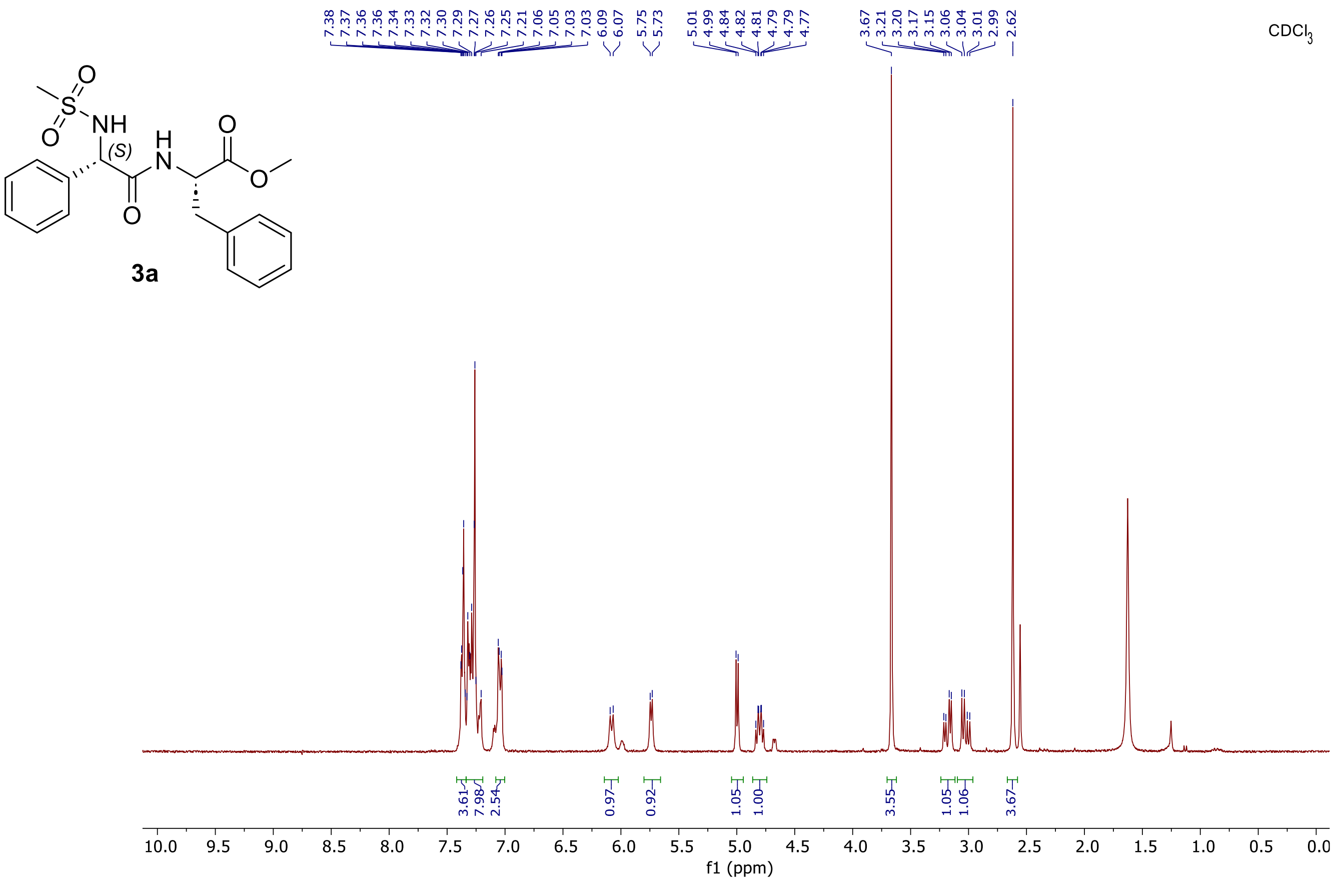
7.38 7.37 7.36 7.36 7.34 7.33 7.32 7.30 7.29 7.27 7.26 7.25 7.21 7.06 7.05 7.03 6.09 6.07 5.75 5.73 5.01 4.99 4.84 4.82 4.81 4.79 4.79 4.77 3.67 3.21 3.20 3.17 3.15 3.06 3.04 3.01 2.99 2.62
CDCl3
NH
(S)
H
N
O
3a
3.61 7.98 2.54 0.97 0.92 1.05 1.00 3.55 1.05 1.06 3.67
f1 (ppm)

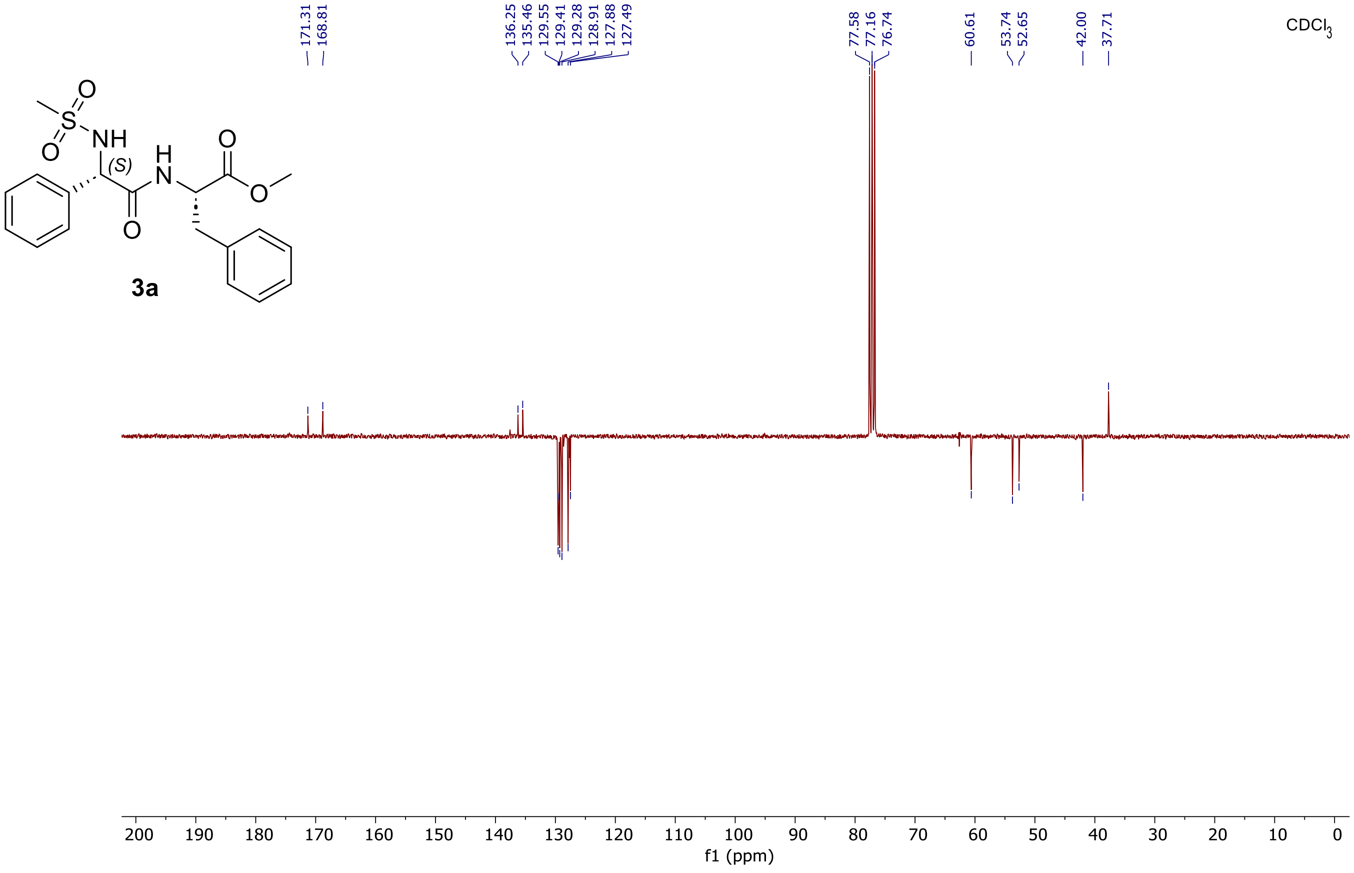
171.31 168.81 136.25 135.46 129.55 129.41 129.28 128.91 127.88 127.49 77.58 77.16 76.74 60.61 53.74 52.65 42.00 37.71
CDCl3
NH
(S)
H
N
O
3a
f1 (ppm)

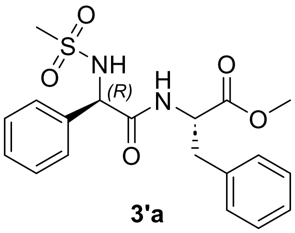
O
S
NH
O
(R)
H
N
O
O
O

3'a

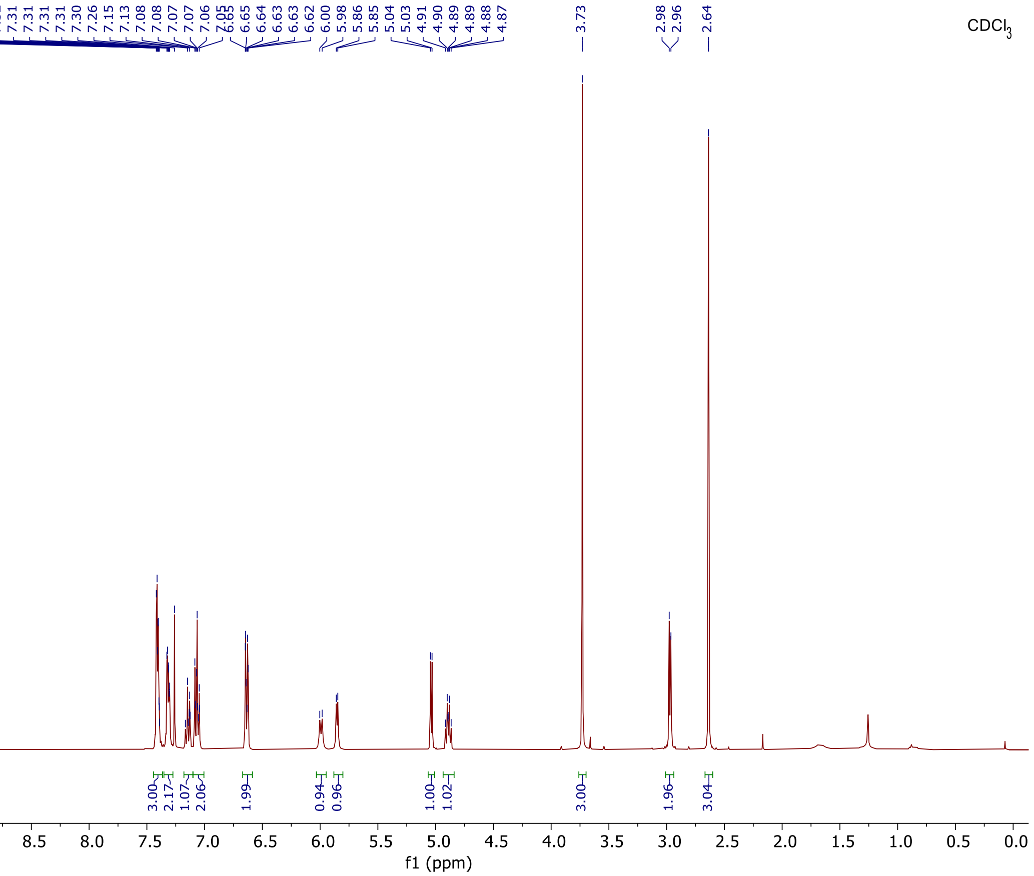
7.42 7.41 7.41 7.40 7.40 7.39 7.33 7.32 7.31 7.31 7.31 7.31 7.30 7.26 7.15 7.13 7.08 7.08 7.07 7.07 7.06 7.05 6.65 6.64 6.63 6.63 6.62 6.00 5.98 5.86 5.85 5.04 5.03 4.91 4.90 4.89 4.89 4.88 4.87
3.73
2.98 2.96
2.64
CDCl3
3.00 2.17 1.07 2.06 1.99 0.94 0.96 1.00 1.02 3.00 1.96 3.04
10.0 9.5 9.0 8.5 8.0 7.5 7.0 6.5 6.0 5.5 5.0 4.5 4.0 3.5 3.0 2.5 2.0 1.5 1.0 0.5 0.0
f1 (ppm)

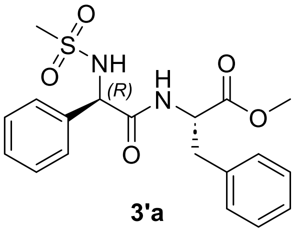
O
S
NH
O
(R)
H
N
O
O
O

3'a

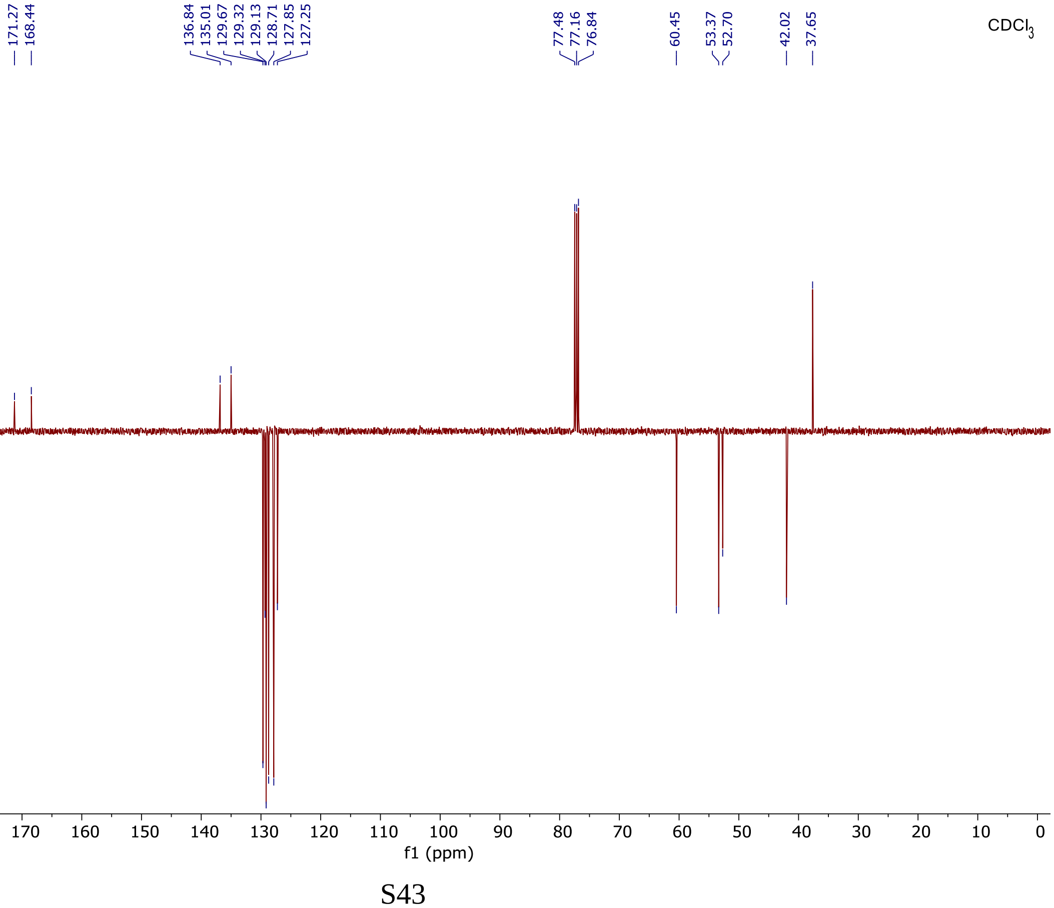
171.27 168.44
136.84 135.01 129.67 129.32 129.13 128.71 127.85 127.25
77.48 77.16 76.84
60.45
53.37 52.70
42.02
37.65
CDCl3
200 190 180 170 160 150 140 130 120 110 100 90 80 70 60 50 40 30 20 10 0
f1 (ppm)

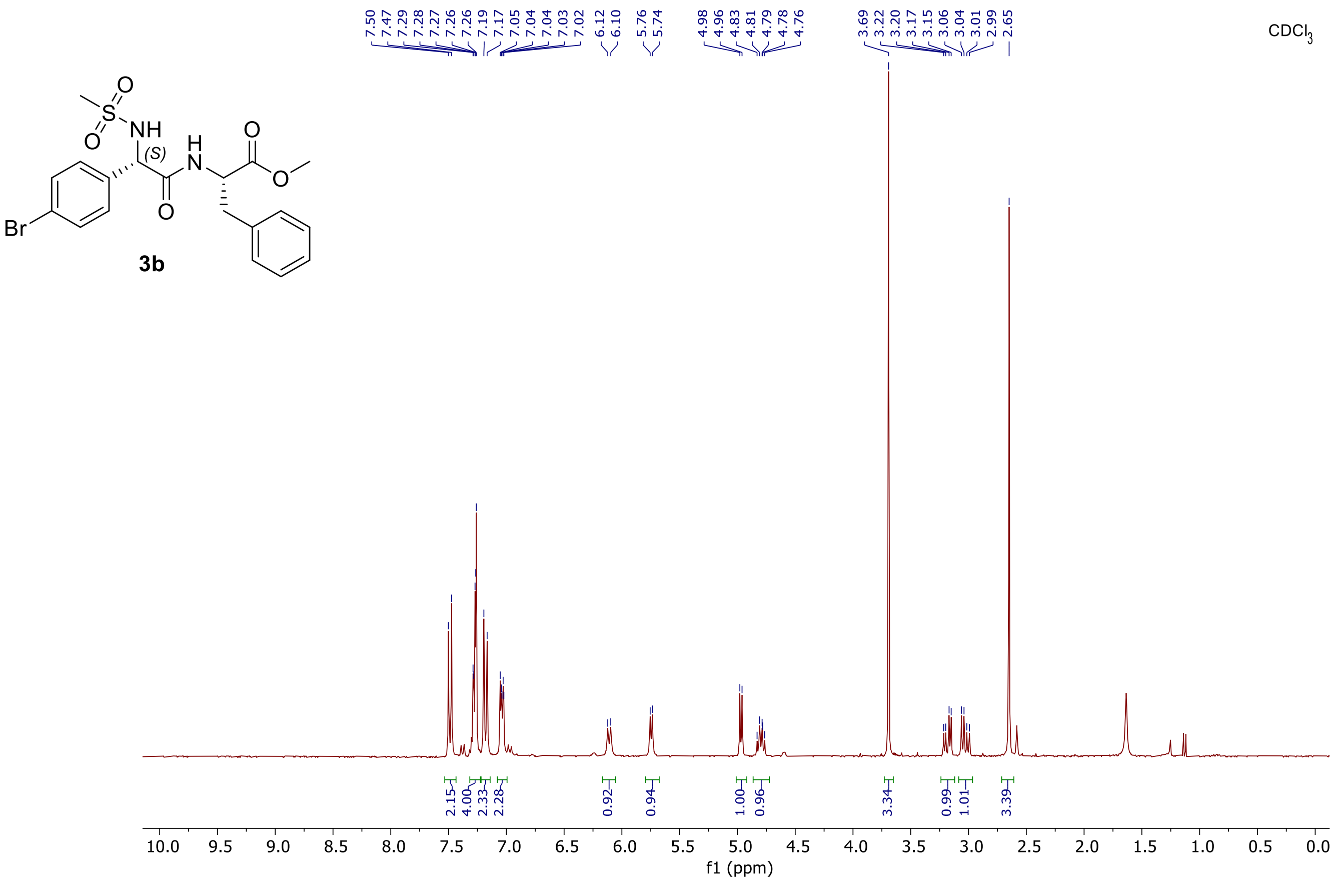

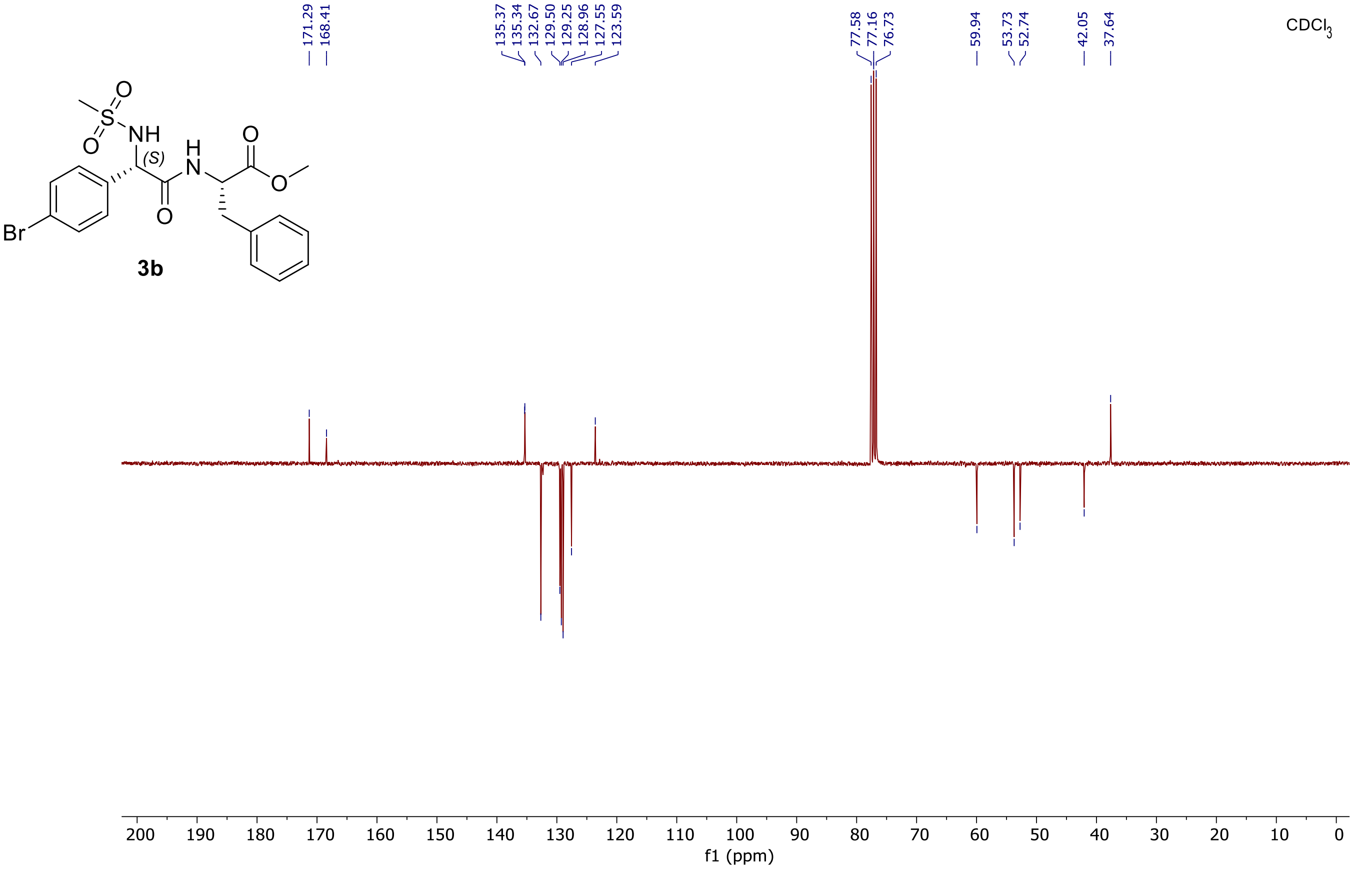

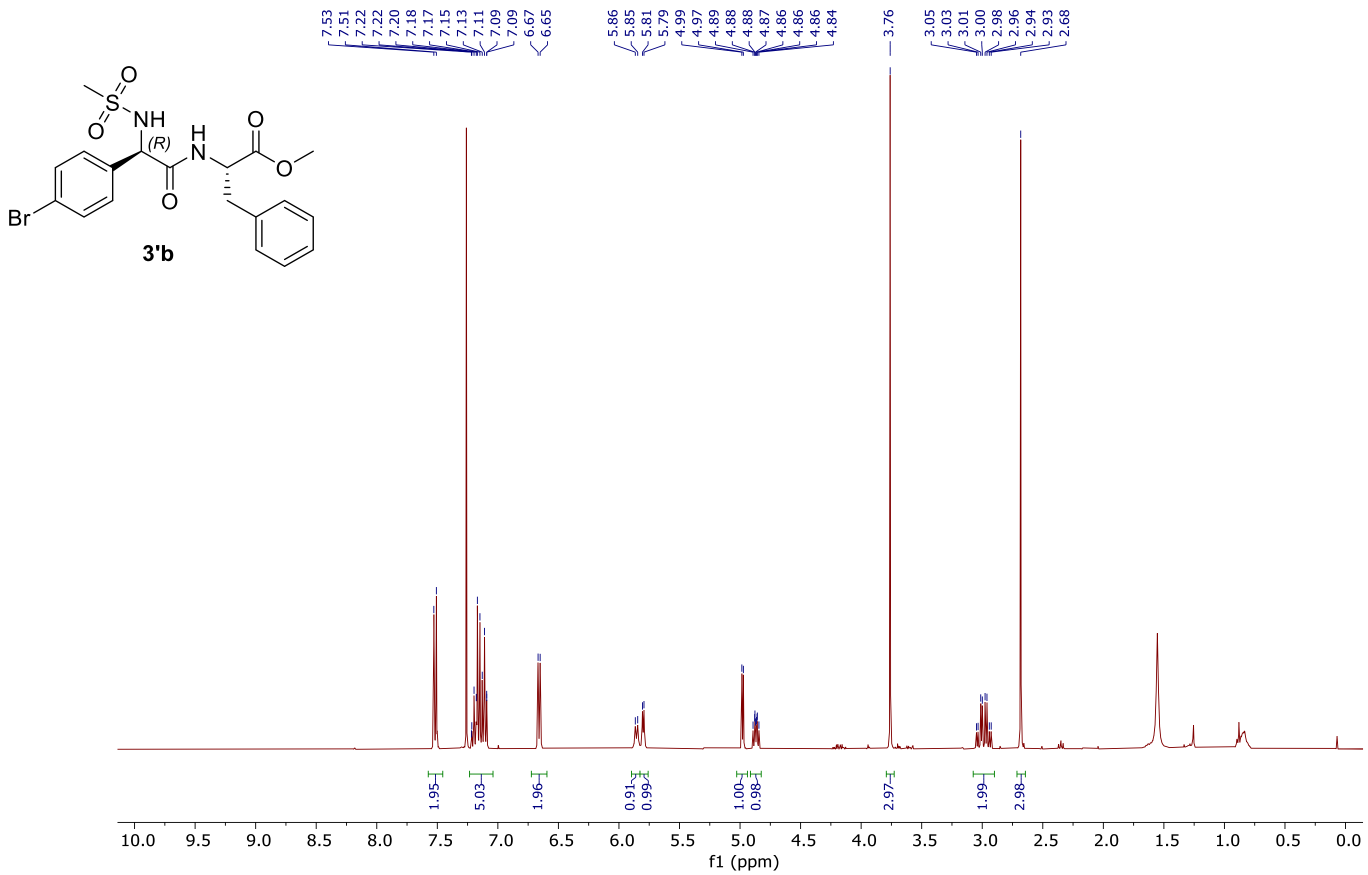
7.53 7.51 7.22 7.22 7.20 7.18 7.17 7.15 7.13 7.11 7.09 7.09 6.67 6.65
5.86 5.85 5.81 5.79 4.99 4.97 4.89 4.88 4.88 4.87 4.86 4.86 4.86 4.84
3.76
3.05 3.03 3.01 3.00 2.98 2.96 2.94 2.93 2.68
NH
(R)
H
N
O
Br
3'b
1.95 5.03 1.96 0.91 0.99 1.00 0.98 2.97 1.99 2.98
10.0 9.5 9.0 8.5 8.0 7.5 7.0 6.5 6.0 5.5 5.0 4.5 4.0 3.5 3.0 2.5 2.0 1.5 1.0 0.5 0.0
f1 (ppm)

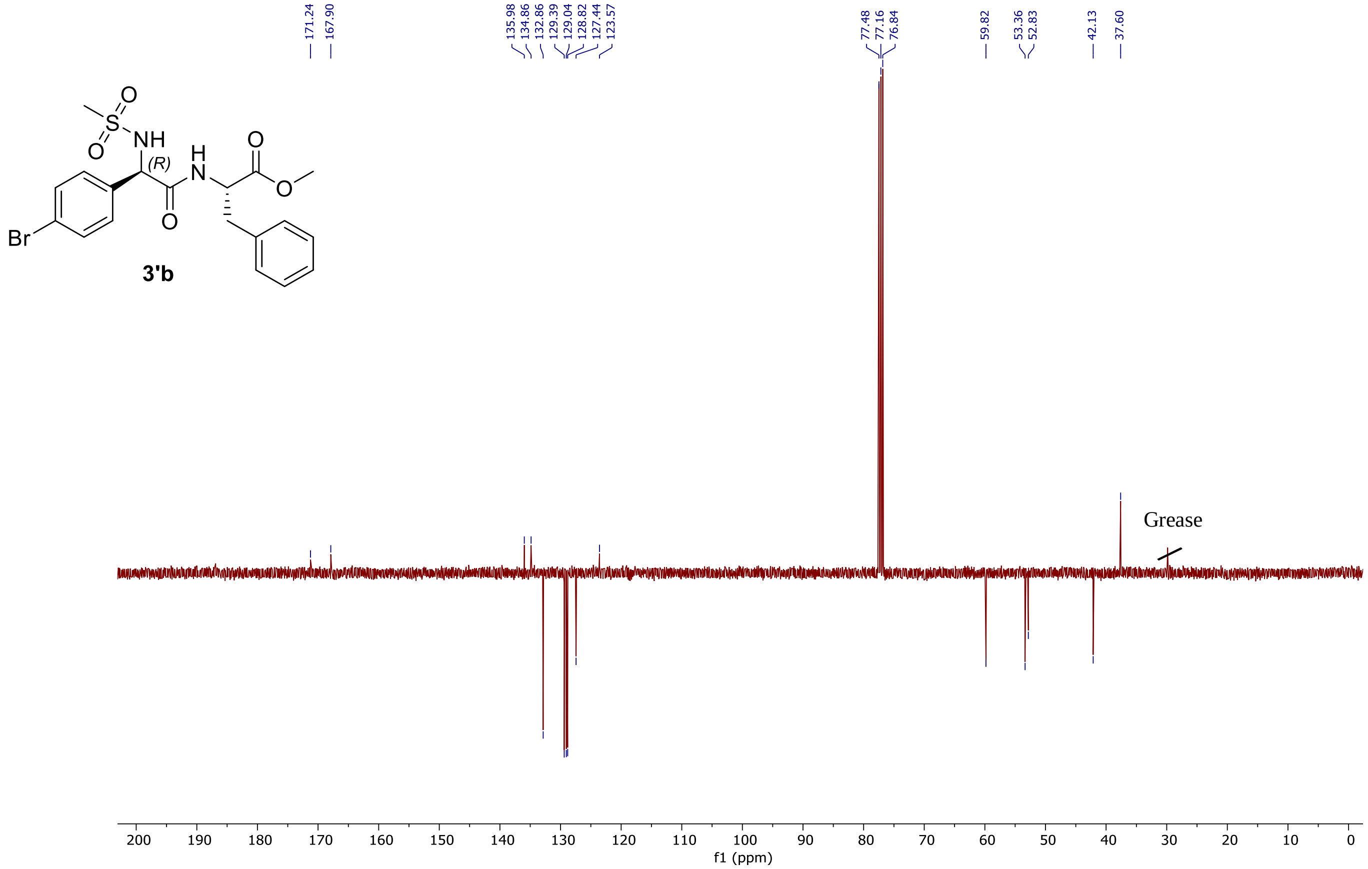
171.24 167.90
135.98 134.86 132.86 129.39 129.04 128.82 127.44 123.57
77.48 77.16 76.84
59.82 53.36 52.83 42.13 37.60
NH
(R)
H
N
O
Br
3'b
Grease
200 190 180 170 160 150 140 130 120 110 100 90 80 70 60 50 40 30 20 10 0
f1 (ppm)

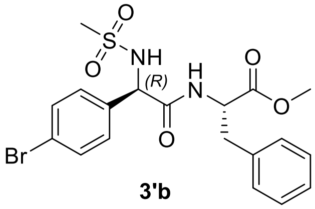

3'b

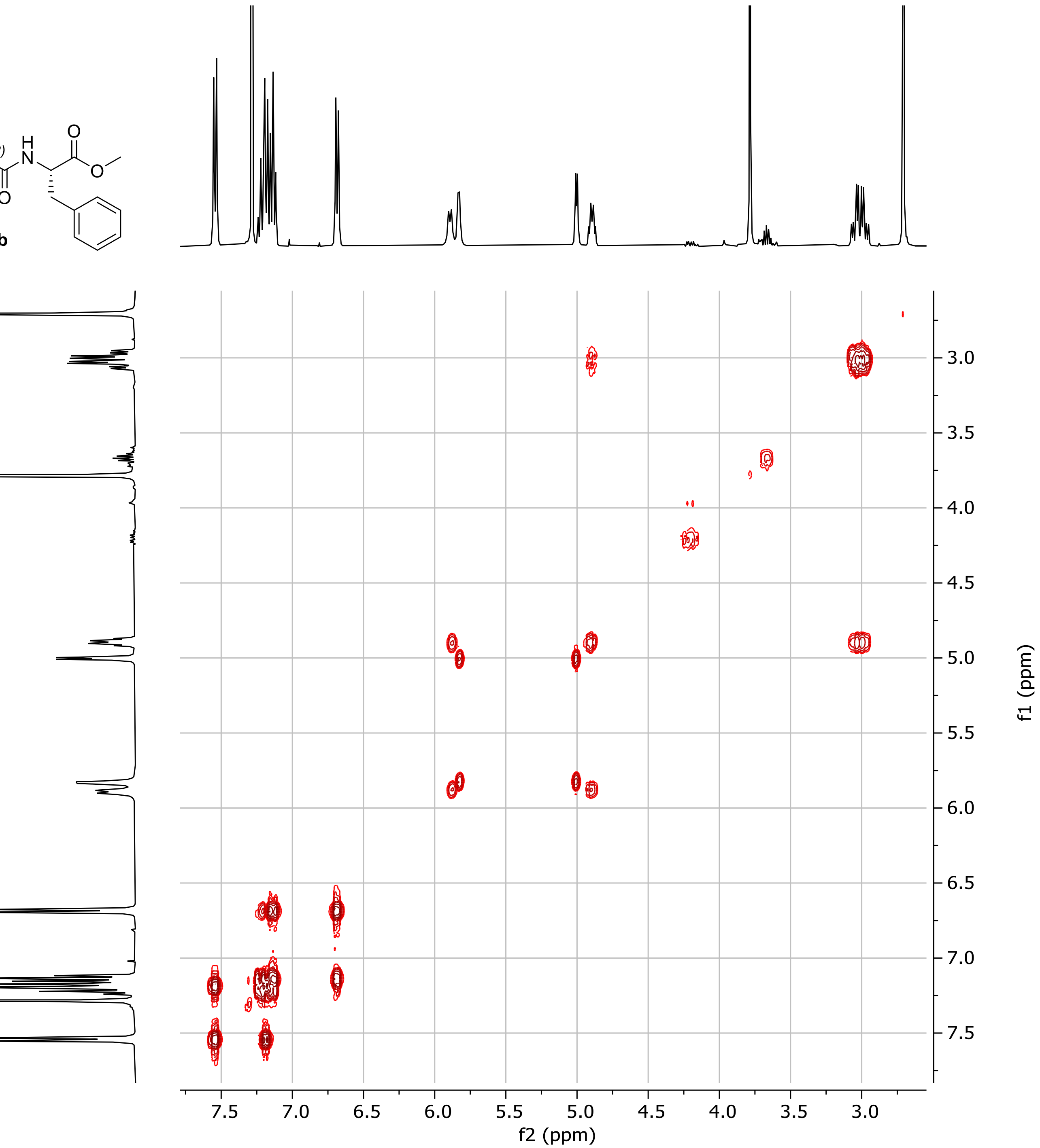

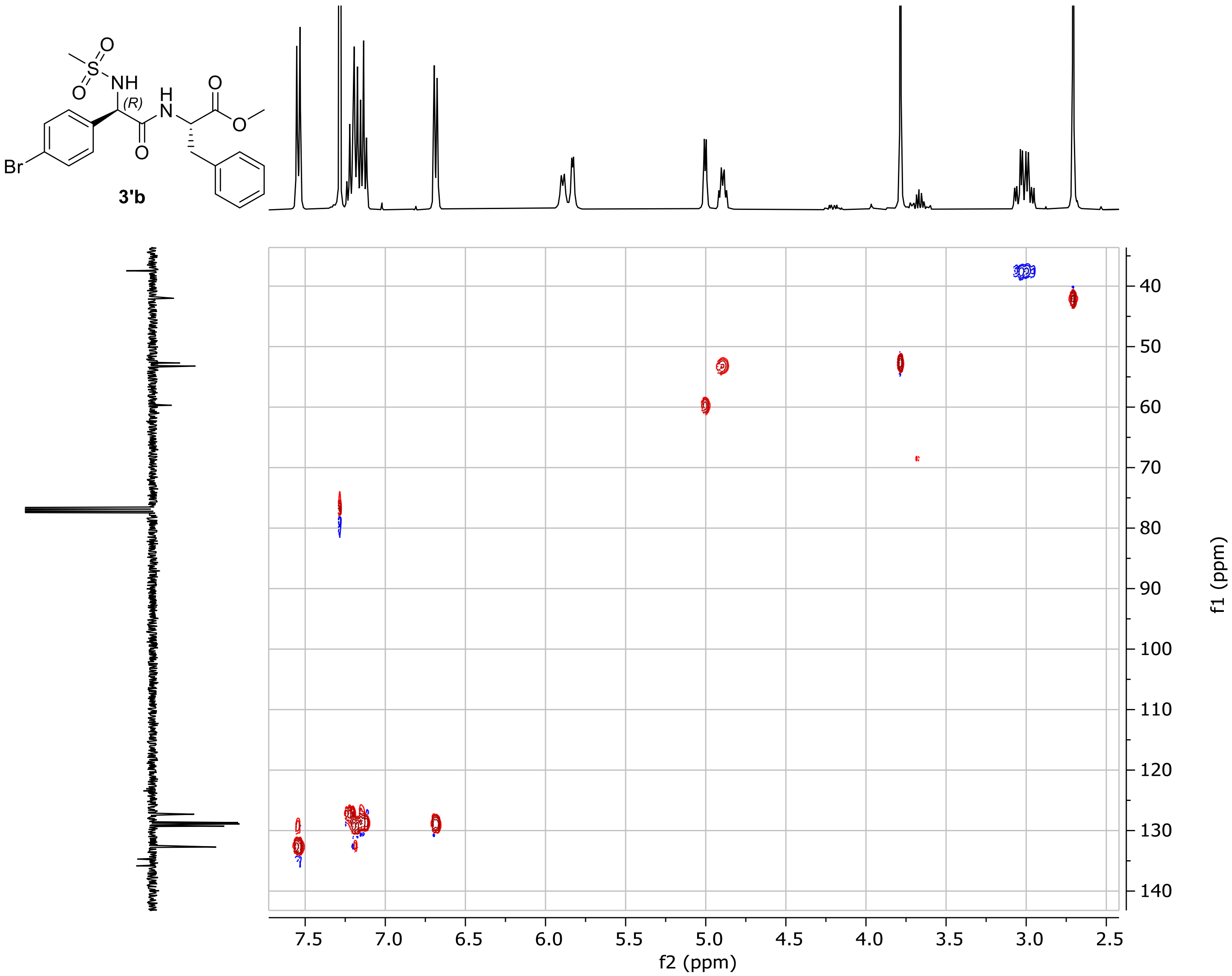

3'b
f1 (ppm)
f2 (ppm)

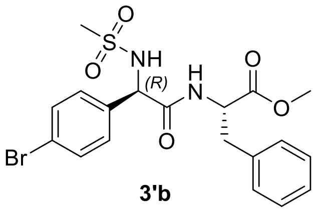

3'b

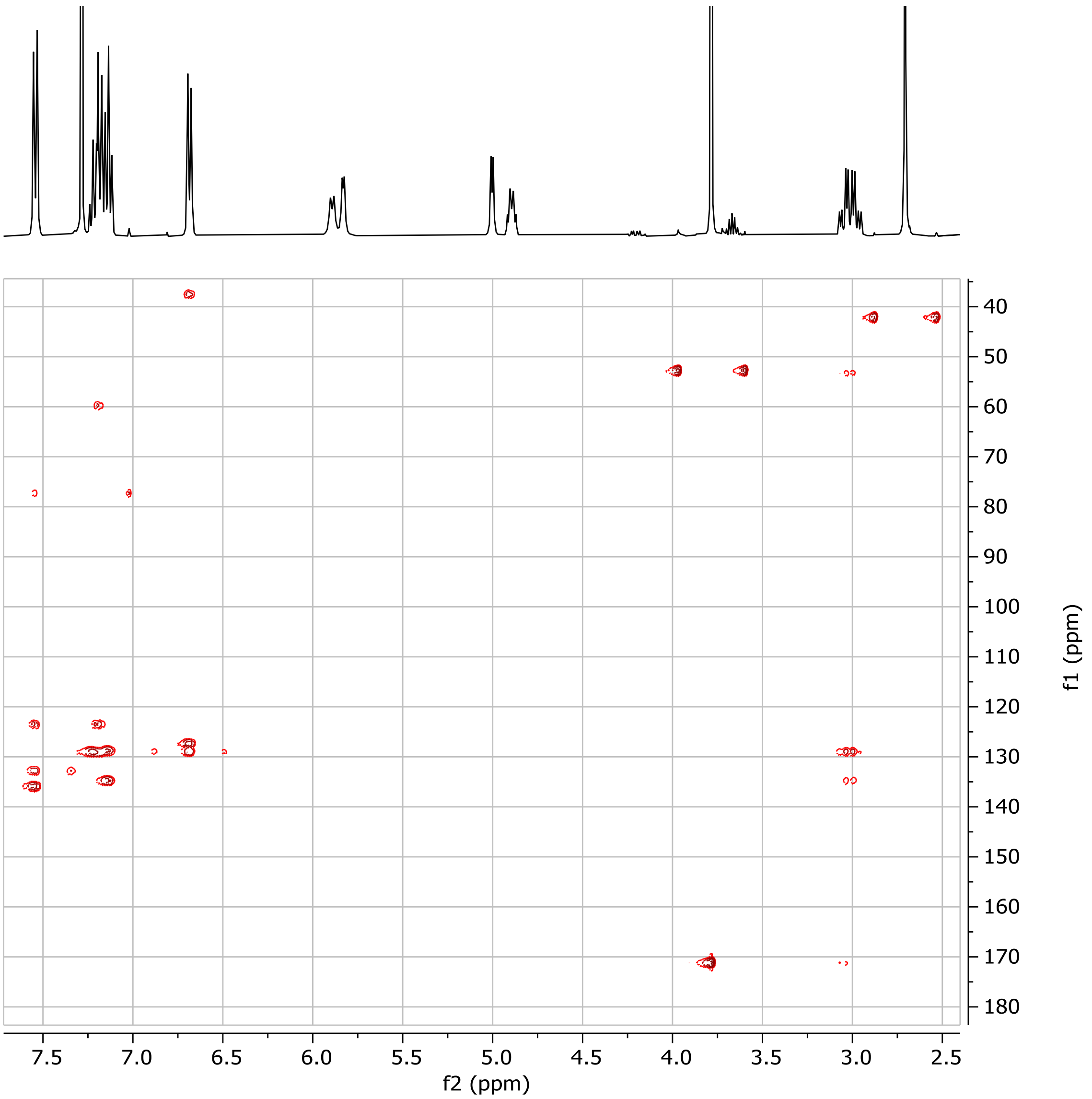

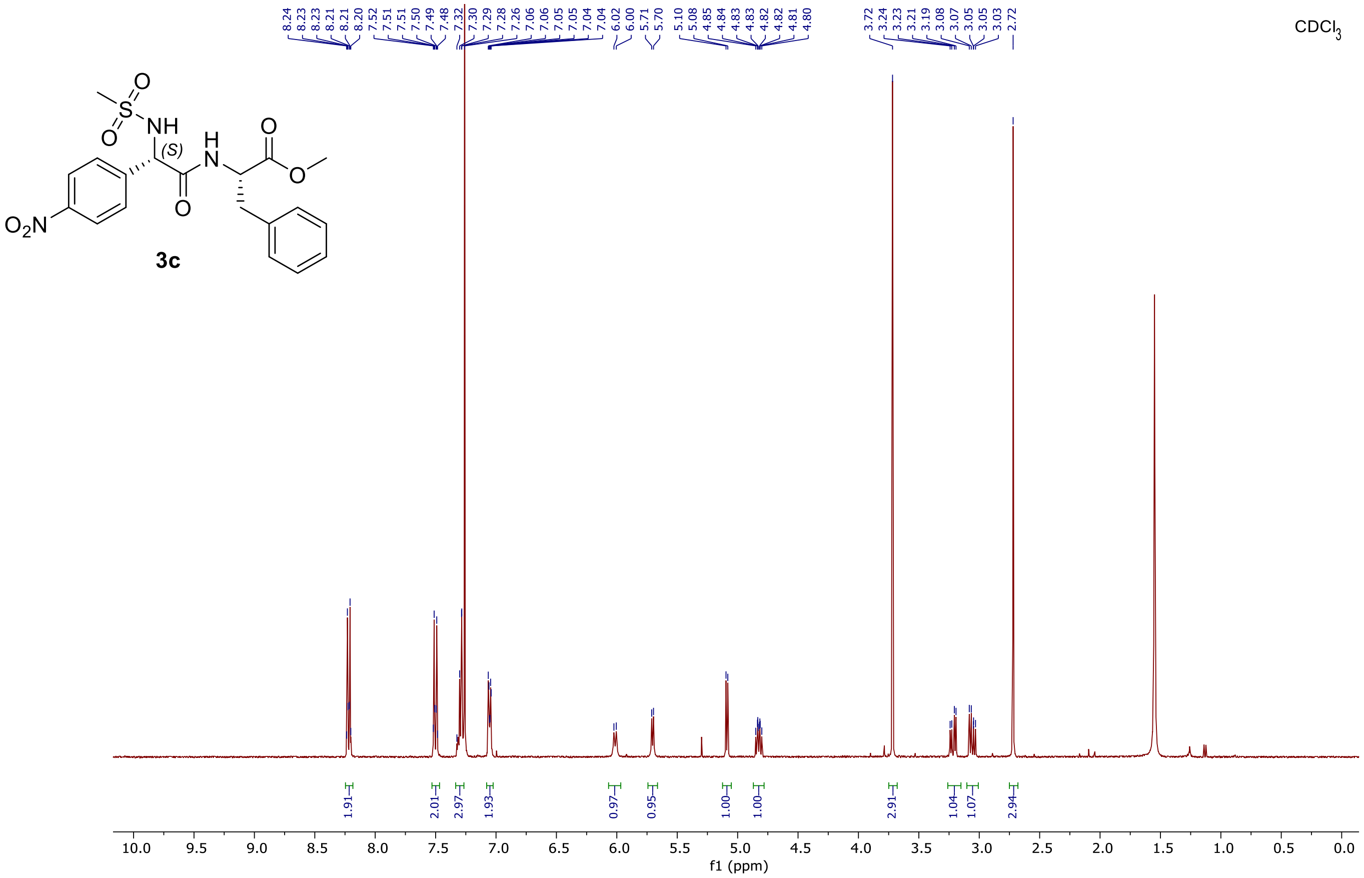

3c

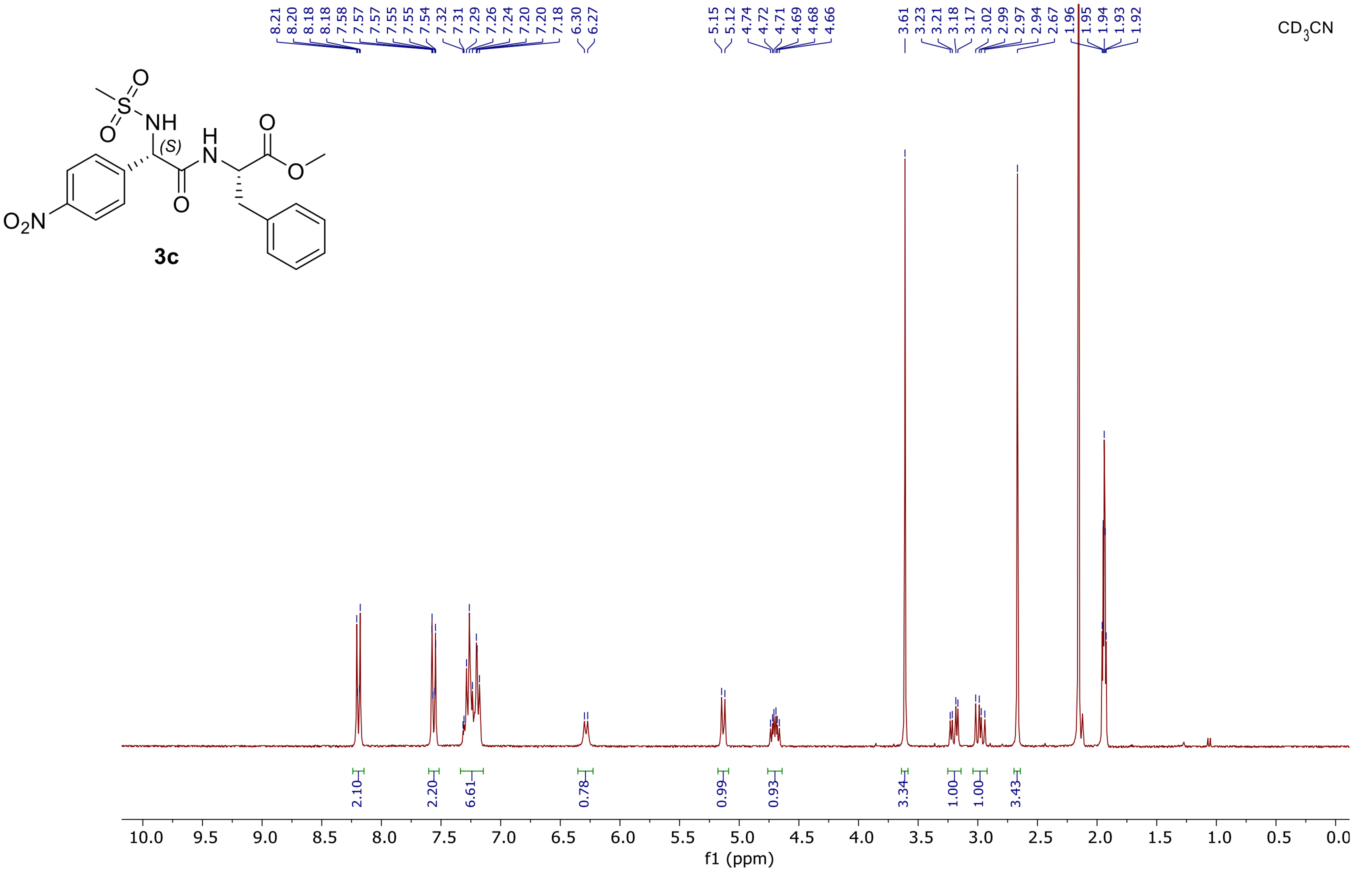

3c

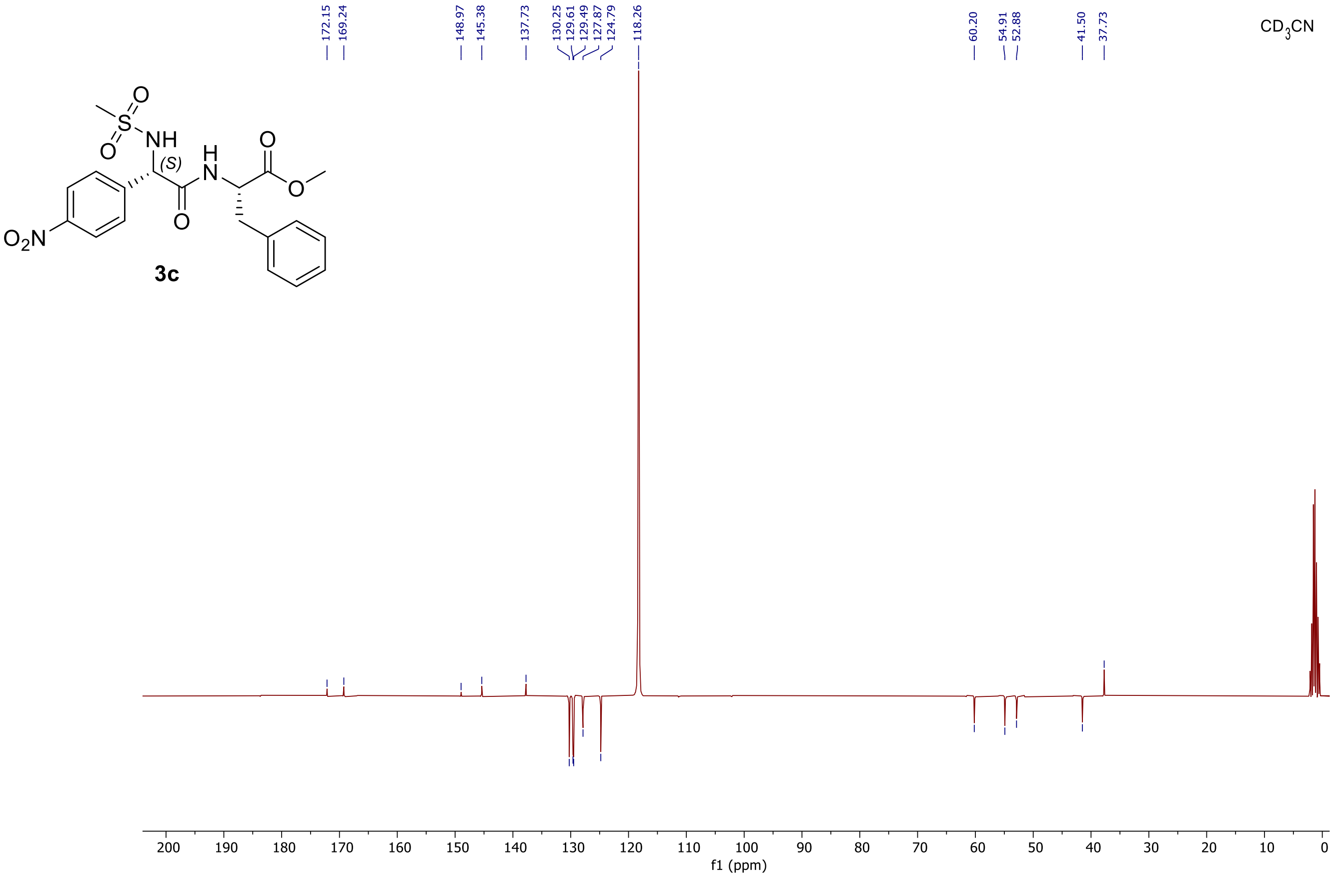
172.15
169.24
148.97
145.38
137.73
130.25
129.61
129.49
127.87
124.79
118.26
60.20
54.91
52.88
41.50
37.73
CD3CN
O2N
NH
(S)
3c
f1 (ppm)

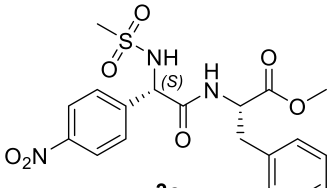

O
S
O
NH
(S)
H
N
O
O
O
O_2N

3c

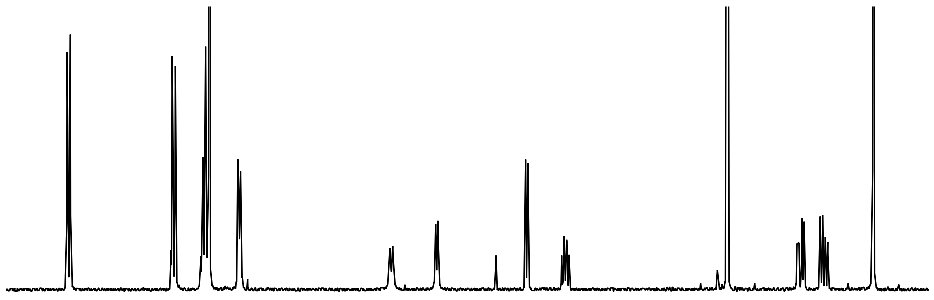

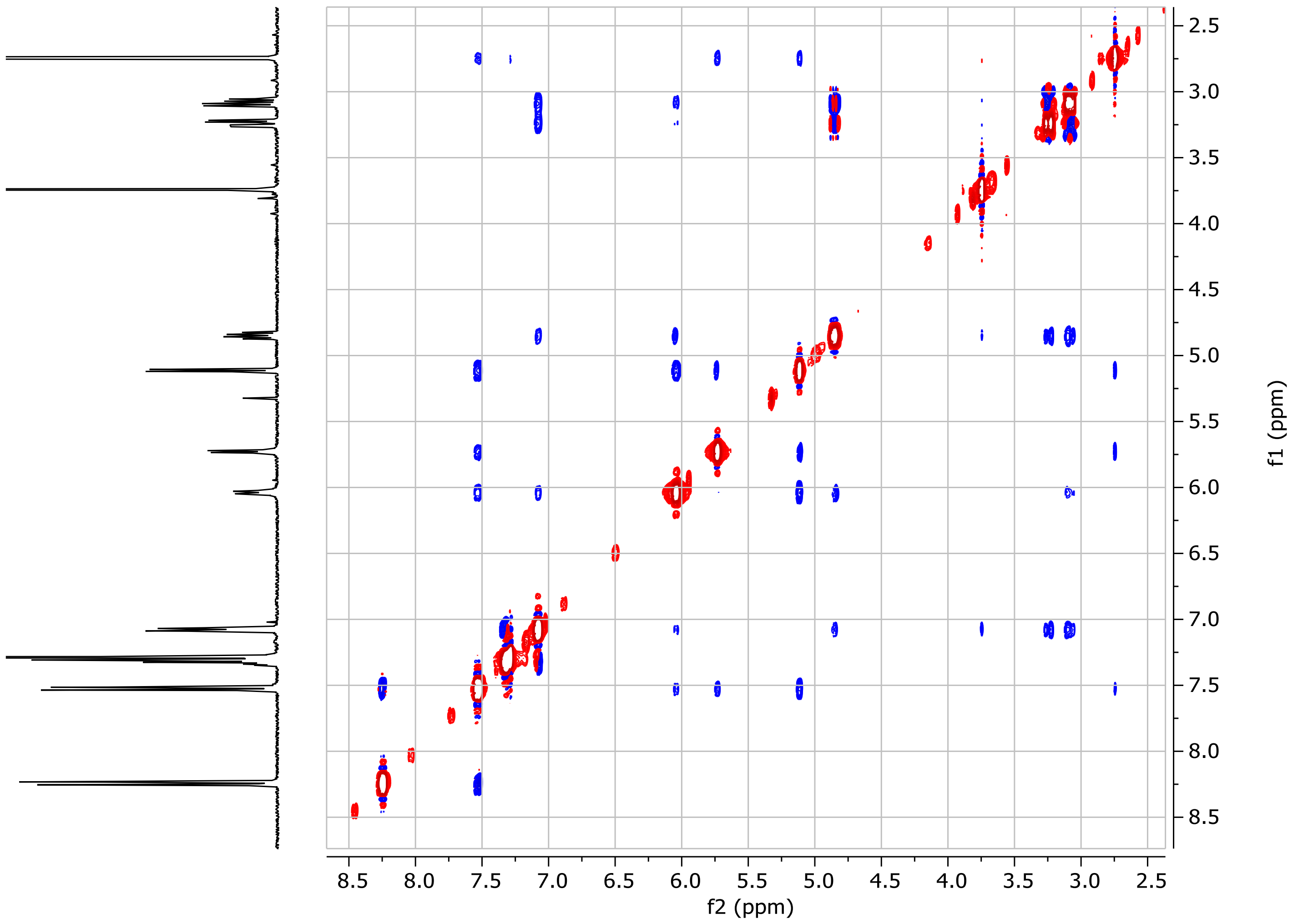

f2 (ppm)
f1 (ppm)
8.5
8.0
7.5
7.0
6.5
6.0
5.5
5.0
4.5
4.0
3.5
3.0
2.5

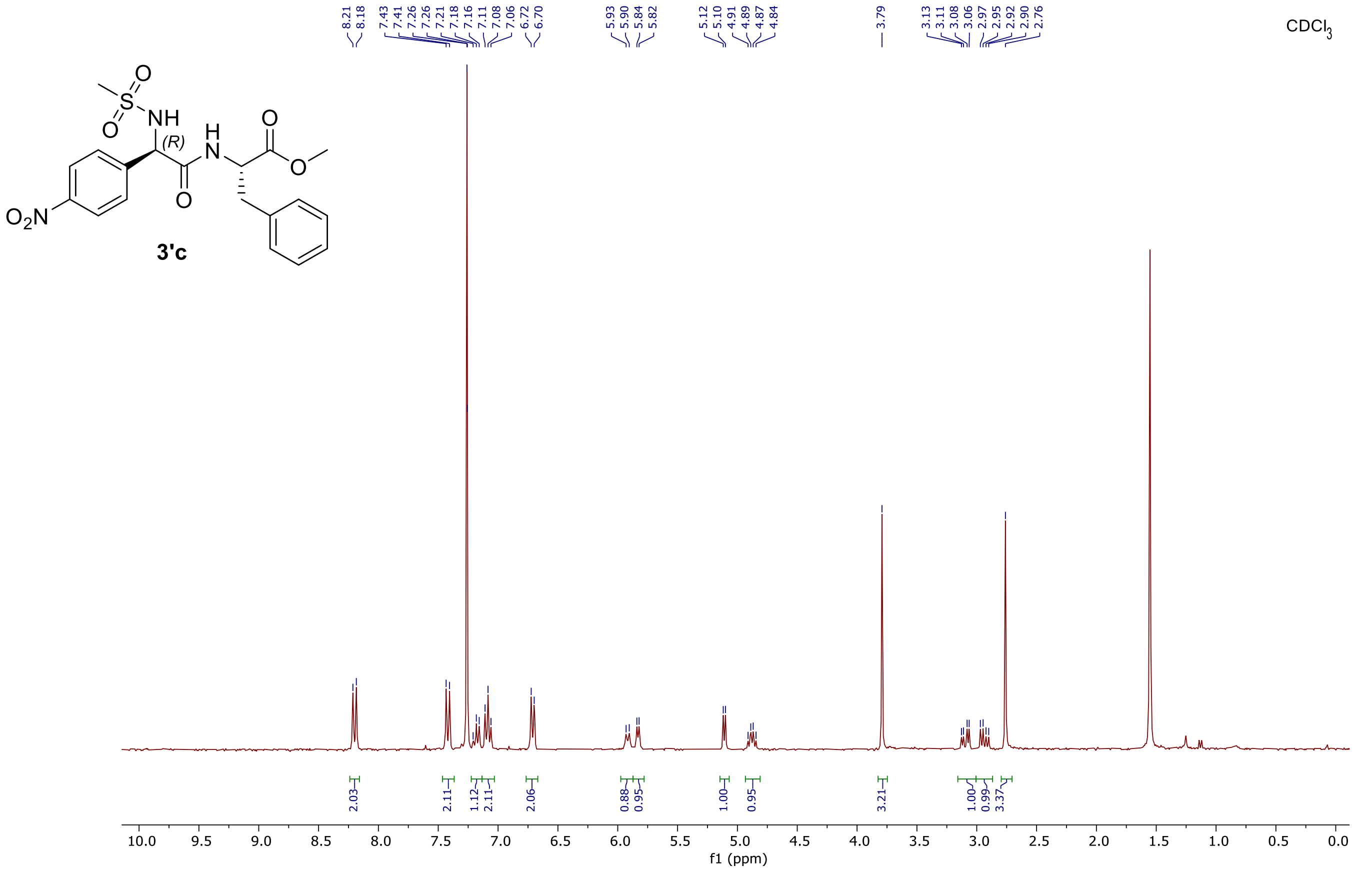

CDCl3
8.21 8.18 7.43 7.41 7.26 7.26 7.21 7.18 7.16 7.11 7.08 7.06 6.72 6.70
5.93 5.90 5.84 5.82
5.12 5.10 4.91 4.89 4.87 4.84
3.79
3.13 3.11 3.08 3.06 2.97 2.95 2.92 2.90 2.76
2.03 2.11 1.12 2.11 2.06 0.88 0.95 1.00 0.95 3.21 1.00 0.99 3.37
f1 (ppm)

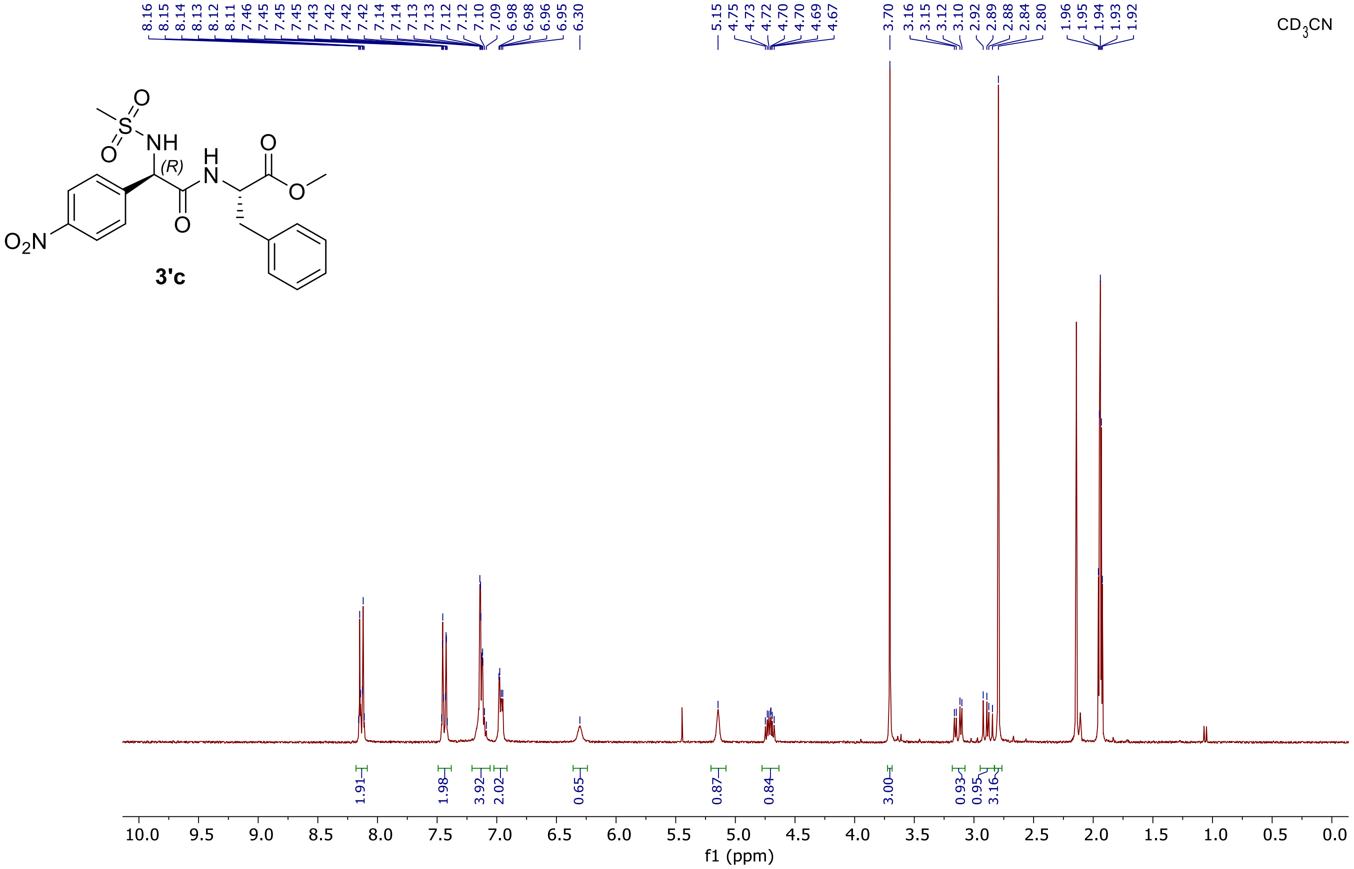

CD3CN
8.16 8.15 8.14 8.13 8.12 8.11 7.46 7.45 7.45 7.45 7.43 7.42 7.42 7.42 7.14 7.14 7.13 7.13 7.12 7.12 7.10 7.09 6.98 6.96 6.95 6.30
5.15 4.75 4.73 4.72 4.70 4.70 4.69 4.67
3.70
3.16 3.15 3.12 3.10 2.92 2.89 2.88 2.84 2.80
1.96 1.95 1.94 1.93 1.92
1.91 1.98 3.92 2.02 0.65 0.87 0.84 3.00 0.93 0.95 3.16
f1 (ppm)

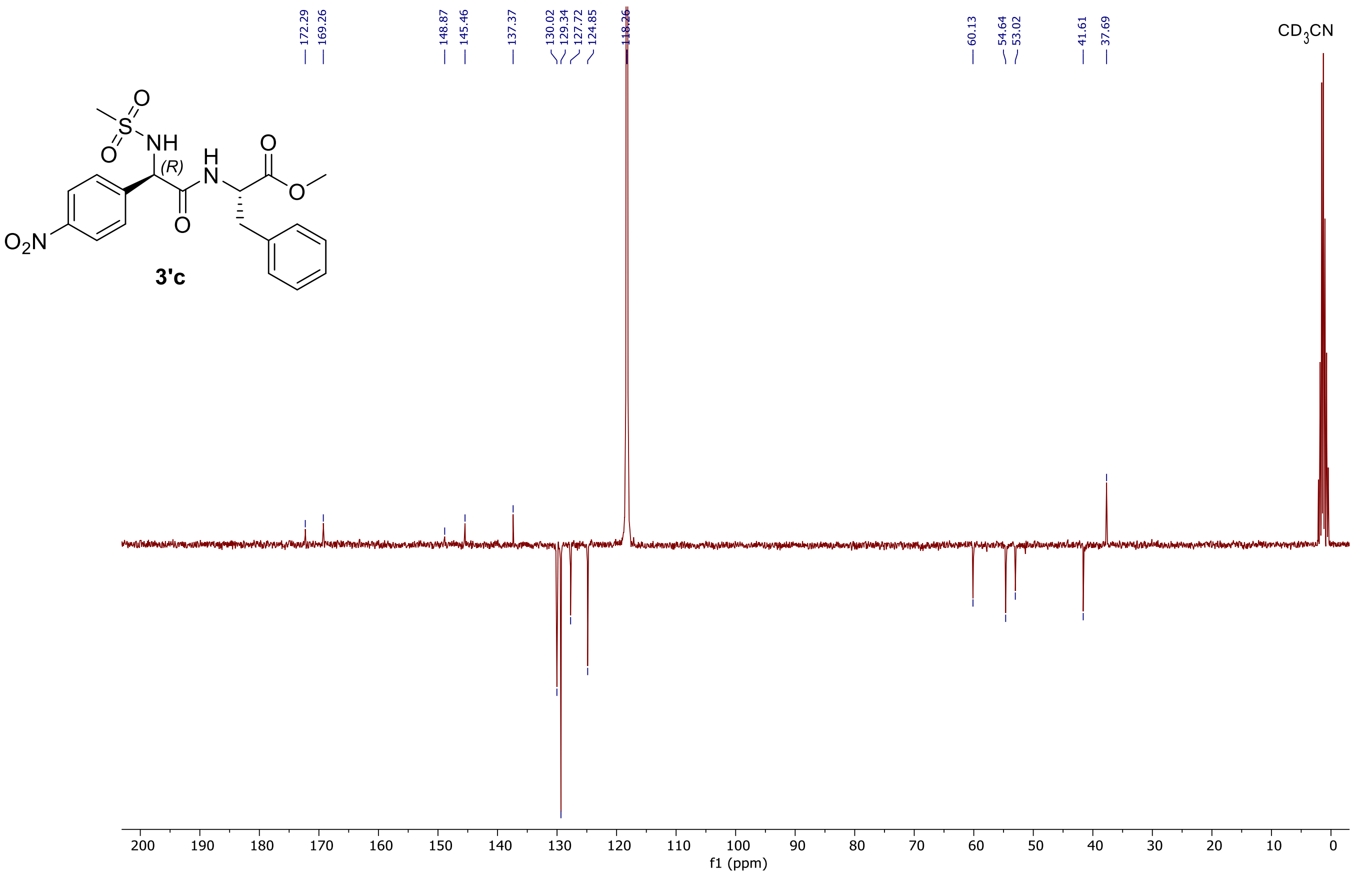

3'c
172.29
169.26
148.87
145.46
137.37
130.02
129.34
127.72
124.85
118.26
60.13
54.64
53.02
41.61
37.69
CD3CN
200 190 180 170 160 150 140 130 120 110 100 90 80 70 60 50 40 30 20 10 0
f1 (ppm)

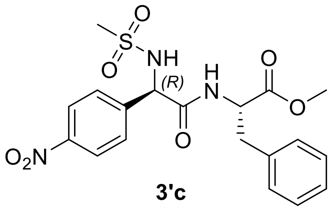
O
S
NH
(R)
H
N
O
O
O
O
O
O_2N

3'c

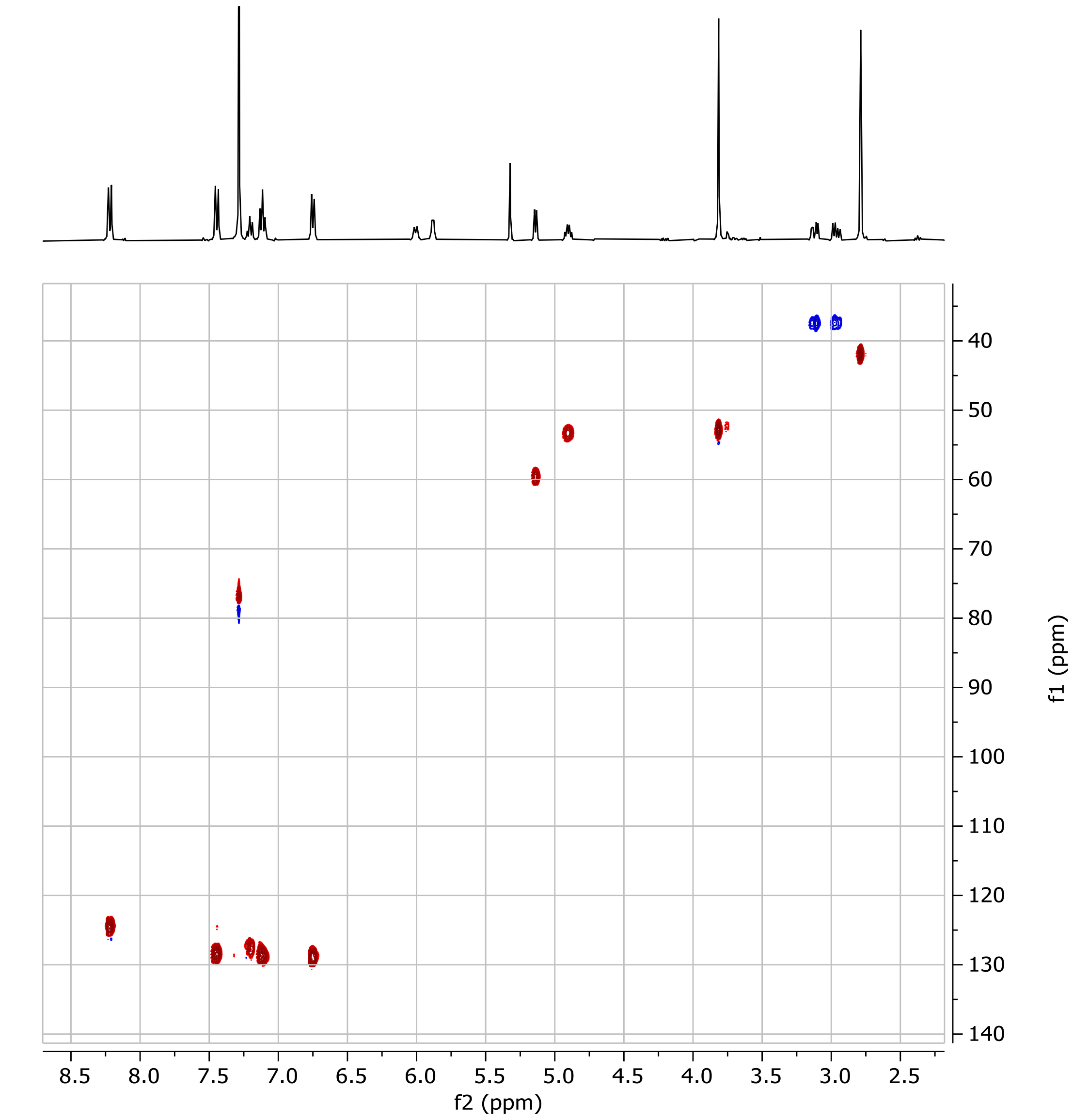
40
50
60
70
80
90
100
110
120
130
140
f1 (ppm)
8.5
8.0
7.5
7.0
6.5
6.0
5.5
5.0
4.5
4.0
3.5
3.0
2.5
f2 (ppm)

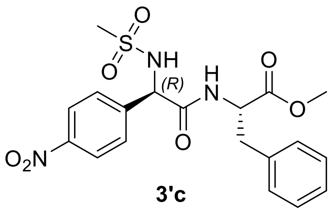

NH
(R)
H
N
O
O
S
O
O
O
O₂N

3'c

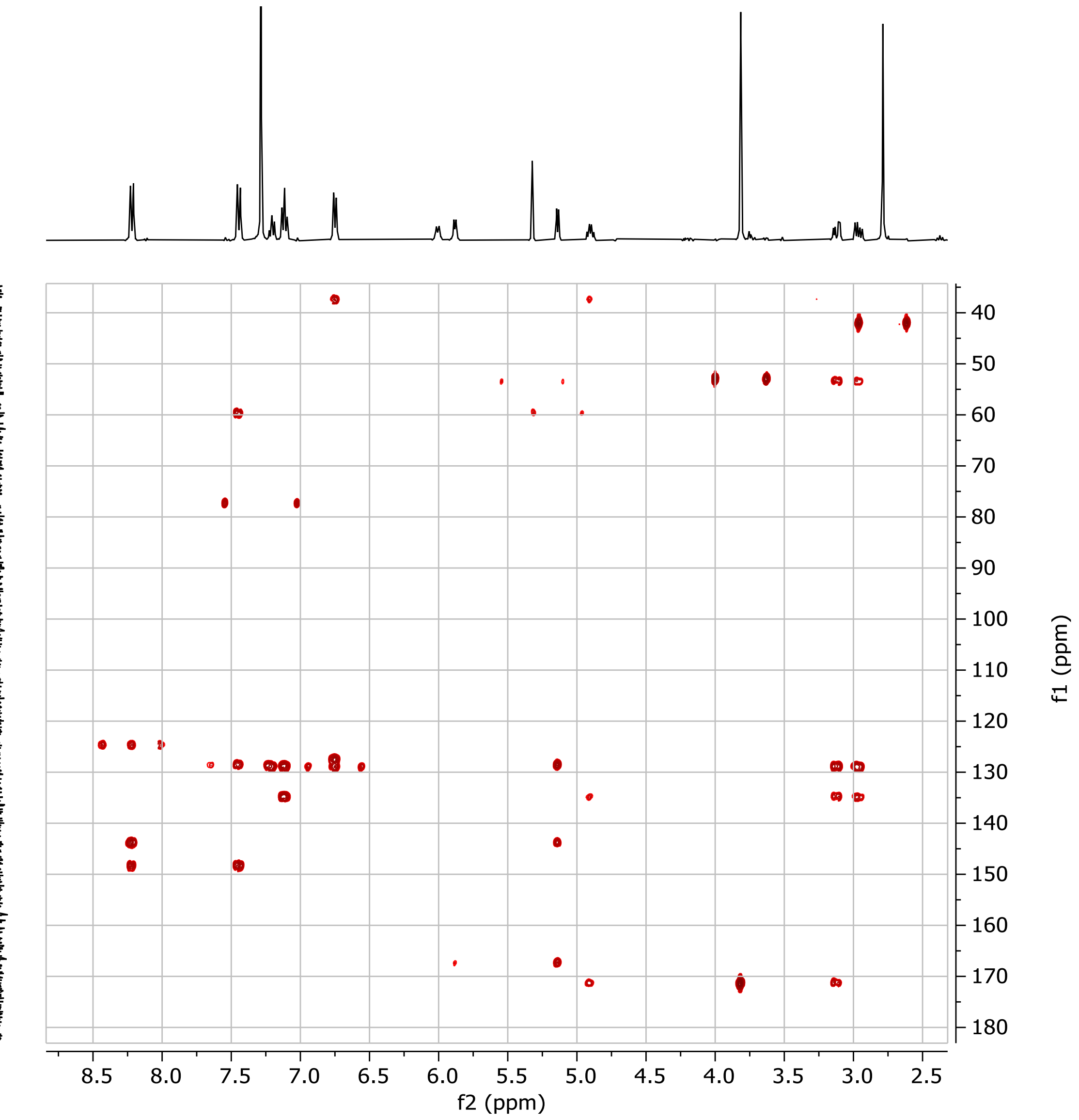

8.5
8.0
7.5
7.0
6.5
6.0
5.5
5.0
4.5
4.0
3.5
3.0
2.5
f2 (ppm)
40
50
60
70
80
90
100
110
120
130
140
150
160
170
180
f1 (ppm)

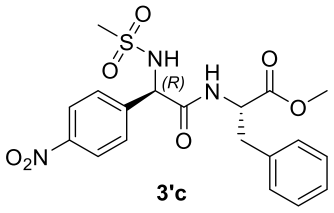
O
S
O
NH
(R)
H
N
O
O
O
O2N

3'c

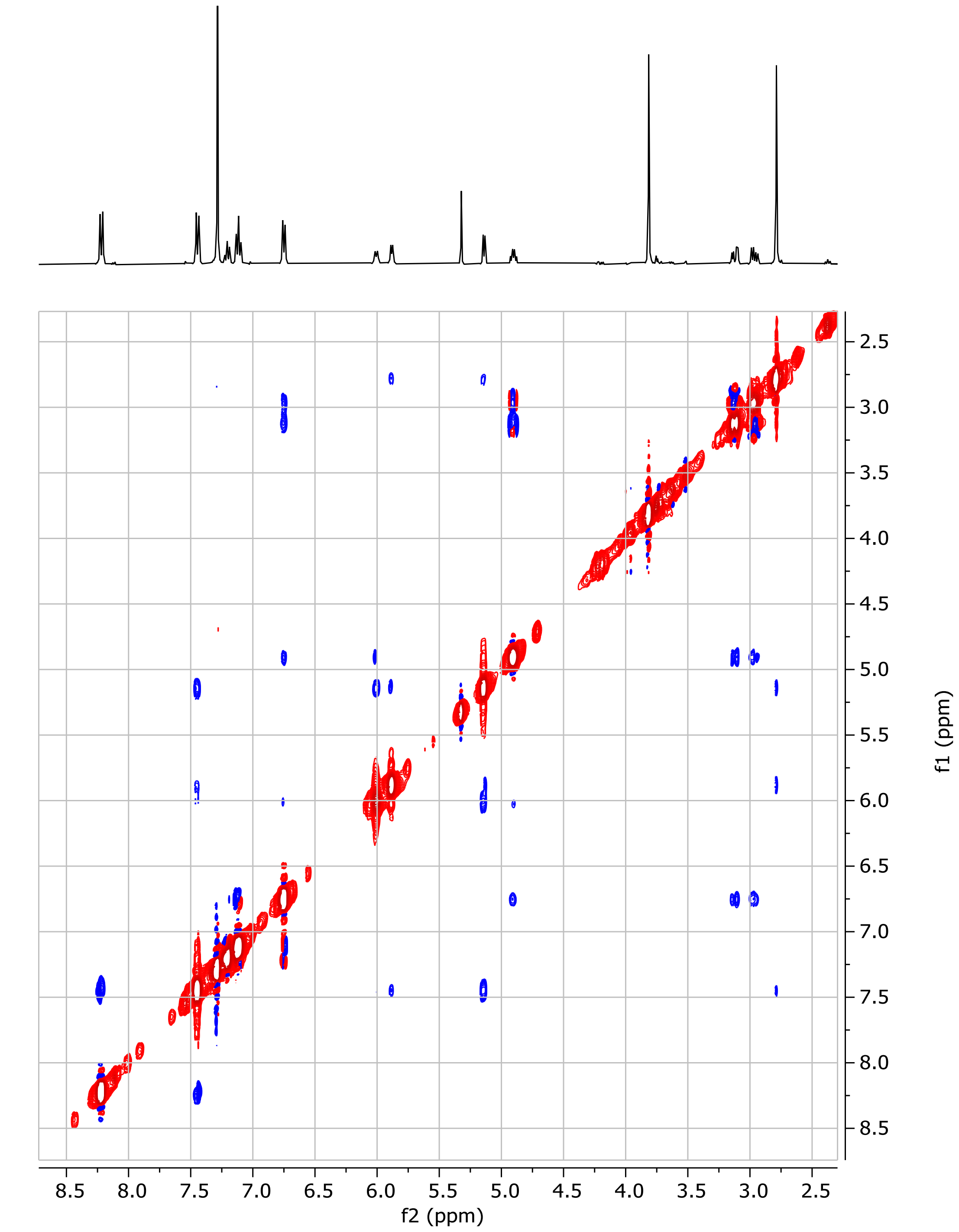
8.5
8.0
7.5
7.0
6.5
6.0
5.5
5.0
4.5
4.0
3.5
3.0
2.5
f2 (ppm)
f1 (ppm)

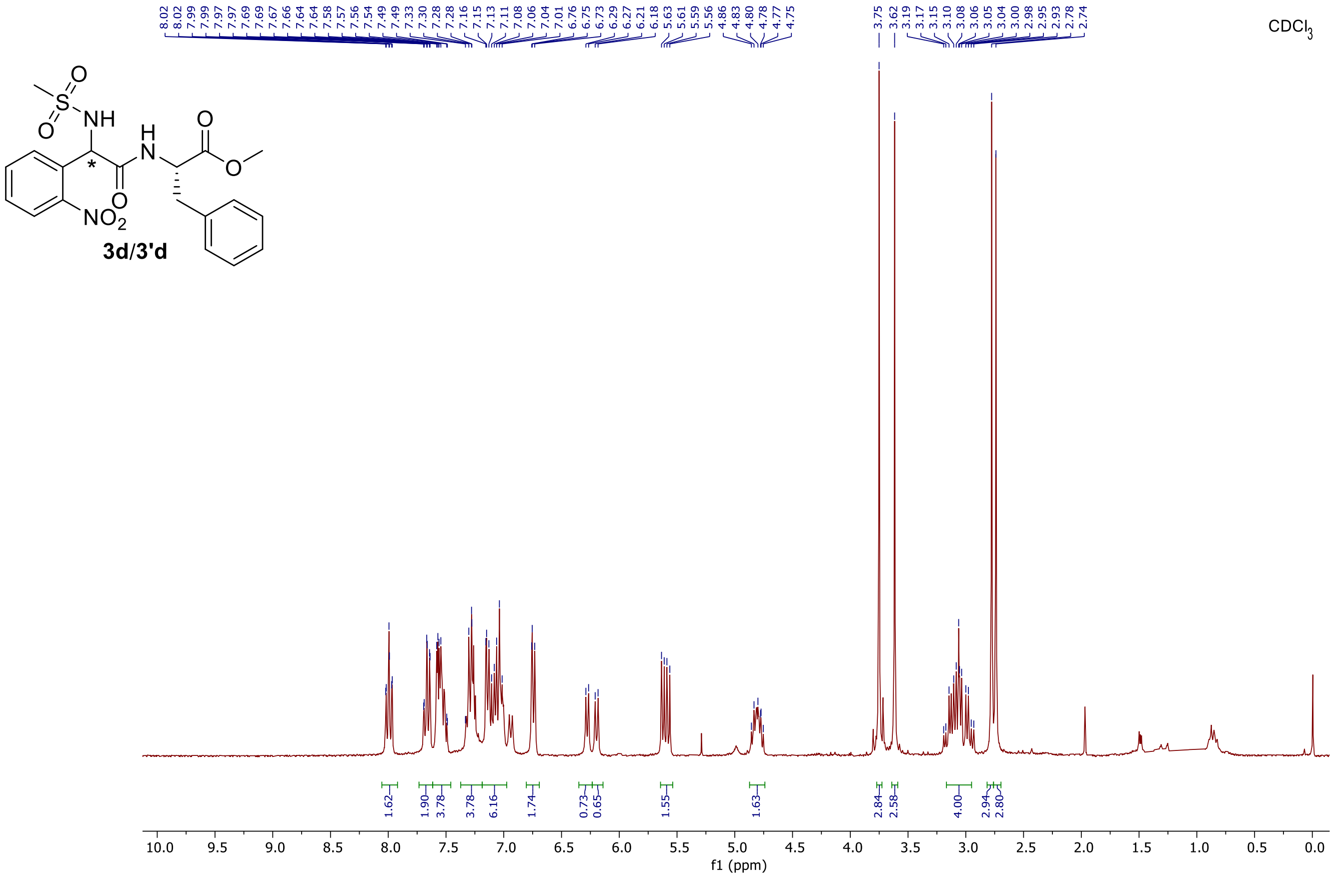
8.02 8.02 7.99 7.97 7.97 7.69 7.69 7.67 7.66 7.64 7.64 7.58 7.57 7.56 7.54 7.49 7.33 7.30 7.28 7.16 7.15 7.13 7.11 7.08 7.06 7.04 7.01 6.76 6.75 6.73 6.29 6.27 6.21 6.18 5.63 5.61 5.59 5.56 4.86 4.83 4.80 4.78 4.77 4.75
3.75 3.62 3.19 3.17 3.15 3.10 3.08 3.06 3.05 3.04 3.00 2.98 2.95 2.93 2.78 2.74
CDCl3
3d/3'd
1.62 1.90 3.78 3.78 6.16 1.74 0.73 0.65 1.55 1.63 2.84 2.58 4.00 2.94 2.80
f1 (ppm)

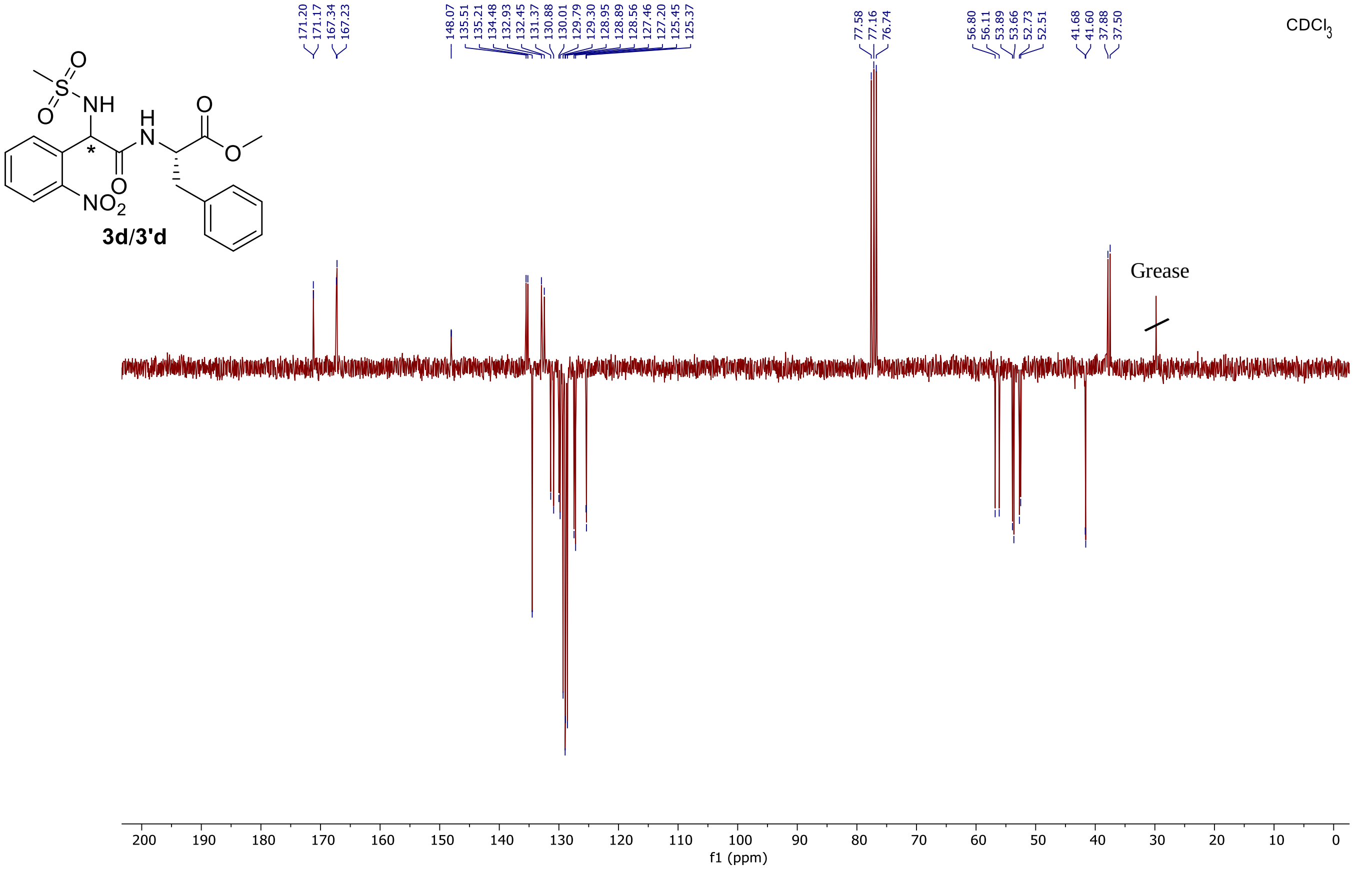
171.20 171.17 167.34 167.23
148.07 135.51 135.21 134.48 132.93 132.45 131.37 130.88 130.01 129.79 129.30 128.95 128.89 128.56 127.46 127.20 125.45 125.37
77.58 77.16 76.74
56.80 56.11 53.89 53.66 52.73 52.51
41.68 41.60 37.88 37.50
CDCl3
3d/3'd
Grease
f1 (ppm)

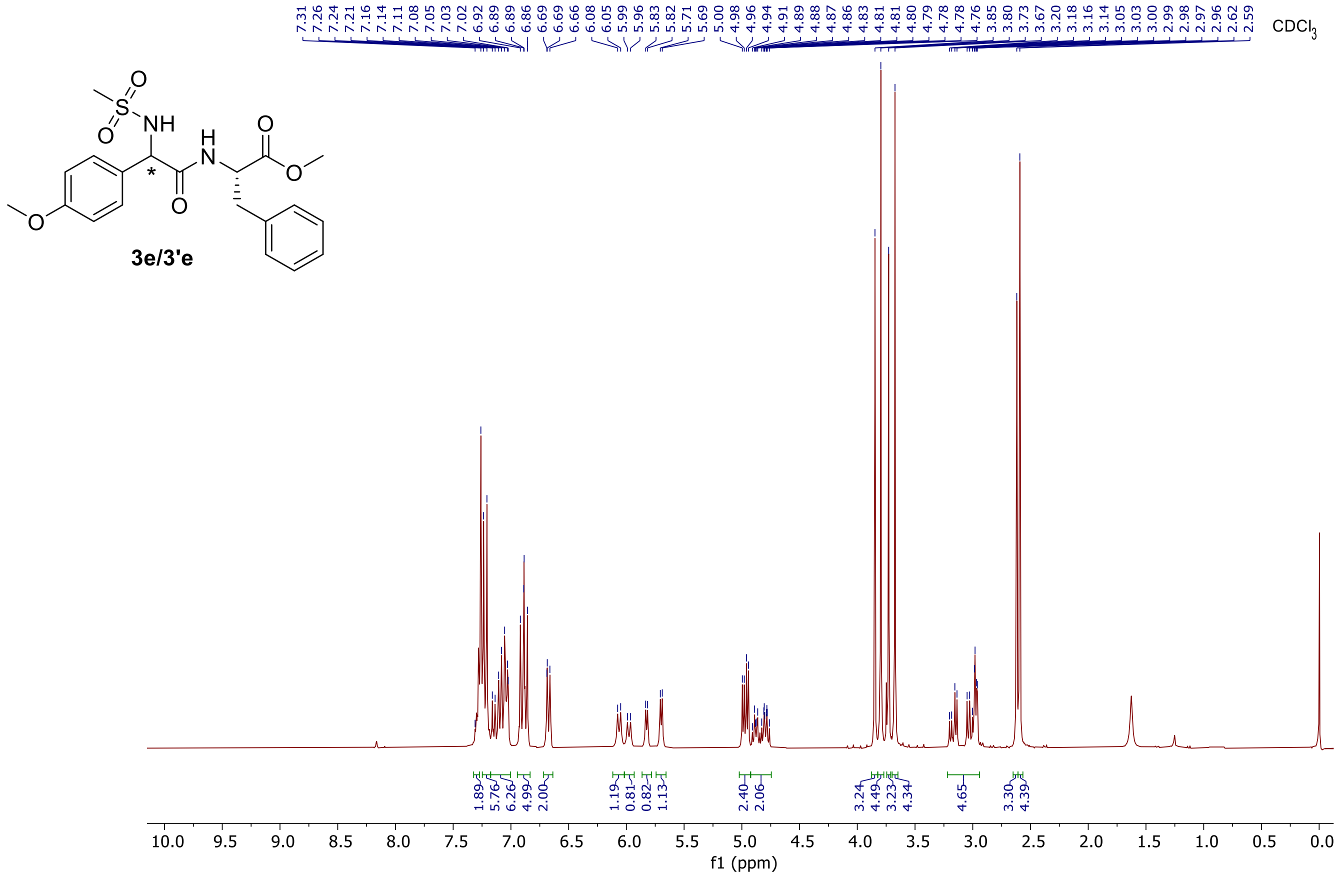
7.31 7.26 7.24 7.21 7.16 7.14 7.11 7.08 7.05 7.03 7.02 6.92 6.89 6.89 6.86 6.69 6.69 6.66 6.08 6.05 5.99 5.96 5.83 5.82 5.71 5.69 5.00 4.98 4.96 4.94 4.91 4.89 4.88 4.87 4.86 4.83 4.81 4.81 4.80 4.79 4.78 4.76 3.85 3.80 3.73 3.67 3.20 3.18 3.16 3.14 3.05 3.03 3.00 2.99 2.98 2.97 2.96 2.62 2.59
CDCl3
3e/3'e
1.89 5.76 6.26 4.99 2.00 1.19 0.81 0.82 1.13 2.40 2.06 3.24 4.49 3.23 4.34 4.65 3.30 4.39
f1 (ppm)

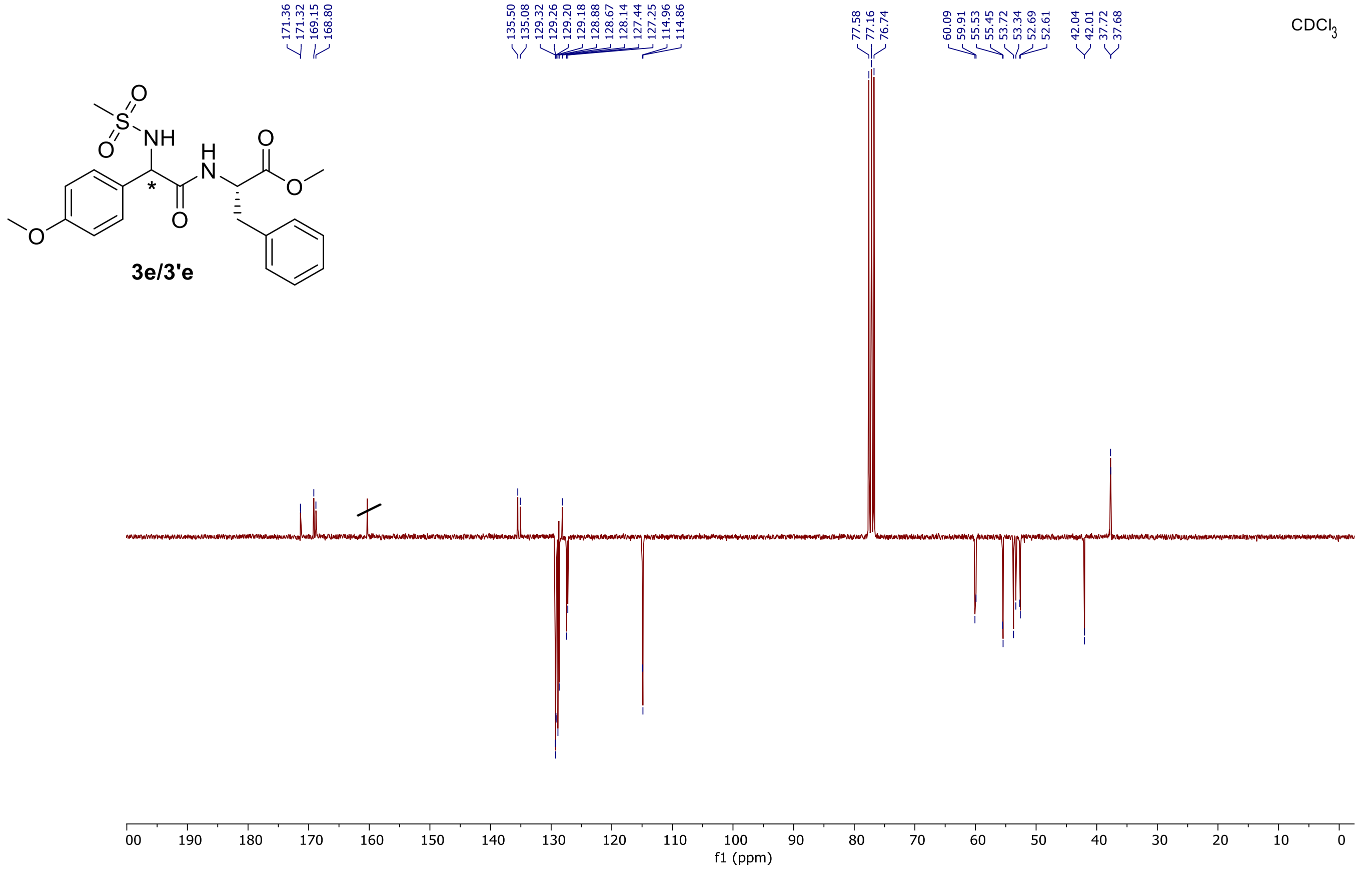
171.36 171.32 169.15 168.80 135.50 135.08 129.32 129.26 129.20 129.18 128.88 128.67 128.14 127.44 127.25 114.96 114.86 77.58 77.16 76.74 60.09 59.91 55.53 55.45 53.72 53.34 52.69 52.61 42.04 42.01 37.72 37.68
CDCl3
3e/3'e
f1 (ppm)

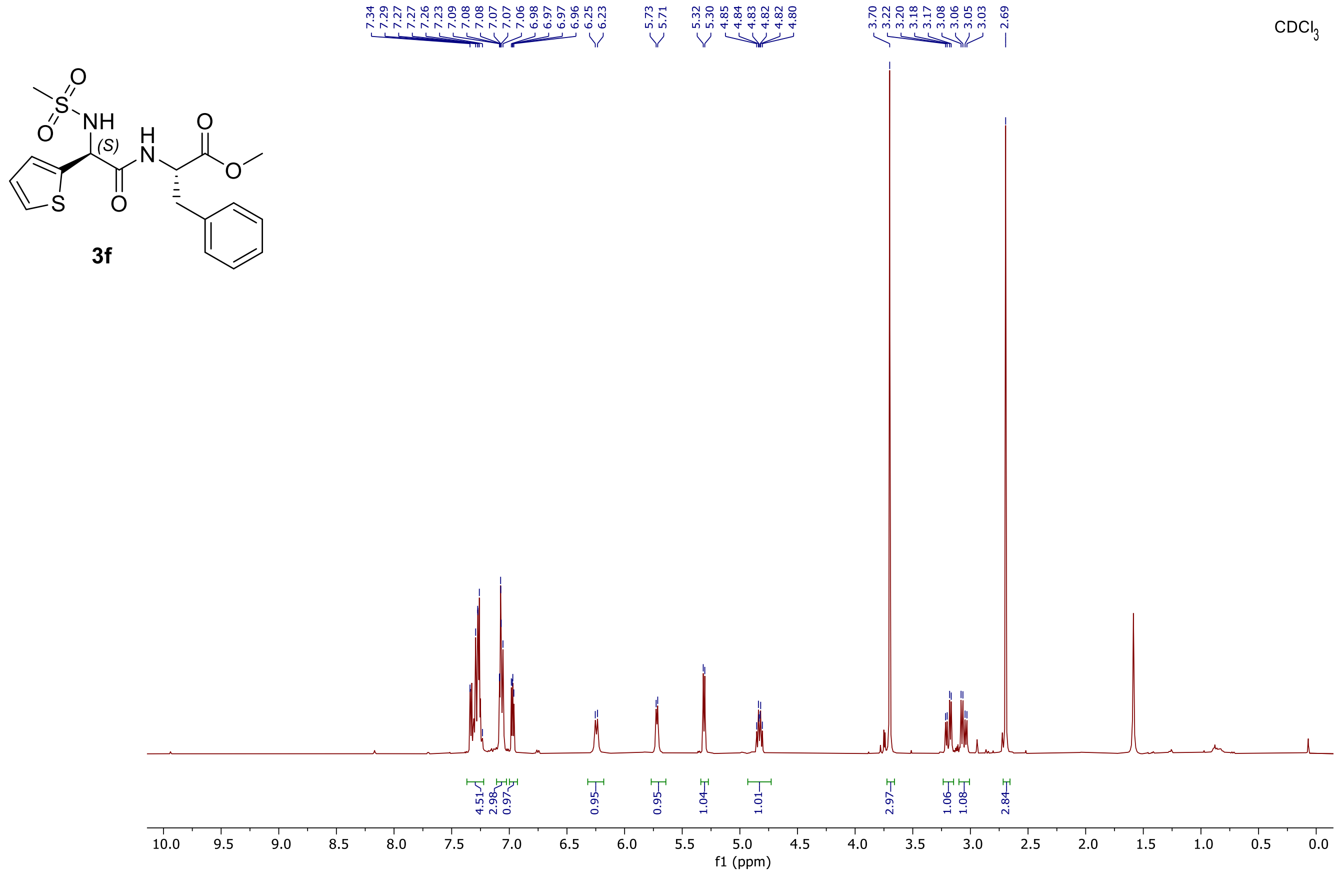

3f
7.34
7.29
7.27
7.27
7.26
7.23
7.09
7.08
7.08
7.07
7.07
7.06
6.98
6.97
6.97
6.96
6.25
6.23
5.73
5.71
5.32
5.30
4.85
4.84
4.83
4.82
4.82
4.80
3.70
3.22
3.20
3.18
3.17
3.08
3.06
3.05
3.03
2.69
CDCl3
4.51
2.98
0.97
0.95
0.95
1.04
1.01
2.97
1.06
1.08
2.84
f1 (ppm)

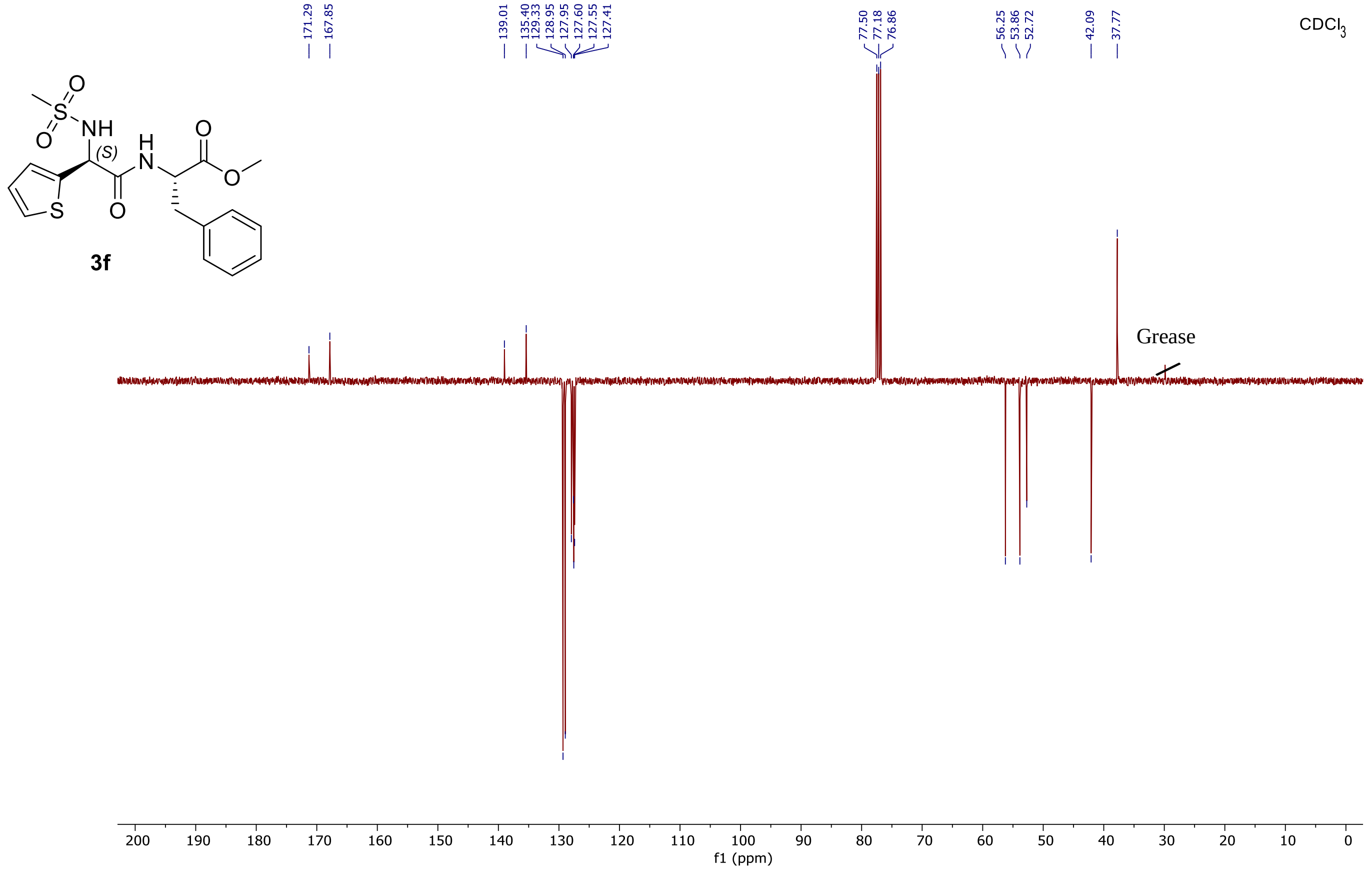

3f
171.29
167.85
139.01
135.40
129.33
128.95
127.95
127.60
127.55
127.41
77.50
77.18
76.86
56.25
53.86
52.72
42.09
37.77
CDCl3
Grease
f1 (ppm)

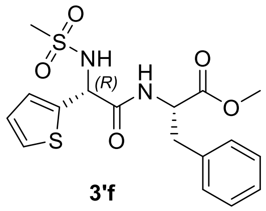

3'f

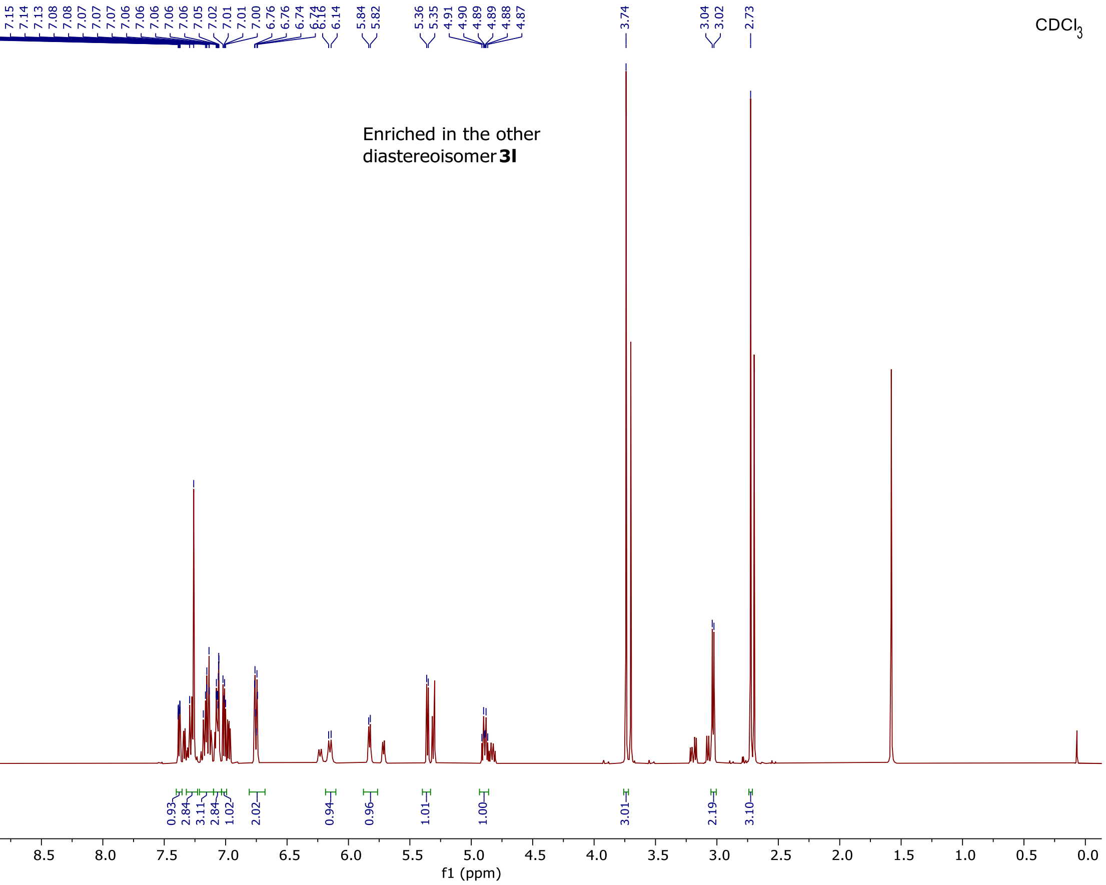

CDCl3
Enriched in the other diastereoisomer 3l
7.39 7.38 7.38 7.37 7.37 7.37 7.37 7.29 7.26 7.16 7.15 7.15 7.14 7.13 7.08 7.08 7.07 7.07 7.06 7.06 7.06 7.06 7.05 7.02 7.01 7.01 7.00 6.76 6.76 6.74 6.74 6.16 6.14 5.84 5.82 5.36 5.35 4.91 4.90 4.89 4.89 4.88 4.87 3.74 3.04 3.02 2.73
0.93 2.84 3.11 2.84 1.02 2.02 0.94 0.96 1.01 1.00 3.01 2.19 3.10
10.0 9.5 9.0 8.5 8.0 7.5 7.0 6.5 6.0 5.5 5.0 4.5 4.0 3.5 3.0 2.5 2.0 1.5 1.0 0.5 0.0
f1 (ppm)

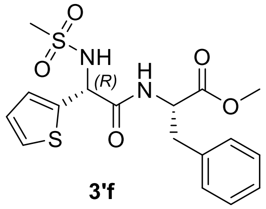

3'f

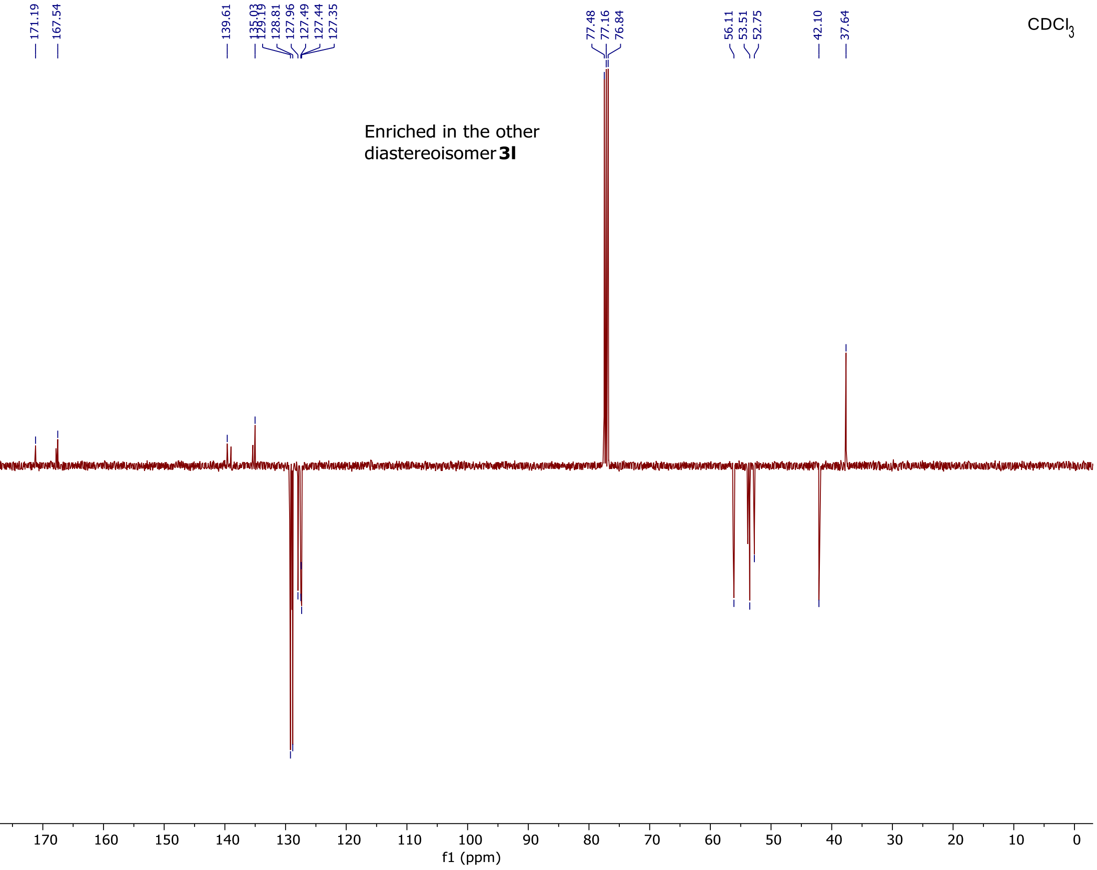

CDCl3
Enriched in the other diastereoisomer 3l
171.19 167.54 139.61 128.81 127.96 127.49 127.44 127.35 77.48 77.16 76.84 56.11 53.51 52.75 42.10 37.64
200 190 180 170 160 150 140 130 120 110 100 90 80 70 60 50 40 30 20 10 0
f1 (ppm)

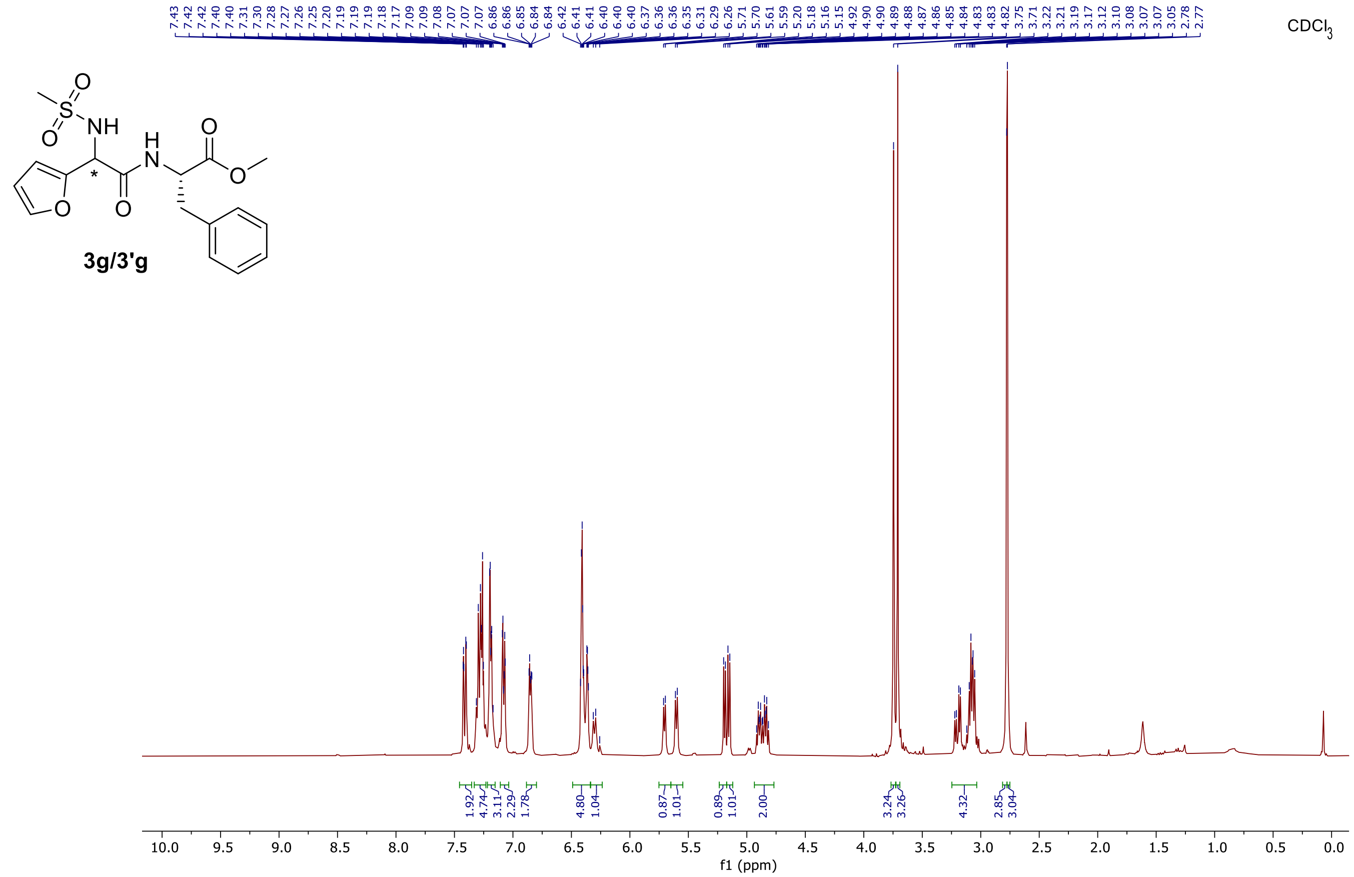
CDCl3
3g/3'g
f1 (ppm)

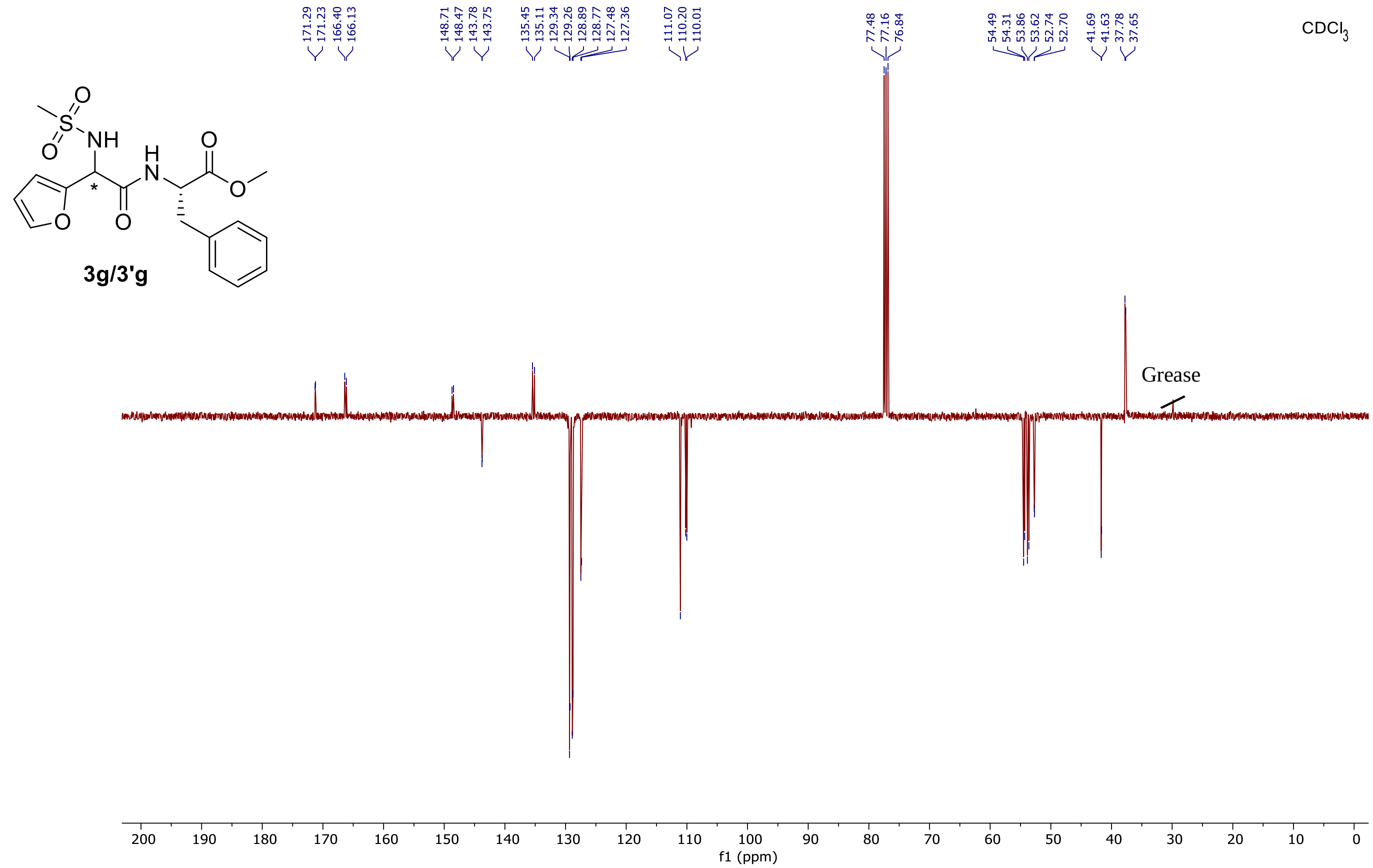
CDCl3
3g/3'g
Grease
f1 (ppm)

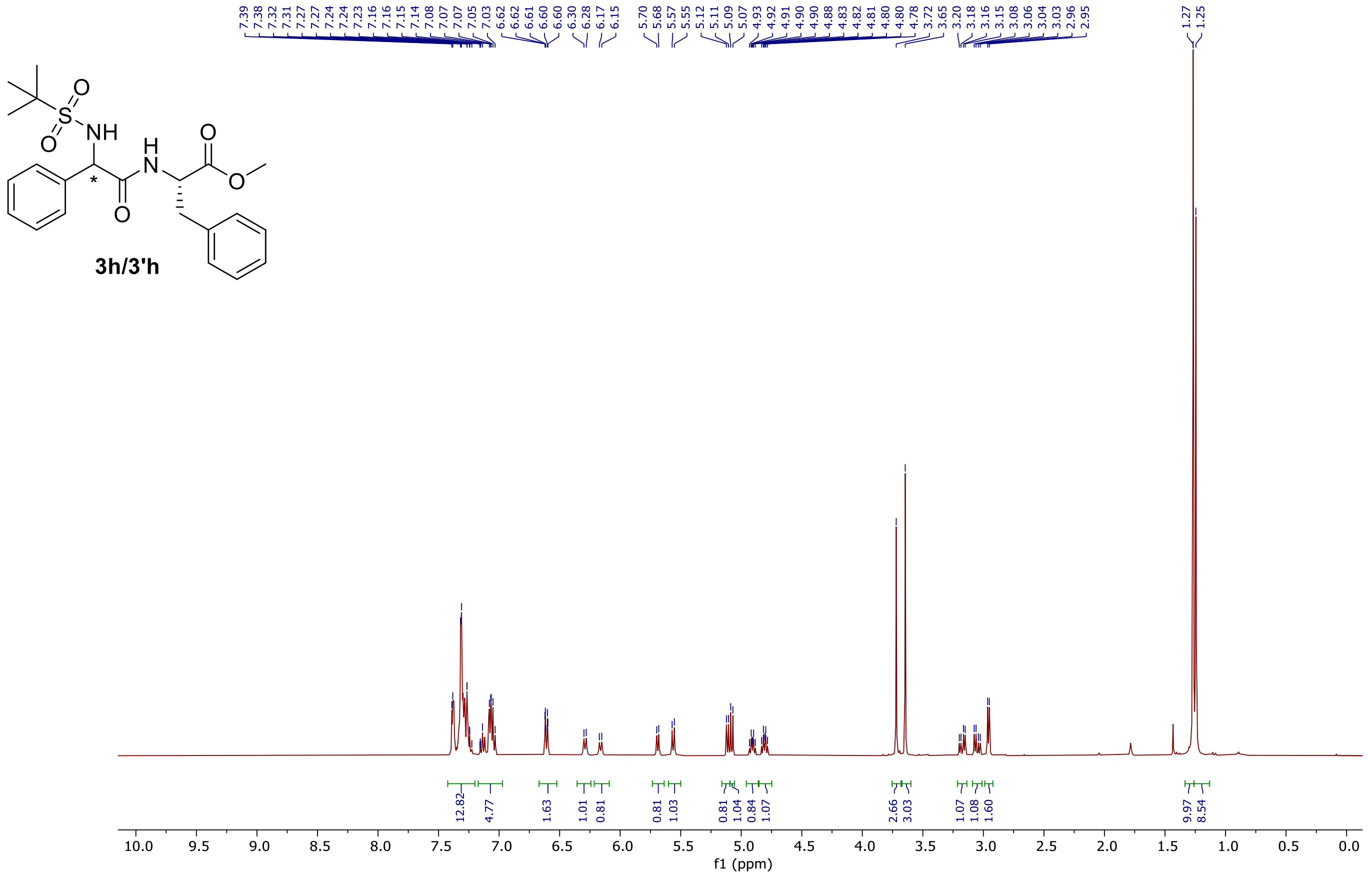
3h/3'h
f1 (ppm)

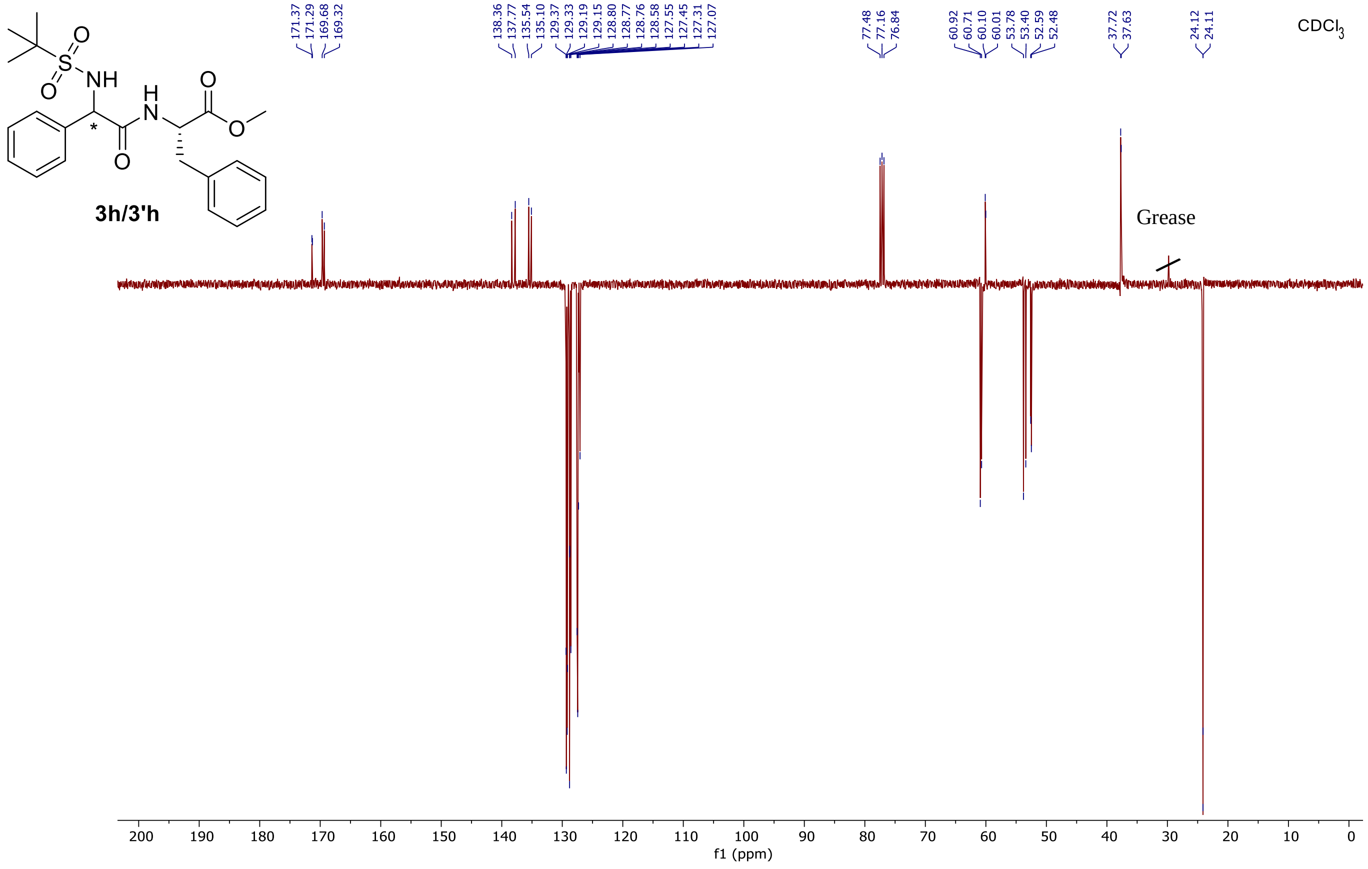
3h/3'h
CDCl3
Grease
f1 (ppm)

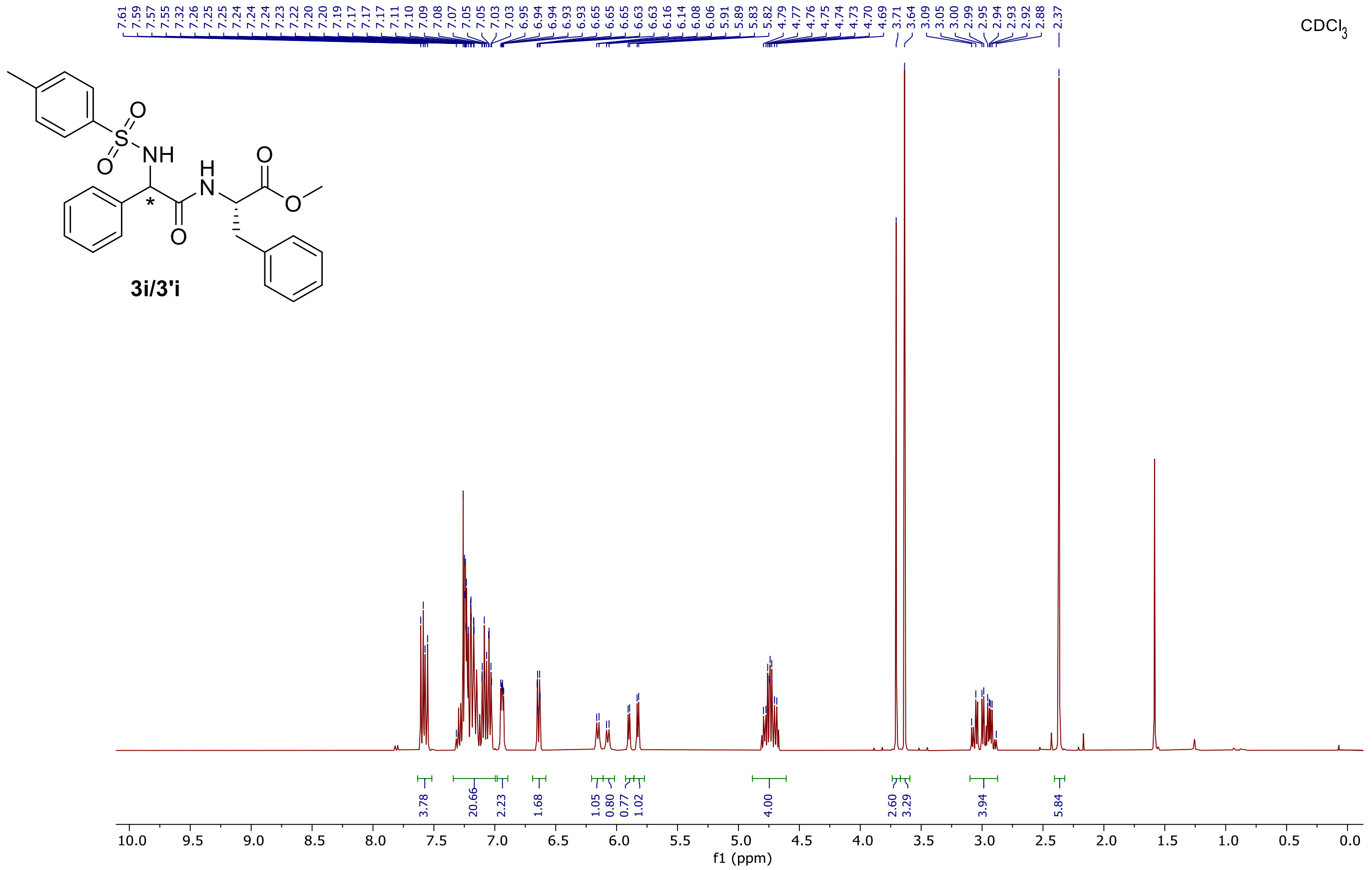

3i/3'i

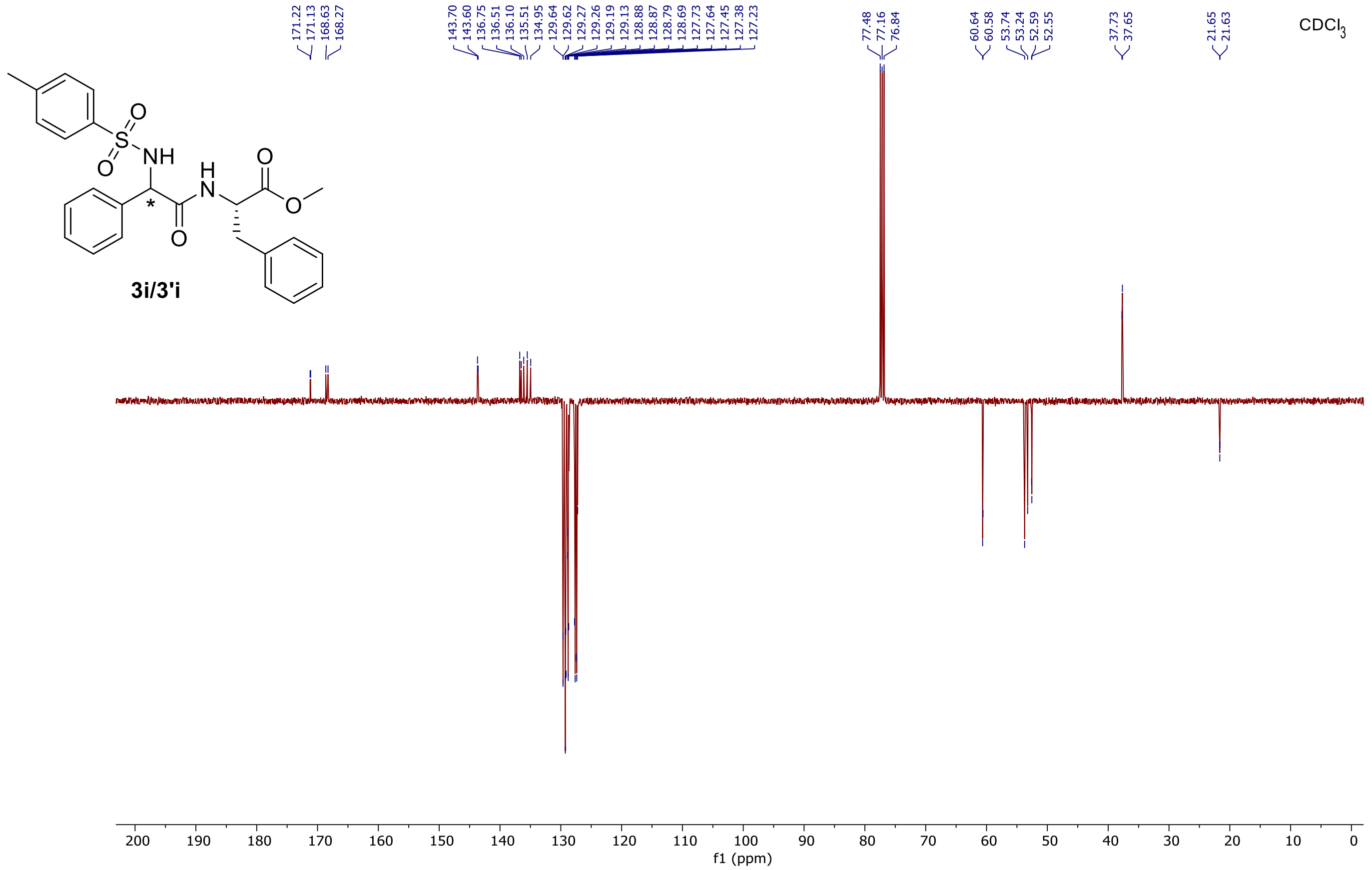

3i/3'i

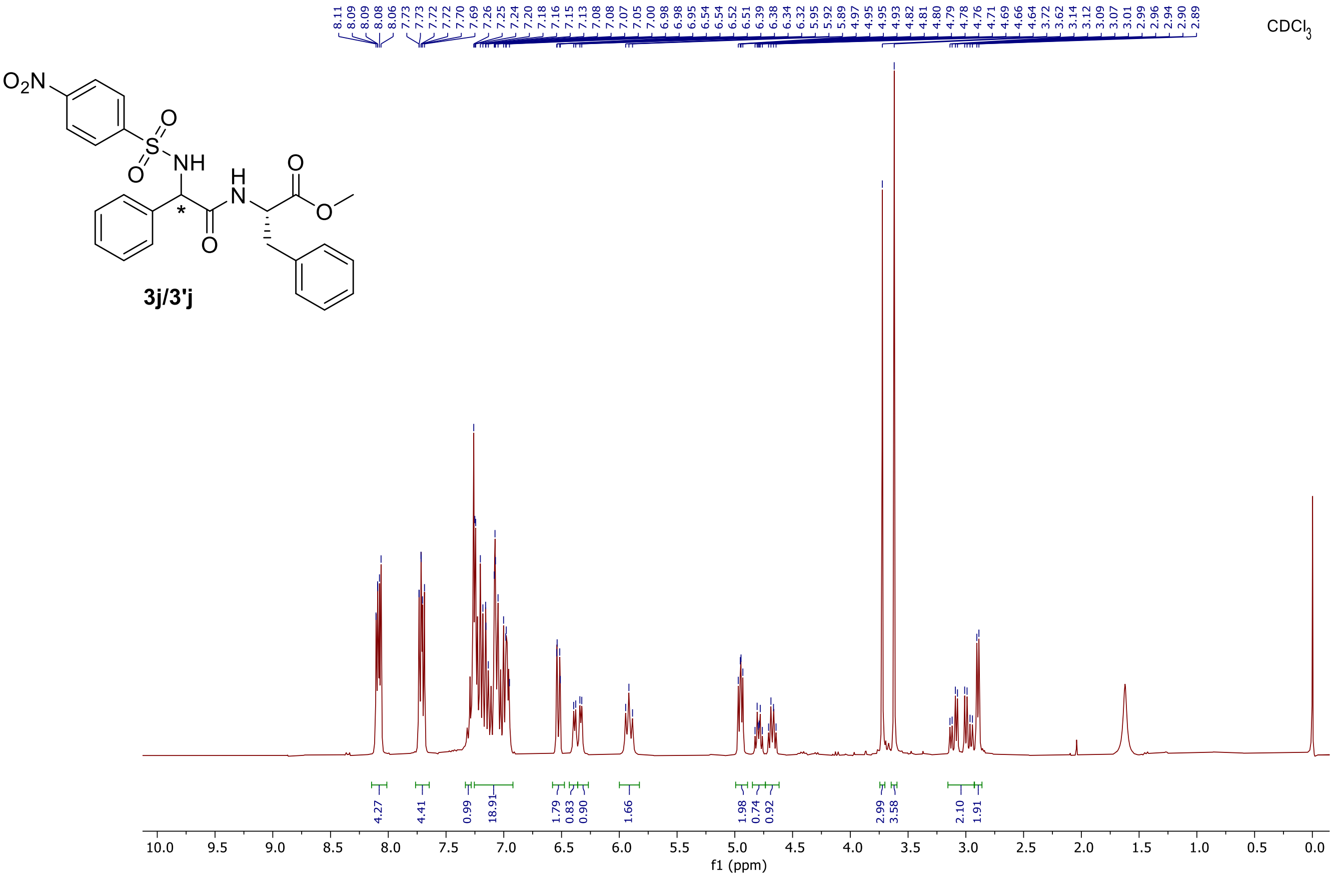

3j/3'j
8.11 8.09 8.08 8.06 7.73 7.73 7.72 7.72 7.70 7.69 7.26 7.25 7.24 7.20 7.18 7.16 7.15 7.13 7.08 7.08 7.07 7.05 7.00 6.98 6.98 6.95 6.54 6.52 6.51 6.39 6.38 6.34 6.32 5.95 5.92 5.89 4.97 4.95 4.95 4.93 4.82 4.81 4.80 4.79 4.78 4.76 4.71 4.69 4.66 4.64 3.72 3.62 3.14 3.12 3.09 3.07 3.01 2.99 2.96 2.94 2.90 2.89
CDCl3
4.27 4.41 0.99 18.91 1.79 0.83 0.90 1.66 1.98 0.74 0.92 2.99 3.58 2.10 1.91
10.0 9.5 9.0 8.5 8.0 7.5 7.0 6.5 6.0 5.5 5.0 4.5 4.0 3.5 3.0 2.5 2.0 1.5 1.0 0.5 0.0
f1 (ppm)

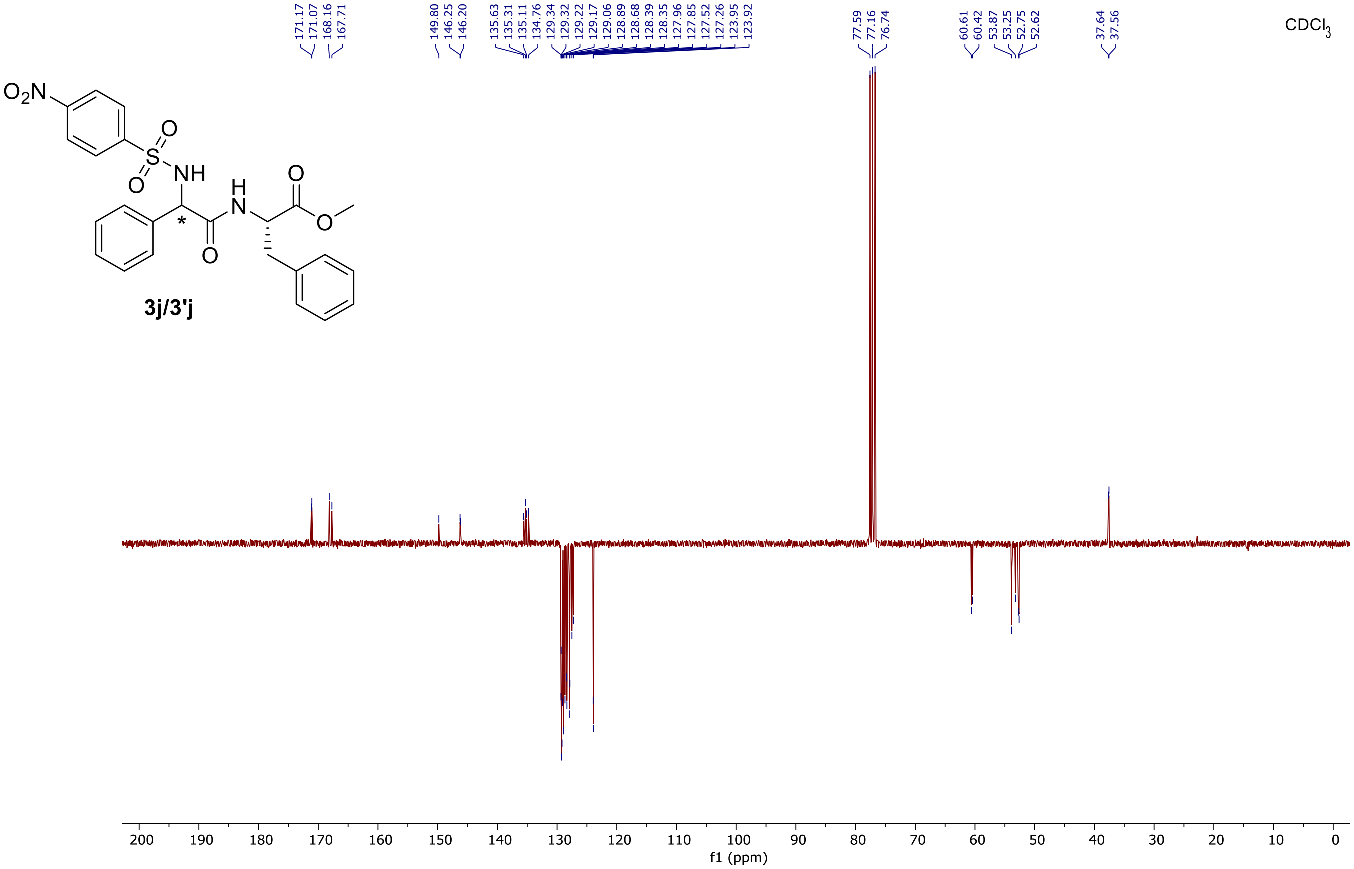

3j/3'j
171.17 171.07 168.16 167.71 149.80 146.25 146.20 135.63 135.31 135.11 134.76 129.34 129.32 129.22 129.17 129.06 128.89 128.68 128.39 128.35 127.96 127.85 127.52 127.26 123.95 123.92 77.59 77.16 76.74 60.61 60.42 53.87 53.25 52.75 52.62 37.64 37.56
CDCl3
200 190 180 170 160 150 140 130 120 110 100 90 80 70 60 50 40 30 20 10 0
f1 (ppm)

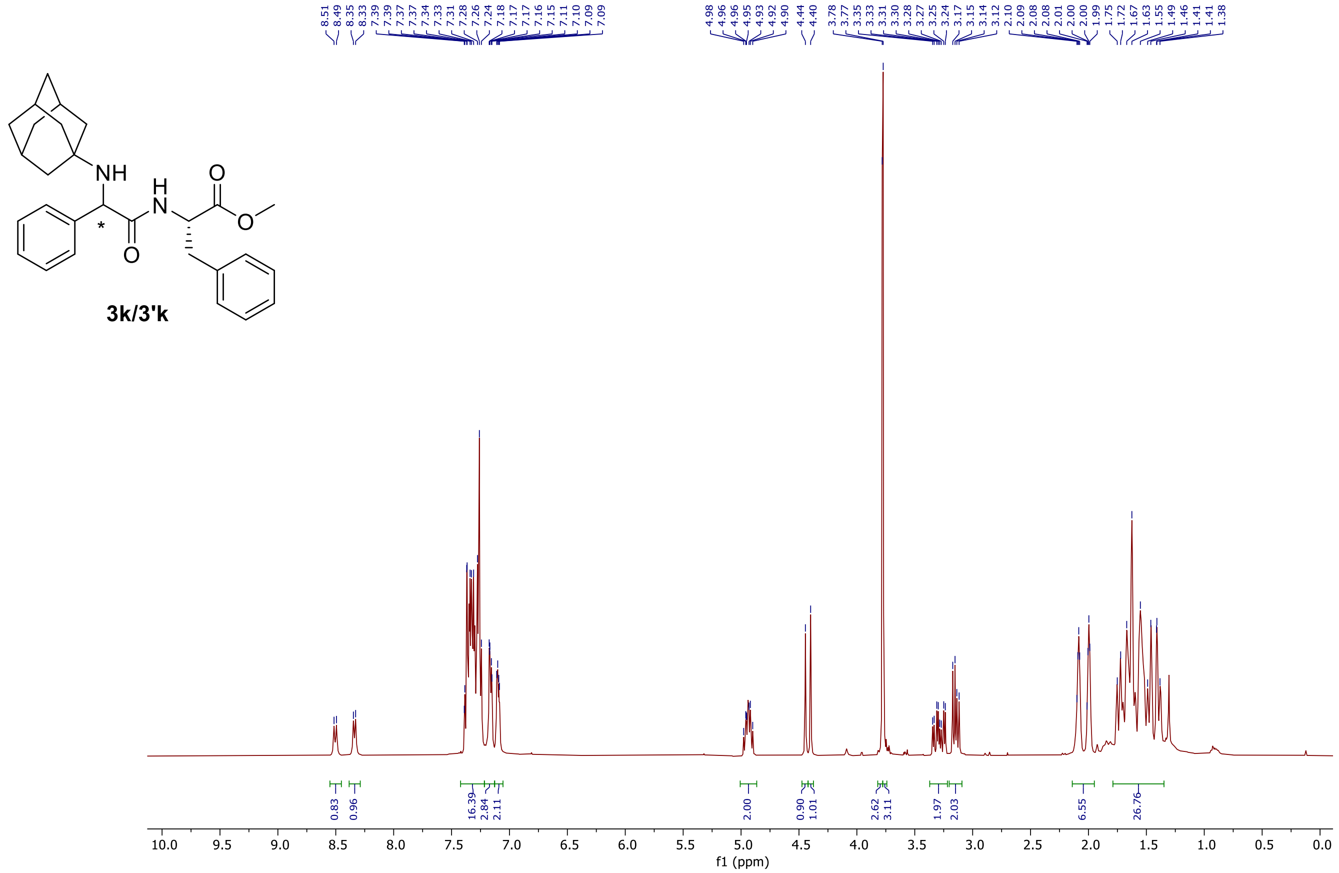
3k/3'k
NH
H
N
O
O
O
*
8.51 8.49 8.35 8.33 7.39 7.39 7.37 7.37 7.34 7.33 7.31 7.28 7.26 7.24 7.18 7.17 7.17 7.16 7.15 7.11 7.10 7.09 7.09
4.98 4.96 4.96 4.95 4.93 4.92 4.90 4.44 4.40 3.78 3.77 3.35 3.33 3.31 3.30 3.28 3.27 3.25 3.24 3.17 3.15 3.14 3.12 2.10 2.09 2.08 2.08 2.01 2.00 2.00 1.99 1.75 1.72 1.67 1.63 1.55 1.49 1.46 1.41 1.38
0.83 0.96 16.39 2.84 2.11 2.00 0.90 1.01 2.62 3.11 1.97 2.03 6.55 26.76
f1 (ppm)

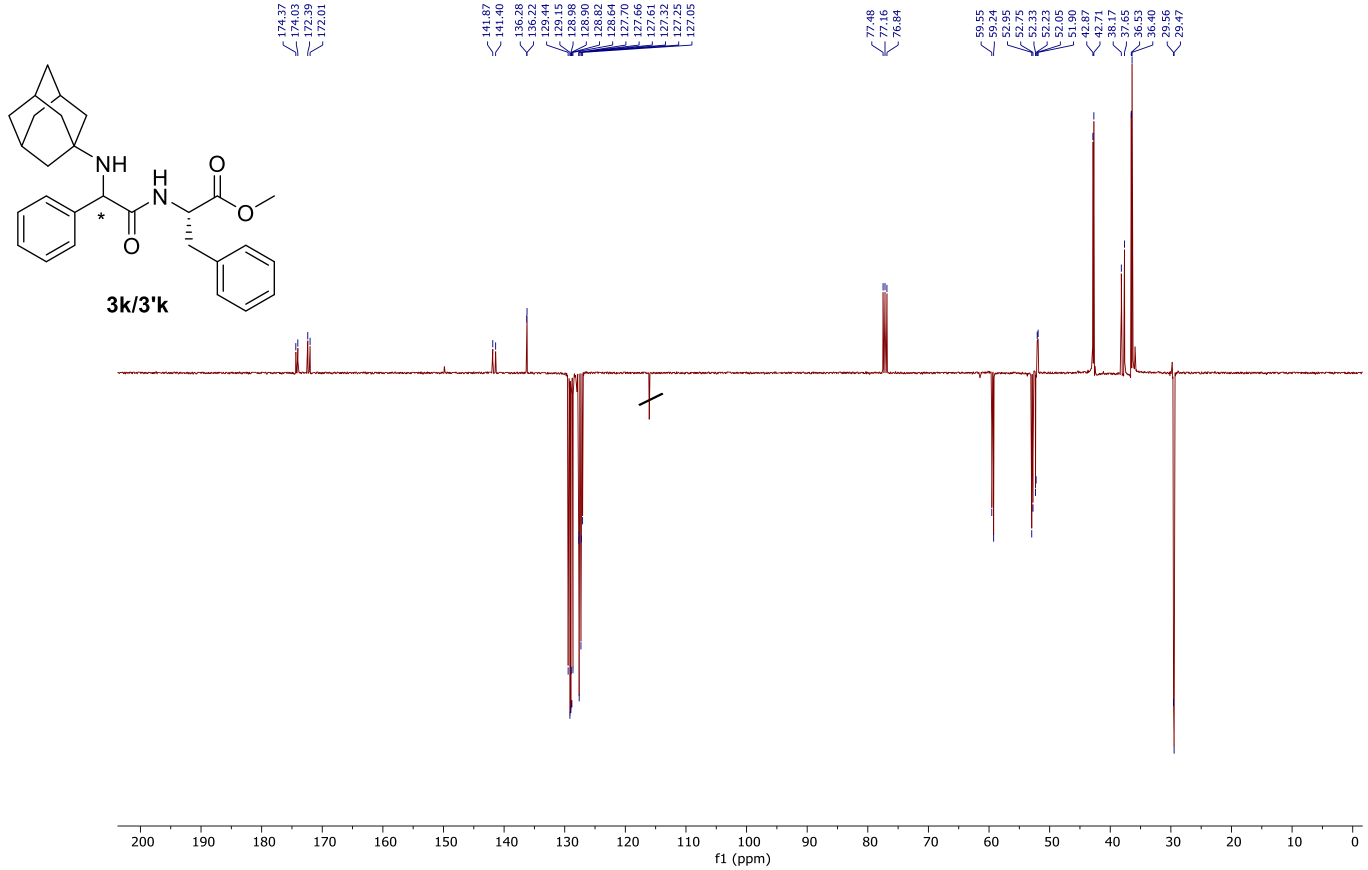
3k/3'k
NH
H
N
O
O
O
*
174.37 174.03 172.39 172.01
141.87 141.40 136.28 136.22 129.44 129.15 128.98 128.90 128.82 128.64 127.70 127.66 127.61 127.32 127.25 127.05
77.48 77.16 76.84
59.55 59.24 52.95 52.75 52.33 52.23 52.05 51.90 42.87 42.71 38.17 37.65 36.53 36.40 29.56 29.47
f1 (ppm)

3l/3'l

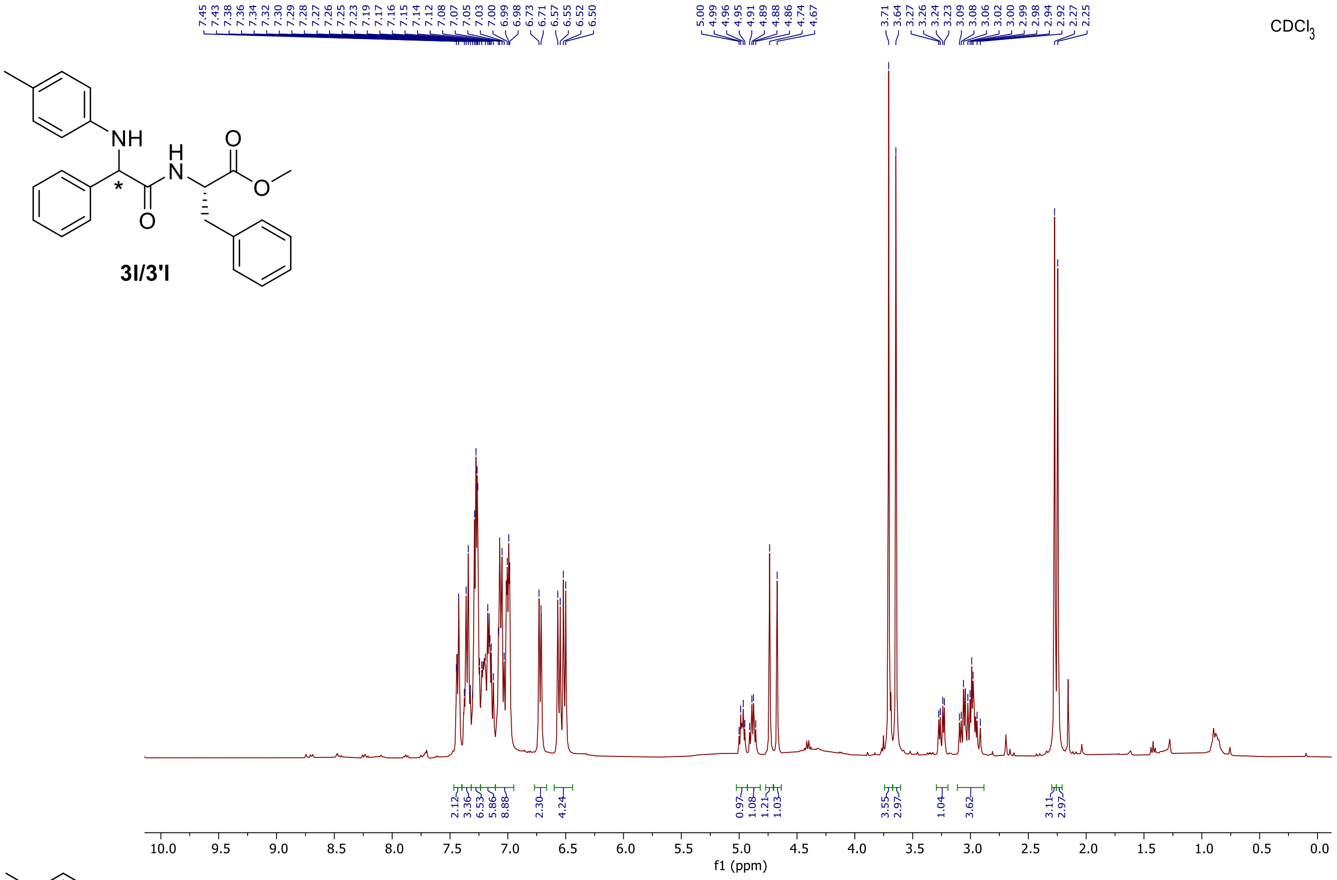
7.45 7.43 7.38 7.36 7.34 7.32 7.30 7.29 7.28 7.27 7.26 7.25 7.23 7.19 7.17 7.16 7.15 7.14 7.12 7.08 7.07 7.05 7.03 7.00 6.99 6.98 6.73 6.71 6.57 6.55 6.52 6.50
5.00 4.99 4.96 4.95 4.91 4.89 4.88 4.86 4.74 4.67
3.71 3.64 3.27 3.26 3.24 3.23 3.09 3.08 3.06 3.02 3.00 2.99 2.98 2.94 2.92 2.27 2.25
CDCl$_3$
2.12 3.36 6.53 5.86 8.88 2.30 4.24 0.97 1.08 1.21 1.03 3.55 2.97 1.04 3.62 3.11 2.97
10.0 9.5 9.0 8.5 8.0 7.5 7.0 6.5 6.0 5.5 5.0 4.5 4.0 3.5 3.0 2.5 2.0 1.5 1.0 0.5 0.0
f1 (ppm)

3l/3'l

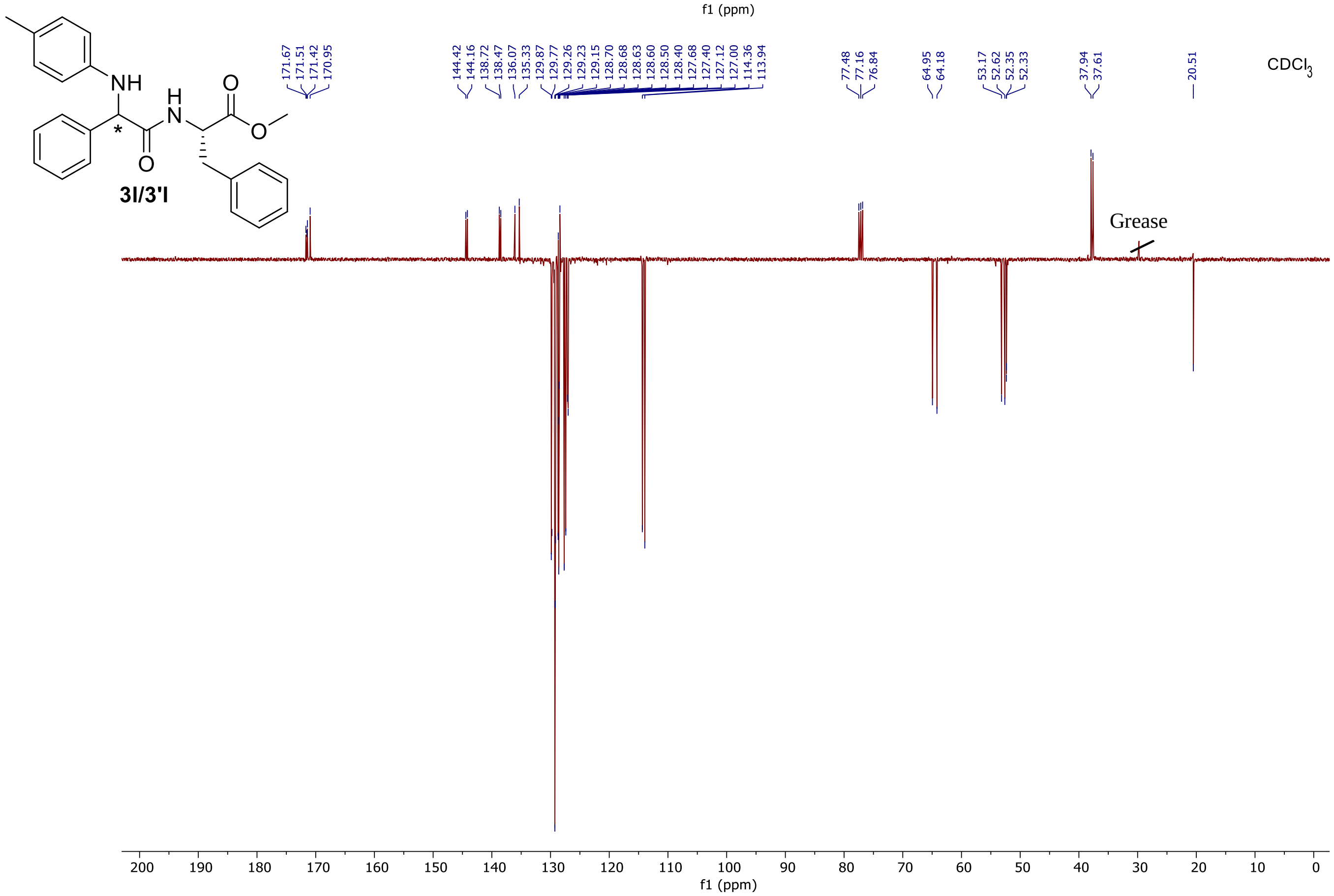
171.67 171.51 171.42 170.95
144.42 144.16 138.72 138.47 136.07 135.33 129.87 129.77 129.26 129.23 129.15 128.70 128.68 128.63 128.60 128.50 128.40 127.68 127.40 127.12 127.00 114.36 113.94
77.48 77.16 76.84
64.95 64.18
53.17 52.62 52.35 52.33
37.94 37.61
20.51
CDCl$_3$
Grease
200 190 180 170 160 150 140 130 120 110 100 90 80 70 60 50 40 30 20 10 0
f1 (ppm)

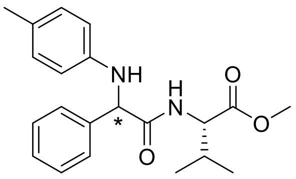

3m/3'm

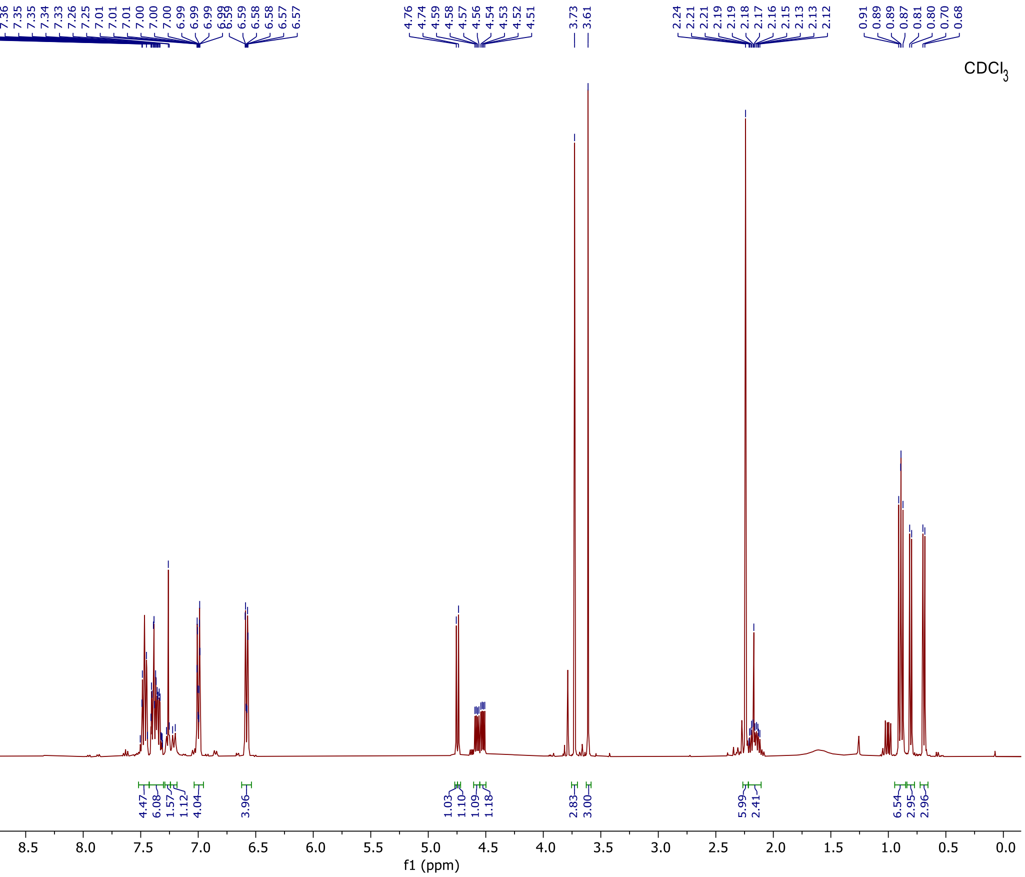
7.49 7.49 7.45 7.41 7.41 7.40 7.39 7.38 7.38 7.37 7.37 7.36 7.35 7.35 7.34 7.33 7.26 7.25 7.01 7.01 7.01 7.00 7.00 7.00 6.99 6.99 6.99 6.59 6.59 6.58 6.58 6.57 6.57
4.76 4.74 4.59 4.58 4.57 4.56 4.54 4.53 4.52 4.51
3.73 3.61
2.24 2.21 2.21 2.19 2.19 2.18 2.17 2.16 2.15 2.13 2.13 2.12
0.91 0.89 0.89 0.87 0.81 0.80 0.70 0.68
CDCl3
4.47 6.08 1.57 1.12 4.04 3.96 1.03 1.10 1.09 1.18 2.83 3.00 5.99 2.41 6.54 2.95 2.96
f1 (ppm)

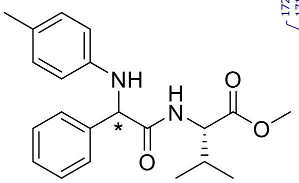

3m/3'm

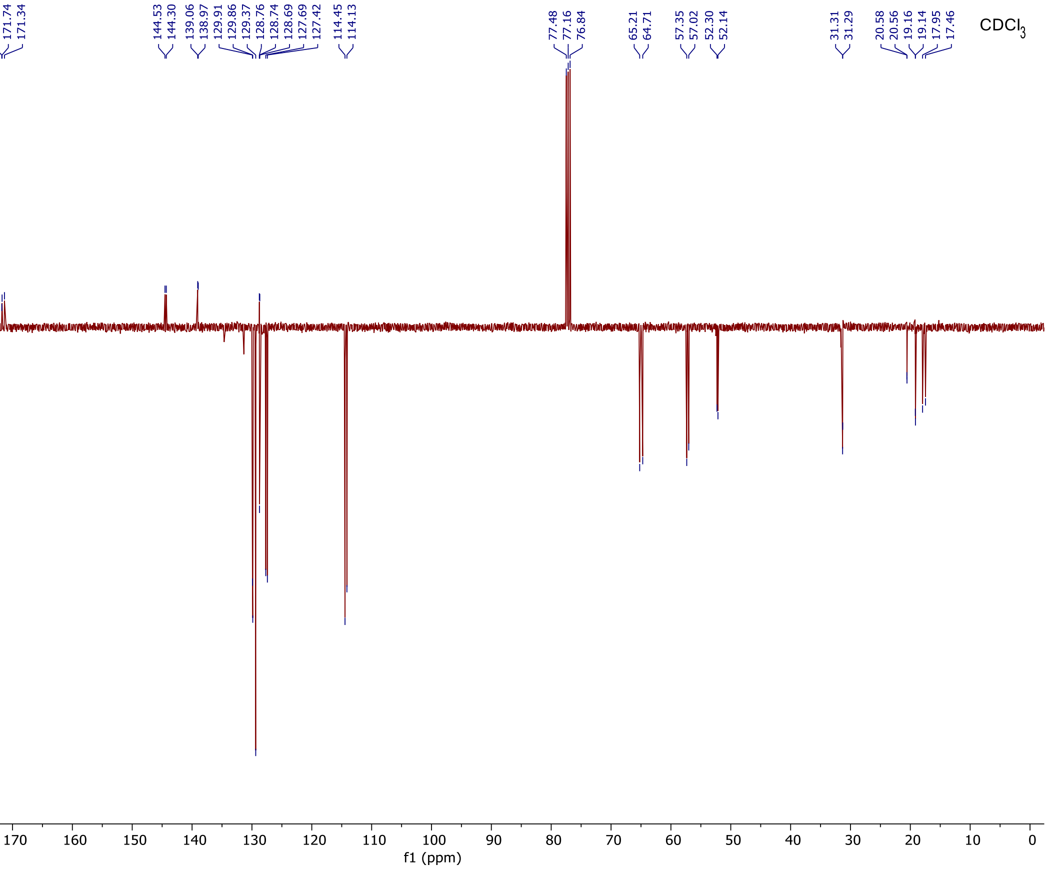
172.40 171.79 171.74 171.34
144.53 144.30 139.06 138.97 129.91 129.86 129.37 128.76 128.74 128.69 127.69 127.42 114.45 114.13
77.48 77.16 76.84
65.21 64.71 57.35 57.02 52.30 52.14
31.31 31.29
20.58 20.56 19.16 19.14 17.95 17.46
CDCl3
f1 (ppm)

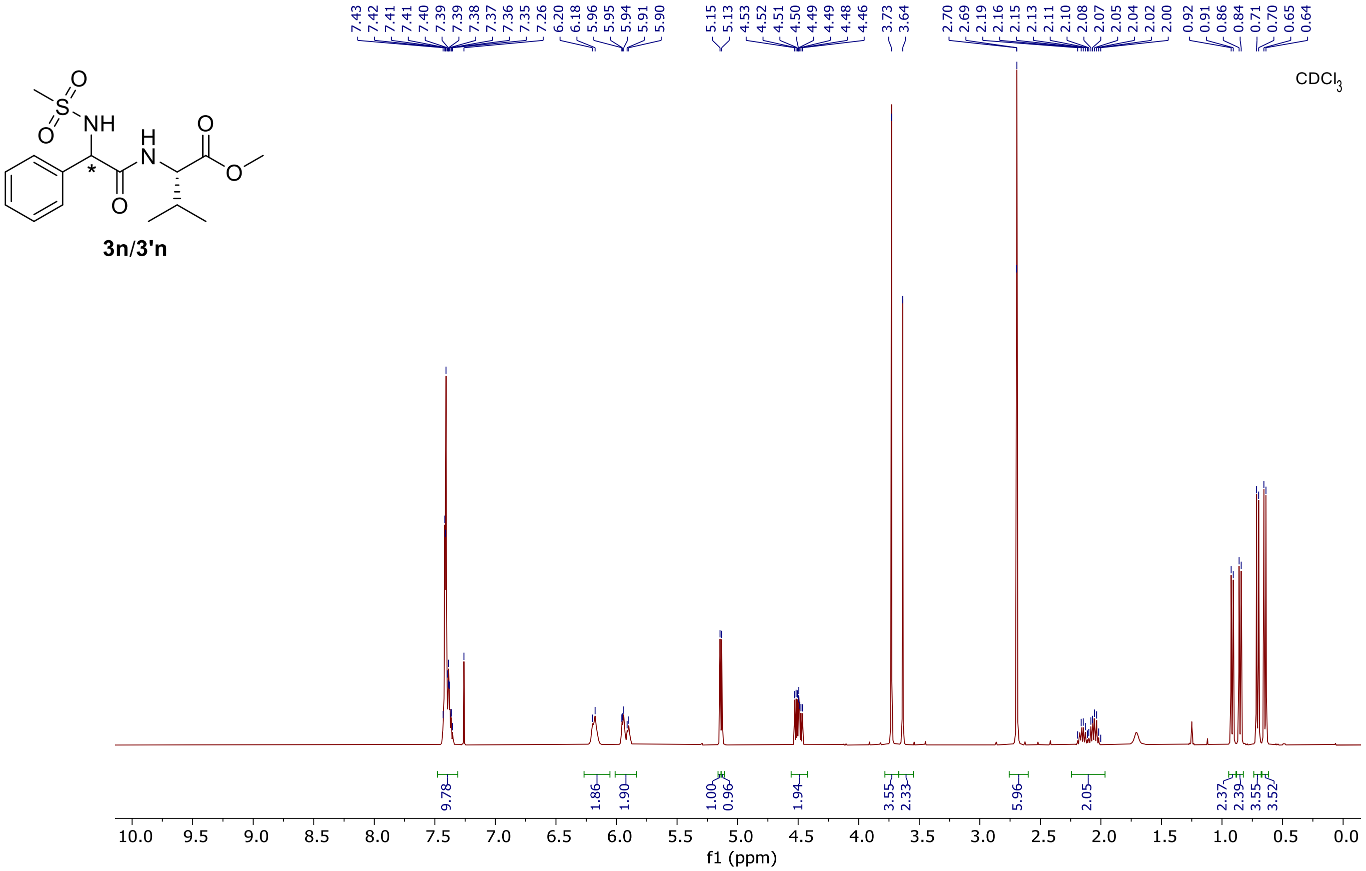

3n/3'n

7.43 7.42 7.41 7.41 7.40 7.39 7.39 7.38 7.37 7.36 7.35 7.26 6.20 6.18 5.96 5.95 5.94 5.91 5.90
5.15 5.13 4.53 4.52 4.51 4.50 4.49 4.49 4.48 4.46 3.73 3.64
2.70 2.69 2.19 2.16 2.15 2.13 2.11 2.10 2.08 2.07 2.05 2.04 2.02 2.00 0.92 0.91 0.86 0.84 0.71 0.70 0.65 0.64
CDCl3
9.78 1.86 1.90 1.00 0.96 1.94 3.55 2.33 5.96 2.05 2.37 2.39 3.55 3.52
10.0 9.5 9.0 8.5 8.0 7.5 7.0 6.5 6.0 5.5 5.0 4.5 4.0 3.5 3.0 2.5 2.0 1.5 1.0 0.5 0.0
f1 (ppm)

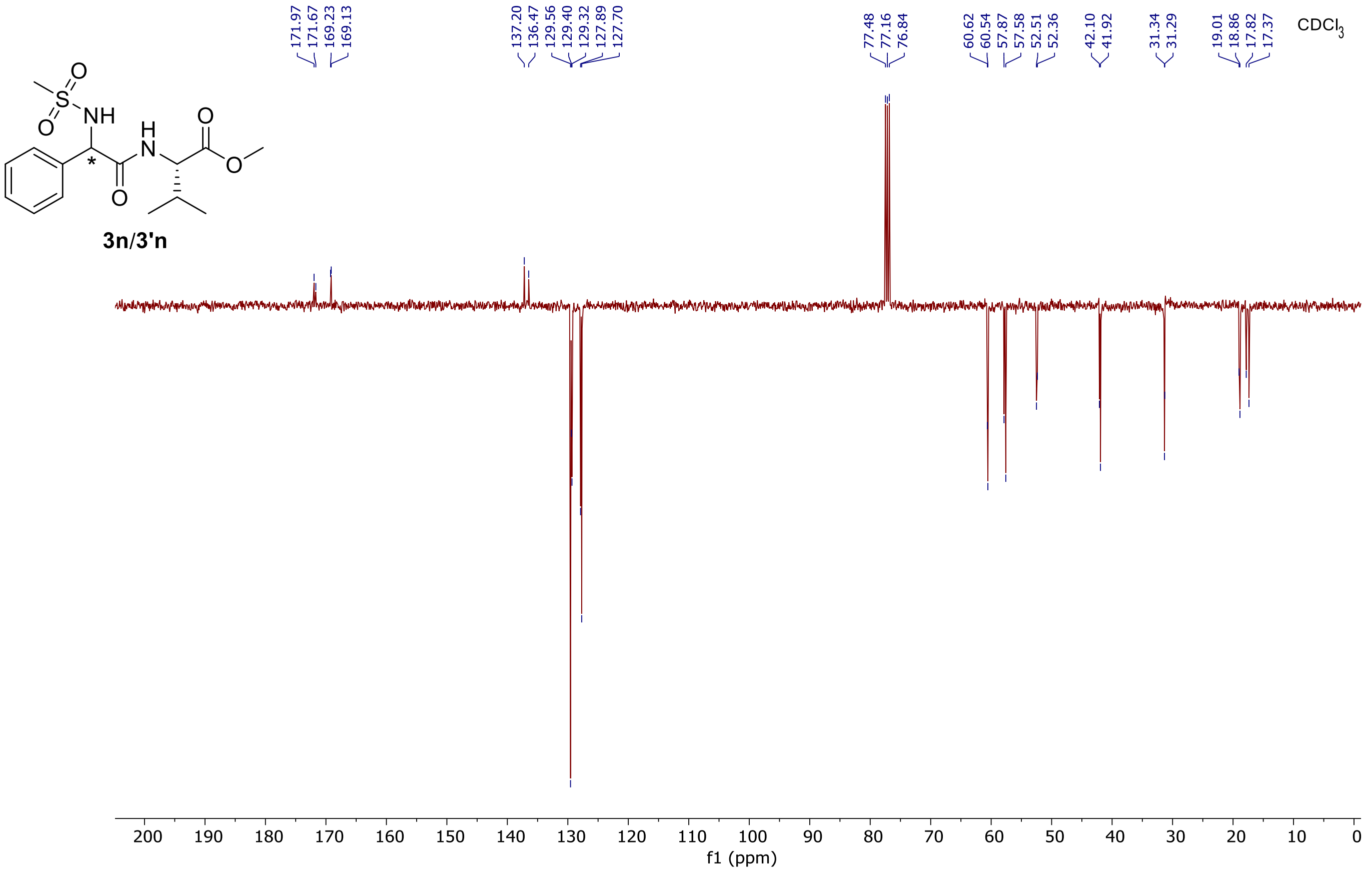

3n/3'n

171.97 171.67 169.23 169.13
137.20 136.47 129.56 129.40 129.32 127.89 127.70
77.48 77.16 76.84
60.62 60.54 57.87 57.58 52.51 52.36 42.10 41.92 31.34 31.29 19.01 18.86 17.82 17.37
CDCl3
200 190 180 170 160 150 140 130 120 110 100 90 80 70 60 50 40 30 20 10 0
f1 (ppm)

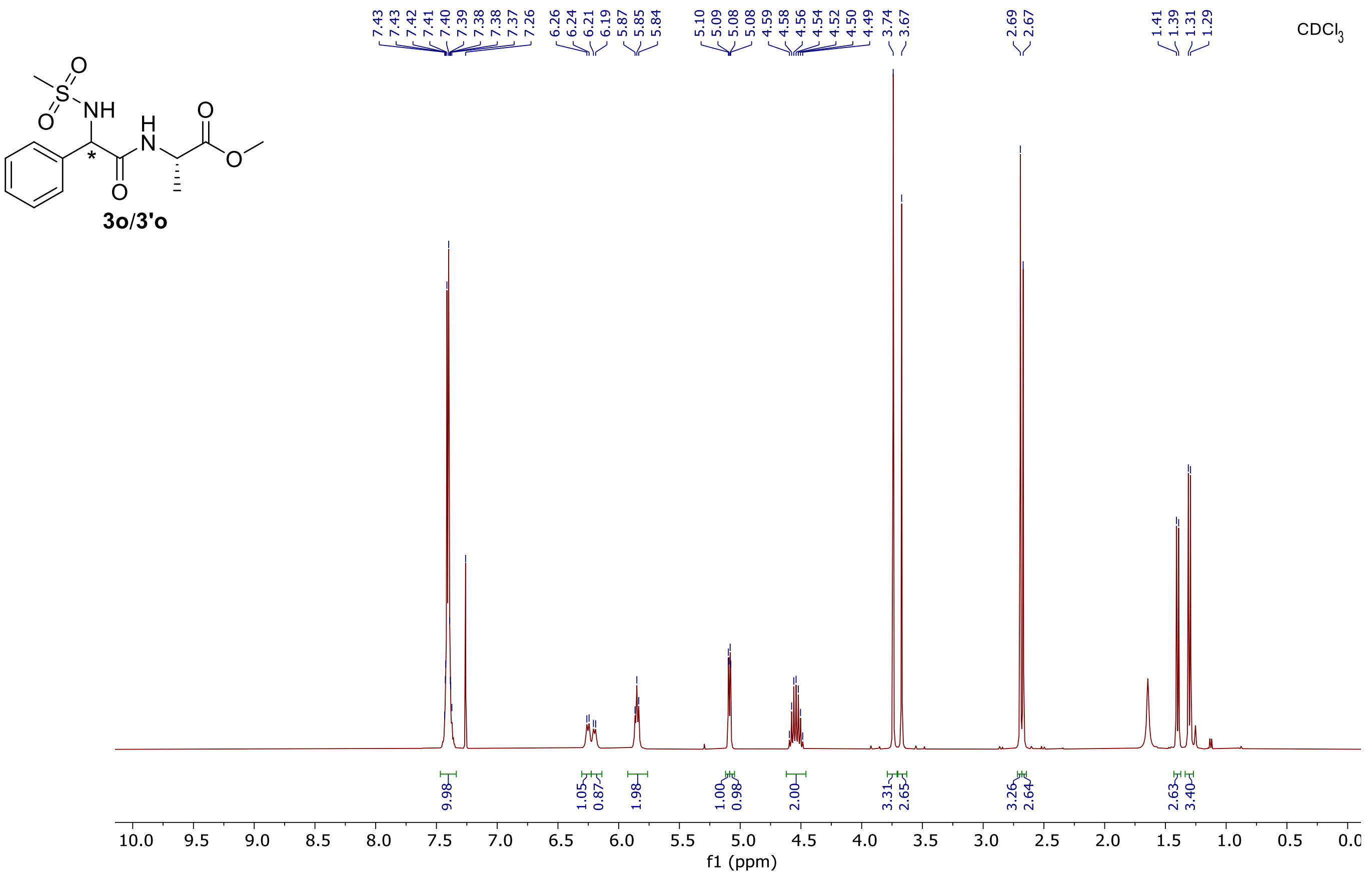

3o/3'o
7.43
7.43
7.42
7.41
7.40
7.39
7.38
7.38
7.37
7.26
6.26
6.24
6.21
6.19
5.87
5.85
5.84
5.10
5.09
5.08
5.08
4.59
4.58
4.56
4.54
4.52
4.50
4.49
3.74
3.67
2.69
2.67
1.41
1.39
1.31
1.29
CDCl3
9.98
1.05
0.87
1.98
1.00
0.98
2.00
3.31
2.65
3.26
2.64
2.63
3.40
10.0 9.5 9.0 8.5 8.0 7.5 7.0 6.5 6.0 5.5 5.0 4.5 4.0 3.5 3.0 2.5 2.0 1.5 1.0 0.5 0.0
f1 (ppm)

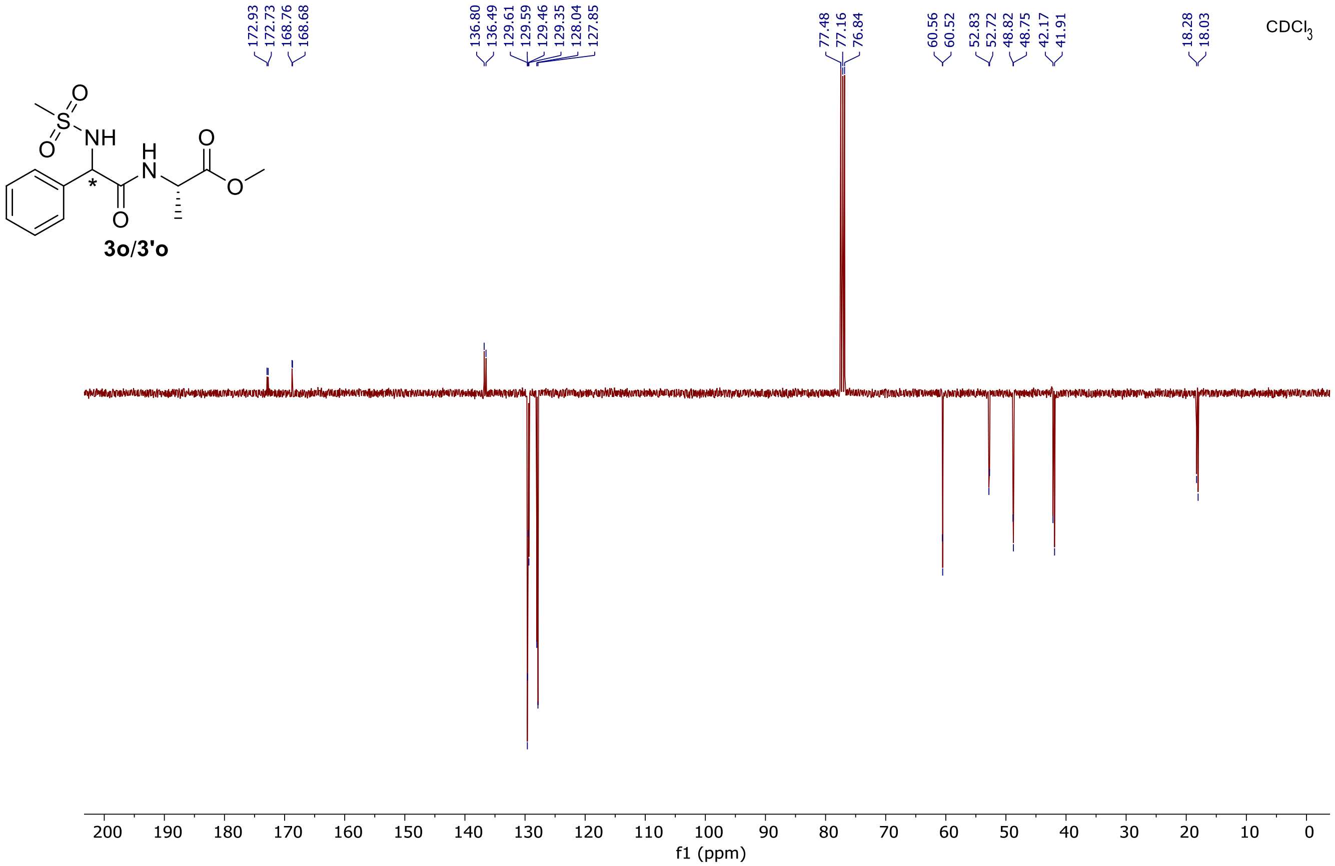

3o/3'o
172.93
172.73
168.76
168.68
136.80
136.49
129.61
129.59
129.46
129.35
128.04
127.85
77.48
77.16
76.84
60.56
60.52
52.83
52.72
48.82
48.75
42.17
41.91
18.28
18.03
CDCl3
200 190 180 170 160 150 140 130 120 110 100 90 80 70 60 50 40 30 20 10 0
f1 (ppm)

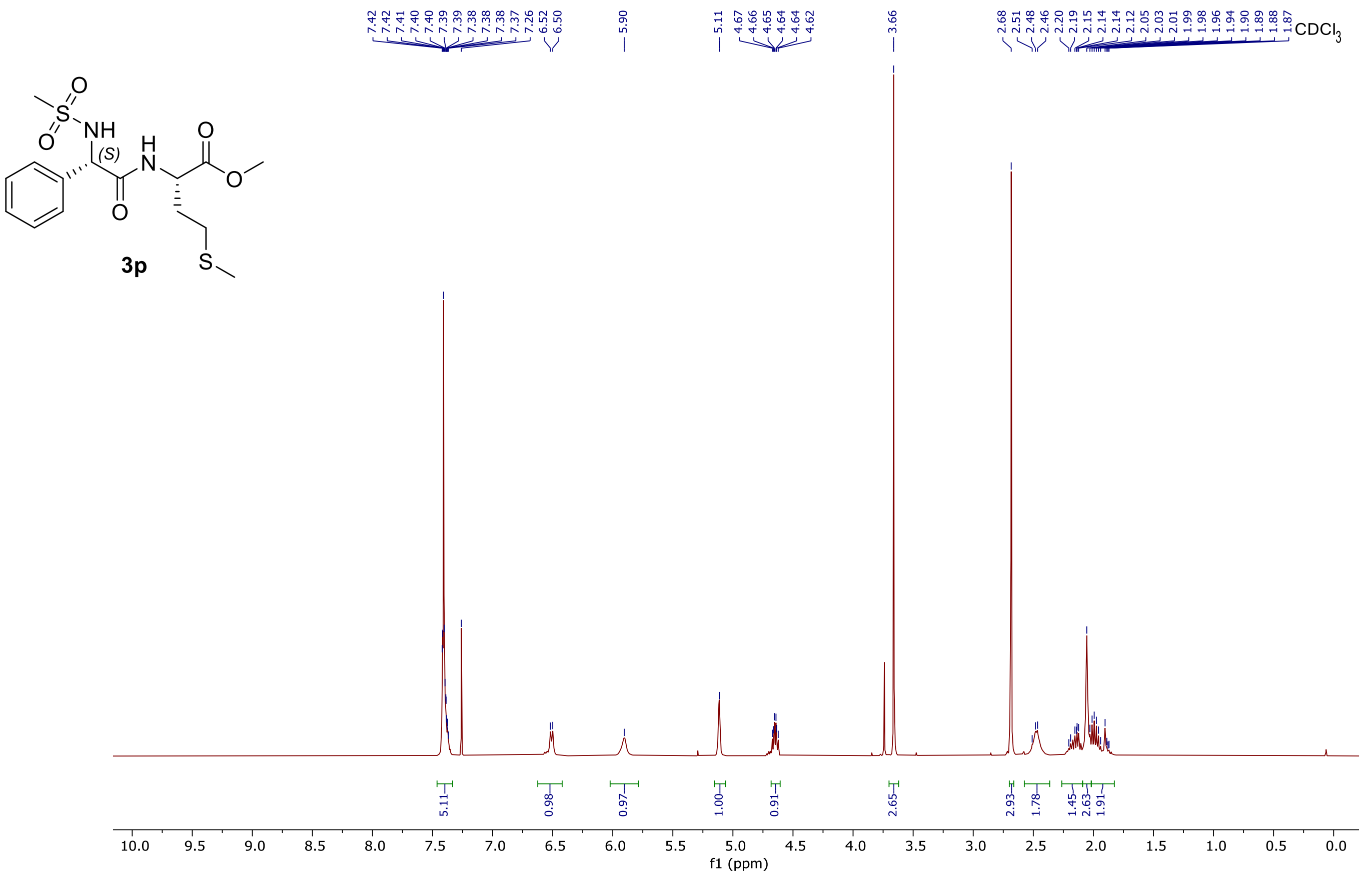
7.42 7.42 7.41 7.40 7.40 7.39 7.39 7.38 7.38 7.38 7.37 7.26 6.52 6.50
5.90
5.11 4.67 4.66 4.65 4.64 4.64 4.62
3.66
2.68 2.51 2.48 2.46 2.20 2.19 2.15 2.14 2.14 2.12 2.05 2.03 2.01 1.99 1.98 1.96 1.94 1.90 1.89 1.88 1.87
CDCl3
NH
(S)
H
N
O
S
3p
5.11 0.98 0.97 1.00 0.91 2.65 2.93 1.78 1.45 2.63 1.91
10.0 9.5 9.0 8.5 8.0 7.5 7.0 6.5 6.0 5.5 5.0 4.5 4.0 3.5 3.0 2.5 2.0 1.5 1.0 0.5 0.0
f1 (ppm)

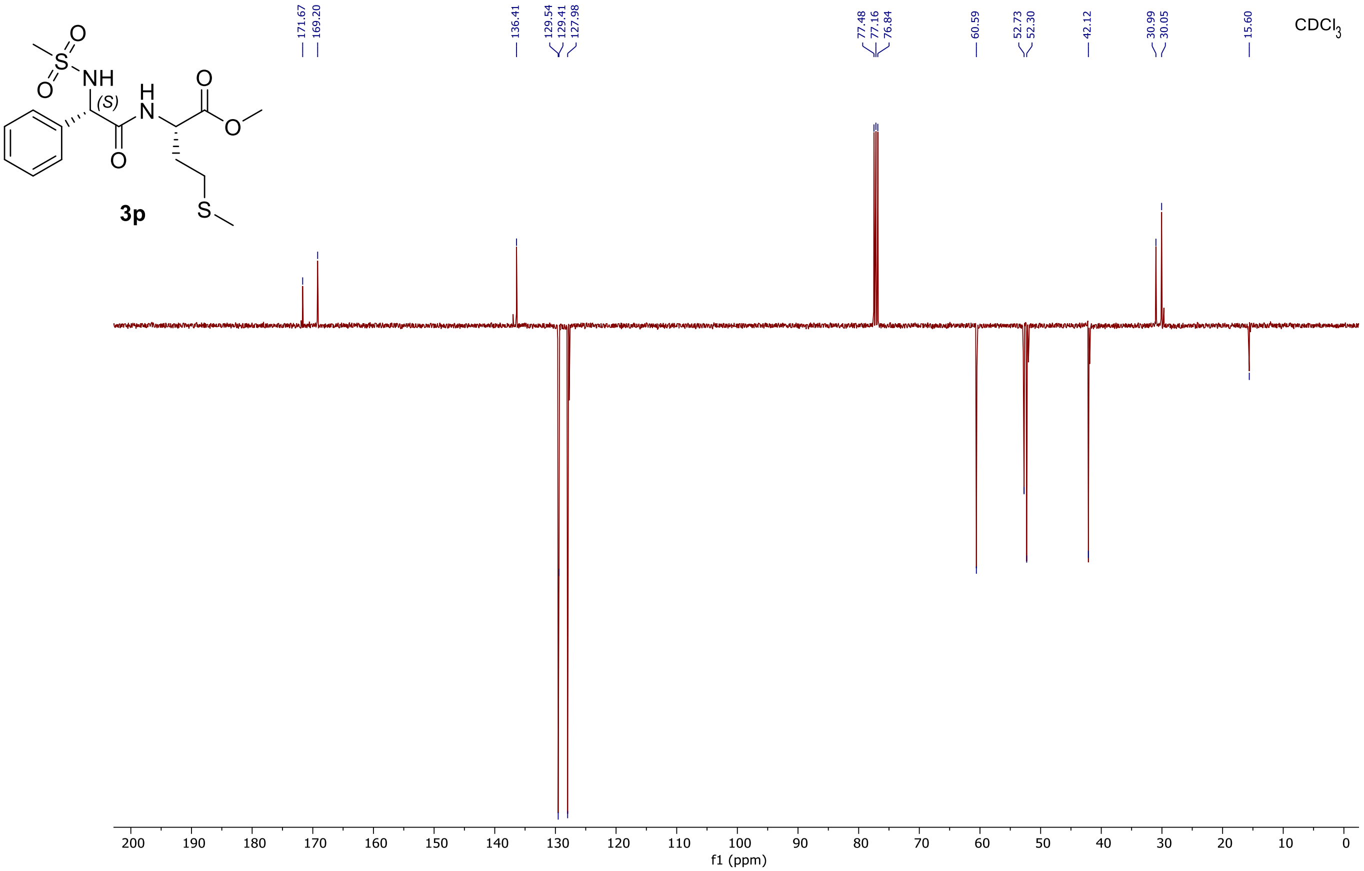
171.67 169.20
136.41
129.54 129.41 127.98
77.48 77.16 76.84
60.59
52.73 52.30
42.12
30.99 30.05
15.60
CDCl3
NH
(S)
H
N
O
S
3p
200 190 180 170 160 150 140 130 120 110 100 90 80 70 60 50 40 30 20 10 0
f1 (ppm)

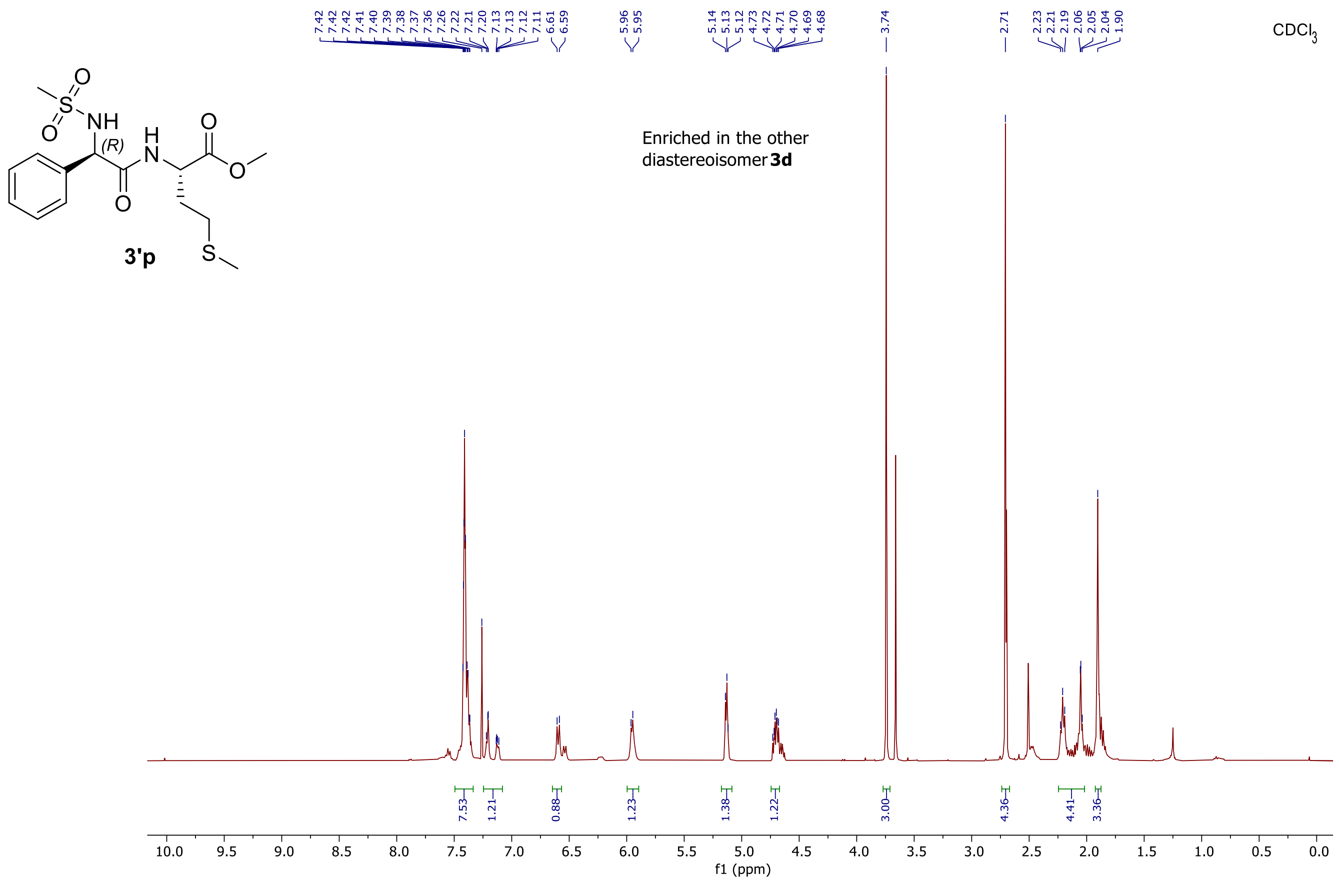
7.42 7.42 7.42 7.41 7.40 7.39 7.38 7.37 7.36 7.26 7.22 7.21 7.20 7.13 7.13 7.12 7.11 6.61 6.59
5.96 5.95
5.14 5.13 5.12 4.73 4.72 4.71 4.70 4.69 4.68
3.74
2.71
2.23 2.21 2.19 2.06 2.05 2.04 1.90
CDCl3
Enriched in the other diastereoisomer 3d
3'p
7.53 1.21 0.88 1.23 1.38 1.22 3.00 4.36 4.41 3.36
f1 (ppm)

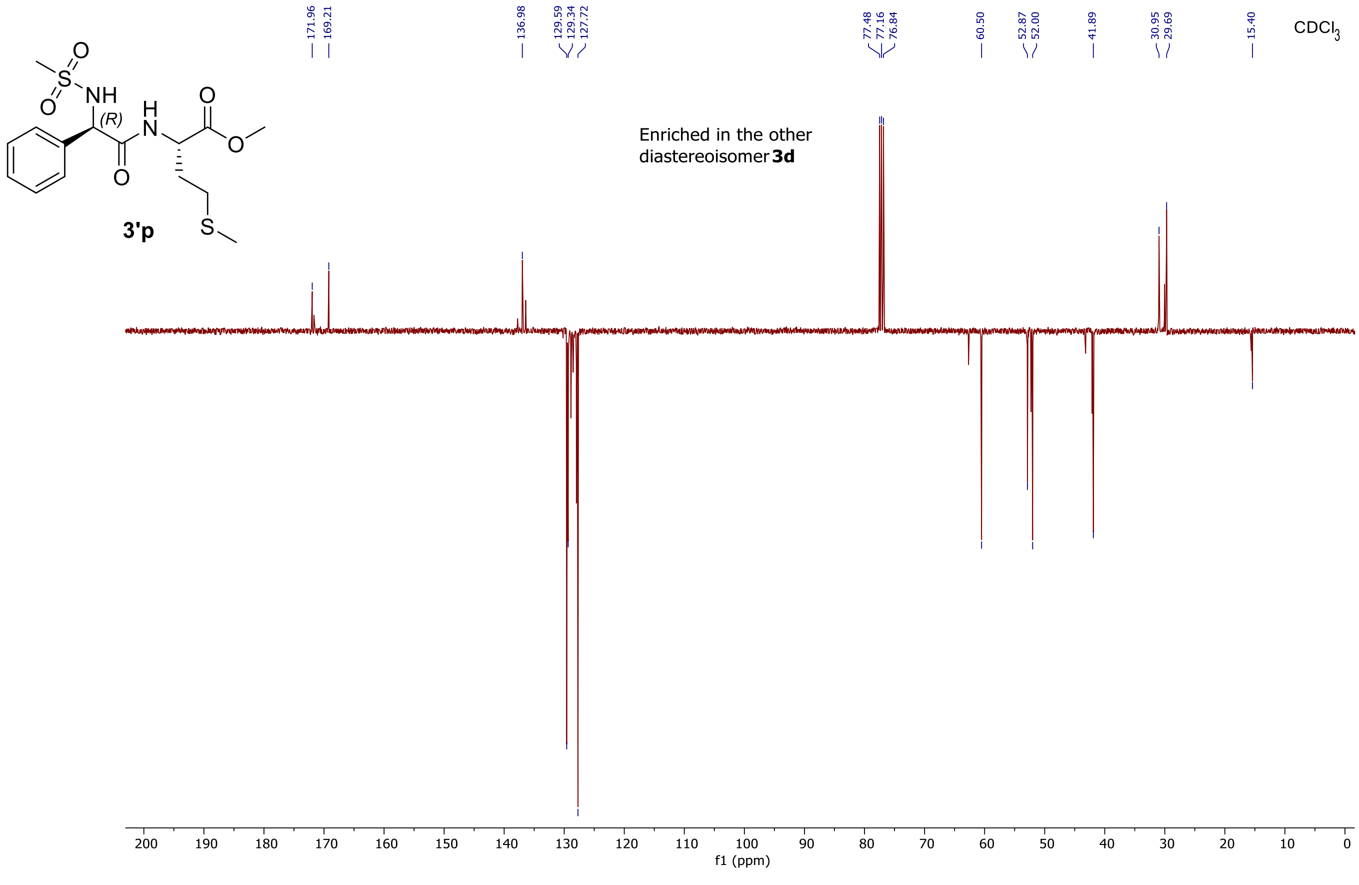
171.96 169.21
136.98
129.59 129.34 127.72
77.48 77.16 76.84
60.50
52.87 52.00
41.89
30.95 29.69
15.40
CDCl3
Enriched in the other diastereoisomer 3d
3'p
f1 (ppm)

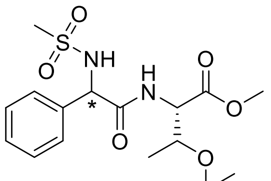

3q/3'q

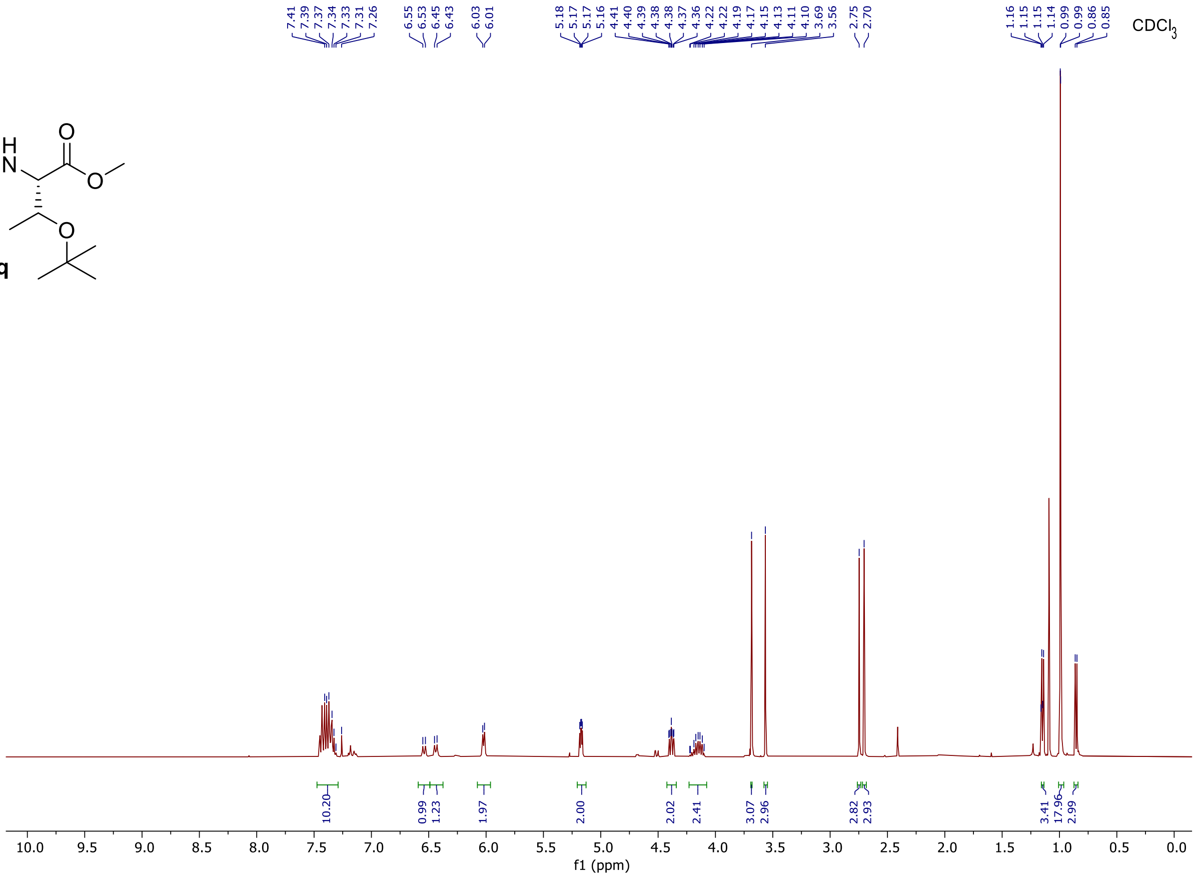
7.41 7.39 7.37 7.34 7.33 7.31 7.26
6.55 6.53 6.45 6.43
6.03 6.01
5.18 5.17 5.17 5.16 4.41 4.40 4.39 4.38 4.38 4.37 4.36 4.22 4.22 4.19 4.17 4.15 4.13 4.11 4.10 3.69 3.56
2.75 2.70
1.16 1.15 1.15 1.14 0.99 0.99 0.86 0.85
CDCl3
10.20 0.99 1.23 1.97 2.00 2.02 2.41 3.07 2.96 2.82 2.93 3.41 17.96 2.99
10.0 9.5 9.0 8.5 8.0 7.5 7.0 6.5 6.0 5.5 5.0 4.5 4.0 3.5 3.0 2.5 2.0 1.5 1.0 0.5 0.0
f1 (ppm)

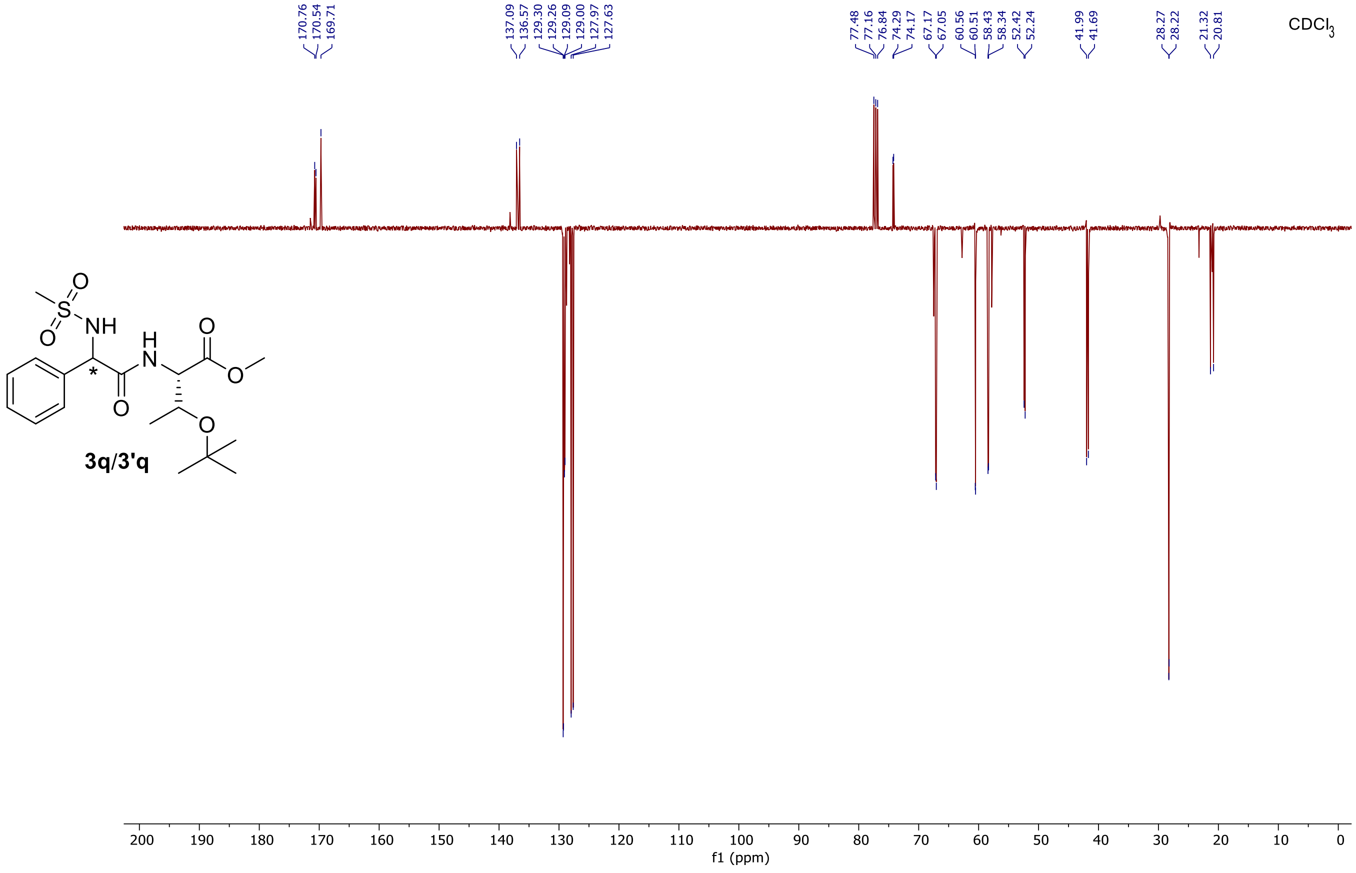
170.76 170.54 169.71
137.09 136.57 129.30 129.26 129.09 129.00 127.97 127.63
77.48 77.16 76.84 74.29 74.17 67.17 67.05 60.56 60.51 58.43 58.34 52.42 52.24
41.99 41.69
28.27 28.22 21.32 20.81
CDCl3
200 190 180 170 160 150 140 130 120 110 100 90 80 70 60 50 40 30 20 10 0
f1 (ppm)

3q/3'q

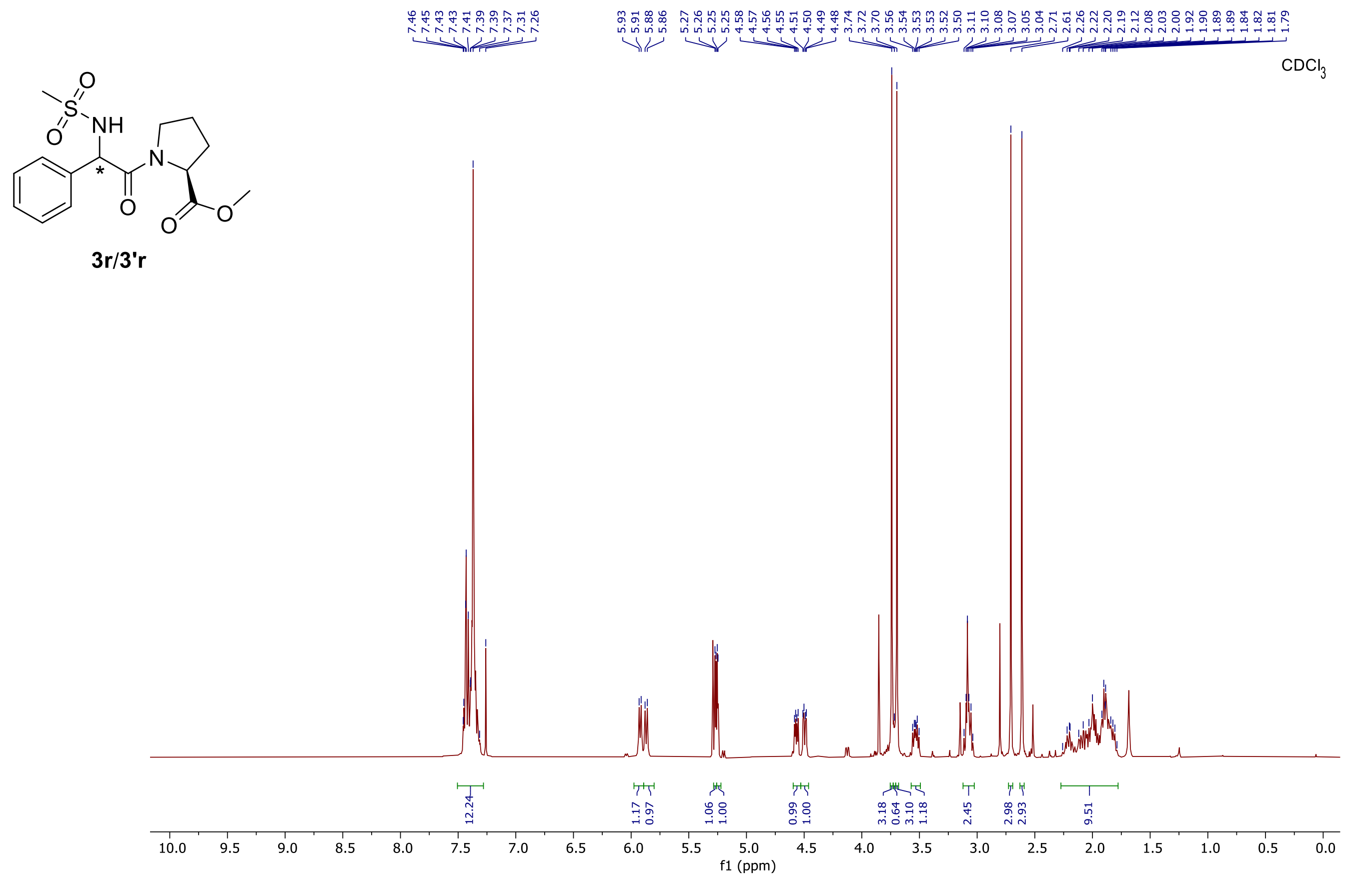
7.46
7.45
7.43
7.43
7.41
7.39
7.39
7.37
7.31
7.26
5.93
5.91
5.88
5.86
5.27
5.26
5.25
5.25
4.58
4.57
4.56
4.55
4.51
4.50
4.49
4.48
3.74
3.72
3.70
3.56
3.54
3.53
3.53
3.52
3.50
3.11
3.10
3.08
3.07
3.05
3.04
2.71
2.61
2.26
2.22
2.20
2.19
2.12
2.08
2.03
2.00
1.92
1.90
1.89
1.84
1.82
1.81
1.79
CDCl3
3r/3'r
12.24
1.17
0.97
1.06
1.00
0.99
1.00
3.18
0.64
3.10
1.18
2.45
2.98
2.93
9.51
f1 (ppm)

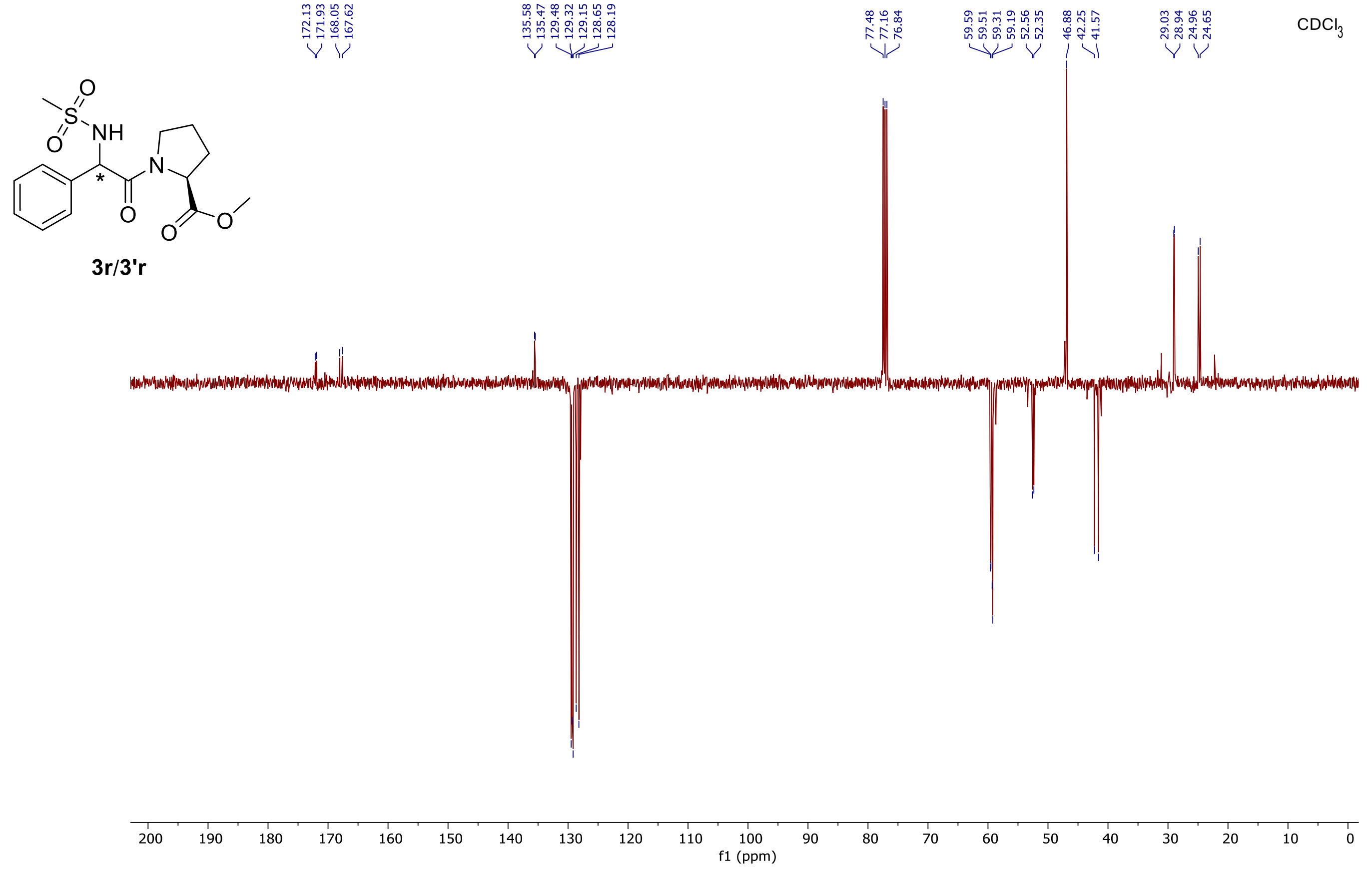
172.13
171.93
168.05
167.62
135.58
135.47
129.48
129.32
129.15
128.65
128.19
77.48
77.16
76.84
59.59
59.51
59.31
59.19
52.56
52.35
46.88
42.25
41.57
29.03
28.94
24.96
24.65
CDCl3
3r/3'r
f1 (ppm)

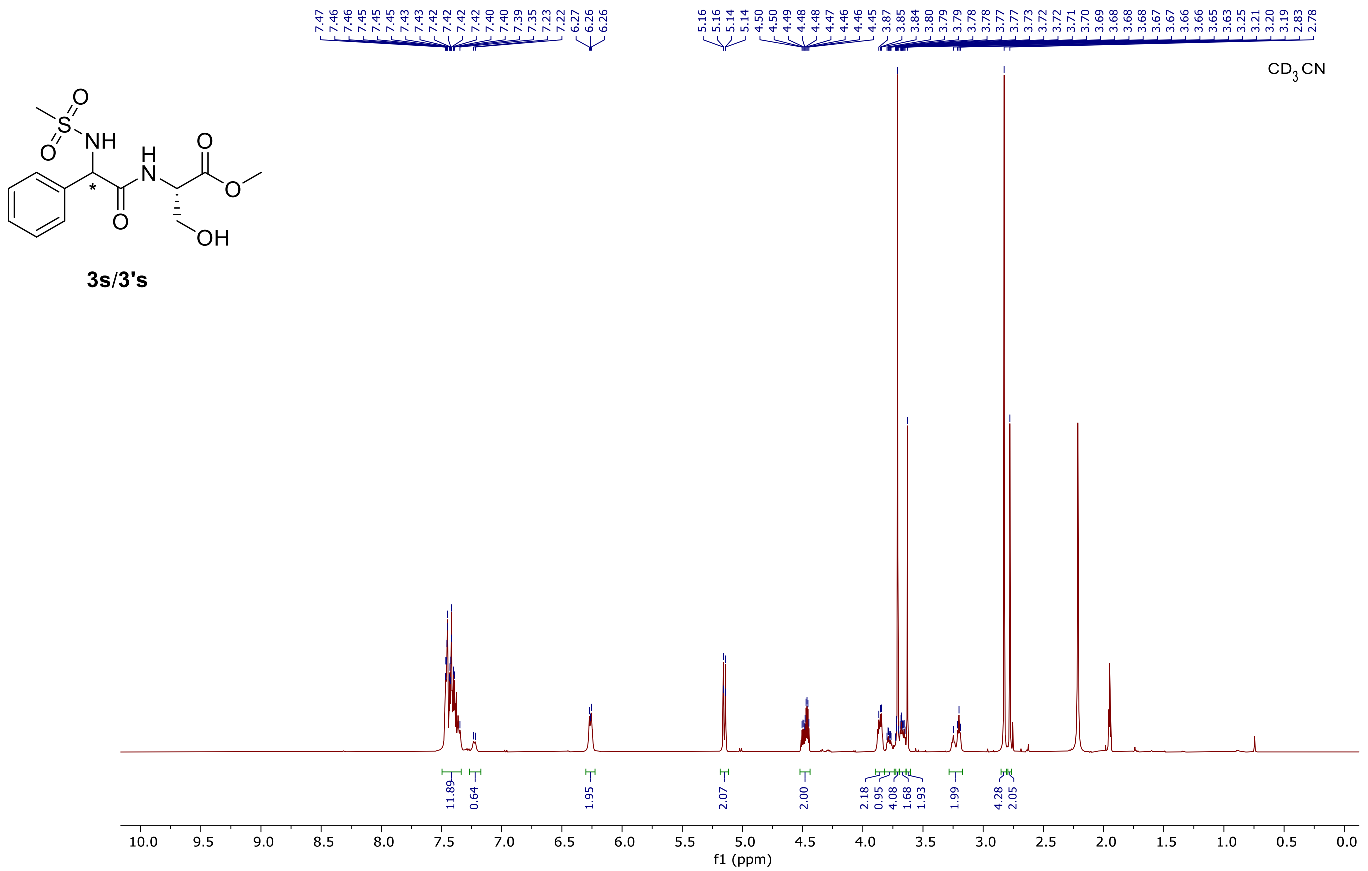

3s/3's

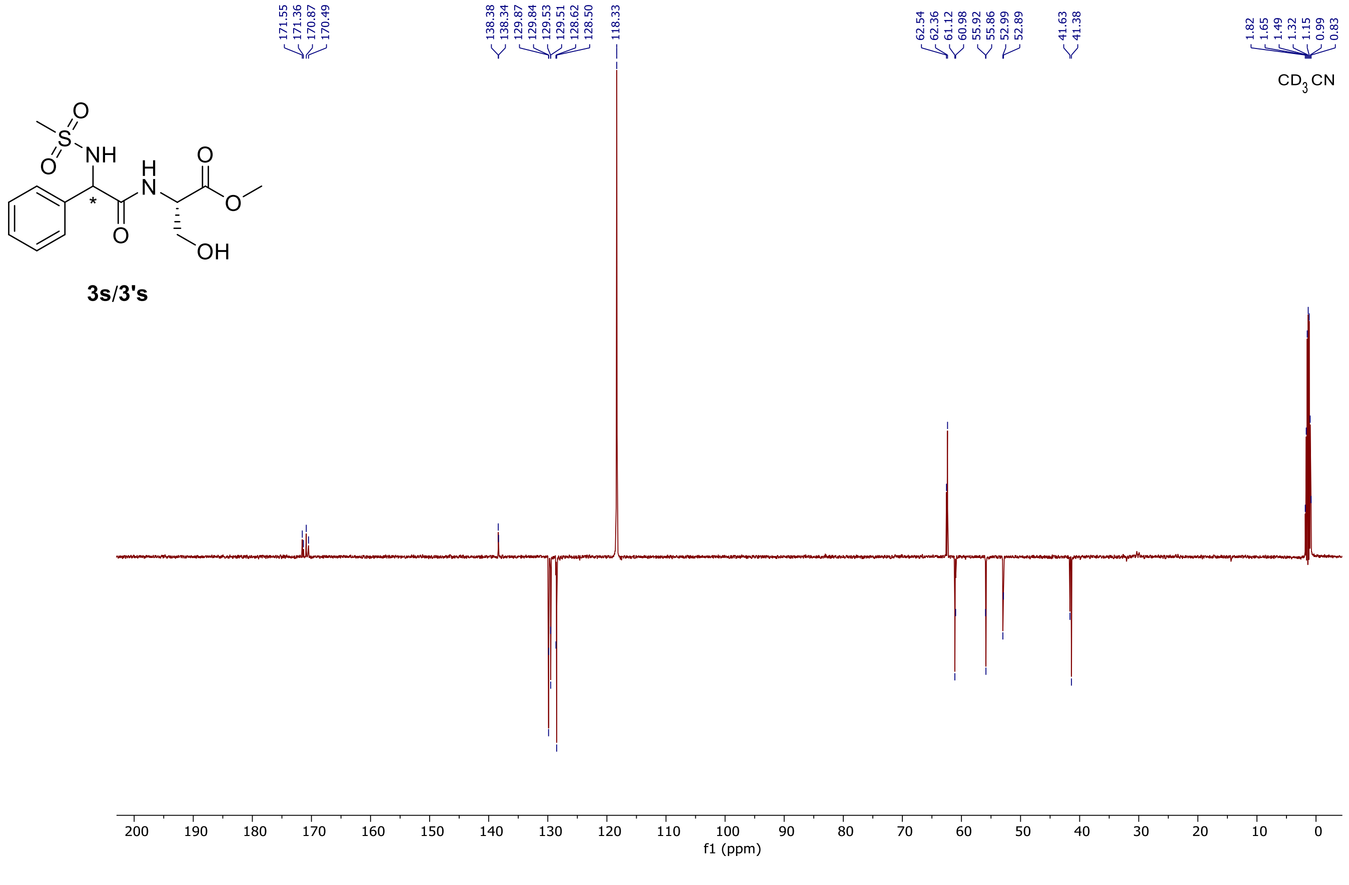

3s/3's

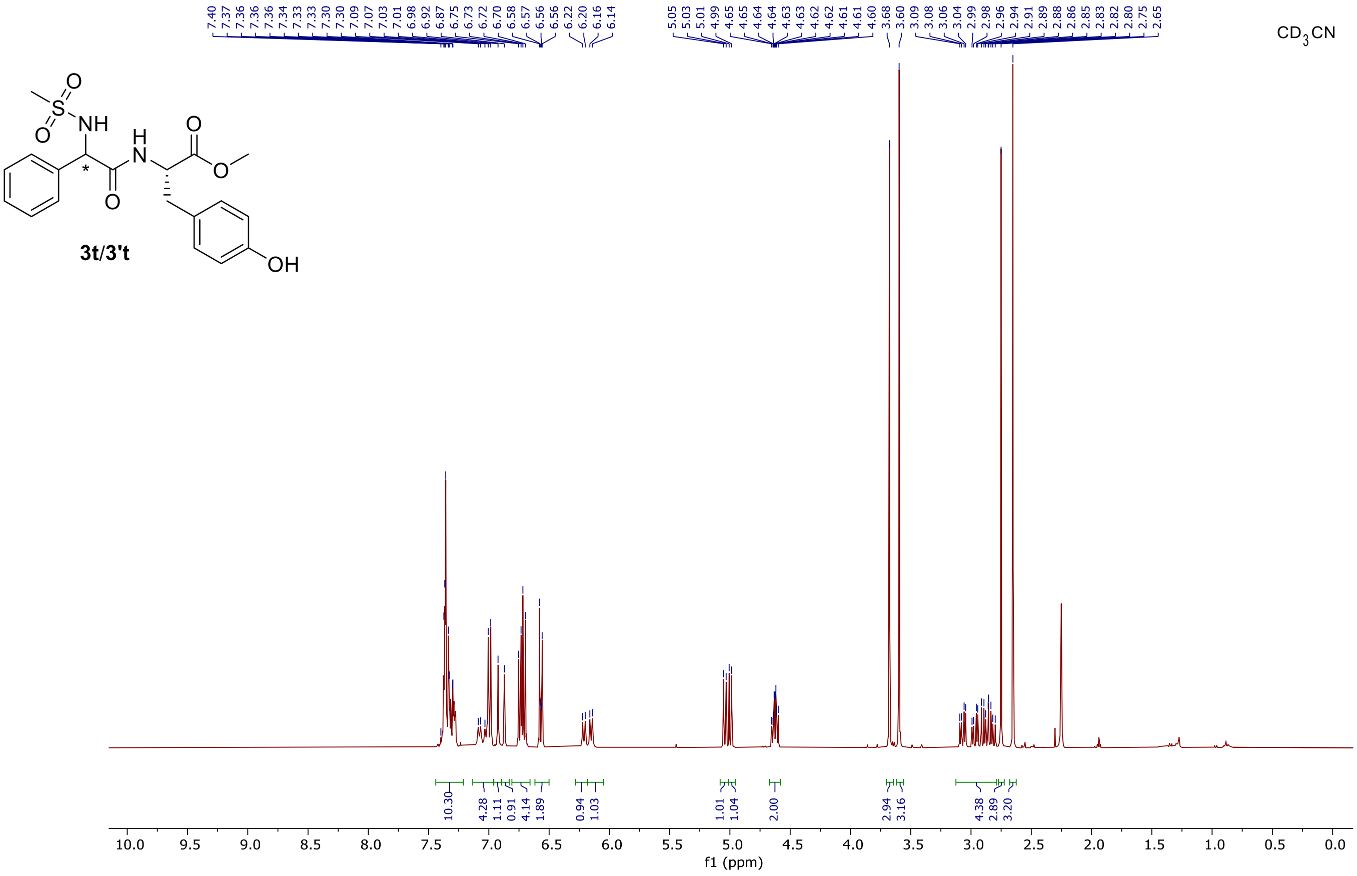
3t/3't
7.40 7.37 7.36 7.36 7.36 7.34 7.33 7.33 7.30 7.30 7.09 7.07 7.03 7.01 6.98 6.92 6.87 6.75 6.73 6.72 6.70 6.58 6.57 6.56 6.56 6.22 6.20 6.16 6.14
5.05 5.03 5.01 4.99 4.65 4.65 4.64 4.64 4.63 4.63 4.62 4.62 4.61 4.61 4.60 3.68 3.60 3.09 3.08 3.06 3.04 2.99 2.98 2.96 2.94 2.91 2.89 2.88 2.86 2.85 2.83 2.82 2.80 2.75 2.65
CD_3CN
10.30 4.28 1.11 0.91 4.14 1.89 0.94 1.03 1.01 1.04 2.00 2.94 3.16 4.38 2.89 3.20
10.0 9.5 9.0 8.5 8.0 7.5 7.0 6.5 6.0 5.5 5.0 4.5 4.0 3.5 3.0 2.5 2.0 1.5 1.0 0.5 0.0
f1 (ppm)

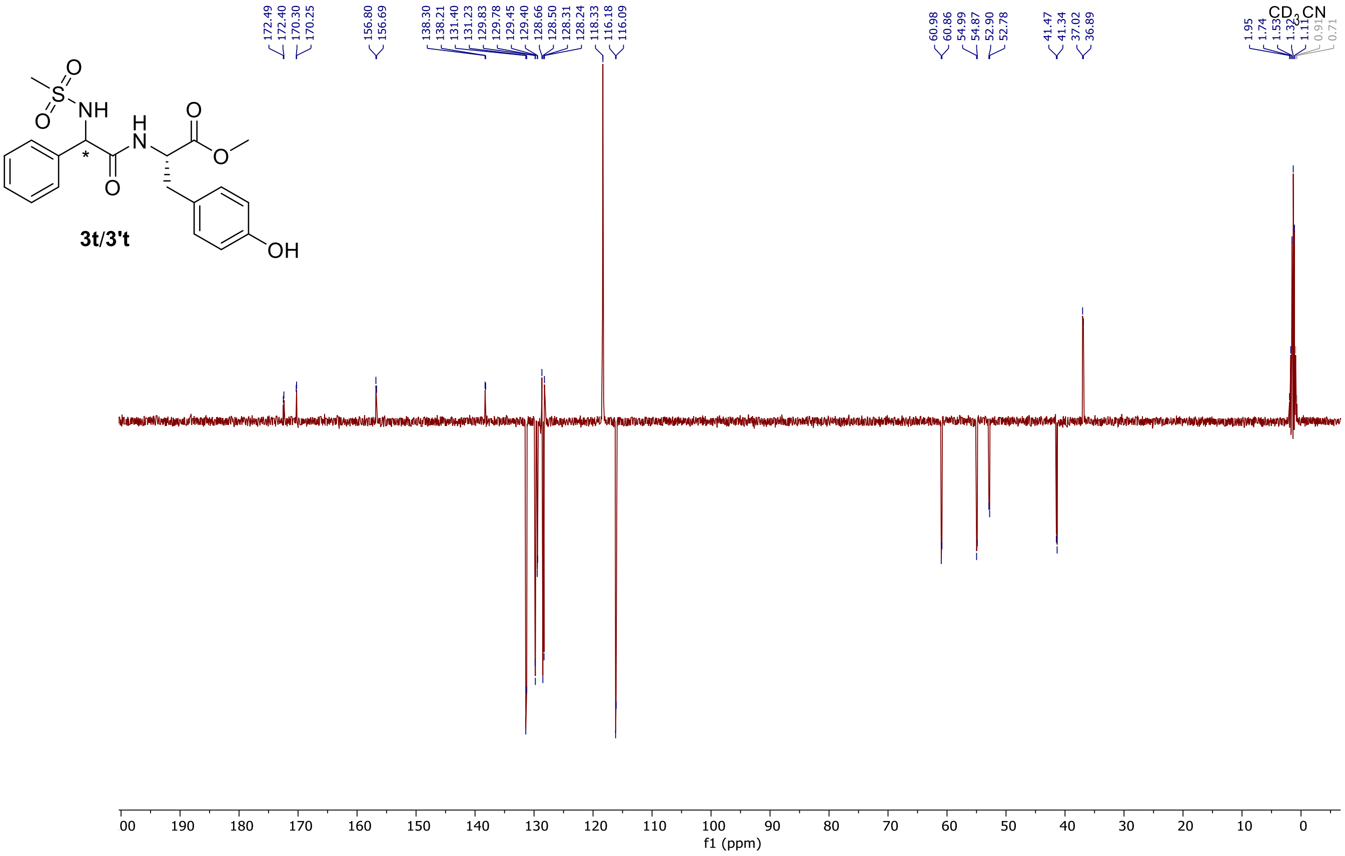
3t/3't
172.49 172.40 170.30 170.25 156.80 156.69 138.30 138.21 131.40 131.23 129.83 129.78 129.45 129.40 128.66 128.50 128.31 128.24 118.33 116.18 116.09
60.98 60.86 54.99 54.87 52.90 52.78 41.47 41.34 37.02 36.89
CD_3CN
1.95 1.74 1.53 1.32 1.11 0.91 0.71
00 190 180 170 160 150 140 130 120 110 100 90 80 70 60 50 40 30 20 10 0
f1 (ppm)

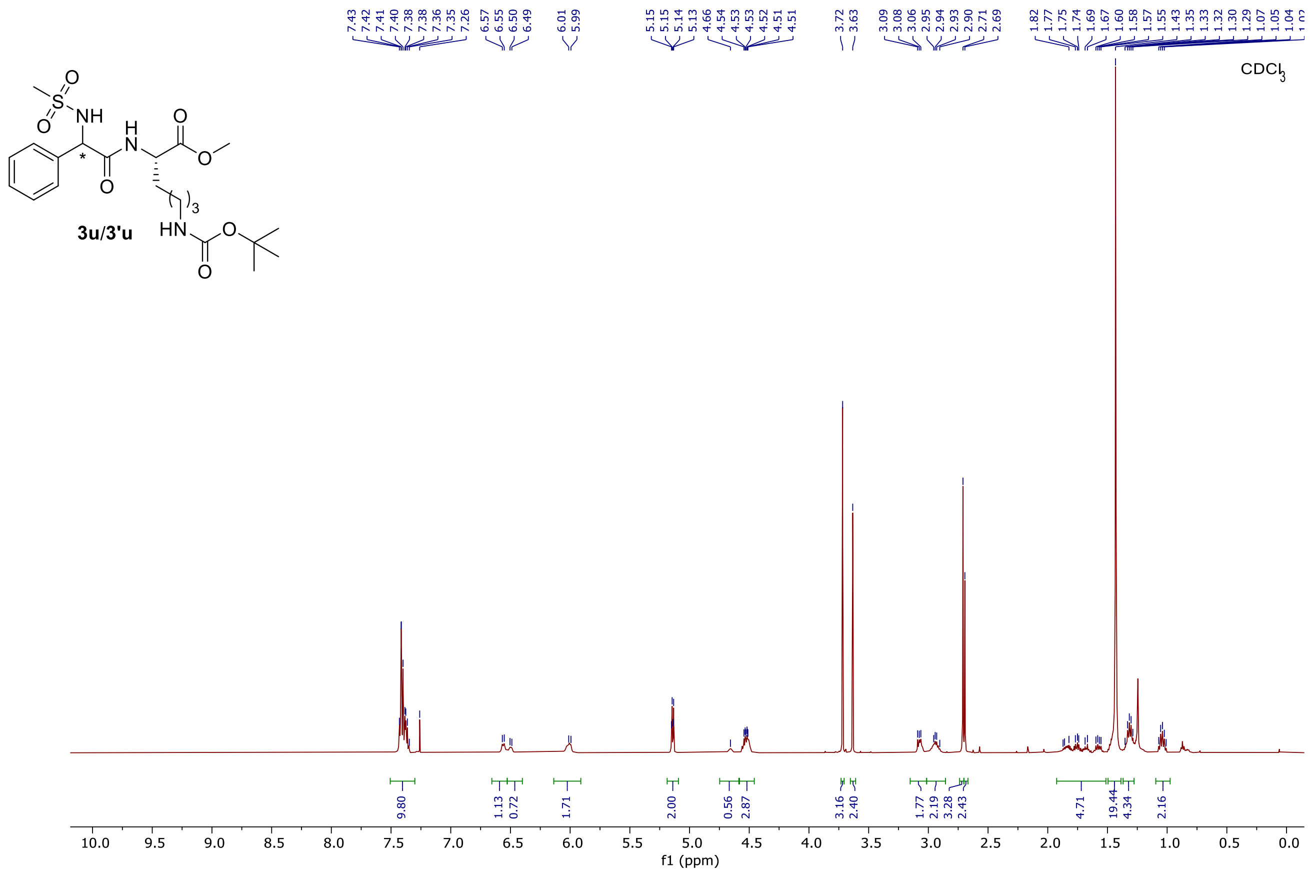
3u/3'u
CDCl3
7.43 7.42 7.41 7.40 7.38 7.38 7.36 7.35 7.26 6.57 6.55 6.50 6.49 6.01 5.99 5.15 5.15 5.14 5.13 4.66 4.54 4.53 4.53 4.52 4.51 4.51 3.72 3.63 3.09 3.08 3.06 2.95 2.94 2.93 2.90 2.71 2.69 1.82 1.77 1.75 1.74 1.69 1.67 1.60 1.58 1.57 1.55 1.43 1.35 1.33 1.32 1.30 1.29 1.07 1.05 1.04
9.80 1.13 0.72 1.71 2.00 0.56 2.87 3.16 2.40 1.77 2.19 3.28 2.43 4.71 19.44 4.34 2.16
f1 (ppm)

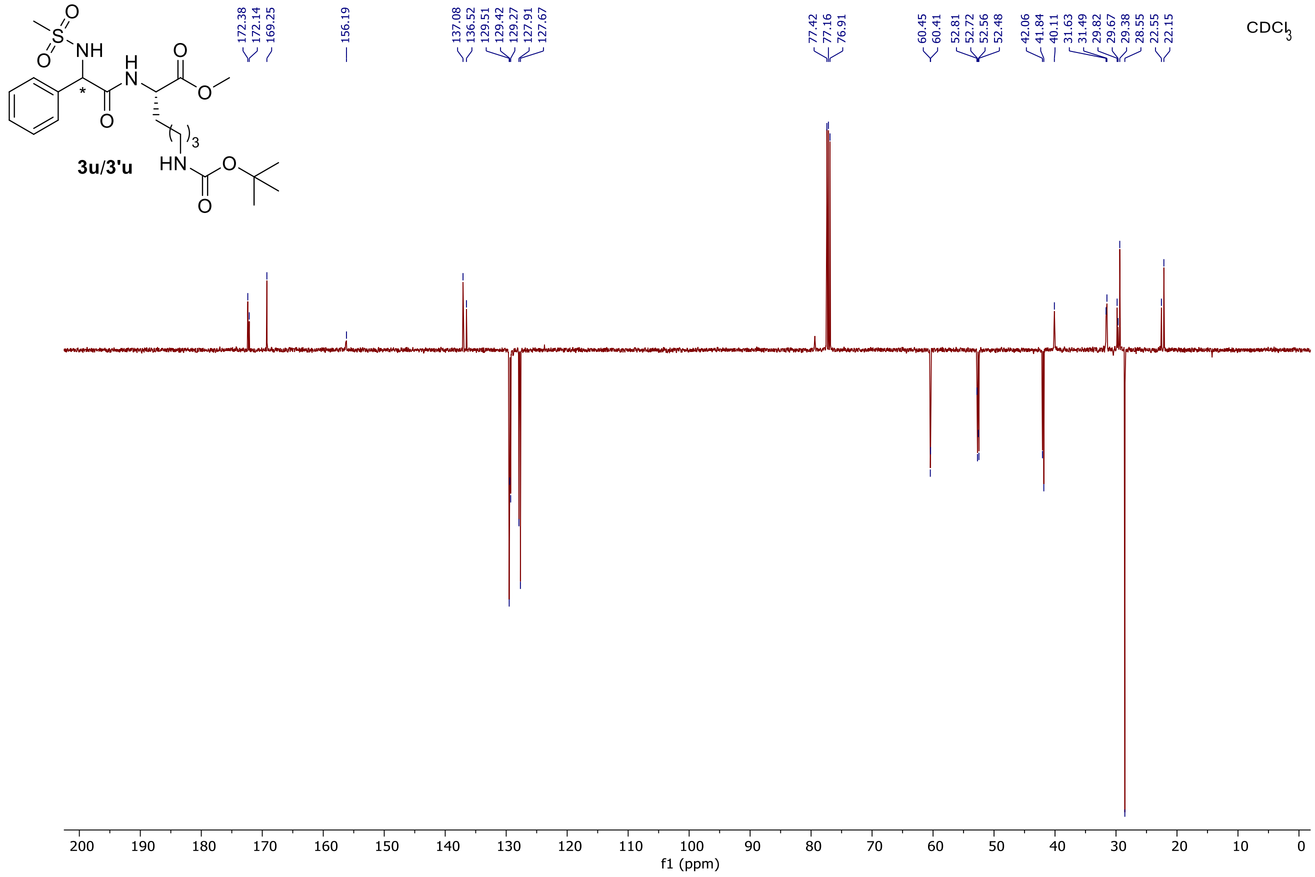
3u/3'u
CDCl3
172.38 172.14 169.25 156.19 137.08 136.52 129.51 129.42 129.27 127.91 127.67 77.42 77.16 76.91 60.45 60.41 52.81 52.72 52.56 52.48 42.06 41.84 40.11 31.63 31.49 29.82 29.67 29.38 28.55 22.55 22.15
f1 (ppm)

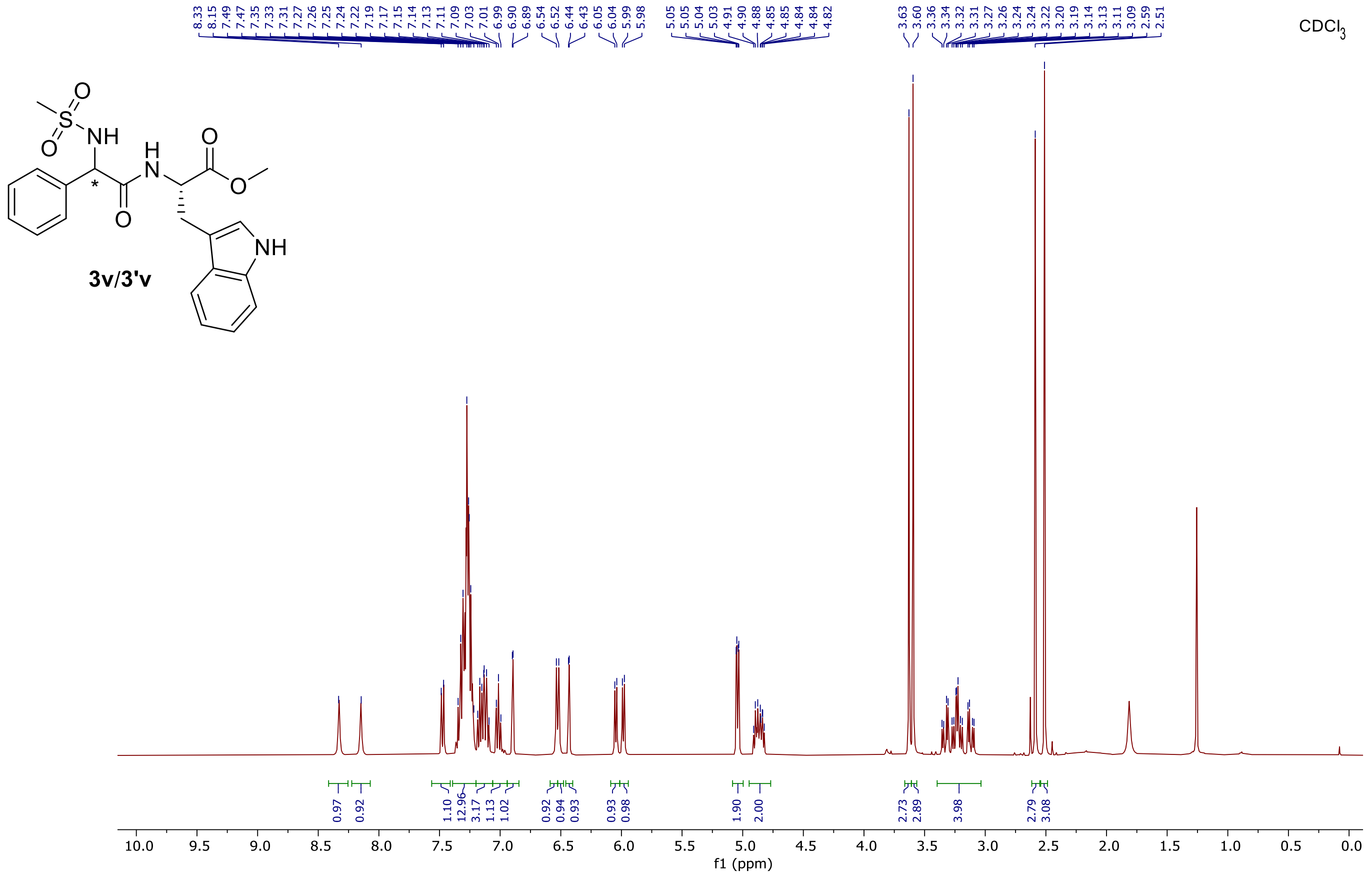

CDCl3
3v/3'v
8.33 8.15 7.49 7.47 7.35 7.33 7.31 7.27 7.26 7.25 7.24 7.22 7.19 7.17 7.15 7.14 7.13 7.11 7.09 7.03 7.01 6.99 6.90 6.89 6.54 6.52 6.44 6.43 6.05 6.04 5.99 5.98 5.05 5.05 5.04 5.03 4.91 4.90 4.88 4.85 4.85 4.84 4.84 4.82 3.63 3.60 3.36 3.34 3.32 3.31 3.27 3.26 3.24 3.24 3.22 3.20 3.19 3.14 3.13 3.11 3.09 2.59 2.51
0.97 0.92 1.10 12.96 3.17 1.13 1.02 0.92 0.94 0.93 0.93 0.98 1.90 2.00 2.73 2.89 3.98 2.79 3.08
f1 (ppm)

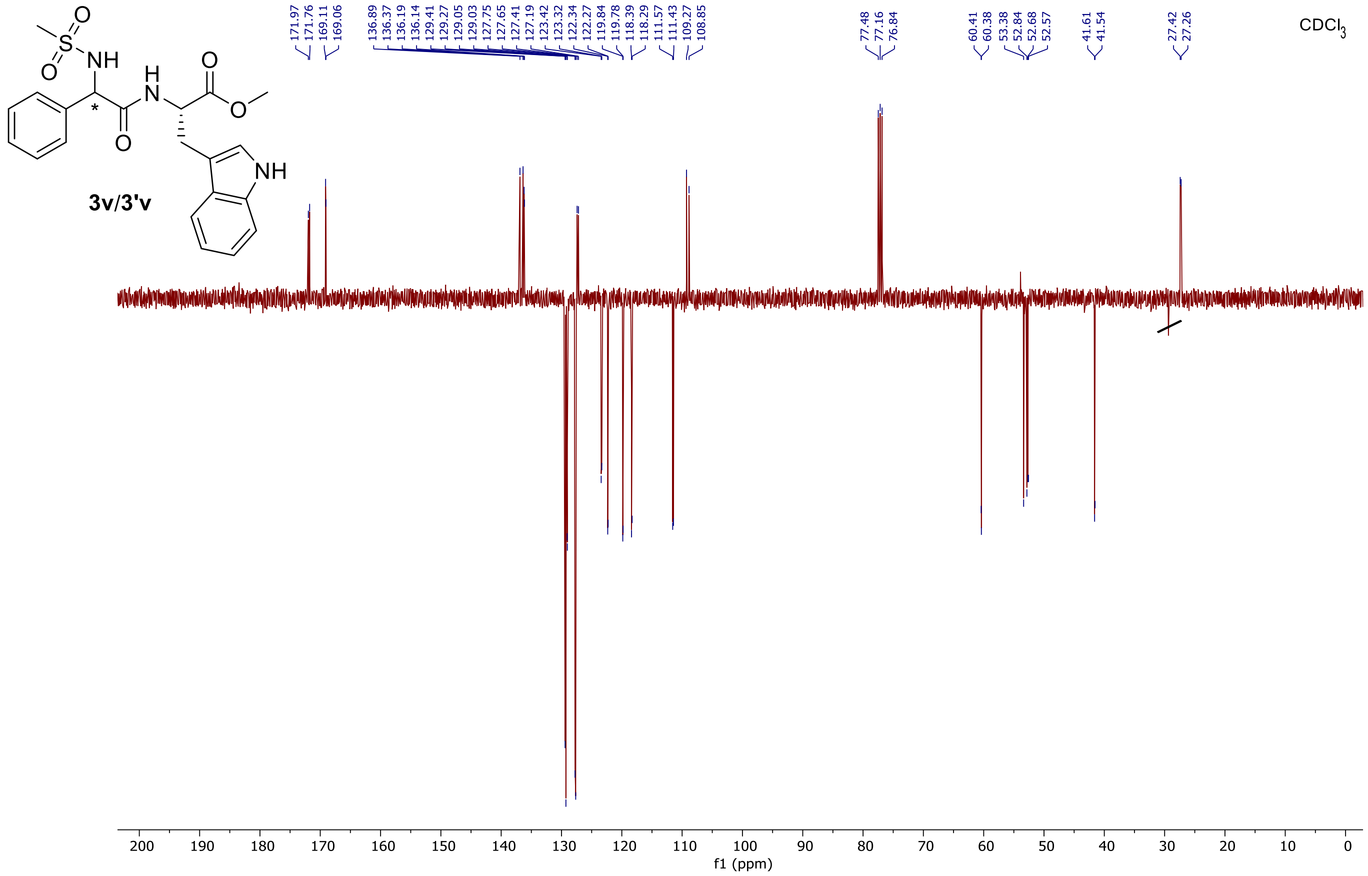

CDCl3
3v/3'v
171.97 171.76 169.11 169.06 136.89 136.37 136.19 136.14 129.41 129.27 129.05 129.03 127.75 127.65 127.41 127.19 123.42 123.32 122.34 122.27 119.84 119.78 118.39 118.29 111.57 111.43 109.27 108.85 77.48 77.16 76.84 60.41 60.38 53.38 52.84 52.68 52.57 41.61 41.54 27.42 27.26
f1 (ppm)

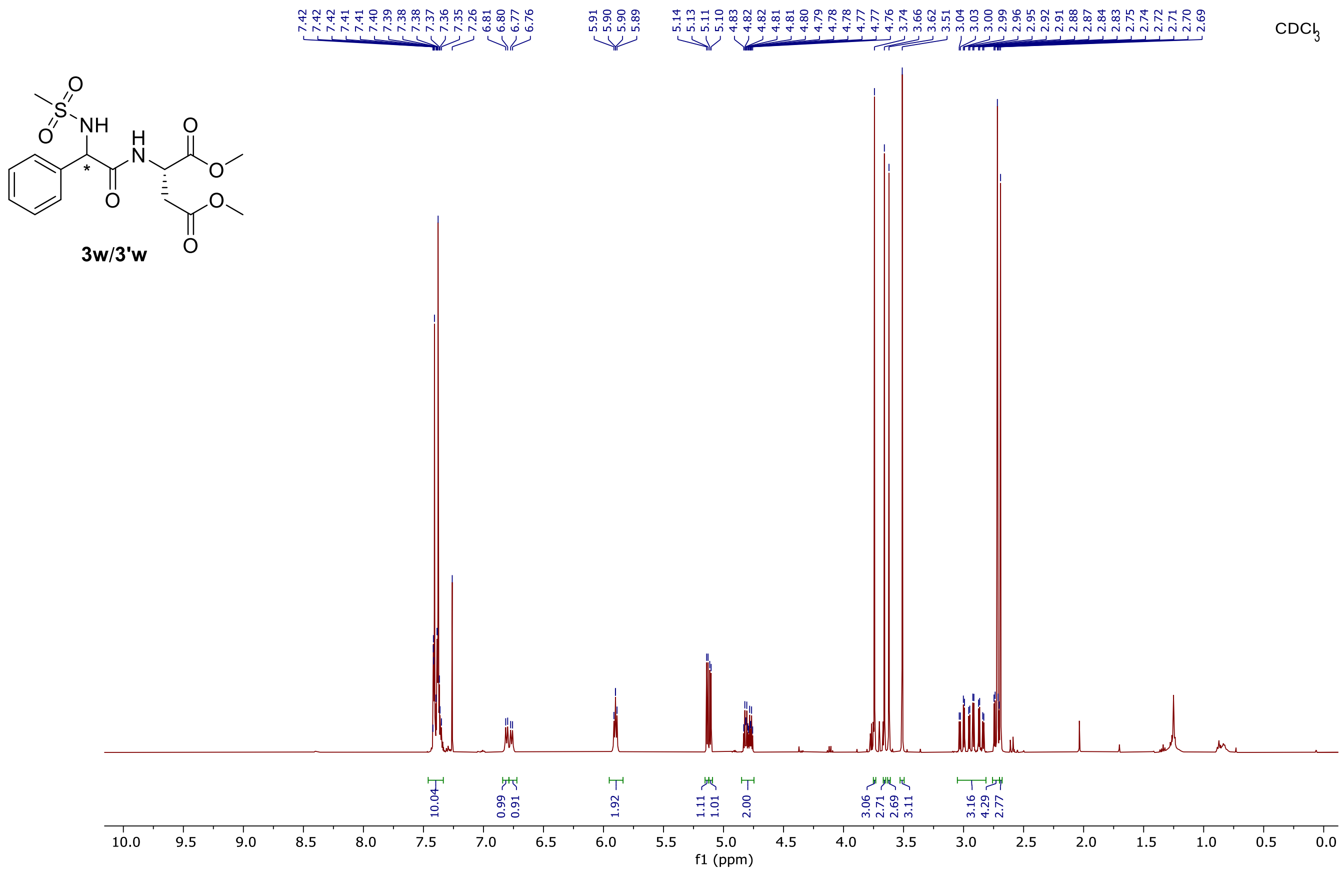
3w/3'w
CDCl3
7.42
7.42
7.42
7.41
7.41
7.40
7.39
7.38
7.38
7.37
7.36
7.35
7.26
6.81
6.80
6.77
6.76
5.91
5.90
5.90
5.89
5.14
5.13
5.11
5.10
4.83
4.82
4.82
4.81
4.81
4.80
4.79
4.78
4.78
4.77
4.77
4.76
3.74
3.66
3.62
3.51
3.04
3.03
3.00
2.99
2.96
2.95
2.92
2.91
2.88
2.87
2.84
2.83
2.75
2.74
2.72
2.71
2.70
2.69
10.04
0.99
0.91
1.92
1.11
1.01
2.00
3.06
2.71
2.69
3.11
3.16
4.29
2.77
f1 (ppm)

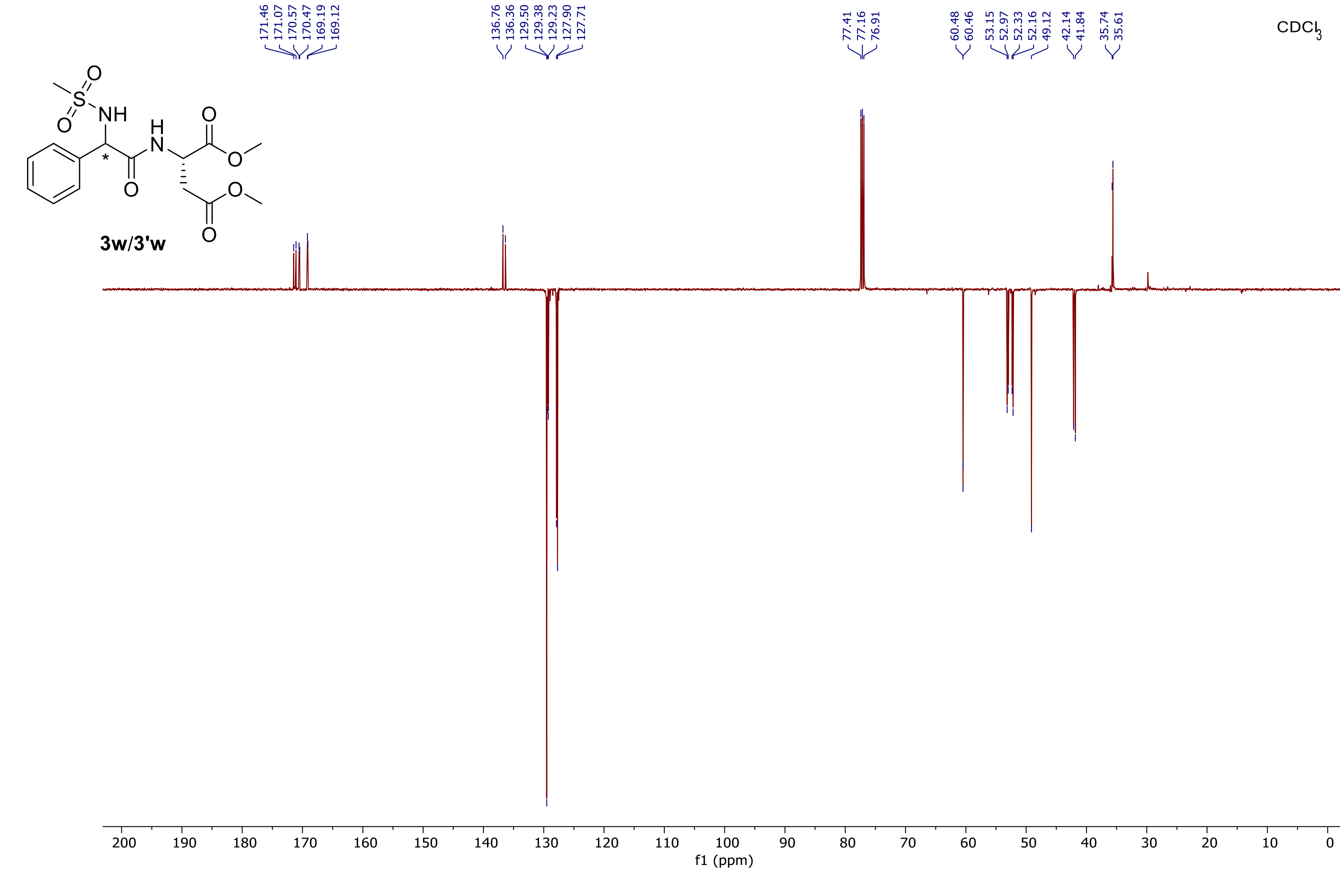
3w/3'w
CDCl3
171.46
171.07
170.57
170.47
169.19
169.12
136.76
136.36
129.50
129.38
129.23
127.90
127.71
77.41
77.16
76.91
60.48
60.46
53.15
52.97
52.33
52.16
49.12
42.14
41.84
35.74
35.61
f1 (ppm)

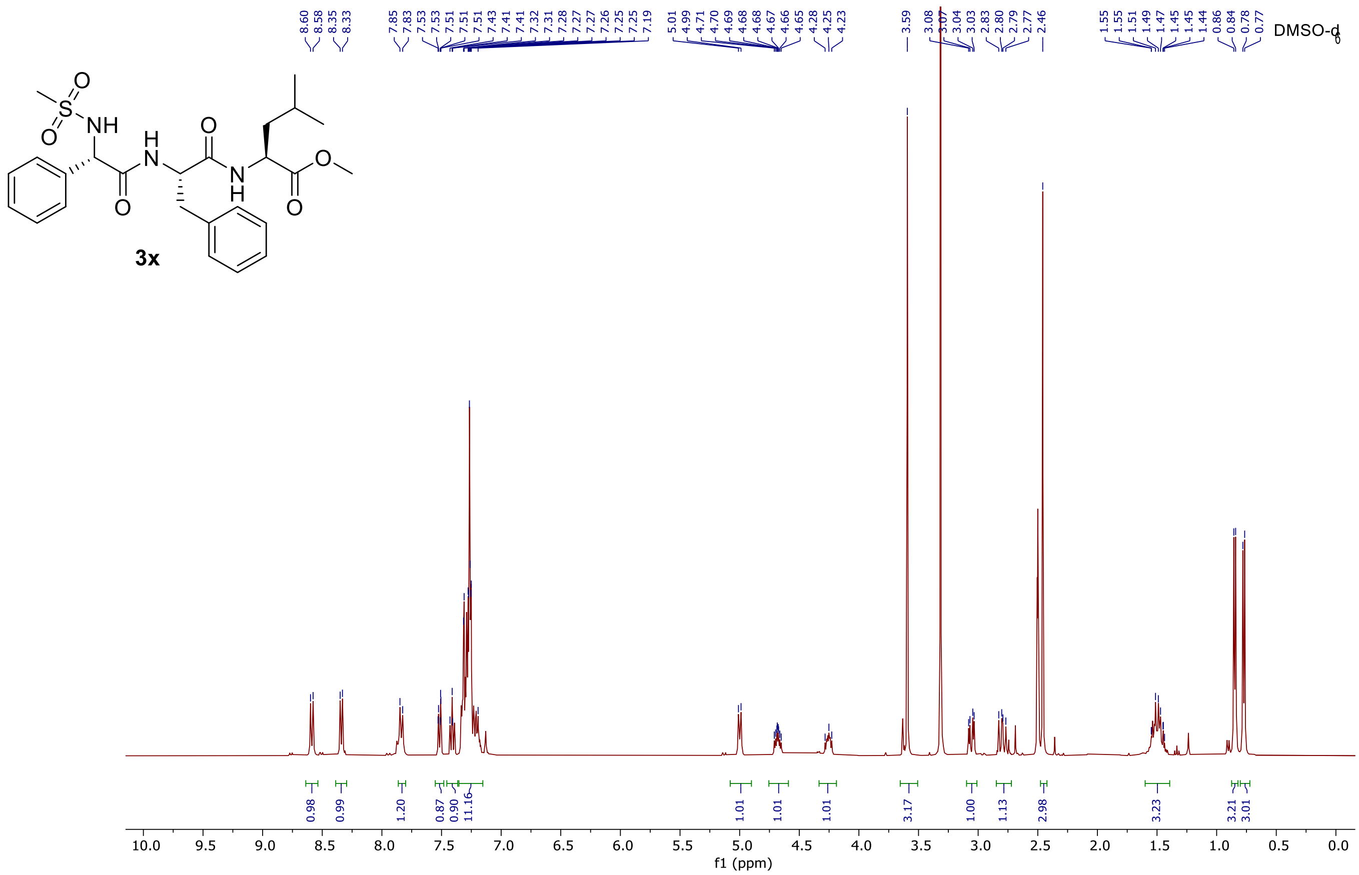

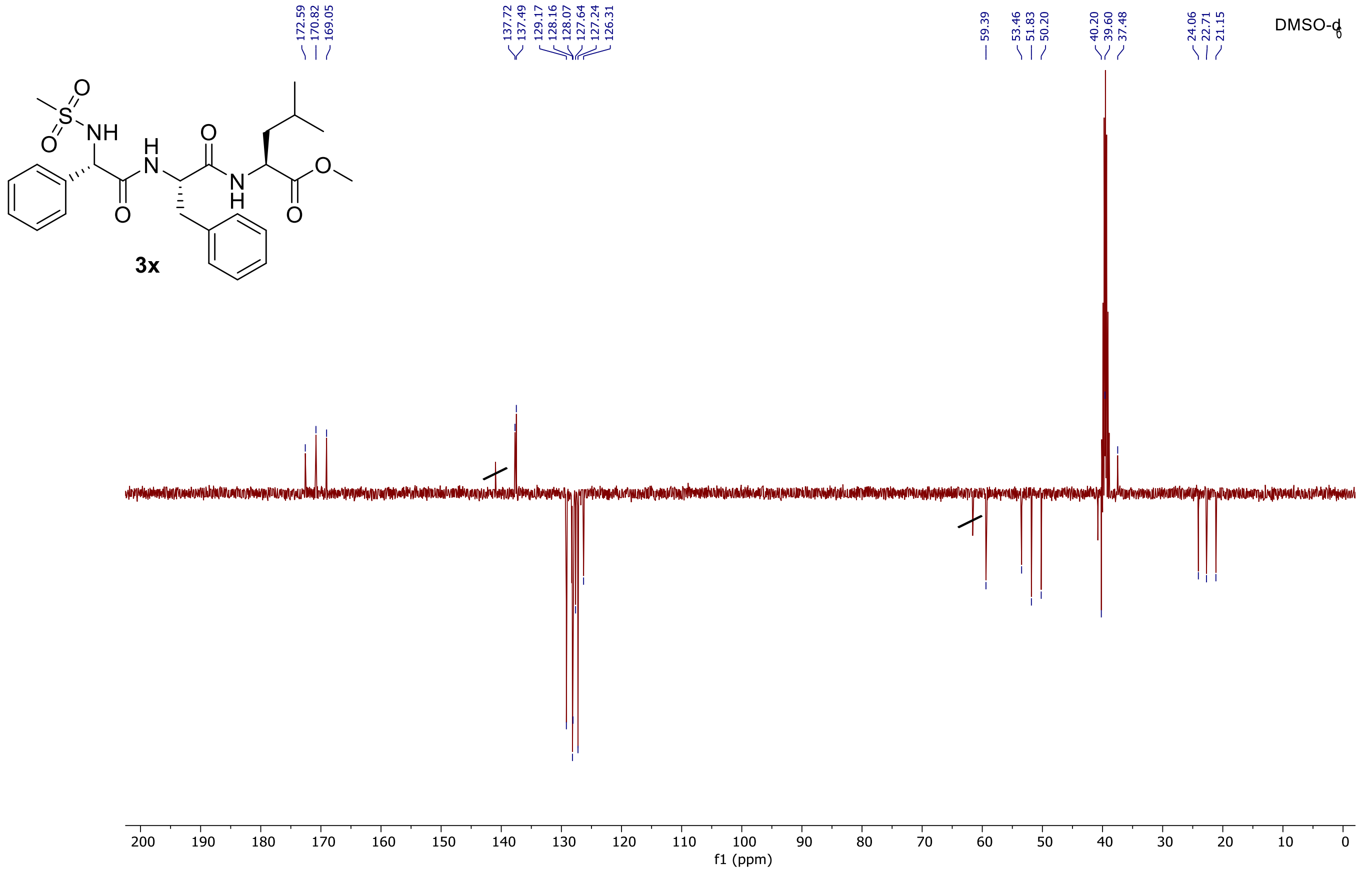

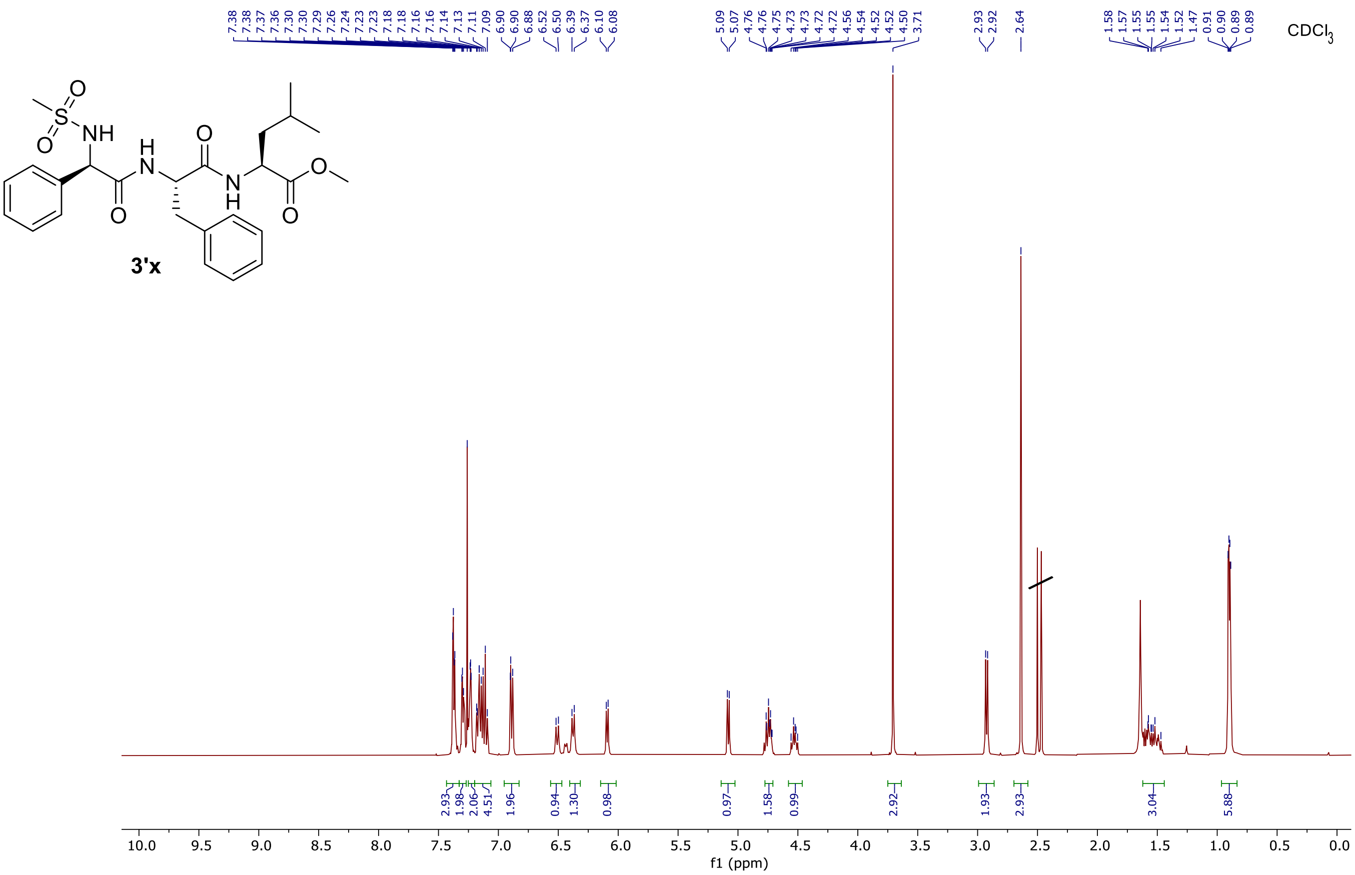

3'x

7.38 7.38 7.37 7.36 7.30 7.30 7.29 7.26 7.24 7.23 7.23 7.18 7.18 7.16 7.16 7.14 7.13 7.11 7.09 6.90 6.90 6.88 6.52 6.50 6.39 6.37 6.10 6.08
5.09 5.07 4.76 4.75 4.73 4.73 4.72 4.72 4.56 4.54 4.52 4.52 4.50 3.71
2.93 2.92 2.64
1.58 1.57 1.55 1.55 1.54 1.52 1.47 0.91 0.90 0.89 0.89
CDCl$_3$
2.93 1.98 2.06 4.51 1.96 0.94 1.30 0.98 0.97 1.58 0.99 2.92 1.93 2.93 3.04 5.88
10.0 9.5 9.0 8.5 8.0 7.5 7.0 6.5 6.0 5.5 5.0 4.5 4.0 3.5 3.0 2.5 2.0 1.5 1.0 0.5 0.0
f1 (ppm)

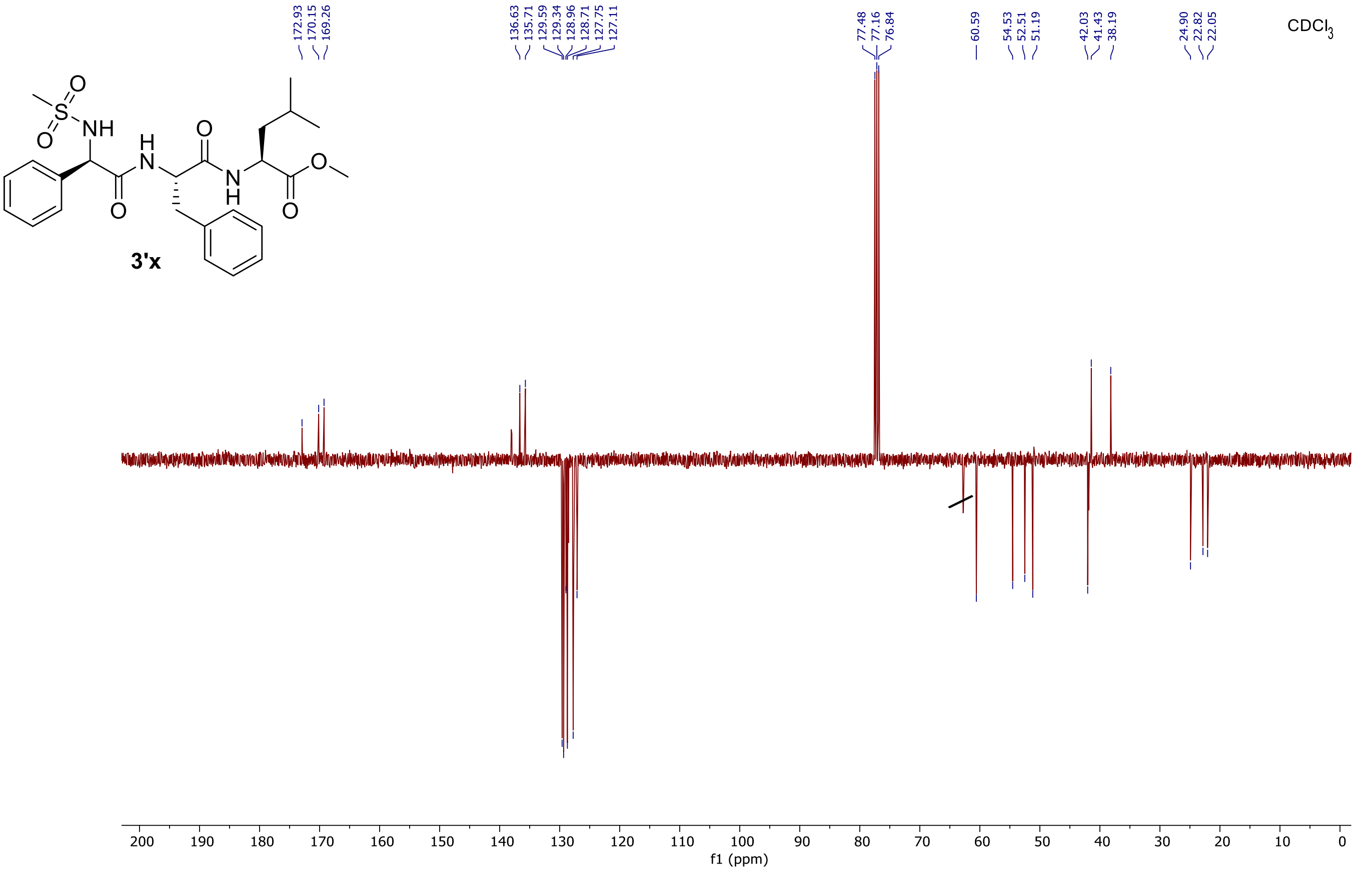

3'x

172.93 170.15 169.26
136.63 135.71 129.59 129.34 128.96 128.71 127.75 127.11
77.48 77.16 76.84
60.59 54.53 52.51 51.19
42.03 41.43 38.19
24.90 22.82 22.05
CDCl$_3$
200 190 180 170 160 150 140 130 120 110 100 90 80 70 60 50 40 30 20 10 0
f1 (ppm)

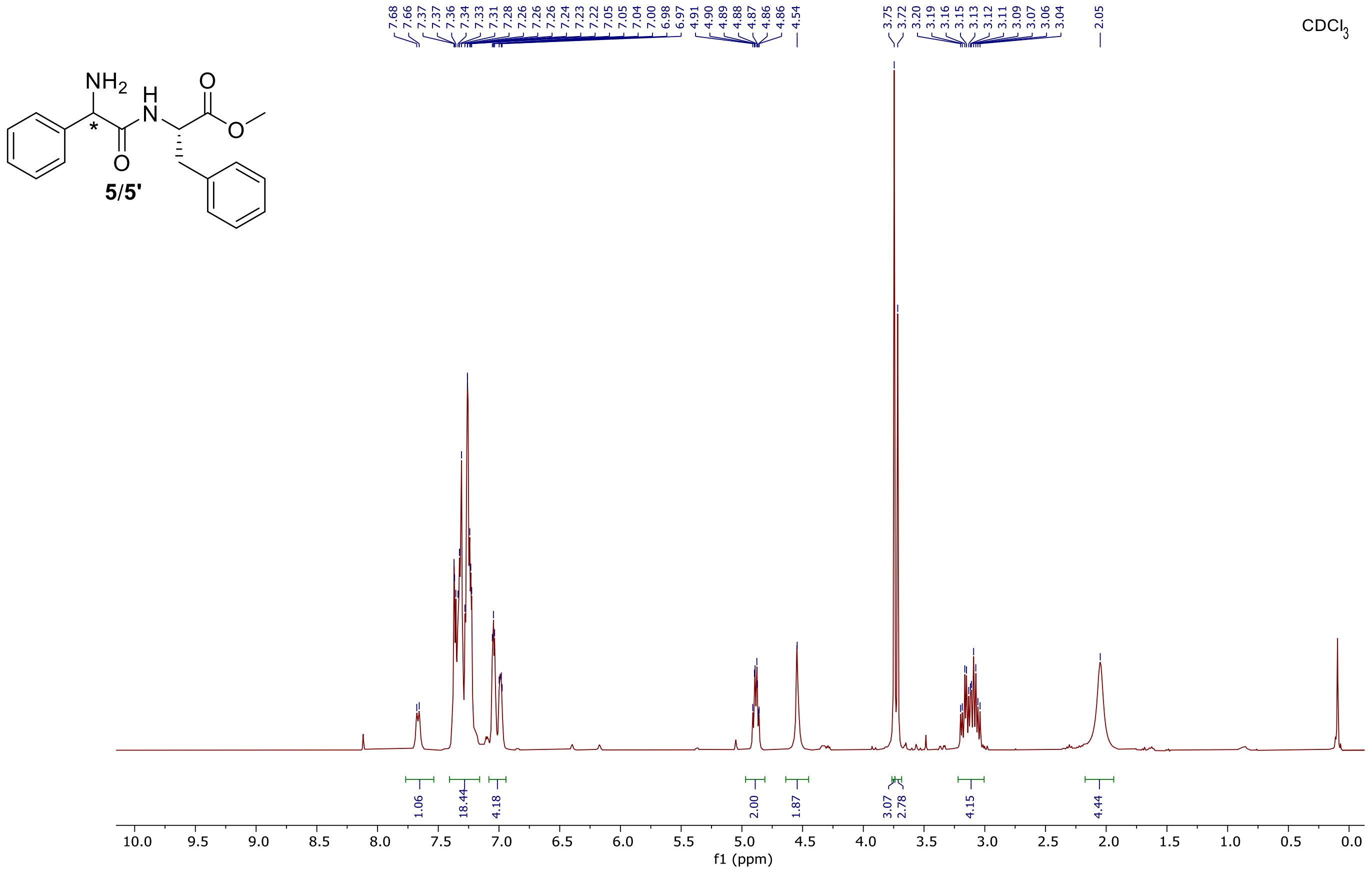

NH2
H
N
O
O
O
*
5/5'
CDCl3
7.68
7.66
7.37
7.37
7.36
7.34
7.33
7.31
7.28
7.26
7.26
7.26
7.24
7.23
7.22
7.05
7.05
7.04
7.00
6.98
6.97
4.91
4.89
4.88
4.87
4.86
4.86
4.54
3.75
3.72
3.20
3.19
3.16
3.15
3.13
3.12
3.11
3.09
3.07
3.06
3.04
2.05
1.06
18.44
4.18
2.00
1.87
3.07
2.78
4.15
4.44
10.0 9.5 9.0 8.5 8.0 7.5 7.0 6.5 6.0 5.5 5.0 4.5 4.0 3.5 3.0 2.5 2.0 1.5 1.0 0.5 0.0
f1 (ppm)

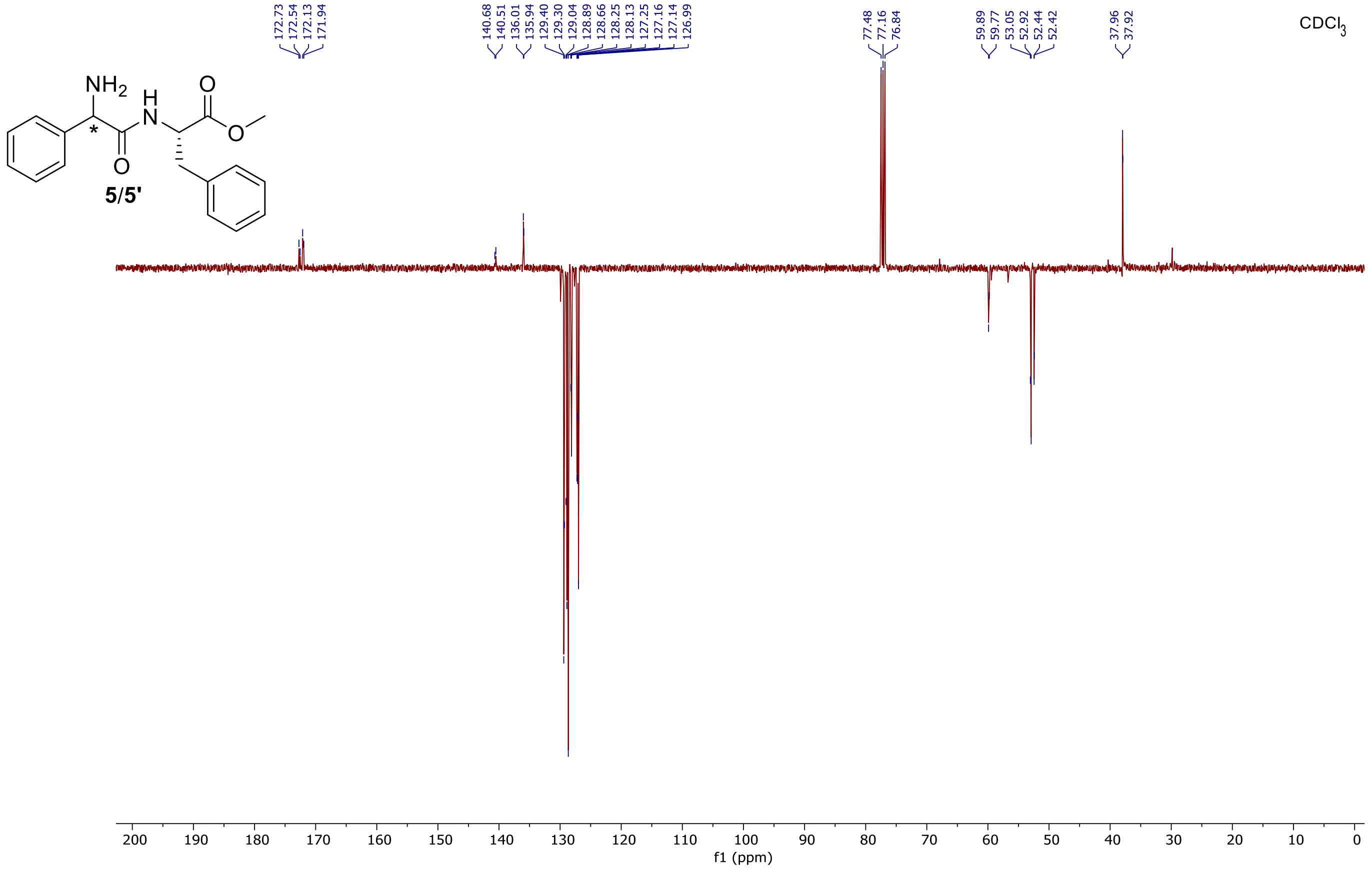

NH2
H
N
O
O
O
*
5/5'
CDCl3
172.73
172.54
172.13
171.94
140.68
140.51
136.01
135.94
129.40
129.30
129.04
128.89
128.66
128.25
128.13
127.25
127.16
127.14
126.99
77.48
77.16
76.84
59.89
59.77
53.05
52.92
52.44
52.42
37.96
37.92
200 190 180 170 160 150 140 130 120 110 100 90 80 70 60 50 40 30 20 10 0
f1 (ppm)

7. Single-crystal X-ray Diffraction Analyses of 3’b

CCDC 2342122 contain the supplementary crystallographic data for this paper. These data can be obtained free of charge from The Cambridge Crystallographic Data Centre via the following URL: www.ccdc.cam.ac.uk/structureswww.ccdc.cam.ac.uk/structures

Material appearance: colorless needles (Figure S10)
Crystallization method: slow evaporation
Sample description: needle, transparent, colourless with dimensions 0.500 x 0.150 x 0.075 mm.
Mounting: on a glass fiber, with perfluorinated oil
Comments: The sample shows pleochroism (from colorless to brown) under polarized light. It was cut from a longer rod using a cutter and polished by mechanical ablation in a drop of perfluorinated oil.

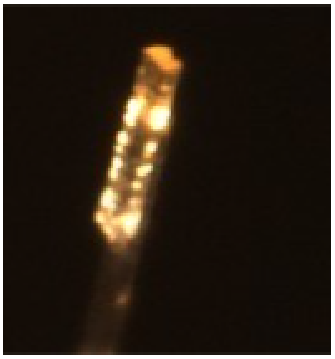

Figure S10. Crystal sample of **3’b** mounted on a glass fibre.

Instrumental specs and data collection information

Device: Rigaku XtaLAB Synergy-S 4-circle diffractometer
Source: microfocus sealed tube
Detector: Hybrid Photon Counting (HPC)
Experiment temperature: 100(2) K
Cryostat: Oxford Cryosystem1000, N_2 flow
Wavelength: Cu K_α (1.54184 Å).
Data collection extent: full sphere within $\sin\vartheta/\lambda = 0.6$ Å^{-1}
Data collections specs: Detector-to-sample distance: variable, several ω-scan run.
Measured reflections: 15620, 4133 independent
Maximum resolution (ϑ): 80.342 °
Completeness: 92.8 % (at full sphere resolution)

Data reduction programs

Integration: CrysalisPro
Reduction: CrystalsPro
Structure solution and refinement: Shelxs2014, Shelxl 2014

Unit cell, lattice and crystal system:

Bravais lattice: Monoclinic, Primitive

Space group: P 2_1, n º 4
Point group: 2
Laue group: 2/m, number 2
Unit cell (Å, deg, Å^3): a = 10.0814(4), b = 4.7978(2), c = 21.4291(7), α = 90.0, β = 95.103(3), γ = 90.0, V = 1032.39(7) as estimated from 9372 intense reflections among 4.0810 e 77.3320 deg of ϑ (final integration result).
Formula units in cell (Z): 2
Formula units in the asymmetric unit (Z'): 1
Number of electrons in cell (F_{000}): 480
Computed density: 1.510 g/cm^3
Linear absorption coefficient (μ): 3.944 mm^{-1}

Main statistical results:

Final stats for the spherical atom model (Shelxl):
Scale factor: 3.32266(5)
BASF parameter: //
Secondary extinction coefficient: none
$\langle\Delta/\sigma\rangle = 0.000$
R1(F) = 0.0864 for 4001 $F_o > 4\sigma(F_o)$, 0.0884 for all the 4133 independent data
wR(F^2)= 0.2230 for all the measured data
Goodness-of-fit: 1.042
Flack's parameter: 0.5(5)
$\Delta\rho_{MAX/MIN}$ = +2.62 e/Å^3 at ~ 0.93 Å from the C1 carbon –0.93 e/Å^3 at ~ 0.77 Å from the Br1 bromine.

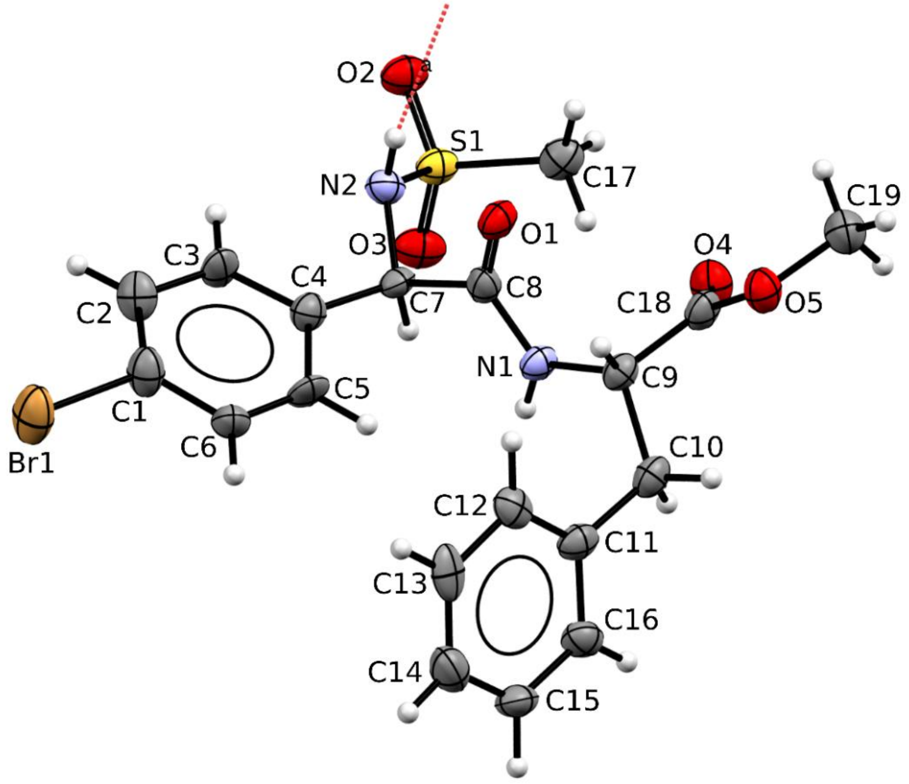

Figure S11. Asymmetric unit of **3'b** at 100 K, with the non-H atom-numbering scheme. Thermal ellipsoids of non-H atoms were drawn at the 50 % probability level. The usual color code was employed for atoms (grey: C; white: H; blue: N; red: O; yellow: S, gold: Br).

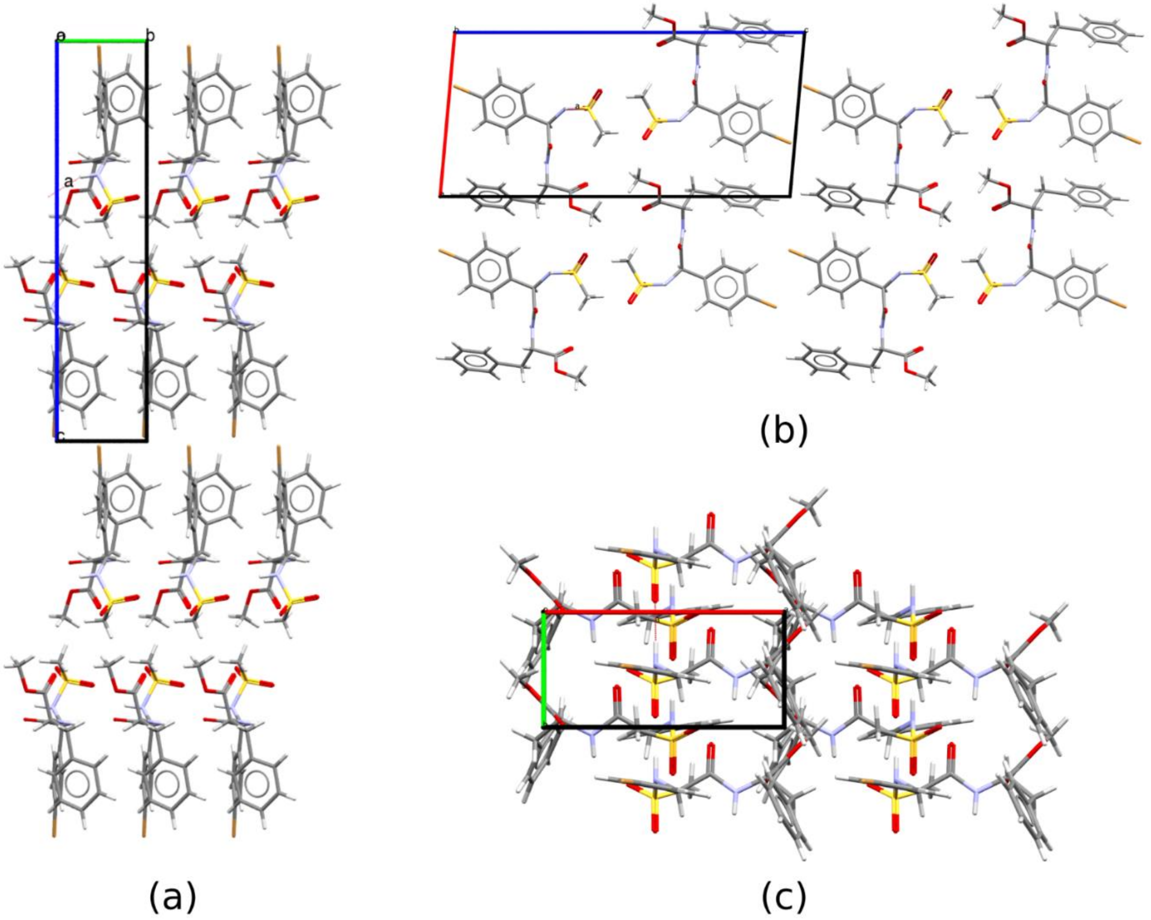

Figure S12. Crystal packing of **3'b** at 100 K, as seen (a) along the *a* cell axis; (b) the *b* cell axis; (c) the *c* cell axis. Color code as in Figure S11.

Discussion and conclusions

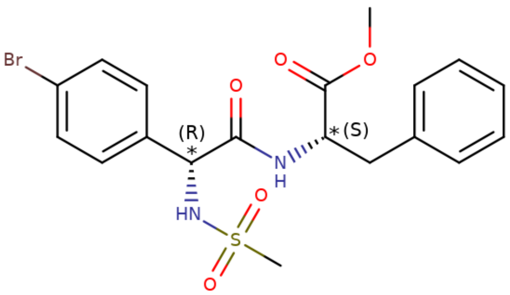

Figure S13. Molecular structure of **3'b**, with the CIP descriptors highlighted in parentheses.

The least squares statistical results reveal that the overall quality of the data collected is poor. The crystal is twinned and the software was able to detect at least two main different crystal orientations, although many more reflections were not indexed and therefore other, less important, components were also present. Since the data quality was not good enough to treat the second component in a confident way, we chose to consider only the primary component. However, the refined geometry is reliable enough to secure the chemical connectivity and the absolute stereochemistry.

The compound crystallizes in the monoclinic achiral non-centrosymmetric space group $P2_1$ (N. 4) as a *R*(C7)-*S*(C9) pure enantiomer. Figure S11 and Figure S13 show the absolute configuration of the chiral centers. Their configurational CIP descriptors are C7 (*R*) and C9 (*S*) (see Figure S11 for atom numbering).

The two phenyl rings are organized in a "cis-like" orientation with respect to the amide bond. The two phenyl rings are rotationally disordered around the C4-C1 and C11-C14 axes producing two possible configurations with a 49%-51% distribution.

Figure S12 shows the main packing motif of **3'b**. Strong hydrogen bonds (HB) are formed between molecules related by translations along the b-axis, forming infinite chains of hydrogen bonded molecules along the a-direction of the crystal.

The first HB donor atom is the N2 nitrogen, while the acceptor is the O3 oxygen bonded to the sulphur atom. The N2-H2N···O3 distance is 2.06 Å and the angle is 157°. The second type of HB is set between the N1 nitrogen and the O1 carbonyl oxygen. The N1-H1N···O1 distance is 2.29 Å and the angle is 160° (See Table S3).

Table S3. Strong N-H···O hydrogen bonds (HBs) of **3'b**.

D–H···A	$d_{D–H}$, Å	$d_{H\cdots A}$, Å	$d_{D\cdots A}$, Å	α_{DHA}, °	Symmetry operation
N2–H2N···O3	0.89(14)	2.06(14)	2.902(4)	157(10)	x, -1+y, z
N1–H1N···O1	0.76(14)	2.29(14)	3.010(10)	160(12)	+x, 1+y, z

No other strong intermolecular interactions are set-up along the other crystal directions, where only week C-H···O contacts are present. The first interaction is set between the chiral center C7 and the carbonyl O1 oxygen atom of the translated-related molecule. The second weak interaction is set between the $–CH_2$ group and the ester –O5– oxygen. The last two interactions are built between the terminal $–CH_3$ groups and the sulfonyl oxygen O2.

Two out of four interactions (see the first two contacts in Table S4) are set up between the aromatic carbon (C2) and the sp3 carbon atoms bonded to the nitrogen ones (C7 and C9) with the two C=O functional groups of the molecules. The last two interactions (C19–H19B···O3) is, on the contrary, set up between the terminal $–S(O_2)-CH_3$ methyl group and the oxygen of the sulphonyl group of another molecule obtained applying the 2_1 screw axis symmetry operator. The same happens in the opposite direction, building up a closed cyclic interaction between the two sulphonyl groups. The following table reports the geometry data for the shortest C-H···O interactions.

Table S4. Weak C-H···O interactions of **3'b**.

D–H···A	$d_{D–H}$, Å	$d_{H\cdots A}$, Å	$d_{D\cdots A}$, Å	α_{DHA}, °	Symmetry operation
C7–H7···O1	1.00	2.23	3.159(9)	154	x, -1+y, z
C10–H10A···O5	0.99	2.41	3.377(11)	165	x, -1+y, z
C17–H17B···O2	0.98	2.55	3.442(11)	152	1-x, 1/2+y, 1-z
C19–H19B···O2	0.98	2.58	3.225(12)	124	1+x, y, z

Overall, the crystal packing is dominated by strong hydrogen-bond interactions set up by the nitrogen and oxygen atoms of translational-related molecules along the b-axis, while all the other interactions are byproducts of steric packing, and they contribute only marginally to the stability of the system.

8. References

1 J. Luo, J. Zhang, *ACS Catal.* **2016**, *6*, 873-877.
2 E. Speckmeier, T. G. Fischer, K. Zeitler, *J. Am. Chem. Soc.* **2018**, *140*, 15353-15365.
3 B. T. Matsuo, P. H. R. Oliveira, J. T. M. Correia, M. W. Paixão, *Org. Lett*. **2021**, *23*, 6775-6779.
4 https://www.kessil.com/products/science_PR160L.php
5 W. C. Still, M. Kahn, A. Mitra, *J. Org. Chem.* **1978**, *43*, 2923-2925.
6 S. Morales, F. G. Guijarro, J. L. G. Ruano, B. Cid, *J. Am. Chem. Soc.* **2014**, *136*, 1082-1089.
7 C. Wang, K. Huang, J. Wang, H. Wang, L. Liu, W. Chang, J. Li, *Adv. Synth. Catal*. **2015**, *357*, 2795-2802.
8 T. Xavier, S. Condon, C. Pichon, E. Le Gall, M. Presset, *J. Org. Chem*. **2021**, *86*, 5452-5462.
9 S. M. Rafferty, J. E. Rutherford, L. Zhang, L. Wang, D. A. Nagib, *J. Am. Chem. Soc.* **2021**, *143*, 5622-5628.
10 D. Uruguchi, R. Tsutsumi, T. Ooi, *Tetrahedron*. **2014**, *70*, 1691-1701.
11 D.-J. Dong, Y. Li, J.-Q. Wang, S.-K. Tian, *Chem. Commun.* **2011**, *47*, 2158-2160.
12 S. A. Cronin, A. G. Collar, S. Gundala, C. Cornaggia, E. Torrente, F. Manoni, A. Botte, B. Twamley, S. J. Connon, *Org. Biomol. Chem*. **2016**, *14*, 6955-6959.
13 D. W. Watson, M. Gill, P. Kemmitt, S. G. Lamont, M. V. Popescu, I. Simpson, *Tetrahedron Lett.* **2018**, *59*, 4479-4482.
14 I. T. Raheem, E. N. Jacobsen, *Adv. Synth. Catal*. **2005**, *347*, 1701-1708.
15 R. Zhang, Z. Zhang, Q. Zhou, L. Yu, J. Wang, *Angew. Chem. Int. Ed*. **2019**, *58*, 5744-5748.
16 J. K. Laha, K. P. Jethava, K. S. S. Tummalapalli, S. Sharma, *Eur. J. Org. Chem*. **2017**, 4617-4624.
17 J. Xu, R. Zhuang, L. Bao, G. Tang and Y. Zhao, *Green Chem*. **2012**, *14*, 2384-2387.
18 X. Wu, L.-P. Zhao, J.-M. Xie, Y.-M. Fu, C.-F. Zhu, Y.-G. Li, *J. Org. Chem.* **2022**, *87*, 801-812.
19 N. Alandini, L. Buzzetti, G. Favi, T. Schulte, L. Candish, K. D. Collins, P. Melchiorre, *Angew. Chem. Int. Ed.* **2020**, *59*, 5248-5253.
20 T. Guo, H. Wang, C. Wang, S. Tang, J. Liu, X. Wang, *J. Org. Chem*. **2022**, *87*, 6852-6859.
21 X.-Y. Ye, G. Wang, Z. Jin, B. Yu, J. Zhang, S. Ren, Y. R. Chi, *J. Am. Chem. Soc*. **2024**, *146*, 5502-5510.
22 B. Li, J. Chen, Z. Zhang, I. D. Gridnev, W. Zhang, *Angew. Chem. Int. Ed*. **2019**, *58*, 7329-7334.
23 Y. Otomaru, N. Tokunaga, R. Shintani, T. Hayashi, *Org. Lett*. **2005**, *7*, 307-310.
24 J. Brom, A. Maruani, L. Micouin, E. Benedetti, *J. Org. Chem.* **2023**, *88*, 5923-5935.
25 S. G. Ouellet, A. M. Walji, D. W. C. Macmillan, *Acc. Chem. Res*. **2007**, *40*, 1327-1339.
26 D. Uraguchi, N. Kinoshita, T. Kizu, T. Ooi, *J. Am. Chem. Soc.* **2015**, *137*, 13768-13771.
